# Supplementary material for: Discovery and application of insertion-deletion (INDEL) polymorphisms for QTL mapping of early life-history traits in Atlantic salmon
Source: BMC Genomics. 2010 Mar 8;11:156. doi: 10.1186/1471-2164-11-156 (PMC2838853; doi:10.1186/1471-2164-11-156)
Supplement: Additional file 2 — Information on developed 76 locus single-run INDEL panel in Atlantic salmon. Information on fluorescence labeling, primer concentrations, PCR pooling and links to alignments, INDEL motifs and GENESCAN (Burge and Karlin 1997) predictions of genes/exons are available in html format. [file 1471-2164-11-156-S2.ZIP › Additionalfile2/table32.html]

Contig Alignment

```
Base	A B C D E F G H I J K L M N O P Q R S T U V W X Y Z a b c d e f g h i j k l m n o p q r s t u v w x y z A B C D E F G H I J K L M N O P Q R S T U V W X Y Z a b c d e f g h i j k l m n o p q r s t u v w x y z A B C D E F G H I J K L M N O P Q R S T U V W X Y Z a b c d e f g h i j k l m n o p q r s t u v w x y z A B C D E F G H I J K L M N O P Q R S T U V W X Y Z a b c   
1	. . . . . . . . . . G . . . . . . . . . . . . . . . . . . . . . . . . . . . . . . . . . . . . . . . . . . . . . . . . . . . . . . . . . . . . . . . . . . . . . . . . . . . . . . . . . . . . . . . . . . . . . . . . . . . . . . . . . . . . . . . . . . . . . . . . . . . . . . . . . . . . . . . . . . . . . . . . . . . . . . . . . . . . . . . . . . . . . . . . . . . . . .    
2	. . . . . . . . . . G . . . . . . . . . . . . . . . . . . . . . . . . . . . . . . . . . . . . . . . . . . . . . . . . . . . . . . . . . . . . . . . . . . . . . . . . . . . . . . . . . . . . . . . . . . . . . . . . . . . . . . . . . . . . . . . . . . . . . . . . . . . . . . . . . . . . . . . . . . . . . . . . . . . . . . . . . . . . . . . . . . . . . . . . . . . . . .    
3	. . . . . . . . . . G . . . . . . . . . . . . . . . . . . . . . . . . . . . . . . . . . . . . . . . . . . . . . . . . . . . . . . . . . . . . . . . . . . . . . . . . . . . . . . . . . . . . . . . . . . . . . . . . . . . . . . . . . . . . . . . . . . . . . . . . . . . . . . . . . . . . . . . . . . . . . . . . . . . . . . . . . . . . . . . . . . . . . . . . . . . . . .    
4	. . . . . . . . . . G . . . . . . . . . . . . . . . . . . . . . . . . . . . . . . . . . . . . . . . . . . . . . . . . . . . . . . . . . . . . . . . . . . . . . . . . . . . . . . . . . . . . . . . . . . . . . . . . . . . . . . . . . . . . . . . . . . . . . . . . . . . . . . . . . . . . . . . . . . . . . . . . . . . . . . . . . . . . . . . . . . . . . . . . . . . . . .    
5	. . . . . . . . . . G . . . . . . . . . . . . . . . . . . . . . . . . . . . . . . . . . . . . . . . . . . . . . . . . . . . . . . . . . . . . . . . . . . . . . . . . . . . . . . . . . . . . . . . . . . . . . . . . . . . . . . . . . . . . . . . . . . . . . . . . . . . . . . . . . . . . . . . . . . . . . . . . . . . . . . . . . . . . . . . . . . . . . . . . . . . . . .    
6	. . . . . . . . . . A . . . . . . . . . . . . . . . . . . . . . . . . . . . . . . . . . . . . . . . . . . . . . . . . . . . . . . . . . . . . . . . . . . . . . . . . . . . . . . . . . . . . . . . . . . . . . . . . . . . . . . . . . . . . . . . . . . . . . . . . . . . . . . . . . . . . . . . . . . . . . . . . . . . . . . . . . . . . . . . . . . . . . . . . . . . . . .    
7	. . . . . . . . . . A . . . . . . . . . . . . . . . . . . . . . . . . . . . . . . . . . . . . . . . . . . . . . . . . . . . . . . . . . . . . . . . . . . . . . . . . . . . . . . . . . . . . . . . . . . . . . . . . . . . . . . . . . . . . . . . . . . . . . . . . . . . . . . . . . . . . . . . . . . . . . . . . . . . . . . . . . . . . . . . . . . . . . . . . . . . . . .    
8	. . . . . . . . . . A . . . . . . . . . . . . . . . . . . . . . . . . . . . . . . . . . . . . . . . . . . . . . . . . . . . . . . . . . . . . . . . . . . . . . . . . . . . . . . . . . . . . . . . . . . . . . . . . . . . . . . . . . . . . . . . . . . . . . . . . . . . . . . . . . . . . . . . . . . . . . . . . . . . . . . . . . . . . . . . . . . . . . . . . . . . . . .    
9	. . . . . . . . . . T . . . . . . . . . . . . . . . . . . . . . . . . . . . . . . . . . . . . . . . . . . . . . . . . . . . . . . . . . . . . . . . . . . . . . . . . . . . . . . . . . . . . . . . . . . . . . . . . . . . . . . . . . . . . . . . . . . . . . . . . . . . . . . . . . . . . . . . . . . . . . . . . . . . . . . . . . . . . . . . . . . . . . . . . . . . . . .    
10	. . . . . . . . . . A . . . . . . . . . . . . . . . . . . . . . . . . . . . . . . . . . . . . . . . . . . . . . . . . . . . . . . . . . . . . . . . . . . . . . . . . . . . . . . . . . . . . . . . . . . . . . . . . . . . . . . . . . . . . . . . . . . . . . . . . . . . . . . . . . . . . . . . . . . . . . . . . . . . . . . . . . . . . . . . . . . . . . . . . . . . . . .    
11	. . . . . . . . . . A . . . . . . . . . . . . . . . . . . . . . . . . . . . . . . . . . . . . . . . . . . . . . . . . . . . . . . . . . . . . . . . . . . . . . . . . . . . . . . . . . . . . . . . . . . . . . . . . . . . . . . . . . . . . . . . . . . . . . . . . . . . . . . . . . . . . . . . . . . . . . . . . . . . . . . . . . . . . . . . . . . . . . . . . . . . . . .    
12	. . . . . . . . . . A . . . . . . . . . . . . . . . . . . . . . . . . . . . . . . . . . . . . . . . . . . . . . . . . . . . . . . . . . . . . . . . . . . . . . . . . . . . . . . . . . . . . . . . . . . . . . . . . . . . . . . . . . . . . . . . . . . . . . . . . . . . . . . . . . . . . . . . . . . . . . . . . . . . . . . . . . . . . . . . . . . . . . . . . . . . . . .    
13	. . . . . . . . . . A . . . . . . . . . . . . . . . . . . . . . . . . . . . . . . . . . . . . . . . . . . . . . . . . . . . . . . . . . . . . . . . . . . . . . . . . . . . . . . . . . . . . . . . . . . . . . . . . . . . . . . . . . . . . . . . . . . . . . . . . . . . . . . . . . . . . . . . . . . . . . . . . . . . . . . . . . . . . . . . . . . . . . . . . . . . . . .    
14	. . . . . . . . . . T . . . . . . . . . . . . . . . . . . . . . . . . . . . . . . . . . . . . . . . . . . . . . . . . . . . . . . . . . . . . . . . . . . . . . . . . . . . . . . . . . . . . . . . . . . . . . . . . . . . . . . . . . . . . . . . . . . . . . . . . . . . . . . . . . . . . . . . . . . . . . . . . . . . . . . . . . . . . . . . . . . . . . . . . . . . . . .    
15	. . . . . . . . . . A . . . . . . . . . . . . . . . . . . . . . . . . . . . . . . . . . . . . . . . . . . . . . . . . . . . . . . . . . . . . . . . . . . . . . . . . . . . . . . . . . . . . . . . . . . . . . . . . . . . . . . . . . . . . . . . . . . . . . . . . . . . . . . . . . . . . . . . . . . . . . . . . . . . . . . . . . . . . . . . . . . . . . . . . . . . . . .    
16	. . . . . . . . . . T . . . . . . . . . . . . . . . . . . . . . . . . . . . . . . . . . . . . . . . . . . . . . . . . . . . . . . . . . . . . . . . . . . . . . . . . . . . . . . . . . . . . . . . . . . . . . . . . . . . . . . . . . . . . . . . . . . . . . . . . . . . . . . . . . . . . . . . . . . . . . . . . . . . . . . . . . . . . . . . . . . . . . . . . . . . . . .    
17	. . . . . . . . . . G . . . . . . . . . . . . . . . . . . . . . . . . . . . . . . . . . . . . . . . . . . . . . . . . . . . . . . . . . . . . . . . . . . . . . . . . . . . . . . . . . . . . . . . . . . . . . . . . . . . . . . . . . . . . . . . . . . . . . . . . . . . . . . . . . . . . . . . . . . . . . . . . . . . . . . . . . . . . . . . . . . . . . . . . . . . . . .    
18	. . . . . . . . . . C . . . . . . . . . . . . . . . . . . . . . . . . . . . . . . . . . . . . . . . . . . . . . . . . . . . . . . . . . . . . . . . . . . . . . . . . . . . . . . . . . . . . . . . . . . . . . . . . . . . . . . . . . . . . . . . . . . . . . . . . . . . . . . . . . . . . . . . . . . . . . . . . . . . . . . . . . . . . . . . . . . . . . . . . . . . . . .    
19	. . . . . . . . . . C . . . . . . . . . . . . . . . . . . . . . . . . . . . . . . . . . . . . . . . . . . . . . . . . . . . . . . . . . . . . . . . . . . . . . . . . . . . . . . . . . . . . . . . . . . . . . . . . . . . . . . . . . . . . . . . . . . . . . . . . . . . . . . . . . . . . . . . . . . . . . . . . . . . . . . . . . . . . . . . . . . . . . . . . . . . . . .    
20	. . . . . . . . . . A . . . . . . . . . . . . . . . . . . . . . . . . . . . . . . . . . . . . . . . . . . . . . . . . . . . . . . . . . . . . . . . . . . . . . . . . . . . . . . . . . . . . . . . . . . . . . . . . . . . . . . . . . . . . . . . . . . . . . . . . . . . . . . . . . . . . . . . . . . . . . . . . . . . . . . . . . . . . . . . . . . . . . . . . . . . . . .    
21	. . . . . . . . . . T . . . . . . . . . . . . . . . . . . . . . . . . . . . . . . . . . . . . . . . . . . . . . . . . . . . . . . . . . . . . . . . . . . . . . . . . . . . . . . . . . . . . . . . . . . . . . . . . . . . . . . . . . . . . . . . . . . . . . . . . . . . . . . . . . . . . . . . . . . . . . . . . . . . . . . . . . . . . . . . . . . . . . . . . . . . . . .    
22	. . . . . . . . . . C . . . . . . . . . . . . . . . . . . . . . . . . . . . . . . . . . . . . . . . . . . . . . . . . . . . . . . . . . . . . . . . . . . . . . . . . . . . . . . . . . . . . . . . . . . . . . . . . . . . . . . . . . . . . . . . . . . . . . . . . . . . . . . . . . . . . . . . . . . . . . . . . . . . . . . . . . . . . . . . . . . . . . . . . . . . . . .    
23	. . . . . . . . . . A . . . . . . . . . . . . . . . . . . . . . . . . . . . . . . . . . . . . . . . . . . . . . . . . . . . . . . . . . . . . . . . . . . . . . . . . . . . . . . . . . . . . . . . . . . . . . . . . . . . . . . . . . . . . . . . . . . . . . . . . . . . . . . . . . . . . . . . . . . . . . . . . . . . . . . . . . . . . . . . . . . . . . . . . . . . . . .    
24	. . . . . . . . . . A . . . . . . . . . . . . . . . . . . . . . . . . . . . . . . . . . . . . . . . . . . . . . . . . . . . . . . . . . . . . . . . . . . . . . . . . . . . . . . . . . . . . . . . . . . . . . . . . . . . . . . . . . . . . . . . . . . . . . . . . . . . . . . . . . . . . . . . . . . . . . . . . . . . . . . . . . . . . . . . . . . . . . . . . . . . . . .    
25	. . . . . . . . . . C . . . . . . . . . . . . . . . . . . . . . . . . . . . . . . . . . . . . . . . . . . . . . . . . . . . . . . . . . . . . . . . . . . . . . . . . . . . . . . . . . . . . . . . . . . . . . . . . . . . . . . . . . . . . . . . . . . . . . . . . . . . . . . . . . . . . . . . . . . . . . . . . . . . . . . . . . . . . . . . . . . . . . . . . . . . . . .    
26	. . . . . . . . . . A . . . . . . . . . . . . . . . . . . . . . . . . . . . . . . . . . . . . . . . . . . . . . . . . . . . . . . . . . . . . . . . . . . . . . . . . . . . . . . . . . . . . . . . . . . . . . . . . . . . . . . . . . . . . . . . . . . . . . . . . . . . . . . . . . . . . . . . . . . . . . . . . . . . . . . . . . . . . . . . . . . . . . . . . . . . . . .    
27	. . . . . . . . . . A . . . . . . . . . . . . . . . . . . . . . . . . . . . . . . . . . . . . . . . . . . . . . . . . . . . . . . . . . . . . . . . . . . . . . . . . . . . . . . . . . . . . . . . . . . . . . . . . . . . . . . . . . . . . . . . . . . . . . . . . . . . . . . . . . . . . . . . . . . . . . . . . . . . . . . . . . . . . . . . . . . . . . . . . . . . . . .    
28	. . . . . . . . . . C . . . . . . . . . . . . . . . . . . . . . . . . . . . . . . . . . . . . . . . . . . . . . . . . . . . . . . . . . . . . . . . . . . . . . . . . . . . . . . . . . . . . . . . . . . . . . . . . . . . . . . . . . . . . . . . . . . . . . . . . . . . . . . . . . . . . . . . . . . . . . . . . . . . . . . . . . . . . . . . . . . . . . . . . . . . . . .    
29	. . . . . . . . . . T . . . . . . . . . . . . . . . . . . . . . . . . . . . . . . . . . . . . . . . . . . . . . . . . . . . . . . . . . . . . . . . . . . . . . . . . . . . . . . . . . . . . . . . . . . . . . . . . . . . . . . . . . . . . . . . . . . . . . . . . . . . . . . . . . . . . . . . . . . . . . . . . . . . . . . . . . . . . . . . . . . . . . . . . . . . . . .    
30	. . . . . . . . . . G . . . . . . . . . . . . . . . . . . . . . . . . . . . . . . . . . . . . . . . . . . . . . . . . . . . . . . . . . . . . . . . . . . . . . . . . . . . . . . . . . . . . . . . . . . . . . . . . . . . . . . . . . . . . . . . . . . . . . . . . . . . . . . . . . . . . . . . . . . . . . . . . . . . . . . . . . . . . . . . . . . . . . . . . . . . . . .    
31	. . . . . . . . . . C . . . . . . . . . . . . . . . . . . . . . . . . . . . . . . . . . . . . . . . . . . . . . . . . . . . . . . . . . . . . . . . . . . . . . . . . . . . . . . . . . . . . . . . . . . . . . . . . . . . . . . . . . . . . . . . . . . . . . . . . . . . . . . . . . . . . . . . . . . . . . . . . . . . . . . . . . . . . . . . . . . . . . . . . . . . . . .    
32	. . . . . . . . . . T . . . . . . . . . . . . . . . . . . . . . . . . . . . . . . . . . . . . . . . . . . . . . . . . . . . . . . . . . . . . . . . . . . . . . . . . . . . . . . . . . . . . . . . . . . . . . . . . . . . . . . . . . . . . . . . . . . . . . . . . . . . . . . . . . . . . . . . . . . . . . . . . . . . . . . . . . . . . . . . . . . . . . . . . . . . . . .    
33	. . . . . . . . . . G . . . . . . . . . . . . . . . . . . . . . . . . . . . . . . . . . . . . . . . . . . . . . . . . . . . . . . . . . . . . . . . . . . . . . . . . . . . . . . . . . . . . . . . . . . . . . . . . . . . . . . . . . . . . . . . . . . . . . . . . . . . . . . . . . . . . . . . . . . . . . . . . . . . . . . . . . . . . . . . . . . . . . . . . . . . . . .    
34	. . . . . . . . . . A . . . . . . . . . . . . . . . . . . . . . . . . . . . . . . . . . . . . . . . . . . . . . . . . . . . . . . . . . . . . . . . . . . . . . . . . . . . . . . . . . . . . . . . . . . . . . . . . . . . . . . . . . . . . . . . . . . . . . . . . . . . . . . . . . . . . . . . . . . . . . . . . . . . . . . . . . . . . . . . . . . . . . . . . . . . . . .    
35	. . . . . . . . . . C . . . . . . . . . . . . . . . . . . . . . . . . . . . . . . . . . . . . . . . . . . . . . . . . . . . . . . . . . . . . . . . . . . . . . . . . . . . . . . . . . . . . . . . . . . . . . . . . . . . . . . . . . . . . . . . . . . . . . . . . . . . . . . . . . . . . . . . . . . . . . . . . . . . . . . . . . . . . . . . . . . . . . . . . . . . . . .    
36	. . . . . . . . . . G . . . . . . . . . . . . . . . . . . . . . . . . . . . . . . . . . . . . . . . . . . . . . . . . . . . . . . . . . . . . . . . . . . . . . . . . . . . . . . . . . . . . . . . . . . . . . . . . . . . . . . . . . . . . . . . . . . . . . . . . . . . . . . . . . . . . . . . . . . . . . . . . . . . . . . . . . . . . . . . . . . . . . . . . . . . . . .    
37	. . . . . . . . . . C . . . . . . . . . . . . . . . . . . . . . . . . . . . . . . . . . . . . . . . . . . . . . . . . . . . . . . . . . . . . . . . . . . . . . . . . . . . . . . . . . . . . . . . . . . . . . . . . . . . . . . . . . . . . . . . . . . . . . . . . . . . . . . . . . . . . . . . . . . . . . . . . . . . . . . . . . . . . . . . . . . . . . . . . . . . . . .    
38	. . . . . . . . . . T . . . . . . . . . . . . . . . . . . . . . . . . . . . . . . . . . . . . . . . . . . . . . . . . . . . . . . . . . . . . . . . . . . . . . . . . . . . . . . . . . . . . . . . . . . . . . . . . . . . . . . . . . . . . . . . . . . . . . . . . . . . . . . . . . . . . . . . . . . . . . . . . . . . . . . . . . . . . . . . . . . . . . . . . . . . . . .    
39	. . . . . . . . . . G . . . . . . . . . . . . . . . . . . . . . . . . . . . . . . . . . . . . . . . . . . . . . . . . . . . . . . . . . . . . . . . . . . . . . . . . . . . . . . . . . . . . . . . . . . . . . . . . . . . . . . . . . . . . . . . . . . . . . . . . . . . . . . . . . . . . . . . . . . . . . . . . . . . . . . . . . . . . . . . . . . . . . . . . . . . . . .    
40	. . . . . . . . . . A . . . . . . . . . . . . . . . . . . . . . . . . . . . . . . . . . . . . . . . . . . . . . . . . . . . . . . . . . . . . . . . . . . . . . . . . . . . . . . . . . . . . . . . . . . . . . . . . . . . . . . . . . . . . . . . . . . . . . . . . . . . . . . . . . . . . . . . . . . . . . . . . . . . . . . . . . . . . . . . . . . . . . . . . . . . . . .    
41	. . . . . . . . . . T . . . . . . . . . . . . . . . . . . . . . . . . . . . . . . . . . . . . . . . . . . . . . . . . . . . . . . . . . . . . . . . . . . . . . . . . . . . . . . . . . . . . . . . . . . . . . . . . . . . . . . . . . . . . . . . . . . . . . . . . . . . . . . . . . . . . . . . . . . . . . . . . . . . . . . . . . . . . . . . . . . . . . . . . . . . . . .    
42	. . . . . . . . . . C . . . . . . . . . . . . . . . . . . . . . . . . . . . . . . . . . . . . . . . . . . . . . . . . . . . . . . . . . . . . . . . . . . . . . . . . . . . . . . . . . . . . . . . . . . . . . . . . . . . . . . . . . . . . . . . . . . . . . . . . . . . . . . . . . . . . . . . . . . . . . . . . . . . . . . . . . . . . . . . . . . . . . . . . . . . . . .    
43	. . . . . . . . . . A . . . . . . . . . . . . . . . . . . . . . . . . . . . . . . . . . . . . . . . . . . . . . . . . . . . . . . . . . . . . . . . . . . . . . . . . . . . . . . . . . . . . . . . . . . . . . . . . . . . . . . . . . . . . . . . . . . . . . . . . . . . . . . . . . . . . . . . . . . . . . . . . . . . . . . . . . . . . . . . . . . . . . . . . . . . . . .    
44	. . . . . . . . . . A . . . . . . . . . . . . . . . . . . . . . . . . . . . . . . . . . . . . . . . . . . . . . . . . . . . . . . . . . . . . . . . . . . . . . . . . . . . . . . . . . . . . . . . . . . . . . . . . . . . . . . . . . . . . . . . . . . . . . . . . . . . . . . . . . . . . . . . . . . . . . . . . . . . . . . . . . . . . . . . . . . . . . . . . . . . . . .    
45	. . . . . . . . . . G . . . . . . . . . . . . . . . . . . . . . . . . . . . . . . . . . . . . . . . . . . . . . . . . . . . . . . . . . . . . . . . . . . . . . . . . . . . . . . . . . . . . . . . . . . . . . . . . . . . . . . . . . . . . . . . . . . . . . . . . . . . . . . . . . . . . . . . . . . . . . . . . . . . . . . . . . . . . . . . . . . . . . . . . . . . . . .    
46	. . . . . . . . . . T . . . . . . . . . . . . . . . . . . . . . . . . . . . . . . . . . . . . . . . . . . . . . . . . . . . . . . . . . . . . . . . . . . . . . . . . . . . . . . . . . . . . . . . . . . . . . . . . . . . . . . . . . . . . . . . . . . . . . . . . . . . . . . . . . . . . . . . . . . . . . . . . . . . . . . . . . . . . . . . . . . . . . . . . . . . . . .    
47	. . . . . . . . . . C . . . . . . . . . . . . . . . . . . . . . . . . . . . . . . . . . . . . . . . . . . . . . . . . . . . . . . . . . . . . . . . . . . . . . . . . . . . . . . . . . . . . . . . . . . . . . . . . . . . . . . . . . . . . . . . . . . . . . . . . . . . . . . . . . . . . . . . . . . . . . . . . . . . . . . . . . . . . . . . . . . . . . . . . . . . . . .    
48	. . . . . . . . . . A . . . . . . . . . . . . . . . . . . . . . . . . . . . . . . . . . . . . . . . . . . . . . . . . . . . . . . . . . . . . . . . . . . . . . . . . . . . . . . . . . . . . . . . . . . . . . . . . . . . . . . . . . . . . . . . . . . . . . . . . . . . . . . . . . . . . . . . . . . . . . . . . . . . . . . . . . . . . . . . . . . . . . . . . . . . . . .    
49	. . . . . . . . . . G . . . . . . . . . . . . . . . . . . . . . . . . . . . . . . . . . . . . . . . . . . . . . . . . . . . . . . . . . . . . . . . . . . . . . . . . . . . . . . . . . . . . . . . . . . . . . . . . . . . . . . . . . . . . . . . . . . . . . . . . . . . . . . . . . . . . . . . . . . . . . . . . . . . . . . . . . . . . . . . . . . . . . . . . . . . . . .    
50	. . . . . . . . . . C . . . . . . . . . . . . . . . . . . . . . . . . . . . . . . . . . . . . . . . . . . . . . . . . . . . . . . . . . . . . . . . . . . . . . . . . . . . . . . . . . . . . . . . . . . . . . . . . . . . . . . . . . . . . . . . . . . . . . . . . . . . . . . . . . . . . . . . . . . . . . . . . . . . . . . . . . . . . . . . . . . . . . . . . . . . . . .    
51	. . . . . . . . . . C . . . . . . . . . . . . . . . . . . . . . . . . . . . . . . . . . . . . . . . . . . . . . . . . . . . . . . . . . . . . . . . . . . . . . . . . . . . . . . . . . . . . . . . . . . . . . . . . . . . . . . . . . . . . . . . . . . . . . . . . . . . . . . . . . . . . . . . . . . . . . . . . . . . . . . . . . . . . . . . . . . . . . . . . . . . . . .    
52	. . . . . . . . . . A . . . . . . . . . . . . . . . . . . . . . . . . . . . . . . . . . . . . . . . . . . . . . . . . . . . . . . . . . . . . . . . . . . . . . . . . . . . . . . . . . . . . . . . . . . . . . . . . . . . . . . . . . . . . . . . . . . . . . . . . . . . . . . . . . . . . . . . . . . . . . . . . . . . . . . . . . . . . . . . . . . . . . . . . . . . . . .    
53	. . . . . . . . . . A . . . . . . . . . . . . . . . . . . . . . . . . . . . . . . . . . . . . . . . . . . . . . . . . . . . . . . . . . . . . . . . . . . . . . . . . . . . . . . . . . . . . . . . . . . . . . . . . . . . . . . . . . . . . . . . . . . . . . . . . . . . . . . . . . . . . . . . . . . . . . . . . . . . . . . . . . . . . . . . . . . . . . . . . . . . . . .    
54	. . . . . . . . . . T . . . . . . . . . . . . . . . . . . . . . . . . . . . . . . . . . . . . . . . . . . . . . . . . . . . . . . . . . . . . . . . . . . . . . . . . . . . . . . . . . . . . . . . . . . . . . . . . . . . . . . . . . . . . . . . . . . . . . . . . . . . . . . . . . . . . . . . . . . . . . . . . . . . . . . . . . . . . . . . . . . . . . . . . . . . . . .    
55	. . . . . . . . . . C . . . . . . . . . . . . . . . . . . . . . . . . . . . . . . . . . . . . . . . . . . . . . . . . . . . . . . . . . . . . . . . . . . . . . . . . . . . . . . . . . . . . . . . . . . . . . . . . . . . . . . . . . . . . . . . . . . . . . . . . . . . . . . . . . . . . . . . . . . . . . . . . . . . . . . . . . . . . . . . . . . . . . . . . . . . . . .    
56	. . . . . . . . . . A . . . . . . . . . . . . . . . . . . . . . . . . . . . . . . . . . . . . . . . . . . . . . . . . . . . . . . . . . . . . . . . . . . . . . . . . . . . . . . . . . . . . . . . . . . . . . . . . . . . . . . . . . . . . . . . . . . . . . . . . . . . . . . . . . . . . . . . . . . . . . . . . . . . . . . . . . . . . . . . . . . . . . . . . . . . . . .    
57	. . . . . . . . . . G . . . . . . . . . . . . . . . . . . . . . . . . . . . . . . . . . . . . . . . . . . . . . . . . . . . . . . . . . . . . . . . . . . . . . . . . . . . . . . . . . . . . . . . . . . . . . . . . . . . . . . . . . . . . . . . . . . . . . . . . . . . . . . . . . . . . . . . . . . . . . . . . . . . . . . . . . . . . . . . . . . . . . . . . . . . . . .    
58	. . . . . . . . . . A . . . . . . . . . . . . . . . . . . . . . . . . . . . . . . . . . . . . . . . . . . . . . . . . . . . . . . . . . . . . . . . . . . . . . . . . . . . . . . . . . . . . . . . . . . . . . . . . . . . . . . . . . . . . . . . . . . . . . . . . . . . . . . . . . . . . . . . . . . . . . . . . . . . . . . . . . . . . . . . . . . . . . . . . . . . . . .    
59	. . . . . . . . . . A . . . . . . . . . . . . . . . . . . . . . . . . . . . . . . . . . . . . . . . . . . . . . . . . . . . . . . . . . . . . . . . . . . . . . . . . . . . . . . . . . . . . . . . . . . . . . . . . . . . . . . . . . . . . . . . . . . . . . . . . . . . . . . . . . . . . . . . . . . . . . . . . . . . . . . . . . . . . . . . . . . . . . . . . . . . . . .    
60	. . . . . . . . . . A . . . . . . . . . . . . . . . . . . . . . . . . . . . . . . . . . . . . . . . . . . . . . . . . . . . . . . . . . . . . . . . . . . . . . . . . . . . . . . . . . . . . . . . . . . . . . . . . . . . . . . . . . . . . . . . . . . . . . . . . . . . . . . . . . . . . . . . . . . . . . . . . . . . . . . . . . . . . . . . . . . . . . . . . . . . . . .    
61	. . . . . . . . . . T . . . . . . . . . . . . . . . . . . . . . . . . . . . . . . . . . . . . . . . . . . . . . . . . . . . . . . . . . . . . . . . . . . . . . . . . . . . . . . . . . . . . . . . . . . . . . . . . . . . . . . . . . . . . . . . . . . . . . . . . . . . . . . . . . . . . . . . . . . . . . . . . . . . . . . . . . . . . . . . . . . . . . . . . . . . . . .    
62	. . . . . . . . . . C . . . . . . . . . . . . . . . . . . . . . . . . . . . . . . . . . . . . . . . . . . . . . . . . . . . . . . . . . . . . . . . . . . . . . . . . . . . . . . . . . . . . . . . . . . . . . . . . . . . . . . . . . . . . . . . . . . . . . . . . . . . . . . . . . . . . . . . . . . . . . . . . . . . . . . . . . . . . . . . . . . . . . . . . . . . . . .    
63	. . . . . . . . . . A . . . . . . . . . . . . . . . . . . . . . . . . . . . . . . . . . . . . . . . . . . . . . . . . . . . . . . . . . . . . . . . . . . . . . . . . . . . . . . . . . . . . . . . . . . . . . . . . . . . . . . . . . . . . . . . . . . . . . . . . . . . . . . . . . . . . . . . . . . . . . . . . . . . . . . . . . . . . . . . . . . . . . . . . . . . . . .    
64	. . . . . . . . . . T . . . . . . . . . . . . . . . . . . . . . . . . . . . . . . . . . . . . . . . . . . . . . . . . . . . . . . . . . . . . . . . . . . . . . . . . . . . . . . . . . . . . . . . . . . . . . . . . . . . . . . . . . . . . . . . . . . . . . . . . . . . . . . . . . . . . . . . . . . . . . . . . . . . . . . . . . . . . . . . . . . . . . . . . . . . . . .    
65	. . . . . . . . . . T . . . . . . . . . . . . . . . . . . . . . . . . . . . . . . . . . . . . . . . . . . . . . . . . . . . . . . . . . . . . . . . . . . . . . . . . . . . . . . . . . . . . . . . . . . . . . . . . . . . . . . . . . . . . . . . . . . . . . . . . . . . . . . . . . . . . . . . . . . . . . . . . . . . . . . . . . . . . . . . . . . . . . . . . . . . . . .    
66	. . . . . . . . . . T . . . . . . . . . . . . . . . . . . . . . . . . . . . . . . . . . . . . . . . . . . . . . . . . . . . . . . . . . . . . . . . . . . . . . . . . . . . . . . . . . . . . . . . . . . . . . . . . . . . . . . . . . . . . . . . . . . . . . . . . . . . . . . . . . . . . . . . . . . . . . . . . . . . . . . . . . . . . . . . . . . . . . . . . . . . . . .    
67	. . . . . . . . . . A . . . . . . . . . . . . . . . . . . . . . . . . . . . . . . . . . . . . . . . . . . . . . . . . . . . . . . . . . . . . . . . . . . . . . . . . . . . . . . . . . . . . . . . . . . . . . . . . . . . . . . . . . . . . . . . . . . . . . . . . . . . . . . . . . . . . . . . . . . . . . . . . . . . . . . . . . . . . . . . . . . . . . . . . . . . . . .    
68	. . . . . . . . . . A . . . . . . . . . . . . . . . . . . . . . . . . . . . . . . . . . . . . . . . . . . . . . . . . . . . . . . . . . . . . . . . . . . . . . . . . . . . . . . . . . . . . . . . . . . . . . . . . . . . . . . . . . . . . . . . . . . . . . . . . . . . . . . . . . . . . . . . . . . . . . . . . . . . . . . . . . . . . . . . . . . . . . . . . . . . . . .    
69	. . . . . . . . . . T . . . . . . . . . . . . . . . . . . . . . . . . . . . . . . . . . . . . . . . . . . . . . . . . . . . . . . . . . . . . . . . . . . . . . . . . . . . . . . . . . . . . . . . . . . . . . . . . . . . . . . . . . . . . . . . . . . . . . . . . . . . . . . . . . . . . . . . . . . . . . . . . . . . . . . . . . . . . . . . . . . . . . . . . . . . . . .    
70	. . . . . . . . . . G . . . . . . . . . . . . . . . . . . . . . . . . . . . . . . . . . . . . . . . . . . . . . . . . . . . . . . . . . . . . . . . . . . . . . . . . . . . . . . . . . . . . . . . . . . . . . . . . . . . . . . . . . . . . . . . . . . . . . . . . . . . . . . . . . . . . . . . . . . . . . . . . . . . . . . . . . . . . . . . . . . . . . . . . . . . . . .    
71	. . . . . . . . . . A . . . . . . . . . . . . . . . . . . . . . . . . . . . . . . . . . . . . . . . . . . . . . . . . . . . . . . . . . . . . . . . . . . . . . . . . . . . . . . . . . . . . . . . . . . . . . . . . . . . . . . . . . . . . . . . . . . . . . . . . . . . . . . . . . . . . . . . . . . . . . . . . . . . . . . . . . . . . . . . . . . . . . . . . . . . . . .    
72	. . . . . . . . . . A . . . . . . . . . . . . . . . . . . . . . . . . . . . . . . . . . . . . . . . . . . . . . . . . . . . . . . . . . . . . . . . . . . . . . . . . . . . . . . . . . . . . . . . . . . . . . . . . . . . . . . . . . . . . . . . . . . . . . . . . . . . . . . . . . . . . . . . . . . . . . . . . . . . . . . . . . . . . . . . . . . . . . . . . . . . . . .    
73	. . . . . . . . . . A . . . . . . . . . . . . . . . . . . . . . . . . . . . . . . . . . . . . . . . . . . . . . . . . . . . . . . . . . . . . . . . . . . . . . . . . . . . . . . . . . . . . . . . . . . . . . . . . . . . . . . . . . . . . . . . . . . . . . . . . . . . . . . . . . . . . . . . . . . . . . . . . . . . . . . . . . . . . . . . . . . . . . . . . . . . . . .    
74	. . . . . . . . . . C . . . . . . . . . . . . . . . . . . . . . . . . . . . . . . . . . . . . . . . . . . . . . . . . . . . . . . . . . . . . . . . . . . . . . . . . . . . . . . . . . . . . . . . . . . . . . . . . . . . . . . . . . . . . . . . . . . . . . . . . . . . . . . . . . . . . . . . . . . . . . . . . . . . . . . . . . . . . . . . . . . . . . . . . . . . . . .    
75	. . . . . . . . . . C . . . . . . . . . . . . . . . . . . . . . . . . . . . . . . . . . . . . . . . . . . . . . . . . . . . . . . . . . . . . . . . . . . . . . . . . . . . . . . . . . . . . . . . . . . . . . . . . . . . . . . . . . . . . . . . . . . . . . . . . . . . . . . . . . . . . . . . . . . . . . . . . . . . . . . . . . . . . . . . . . . . . . . . . . . . . . .    
76	. . . . . . . . . . A . . . . . . . . . . . . . . . . . . . . . . . . . . . . . . . . . . . . . . . . . . . . . . . . . . . . . . . . . . . . . . . . . . . . . . . . . . . . . . . . . . . . . . . . . . . . . . . . . . . . . . . . . . . . . . . . . . . . . . . . . . . . . . . . . . . . . . . . . . . . . . . . . . . . . . . . . . . . . . . . . . . . . . . . . . . . . .    
77	. . . . . . . . . . A . . . . . . . . . . . . . . . . . . . . . . . . . . . . . . . . . . . . . . . . . . . . . . . . . . . . . . . . . . . . . . . . . . . . . . . . . . . . . . . . . . . . . . . . . . . . . . . . . . . . . . . . . . . . . . . . . . . . . . . . . . . . . . . . . . . . . . . . . . . . . . . . . . . . . . . . . . . . . . . . . . . . . . . . . . . . . .    
78	. . . . . . . . . . A . . . . . . . . . . . . . . . . . . . . . . . . . . . . . . . . . . . . . . . . . . . . . . . . . . . . . . . . . . . . . . . . . . . . . . . . . . . . . . . . . . . . . . . . . . . . . . . . . . . . . . . . . . . . . . . . . . . . . . . . . . . . . . . . . . . . . . . . . . . . . . . . . . . . . . . . . . . . . . . . . . . . . . . . . . . . . .    
79	. . . . . . . . . . C . . . . . . . . . . . . . . . . . . . . . . . . . . . . . . . . . . . . . . . . . . . . . . . . . . . . . . . . . . . . . . . . . . . . . . . . . . . . . . . . . . . . . . . . . . . . . . . . . . . . . . . . . . . . . . . . . . . . . . . . . . . . . . . . . . . . . . . . . . . . . . . . . . . . . . . . . . . . . . . . . . . . . . . . . . . . . .    
80	. . . . . . . . . . T . . . . . . . . . . . . . . . . . . . . . . . . . . . . . . . . . . . . . . . . . . . . . . . . . . . . . . . . . . . . . . . . . . . . . . . . . . . . . . . . . . . . . . . . . . . . . . . . . . . . . . . . . . . . . . . . . . . . . . . . . . . . . . . . . . . . . . . . . . . . . . . . . . . . . . . . . . . . . . . . . . . . . . . . . . . . . .    
81	. . . . . . . . . . G . . . . . . . . . . . . . . . . . . . . . . . . . . . . . . . . . . . . . . . . . . . . . . . . . . . . . . . . . . . . . . . . . . . . . . . . . . . . . . . . . . . . . . . . . . . . . . . . . . . . . . . . . . . . . . . . . . . . . . . . . . . . . . . . . . . . . . . . . . . . . . . . . . . . . . . . . . . . . . . . . . . . . . . . . . . . . .    
82	. . . . . . . . . . T . . . . . . . . . . . . . . . . . . . . . . . . . . T . . . . . . . . . . . . . . . . . . . . . . . . . . . . . . . . . . . . . . . . . . . . . . . . . . . . . . . . . . . . . . . . . . . . . . . . . . . . . . . . . . . . . . . . . . . . . . . . . . . . . . . . . . . . . . . . . . . . . . . . . . . . . . . . . . . . . . . . . . . . . . . . . . .    
83	. . . . . . . . . . C . . . . . . . . . . . . . . . . . . . . . . . . . . C . . . . . . . . . . . . . . . . . . . . . . . . . . . . . . . . . . . . . . . . . . . . . . . . . . . . . . . . . . . . . . . . . . . . . . . . . . . . . . . . . . . . . . . . . . . . . . . . . . . . . . . . . . . . . . . . . . . . . . . . . . . . . . . . . . . . . . . . . . . . . . . . . . .    
84	. . . . . . . . . . C . . . . . . . . . . . . . . . . . . . . . . . . . . C . . . . . . . . . . . . . . . . . . . . . . . . . . . . . . . . . . . . . . . . . . . . . . . . . . . . . . . . . . . . . . . . . . . . . . . . . . . . . . . . . . . . . . . . . . . . . . . . . . . . . . . . . . . . . . . . . . . . . . . . . . . . . . . . . . . . . . . . . . . . . . . . . . .    
85	. . . . . . . . . . A . . . . . . . . . . . . . . . . . . . . . . . . . . A . . . . . . . . . . . . . . . . . . . . . . . . . . . . . . . . . . . . . . . . . . . . . . . . . . . . . . . . . . . . . . . . . . . . . . . . . . . . . . . . . . . . . . . . . . . . . . . . . . . . . . . . . . . . . . . . . . . . . . . . . . . . . . . . . . . . . . . . . . . . . . . . . . .    
86	. . . . . . . . . . A . . . . . . . . . . . . . . . . . . . . . . . . . . A . . . . . . . . . . . . . . . . . . . . . . . . . . . . . . . . . . . . . . . . . . . . . . . . . . . . . . . . . . . . . . . . . . . . . . . . . . . . . . . . . . . . . . . . . . . . . . . . . . . . . . . . . . . . . . . . . . . . . . . . . . . . . . . . . . . . . . . . . . . . . . . . . . .    
87	. . . . . . . . . . C . . . . . . . . . . . . . . . . . . . . . . . . . . C . . . . . . . . . . . . . . . . . . . . . . . . . . . . . . . . . . . . . . . . . . . . . . . . . . . . . . . . . . . . . . . . . . . . . . . . . . . . . . . . . . . . . . . . . . . . . . . . . . . . . . . . . . . . . . . . . . . . . . . . . . . . . . . . . . . . . . . . . . . . . . . . . . .    
88	. . . . . . . . . . C . . . . . . . . . . . . . . . . . . . . . . . . . . C . . . . . . . . . . . . . . . . . . . . . . . . . . . . . . . . . . . . . . . . . . . . . . . . . . . . . . . . . . . . . . . . . . . . . . . . . . . . . . . . . . . . . . . . . . . . . . . . . . . . . . . . . . . . . . . . . . . . . . . . . . . . . . . . . . . . . . . . . . . . . . . . . . .    
89	. . . . . . . . . . T . . . . . . . . . . . . . . . . . . . . . . . . . . T . . . . . . . . . . . . . . . . . . . . . . . . . . . . . . . . . . . . . . . . . . . . . . . . . . . . . . . . . . . . . . . . . . . . . . . . . . . . . . . . . . . . . . . . . . . . . . . . . . . . . . . . . . . . . . . . . . . . . . . . . . . . . . . . . . . . . . . . . . . . . . . . . . .    
90	. . . . . . . . . . G . . . . . . . . . . . . . . . . . . . . . . . . . . G . . . . . . . . . . . . . . . . . . . . . . . . . . . . . . . . . . . . . . . . . . . . . . . . . . . . . . . . . . . . . . . . . . . . . . . . . . . . . . . . . . . . . . . . . . . . . . . . . . . . . . . . . . . . . . . . . . . . . . . . . . . . . . . . . . . . . . . . . . . . . . . . . . .    
91	. . . . . . . . . . A . . . . . . . . . . . . . . . . . . . . . . . . . . A . . . . . . . . . . . . . . . . . . . . . . . . . . . . . . . . . . . . . . . . . . . . . . . . . . . . . . . . . . . . . . . . . . . . . . . . . . . . . . . . . . . . . . . . . . . . . . . . . . . . . . . . . . . . . . . . . . . . . . . . . . . . . . . . . . . . . . . . . . . . . . . . . . .    
92	. . . . . . . . . . G . . . . . . . . . . . . . . . . . . . . . . . . . . G . . . . . . . . . . . . . . . . . . . . . . . . . . . . . . . . . . . . . . . . . . . . . . . . . . . . . . . . . . . . . . . . . . . . . . . . . . . . . . . . . . . . . . . . . . . . . . . . . . . . . . . . . . . . . . . . . . . . . . . . . . . . . . . . . . . . . . . . . . . . . . . . . . .    
93	. . . . . . . . . . T . . . . . . . . . . . . . . . . . . . . . . . . . . T . . . . . . . . . . . . . . . . . . . . . . . . . . . . . . . . . . . . . . . . . . . . . . . . . . . . . . . . . . . . . . . . . . . . . . . . . . . . . . . . . . . . . . . . . . . . . . . . . . . . . . . . . . . . . . . . . . . . . . . . . . . . . . . . . . . . . . . . . . . . . . . . . . .    
94	. . . . . . . . . . T . . . . . . . . . . . . . . . . . . . . . . . . . . T . . . . . . . . . . . . . . . . . . . . . . . . . . . . . . . . . . . . . . . . . . . . . . . . . . . . . . . . . . . . . . . . . . . . . . . . . . . . . . . . . . . . . . . . . . . . . . . . . . . . . . . . . . . . . . . . . . . . . . . . . . . . . . . . . . . . . . . . . . . . . . . . . . .    
95	. . . . . . . . . . T . . . . . . . . . . . . . . . . . . . . . . . . . . T . . . . . . . . . . . . . . . . . . . . . . . . . . . . . . . . . . . . . . . . . . . . . . . . . . . . . . . . . . . . . . . . . . . . . . . . . . . . . . . . . . . . . . . . . . . . . . . . . . . . . . . . . . . . . . . . . . . . . . . . . . . . . . . . . . . . . . . . . . . . . . . . . . .    
96	. . . . . . . . . . A . . . . . . . . . . . . A . . . . . . . . . . . . . A . . . . . . . . . . . . . . . . . . . . . . . . . . . . . . . . . . . . . . . . . . . . . . . . . . . . . . . . . . . . . . . . . . . . . . . . . . . . . . . . . . . . . . . . . . . . . . . . . . . . . . . . . . . . . . . . . . . . . . . . . . . . . . . . . . . . . . . . . . . . . . . . . . .    
97	. . . . . . . . . . T . . . . . . . . . . . . T . . . . . . . . . . . . . T . . . . . . . . . . . . . . . . . . . . . . . . . . . . . . . . . . . . . . . . . . . . . . . . . . . . . . . . . . . . . . . . . . . . . . . . . . . . . . . . . . . . . . . . . . . . . . . . . . . . . . . . . . . . . . . . . . . . . . . . . . . . . . . . . . . . . . . . . . . . . . . . . . .    
98	. . . . . . . . . . T . . . . . . . . . . . . T . . . . . . . . . . . . . T . . . . . . . . . . . . . . . . . . . . . . . . . . . . . . . . . . . . . . . . . . . . . . . . . . . . . . . . . . . . . . . . . . . . . . . . . . . . . . . . . . . . . . . . . . . . . . . . . . . . . . . . . . . . . . . . . . . . . . . . . . . . . . . . . . . . . . . . . . . . . . . . . . .    
99	. . . . . . . . . . T . . . . . . . . . . . . T . . . . . . . . . . . . . T . . . . . . . . . . . . . . . . . . . . . . . . . . . . . . . . . . . . . . . . . . . . . . . . . . . . . . . . . . . . . . . . . . . . . . . . . . . . . . . . . . . . . . . . . . . . . . . . . . . . . . . . . . . . . . . . . . . . . . . . . . . . . . . . . . . . . . . . . . . . . . . . . . .    
100	. . . . . . . . . . T . . . . . . . . . . . . T . . . . . . . . . . . . . T . . . . . . . . . . . . . . . . . . . . . . . . . . . . . . . . . . . . . . . . . . . . . . . . . . . . . . . . . . . . . . . . . . . . . . . . . . . . . . . . . . . . . . . . . . . . . . . . . . . . . . . . . . . . . . . . . . . . . . . . . . . . . . . . . . . . . . . . . . . . . . . . . . .    
101	. . . . . . . . . . A . . . . . . . . . . . . A . . . . . . . . . . . . . A . . . . . . . . . . . . . . . . . . . . . . . . . . . . . . . . . . . . . . . . . . . . . . . . . . . . . . . . . . . . . . . . . . . . . . . . . . . . . . . . . . . . . . . . . . . . . . . . . . . . . . . . . . . . . . . . . . . . . . . . . . . . . . . . . . . . . . . . . . . . . . . . . . .    
102	. . . . . . . . . . T . . . . . . . . . . . . T . . . . . . . . . . . . . T . . . . . . . . . . . . . . . . . . . . . . . . . . . . . . . . . . . . . . . . . . . . . . . . . . . . . . . . . . . . . . . . . . . . . . . . . . . . . . . . . . . . . . . . . . . . . . . . . . . . . . . . . . . . . . . . . . . . . . . . . . . . . . . . . . . . . . . . . . . . . . . . . . .    
103	. . . . . . . . . . G . . . . . . . . . . . . G . . . . . . . . . . . . . G . . . . . . . . . . . . . . . . . . . . . . . . . . . . . . . . . . . . . . . . . . . . . . . . . . . . . . . . . . . . . . . . . . . . . . . . . . . . . . . . . . . . . . . . . . . . . . . . . . . . . . . . . . . . . . . . . . . . . . . . . . . . . . . . . . . . . . . . . . . . . . . . . . .    
104	. . . . . . . . . . T . . . . . . . . . . . . T . . . . . . . . . . . . . T . . . . . . . . . . . . . . . . . . . . . . . . . . . . . . . . . . . . . . . . . . . . . . . . . . . . . . . . . . . . . . . . . . . . . . . . . . . . . . . . . . . . . . . . . . . . . . . . . . . . . . . . . . . . . . . . . . . . . . . . . . . . . . . . . . . . . . . . . . . . . . . . . . .    
105	. . . . . . . . . . G . . . . . . . . . . . . G . . . . . . . . . . . . . G . . . . . . . . . . . . . . . . . . . . . . . . . . . . . . . . . . . . . . . . . . . . . . . . . . . . . . . . . . . . . . . . . . . . . . . . . . . . . . . . . . . . . . . . . . . . . . . . . . . . . . . . . . . . . . . . . . . . . . . . . . . . . . . . . . . . . . . . . . . . . . . . . . .    
106	. . . . . . . . . . T . . . . . . . . . . . . T . . . . . . . . . . . . . T . . . . . . . . . . . . . . . . . . . . . . . . . . . . . . . . . . . . . . . . . . . . . . . . . . . . . . . . . . . . . . . . . . . . . . . . . . . . . . . . . . . . . . . . . . . . . . . . . . . . . . . . . . . . . . . . . . . . . . . . . . . . . . . . . . . . . . . . . . . . . . . . . . .    
107	. . . . . . . . . . C . . . . . . . . . . . . C . . . . . . . . . . . . . C . . . . . . . . . . . . . . . . . . . . . . . . . . . . . . . . . . . . . . . . . . . . . . . . . . . . . . . . . . . . . . . . . . . . . . . . . . . . . . . . . . . . . . . . . . . . . . . . . . . . . . . . . . . . . . . . . . . . . . . . . . . . . . . . . . . . . . . . . . . . . . . . . . .    
108	. . . . . . . . . . A . . . . . . . . . . . . A . . . . . . . . . . . . . A . . . . . . . . . . . . . . . . . . . . . . . . . . . . . . . . . . . . . . . . . . . . . . . . . . . . . . . . . . . . . . . . . . . . . . . . . . . . . . . . . . . . . . . . . . . . . . . . . . . . . . . . . . . . . . . . . . . . . . . . . . . . . . . . . . . . . . . . . . . . . . . . . . .    
109	. . . . . . . . . . G . . . . . . . . . . . . G . . . . . . . . . . . . . G . . . . . . . . . . . . . . . . . . . . . . . . . . . . . . . . . . . . . . . . . . . . . . . . . . . . . . . . . . . . . . . . . . . . . . . . . . . . . . . . . . . . . . . . . . . . . . . . . . . . . . . . . . . . . . . . . . . . . . . . . . . . . . . . . . . . . . . . . . . . . . . . . . .    
110	. . . . . . . . . . G . . . . . . . . . . . . G . . . . . . . . . . . . . G . . . . . . . . . . . . . . . . . . . . . . . . . . . . . . . . . . . . . . . . . . . . . . . . . . . . . . . . . . . . . . . . . . . . . . . . . . . . . . . . . . . . . . . . . . . . . . . . . . . . . . . . . . . . . . . . . . . . . . . . . . . . . . . . . . . . . . . . . . . . . . . . . . .    
111	. . . . . . . . . . G . . . . . . . . . . . . G . . . . . . . . . . . . . G . . . . . . . . . . . . . . . . . . . . . . . . . . . . . . . . . . . . . . . . . . . . . . . . . . . . . . . . . . . . . . . . . . . . . . . . . . . . . . . . . . . . . . . . . . . . . . . . . . . . . . . . . . . . . . . . . . . . . . . . . . . . . . . . . . . . . . . . . . . . . . . . . . .    
112	. . . . . A . . . . A . . . . . . . . . . . . A . . . . . . . . . . . . . A . . . . . . . . . . . . . . . . . . . . . . . . . . . . . . . . . . . . . . . . . . . . . . . . . . . . . . . . . . . . . . . . . . . . . . . . . . . . . . . . . . . . . . . . . . . . . . . . . . . . . . . . . . . . . . . . . . . . . . . . . . . . . . . . . . . . . . . . . . . . . . . . . . .    
113	. . . . . C . . . . C . . . . . . . . . . . . C . . . . . . . . . . . . . C . . . . . . . . . . . . . . . . . . . . . . . . . . . . . . . . . . . . . . . . . . . . . . . . . . . . . . . . . . . . . . . . . . . . . . . . . . . . . . . . . . . . . . . . . . . . . . . . . . . . . . . . . . . . . . . . . . . . . . . . . . . . . . . . . . . . . . . . . . . . . . . . . . .    
114	. . . . . T . . . . T . . . . . . . . . . . . T . . . . . . . . . . . . . T . . . . . . . . . . . . . . . . . . . . . . . . . . . . . . . . . . . . . . . . . . . . . . . . . . . . . . . . . . . . . . . . . . . . . . . . . . . . . . . . . . . . . . . . . . . . . . . . . . . . . . . . . . . . . . . . . . . . . . . . . . . . . . . . . . . . . . . . . . . . . . . . . . .    
115	. . . . . T . . . . T . . . . . . . . . . . . T . . . . . . . . . . . . . T . . . . . . . . . . . . . . . . . . . . . . . . . . . . . . . . . . . . . . . . . . . . . . . . . . . . . . . . . . . . . . . . . . . . . . . . . . . . . . . . . . . . . . . . . . . . . . . . . . . . . . . . . . . . . . . . . . . . . . . . . . . . . . . . . . . . . . . . . . . . . . . . . . .    
116	. . . . . T . . . . T . . . . . . . . . . . . T . . . . . . . . . . . . . T . . . . . . . . . . . . . . . . . . . . . . . . . . . . . . . . . . . . . . . . . . . . . . . . . . . . . . . . . . . . . . . . . . . . . . . . . . . . . . . . . . . . . . . . . . . . . . . . . . . . . . . . . . . . . . . . . . . . . . . . . . . . . . . . . . . . . . . . . . . . . . . . . . .    
117	. . . . . C . . . . C . . . . . . . . . . . . C . . . . . . . . . . . . . C . . . . . . . . . . . . . . . . . . . . . . . . . . . . . . . . . . . . . . . . . . . . . . . . . . . . . . . . . . . . . . . . . . . . . . . . . . . . . . . . . . . . . . . . . . . . . . . . . . . . . . . . . . . . . . . . . . . . . . . . . . . . . . . . . . . . . . . . . . . . . . . . . . .    
118	. . . . . C . . . . C . . . . . . . . . . . . C . . . . . . . . . . . . . C . . . . . . . . . . . . . . . . . . . . . . . . . . . . . . . . . . . . . . . . . . . . . . . . . . . . . . . . . . . . . . . . . . . . . . . . . . . . . . . . . . . . . . . . . . . . . . . . . . . . . . . . . . . . . . . . . . . . . . . . . . . . . . . . . . . . . . . . . . . . . . . . . . .    
119	. . . . . T . . . . T . . . . . . . . . . . . T . . . . . . . . . . . . . T . . . . . . . . . . . . . . . . . . . . . . . . . . . . . . . . . . . . . . . . . . . . . . . . . . . . . . . . . . . . . . . . . . . . . . . . . . . . . . . . . . . . . . . . . . . . . . . . . . . . . . . . . . . . . . . . . . . . . . . . . . . . . . . . . . . . . . . . . . . . . . . . . . .    
120	. . . . . T . . . . T . . . . . . . . . . . . T . . . . . . . . . . . . . T . . . . . . . . . . . . . . . . . . . . . . . . . . . . . . . . . . . . . . . . . . . . . . . . . . . . . . . . . . . . . . . . . . . . . . . . . . . . . . . . . . . . . . . . . . . . . . . . . . . . . . . . . . . . . . . . . . . . . . . . . . . . . . . . . . . . . . . . . . . . . . . . . . .    
121	. . . . . G . . . . G . . . . . . . . . . . . G . . . . . . . . . . . . . G . . . . . . . . . . . . . . . . . . . . . . . . . . . . . . . . . . . . . . . . . . . . . . . . . . . . . . . . . . . . . . . . . . . . . . . . . . . . . . . . . . . . . . . . . . . . . . . . . . . . . . . . . . . . . . . . . . . . . . . . . . . . . . . . . . . . . . . . . . . . . . . . . . .    
122	. . . . . A . . . . A . . . . . . . . . . . . A . . . . . . . . . . . . . A . . . . . . . . . . . . . . . . . . . . . . . . . . . . . . . . . . . . . . . . . . . . . . . . . . . . . . . . . . . . . . . . . . . . . . . . . . . . . . . . . . . . . . . . . . . . . . . . . . . . . . . . . . . . . . . . . . . . . . . . . . . . . . . . . . . . . . . . . . . . . . . . . . .    
123	. . . . . A . . . . A . . . . . . . . . . . . A . . . . . . . . . . . . . A . . . . . . . . . . . . . . . . . . . . . . . . . . . . . . . . . . . . . . . . . . . . . . . . . . . . . . . . . . . . . . . . . . . . . . . . . . . . . . . . . . . . . . . . . . . . . . . . . . . . . . . . . . . . . . . . . . . . . . . . . . . . . . . . . . . . . . . . . . . . . . . . . . .    
124	. . . . . T . . . . T . . . . . . . . . . . . T . . . . . . . . . . . . . T . . . . . . . . . . . . . . . . . . . . . . . . . . . . . . . . . . . . . . . . . . . . . . . . . . . . . . . . . . . . . . . . . . . . . . . . . . . . . . . . . . . . . . . . . . . . . . . . . . . . . . . . . . . . . . . . . . . . . . . . . . . . . . . . . . . . . . . . . . . . . . . . . . .    
125	. . . . . T . . . . T . . . . . . . . . . . . T . . . . . . . . . . . . . T . . . . . . . . . . . . . . . . . . . . . . . . . . . . . . . . . . . . . . . . . . . . . . . . . . . . . . . . . . . . . . . . . . . . . . . . . . . . . . . . . . . . . . . . . . . . . . . . . . . . . . . . . . . . . . . . . . . . . . . . . . . . . . . . . . . . . . . . . . . . . . . . . . .    
126	. . . . . T . . . . T . . . . . . . . . . . . T . . . . . . . . . . . . . T . . . . . . . . . . . . . . . . . . . . . . . . . . . . . . . . . . . . . . . . . . . . . . . . . . . . . . . . . . . . . . . . . . . . . . . . . . . . . . . . . . . . . . . . . . . . . . . . . . . . . . . . . . . . . . . . . . . . . . . . . . . . . . . . . . . . . . . . . . . . . . . . . . .    
127	. . . . . G . . . . G . . . . . . . . . . . G G . . . . . . . . . . . . . G . . . . . . . . . . . . . . . . . . . . . . . . . . . . . . . . . . . . . . . . . . . . . . . . . . . . . . . . . . . . . . . . . . . . . . . . . . . . . . . . . . . . . . . . . . . . . . . . . . . . . . . . . . . . . . . . . . . . . . . . . . . . . . . . . . . . . . . . . . . . . . . . . . .    
128	. . . . . A . . . . A . . . . . . . . . . . A A . . . . . . . . . . . . . A . . . . . . . . . . . . . . . . . . . . . . . . . . . . . . . . . . . . . . . . . . . . . . . . . . . . . . . . . . . . . . . . . . . . . . . . . . . . . . . . . . . . . . . . . . . . . . . . . . . . . . . . . . . . . . . . . . . . . . . . . . . . . . . . . . . . . . . . . . . . . . . . . . .    
129	. . . . . C . . . . C . . . . . . . . . . . C C . . . . . . . . . . . . . C . . . . . . . . . . . . . . . . . . . . . . . . . . . . . . . . . . . . . . . . . . . . . . . . . . . . . . . . . . . . . . . . . . . . . . . . . . . . . . . . . . . . . . . . . . . . . . . . . . . . . . . . . . . . . . . . . . . . . . . . . . . . . . . . . . . . . . . . . . . . . . . . . . .    
130	. . . . . C . . . . C . . . . . . . . . . . C C . . . . . . . . . . . . . C . . . . . . . . . . . . . . . . . . . . . . . . . . . . . . . . . . . . . . . . . . . . . . . . . . . . . . . . . . . . . . . . . . . . . . . . . . . . . . . . . . . . . . . . . . . . . . . . . . . . . . . . . . . . . . . . . . . . . . . . . . . . . . . . . . . . . . . . . . . . . . . . . . .    
131	. . . . . A . . . . A . . . . . . . . . . . A A A A . . . . . . . . . . . A A A . . . . . . . . . . . . . . . . . . . . . . . . . . . . . . . . . . . . . . . . . . . . . . . . . . . . . . . . . . . . . . . . . . . . . . . . . . . . . . . . . . . . . . . . . . . . . . . . . . . . . . . . . . . . . . . . . . . . . . . . . . . . . . . . . . . . . . . . . . . . . . . . .    
132	. . . . . A . . . . A . . . . . . . . . . . A A A A . . . . . . . . . . . A A A . . . . . . . . . . . . . . . . . . . . . . . . . . . . . . . . . . . . . . . . . . . . . . . . . . . . . . . . . . . . . . . . . . . . . . . . . . . . . . . . . . . . . . . . . . . . . . . . . . . . . . . . . . . . . . . . . . . . . . . . . . . . . . . . . . . . . . . . . . . . . . . . .    
133	. . . . . T . . . . T . . . . . . . . . . . T T T T . . . . . . . . . . . T T T . . . . . . . . . . . . . . . . . . . . . . . . . . . . . . . . . . . . . . . . . . . . . . . . . . . . . . . . . . . . . . . . . . . . . . . . . . . . . . . . . . . . . . . . . . . . . . . . . . . . . . . . . . . . . . . . . . . . . . . . . . . . . . . . . . . . . . . . . . . . . . . . .    
134	. . . . . C . . . . C . . . . . . . . . . . C C C C . . . . . . . . . . . C C C . . . . . . . . . . . . . . . . . . . . . . . . . . . . . . . . . . . . . . . . . . . . . . . . . . . . . . . . . . . . . . . . . . . . . . . . . . . . . . . . . . . . . . . . . . . . . . . . . . . . . . . . . . . . . . . . . . . . . . . . . . . . . . . . . . . . . . . . . . . . . . . . .    
135	. . . . . T . . . . T . . . . . . . . . . . T T T T . . . . . . . . . . . T T T . . . . . . . . . . . . . . . . . . . . . . . . . . . . . . . . . . . . . . . . . . . . . . . . . . . . . . . . . . . . . . . . . . . . . . . . . . . . . . . . . . . . . . . . . . . . . . . . . . . . . . . . . . . . . . . . . . . . . . . . . . . . . . . . . . . . . . . . . . . . . . . . .    
136	. . . . . C . . . . C . . . . . . . . . . . C C C C . . . . . . . . . . . C C C . . . . . . . . . . . . . . . . . . . . . . . . . . . . . . . . . . . . . . . . . . . . . . . . . . . . . . . . . . . . . . . . . . . . . . . . . . . . . . . . . . . . . . . . . . . . . . . . . . . . . . . . . . . . . . . . . . . . . . . . . . . . . . . . . . . . . . . . . . . . . . . . .    
137	. . . . . A . . . . A . . . . . . . . . . . A A A A . . . . . . . . . . . A A A A . . . . . . . . . . . . . . . . . . . . . . . . . . . . . . . . . . . . . . . . . . . . . . . . . . . . . . . . . . . . . . . . . . . . . . . . . . . . . . . . . . . . . . . . . . . . . . . . . . . . . . . . . . . . . . . . . . . . . . . . . . . . . . . . . . . . . . . . . . . . . . . .    
138	. . . . . A . . . . A . . . . . . . . . . . A A A A . . . . . . . . . . . A A A A . . . . . . . . . . . . . . . . . . . . . . . . . . . . . . . . . . . . . . . . . . . . . . . . . . . . . . . . . . . . . . . . . . . . . . . . . . . . . . . . . . . . . . . . . . . . . . . . . . . . . . . . . . . . . . . . . . . . . . . . . . . . . . . . . . . . . . . . . . . . . . . .    
139	. . . . . T . . . . T . . . . . . . . . . . T T T T . . . . . . . . . . . T T T T . . . . . . . . . . . . . . . . . . . . . . . . . . . . . . . . . . . . . . . . . . . . . . . . . . . . . . . . . . . . . . . . . . . . . . . . . . . . . . . . . . . . . . . . . . . . . . . . . . . . . . . . . . . . . . . . . . . . . . . . . . . . . . . . . . . . . . . . . . . . . . . .    
140	. . . . . T . . . . T . . . . . . . . . . . T T T T . . . . . . . . . . . T T T T . . . . . . . . . . . . . . . . . . . . . . . . . . . . . . . . . . . . . . . . . . . . . . . . . . . . . . . . . . . . . . . . . . . . . . . . . . . . . . . . . . . . . . . . . . . . . . . . . . . . . . . . . . . . . . . . . . . . . . . . . . . . . . . . . . . . . . . . . . . . . . . .    
141	. . . . . T . . . . T . . . . . . . . . . . T T T T . . . . . . . . . . . T T T T . . . . . . . . . . . . . . . . . . . . . . . . . . . . . . . . . . . . . . . . . . . . . . . . . . . . . . . . . . . . . . . . . . . . . . . . . . . . . . . . . . . . . . . . . . . . . . . . . . . . . . . . . . . . . . . . . . . . . . . . . . . . . . . . . . . . . . . . . . . . . . . .    
142	. . . . . C . . . . C . . . . . . . . . . . C C C C . . . . . . . . . . . C C C C . . . . . . . . . . . . . . . . . . . . . . . . . . . . . . . . . . . . . . . . . . . . . . . . . . . . . . . . . . . . . . . . . . . . . . . . . . . . . . . . . . . . . . . . . . . . . . . . . . . . . . . . . . . . . . . . . . . . . . . . . . . . . . . . . . . . . . . . . . . . . . . .    
143	. . . . . G . . . . G . . . . . . . . . . . G G G G . . . . . . . . . . . G G G G . . . . . . . . . . . . . . . . . . . . . . . . . . . . . . . . . . . . . . . . . . . . . . . . . . . . . . . . . . . . . . . . . . . . . . . . . . . . . . . . . . . . . . . . . . . . . . . . . . . . . . . . . . . . . . . . . . . . . . . . . . . . . . . . . . . . . . . . . . . . . . . .    
144	. . . . . C C . . . C . . . . . . . . . . . C C C C . . . . . . . . . . . C C C C . . . . . . . . . . . . . . . . . . . . . . . . . . . . . . . . . . . . . . . . . . . . . . . . . . . . . . . . . . . . . . . . . . . . . . . . . . . . . . . . . . . . . . . . . . . . . . . . . . . . . . . . . . . . . . . . . . . . . . . . . . . . . . . . . . . . . . . . . . . . . . . .    
145	. . . . . T T . . . T . . . . . . . . . . . T T T T . . . . . . . . . . . T T T T . . . . . . . . . . . . . . . . . . . . . . . . . . . . . . . . . . . . . . . . . . . . . . . . . . . . . . . . . . . . . . . . . . . . . . . . . . . . . . . . . . . . . . . . . . . . . . . . . . . . . . . . . . . . . . . . . . . . . . . . . . . . . . . . . . . . . . . . . . . . . . . .    
146	. . . . . T T . . . T . . . . . . . . . . . T T T T . . . . . . . . . . . T T T T . . . . . . . . . . . . . . . . . . . . . . . . . . . . . . . . . . . . . . . . . . . . . . . . . . . . . . . . . . . . . . . . . . . . . . . . . . . . . . . . . . . . . . . . . . . . . . . . . . . . . . . . . . . . . . . . . . . . . . . . . . . . . . . . . . . . . . . . . . . . . . . .    
147	. . . . . C C . . . C . . . . . . . . . . . C C C C . . . . . . . . . . . C C C C . . . . . . . . . . . . . . . . . . . . . . . . . . . . . . . . . . . . . . . . . . . . . . . . . . . . . . . . . . . . . . . . . . . . . . . . . . . . . . . . . . . . . . . . . . . . . . . . . . . . . . . . . . . . . . . . . . . . . . . . . . . . . . . . . . . . . . . . . . . . . . . .    
148	. . . . . T T . . . T . . . . . . . . . . . T T T T . . . . . . . . . . . T T T T . . . . . . . . . . . . . . . . . . . . . . . . . . . . . . . . . . . . . . . . . . . . . . . . . . . . . . . . . . . . . . . . . . . . . . . . . . . . . . . . . . . . . . . . . . . . . . . . . . . . . . . . . . . . . . . . . . . . . . . . . . . . . . . . . . . . . . . . . . . . . . . .    
149	. . . . . C C . . . C . . . . . . . . . . . C C C C . . . . . . . . . . . C C C C . . . . . . . . . . . . . . . . . . . . . . . . . . . . . . . . . . . . . . . . . . . . . . . . . . . . . . . . . . . . . . . . . . . . . . . . . . . . . . . . . . . . . . . . . . . . . . . . . . . . . . . . . . . . . . . . . . . . . . . . . . . . . . . . . . . . . . . . . . . . . . . .    
150	. . . . . C C . . . C . . . . . . . . . . . C C C C . . . . . . . . . . . C C C C . . . . . . . . . . . . . . . . . . . . . . . . . . . . . . . . . . . . . . . . . . . . . . . . . . . . . . . . . . . . . . . . . . . . . . . . . . . . . . . . . . . . . . . . . . . . . . . . . . . . . . . . . . . . . . . . . . . . . . . . . . . . . . . . . . . . . . . . . . . . . . . .    
151	. . . . . T T . . . T . . . . . . . . . . . T T T T . . . . . . . . . . . T T T T . . . . . . . . . . . . . . . . . . . . . . . . . . . . . . . . . . . . . . . . . . . . . . . . . . . . . . . . . . . . . . . . . . . . . . . . . . . . . . . . . . . . . . . . . . . . . . . . . . . . . . . . . . . . . . . . . . . . . . . . . . . . . . . . . . . . . . . . . . . . . . . .    
152	. . . . . C C . . . C . . . . . . . . . . . C C C C . . . . . . . . . . . C C C C . . . . . . . . . . . . . . . . . . . . . . . . . . . . . . . . . . . . . . . . . . . . . . . . . . . . . . . . . . . . . . . . . . . . . . . . . . . . . . . . . . . . . . . . . . . . . . . . . . . . . . . . . . . . . . . . . . . . . . . . . . . . . . . . . . . . . . . . . . . . . . . .    
153	. . . . . G G . . . G . . . . . . . . . . . G G G G . . . . . . . . . . . G G G G . . . . . . . . . . . . . . . . . . . . . . . . . . . . . . . . . . . . . . . . . . . . . . . . . . . . . . . . . . . . . . . . . . . . . . . . . . . . . . . . . . . . . . . . . . . . . . . . . . . . . . . . . . . . . . . . . . . . . . . . . . . . . . . . . . . . . . . . . . . . . . . .    
154	. . . . . G G . . . G . . . . . . . . . . . G G G G . . . . . . . . . . . G G G G . . . . . . . . . . . . . . . . . . . . . . . . . . . . . . . . . . . . . . . . . . . . . . . . . . . . . . . . . . . . . . . . . . . . . . . . . . . . . . . . . . . . . . . . . . . . . . . . . . . . . . . . . . . . . . . . . . . . . . . . . . . . . . . . . . . . . . . . . . . . . . . .    
155	. . . . . A A . . . A . . . . . . . . . . . A A A A . . . . . . . . . . . A A A A . . . . . . . . . . . . . . . . . . . . . . . . . . . . . . . . . . . . . . . . . . . . . . . . . . . . . . . . . . . . . . . . . . . . . . . . . . . . . . . . . . . . . . . . . . . . . . . . . . . . . . . . . . . . . . . . . . . . . . . . . . . . . . . . . . . . . . . . . . . . . . . .    
156	. . . . . C C . . . C . . . . . . . . . . . C C C C . . . . . . . . . . . C C C C . . . . . . . . . . . . . . . . . . . . . . . . . . . . . . . . . . . . . . . . . . . . . . . . . . . . . . . . . . . . . . . . . . . . . . . . . . . . . . . . . . . . . . . . . . . . . . . . . . . . . . . . . . . . . . . . . . . . . . . . . . . . . . . . . . . . . . . . . . . . . . . .    
157	. . . . . A A . . . A . . . . . . . . . . . A A A A . . . . . . . . . . . A A A A . . . . . . . . . . . . . . . . . . . . . . . . . . . . . . . . . . . . . . . . . . . . . . . . . . . . . . . . . . . . . . . . . . . . . . . . . . . . . . . . . . . . . . . . . . . . . . . . . . . . . . . . . . . . . . . . . . . . . . . . . . . . . . . . . . . . . . . . . . . . . . . .    
158	. . . . . G G . . . G . . . . . . . . . . . G G G G . . . . . . . . . . . G G G G . . . . . . . . . . . . . . . . . . . . . . . . . . . . . . . . . . . . . . . . . . . . . . . . . . . . . . . . . . . . . . . . . . . . . . . . . . . . . . . . . . . . . . . . . . . . . . . . . . . . . . . . . . . . . . . . . . . . . . . . . . . . . . . . . . . . . . . . . . . . . . . .    
159	. . . . . G G . . . G . . . . . . . . . . . G G G G G . . . . . . . . . . G G G G . . . . . . . . . . . . . . . . . . . . . . . . . . . . . . . . . . . . . . . . . . . . . . . . . . . . . . . . . . . . . . . . . . . . . . . . . . . . . . . . . . . . . . . . . . . . . . . . . . . . . . . . . . . . . . . . . . . . . . . . . . . . . . . . . . . . . . . . . . . . . . . .    
160	. . . . . T T . . . T . . . . . . . . . . . T T T T T . . . . . . . . . . T T T T . . . . . . . . . . . . . . . . . . . . . . . . . . . . . . . . . . . . . . . . . . . . . . . . . . . . . . . . . . . . . . . . . . . . . . . . . . . . . . . . . . . . . . . . . . . . . . . . . . . . . . . . . . . . . . . . . . . . . . . . . . . . . . . . . . . . . . . . . . . . . . . .    
161	. . . . . T T . . . T . . . . . . . . . . . T T T T T . . . . . . . . . . T T T T . . . . . . . . . . . . . . . . . . . . . . . . . . . . . . . . . . . . . . . . . . . . . . . . . . . . . . . . . . . . . . . . . . . . . . . . . . . . . . . . . . . . . . . . . . . . . . . . . . . . . . . . . . . . . . . . . . . . . . . . . . . . . . . . . . . . . . . . . . . . . . . .    
162	. . . . . A A . . . A . . . . . . . . . . . A A A A A . . . . . . . . . . A A A A . . . . . . . . . . . . . . . . . . . . . . . . . . . . . . . . . . . . . . . . . . . . . . . . . . . . . . . . . . . . . . . . . . . . . . . . . . . . . . . . . . . . . . . . . . . . . . . . . . . . . . . . . . . . . . . . . . . . . . . . . . . . . . . . . . . . . . . . . . . . . . . .    
163	. . . . . T T . . . T . . . . . . . . . . . T T T T T . . . . . . . . . . T T T T . . . . . . . . . . . . . . . . . . . . . . . . . . . . . . . . . . . . . . . . . . . . . . . . . . . . . . . . . . . . . . . . . . . . . . . . . . . . . . . . . . . . . . . . . . . . . . . . . . . . . . . . . . . . . . . . . . . . . . . . . . . . . . . . . . . . . . . . . . . . . . . .    
164	. . . . . A A . . . A . . . . . . . . . . . A A A A A . . . . . . . . . . A A A A . . . . . . . . . . . . . . . . . . . . . . . . . . . . . . . . . . . . . . . . . . . . . . . . . . . . . . . . . . . . . . . . . . . . . . . . . . . . . . . . . . . . . . . . . . . . . . . . . . . . . . . . . . . . . . . . . . . . . . . . . . . . . . . . . . . . . . . . . . . . . . . .    
165	. . . . . A A A . . A . . . . . . . . . . . A A A A A . . . . . . . . . . A A A A . . . . . . . . . . . . . . . . . . . . . . . . . . . . . . . . . . . . . . . . . . . . . . . . . . . . . . . . . . . . . . . . . . . . . . . . . . . . . . . . . . . . . . . . . . . . . . . . . . . . . . . . . . . . . . . . . . . . . . . . . . . . . . . . . . . . . . . . . . . . . . . .    
166	. . . . . A A A . . A . . . . . . . . . . . A A A A A . . . . . . . . . . A A A A . . . . . . . . . . . . . . . . . . . . . . . . . . . . . . . . . . . . . . . . . . . . . . . . . . . . . . . . . . . . . . . . . . . . . . . . . . . . . . . . . . . . . . . . . . . . . . . . . . . . . . . . . . . . . . . . . . . . . . . . . . . . . . . . . . . . . . . . . . . . . . . .    
167	. . . . . T T T . . T . . . . . . . . . . . T T T T T . . . . . . . . . . T T T T . . . . . . . . . . . . . . . . . . . . . . . . . . . . . . . . . . . . . . . . . . . . . . . . . . . . . . . . . . . . . . . . . . . . . . . . . . . . . . . . . . . . . . . . . . . . . . . . . . . . . . . . . . . . . . . . . . . . . . . . . . . . . . . . . . . . . . . . . . . . . . . .    
168	. . . . . T T T . . T . . . . . . . . . . . T T T T T . . . . . . . . . . T T T T . . . . . . . . . . . . . . . . . . . . . . . . . . . . . . . . . . . . . . . . . . . . . . . . . . . . . . . . . . . . . . . . . . . . . . . . . . . . . . . . . . . . . . . . . . . . . . . . . . . . . . . . . . . . . . . . . . . . . . . . . . . . . . . . . . . . . . . . . . . . . . . .    
169	. . . . . G G G . . G . . . . . . . . . . . G G G G G . . . . . . . . . . G G G G . . . . . . . . . . . . . . . . . . . . . . . . . . . . . . . . . . . . . . . . . . . . . . . . . . . . . . . . . . . . . . . . . . . . . . . . . . . . . . . . . . . . . . . . . . . . . . . . . . . . . . . . . . . . . . . . . . . . . . . . . . . . . . . . . . . . . . . . . . . . . . . .    
170	. . . . . T T T . . T . . . . . . . . . . . T T T T T . . . . . . . . . . T T T T . . . . . . . . . . . . . . . . . . . . . . . . . . . . . . . . . . . . . . . . . . . . . . . . . . . . . . . . . . . . . . . . . . . . . . . . . . . . . . . . . . . . . . . . . . . . . . . . . . . . . . . . . . . . . . . . . . . . . . . . . . . . . . . . . . . . . . . . . . . . . . . .    
171	. . . . . G G G . . G . . . . . . . . . . . G G G G G . . . . . . . . . . G G G G . . . . . . . . . . . . . . . . . . . . . . . . . . . . . . . . . . . . . . . . . . . . . . . . . . . . . . . . . . . . . . . . . . . . . . . . . . . . . . . . . . . . . . . . . . . . . . . . . . . . . . . . . . . . . . . . . . . . . . . . . . . . . . . . . . . . . . . . . . . . . . . .    
172	. . . . . T T T . . T . . . . . . . . . . . T T T T T . . . . . . . . . . T T T T . . . . . . . . . . . . . . . . . . . . . . . . . . . . . . . . . . . . . . . . . . . . . . . . . . . . . . . . . . . . . . . . . . . . . . . . . . . . . . . . . . . . . . . . . . . . . . . . . . . . . . . . . . . . . . . . . . . . . . . . . . . . . . . . . . . . . . . . . . . . . . . .    
173	. . . . . T T T . . T . . . . . . . . . . . T T T T T . . . . . . . . . . T T T T . . . . . . . . . . . . . . . . . . . . . . . . . . . . . . . . . . . . . . . . . . . . . . . . . . . . . . . . . . . . . . . . . . . . . . . . . . . . . . . . . . . . . . . . . . . . . . . . . . . . . . . . . . . . . . . . . . . . . . . . . . . . . . . . . . . . . . . . . . . . . . . .    
174	. . . . . T T T . . T . . . . . . . . . . . T T T T T . . . . . . . . . . T T T T . . . . . . . . . . . . . . . . . . . . . . . . . . . . . . . . . . . . . . . . . . . . . . . . . . . . . . . . . . . . . . . . . . . . . . . . . . . . . . . . . . . . . . . . . . . . . . . . . . . . . . . . . . . . . . . . . . . . . . . . . . . . . . . . . . . . . . . . . . . . . . . .    
175	. . . . . G G G . . G . . . . . . . . . . . G G G G G . . . . . . . . . . G G G G . . . . . . . . . . . . . . . . . . . . . . . . . . . . . . . . . . . . . . . . . . . . . . . . . . . . . . . . . . . . . . . . . . . . . . . . . . . . . . . . . . . . . . . . . . . . . . . . . . . . . . . . . . . . . . . . . . . . . . . . . . . . . . . . . . . . . . . . . . . . . . . .    
176	. . . . . T T T . . T T . . . . . . . . . . T T T T T . . . . . . . . . . T T T T . . . . . . . . . . . . . . . . . . . . . . . . . . . . . . . . . . . . . . . . . . . . . . . . . . . . . . . . . . . . . . . . . . . . . . . . . . . . . . . . . . . . . . . . . . . . . . . . . . . . . . . . . . . . . . . . . . . . . . . . . . . . . . . . . . . . . . . . . . . . . . . .    
177	. . . . . T T T . . T T . . . . . . . . . . T T T T T . . . . . . . . . . T T T T . T . . . . . . . . . . . . . . . . . . . . . . . . . . . . . . . . . . . . . . . . . . . . . . . . . . . . . . . . . . . . . . . . . . . . . . . . . . . . . . . . . . . . . . . . . . . . . . . . . . . . . . . . . . . . . . . . . . . . . . . . . . . . . . . . . . . . . . . . . . . . . .    
178	. . . . . T T T . . T T . . . . . . . . . . T T T T T . . . . . . . . . . T T T T . T . . . . . . . . . . . . . . . . . . . . . . . . . . . . . . . . . . . . . . . . . . . . . . . . . . . . . . . . . . . . . . . . . . . . . . . . . . . . . . . . . . . . . . . . . . . . . . . . . . . . . . . . . . . . . . . . . . . . . . . . . . . . . . . . . . . . . . . . . . . . . .    
179	. . . . . T T T . . T T . . . . . . . . . . T T T T T . . . . . . . . . . T T T T . T . . . . . . . . . . . . . . . . . . . . . . . . . . . . . . . . . . . . . . . . . . . . . . . . . . . . . . . . . . . . . . . . . . . . . . . . . . . . . . . . . . . . . . . . . . . . . . . . . . . . . . . . . . . . . . . . . . . . . . . . . . . . . . . . . . . . . . . . . . . . . .    
180	. . . . . G G G . . G G . . . . . . . . . . G G G G G . . . . . . . . . . G G G G . G . . . . . . . . . . . . . . . . . . . . . . . . . . . . . . . . . . . . . . . . . . . . . . . . . . . . . . . . . . . . . . . . . . . . . . . . . . . . . . . . . . . . . . . . . . . . . . . . . . . . . . . . . . . . . . . . . . . . . . . . . . . . . . . . . . . . . . . . . . . . . .    
181	. . . . . G G G . . G G . . . . . . . . . . G G G G G . . . . . . . . . . G G G G . G . . . . . . . . . . . . . . . . . . . . . . . . . . . . . . . . . . . . . . . . . . . . . . . . . . . . . . . . . . . . . . . . . . . . . . . . . . . . . . . . . . . . . . . . . . . . . . . . . . . . . . . . . . . . . . . . . . . . . . . . . . . . . . . . . . . . . . . . . . . . . .    
182	. . . . . G G G . . G G . . . . . . . . . . G G G G G . . . . . . . . . . G G G G . G . . . . . . . . . . . . . . . . . . . . . . . . . . . . . . . . . . . . . . . . . . . . . . . . . . . . . . . . . . . . . . . . . . . . . . . . . . . . . . . . . . . . . . . . . . . . . . . . . . . . . . . . . . . . . . . . . . . . . . . . . . . . . . . . . . . . . . . . . . . . . .    
183	. . . . . T T T . . T T . . . . . . . . . . T T T T T . . . . . . . . . . T T T T . T . . . . . . . . . . . . . . . . . . . . . . . . . . . . . . . . . . . . . . . . . . . . . . . . . . . . . . . . . . . . . . . . . . . . . . . . . . . . . . . . . . . . . . . . . . . . . . . . . . . . . . . . . . . . . . . . . . . . . . . . . . . . . . . . . . . . . . . . . . . . . .    
184	. . . . . G G G . . G G . . . . . . . . . . G G G G G . . . . . . . . . . G G G G . G . . . . . . . . . . . . . . . . . . . . . . . . . . . . . . . . . . . . . . . . . . . . . . . . . . . . . . . . . . . . . . . . . . . . . . . . . . . . . . . . . . . . . . . . . . . . . . . . . . . . . . . . . . . . . . . . . . . . . . . . . . . . . . . . . . . . . . . . . . . . . .    
185	. . . . . A A A . . A A . . . . . . . . . . A A A A A . . . . . . . . . . A A A A . A . . . . . . . . . . . . . . . . . . . . . . . . . . . . . . . . . . . . . . . . . . . . . . . . . . . . . . . . . . . . . . . . . . . . . . . . . . . . . . . . . . . . . . . . . . . . . . . . . . . . . . . . . . . . . . . . . . . . . . . . . . . . . . . . . . . . . . . . . . . . . .    
186	. . . . . G G G . . A G . . . . . . . . . . G G G G G . . . . . . . . . . G G G G . G . . . . . . . . . . . . . . . . . . . . . . . . . . . . . . . . . . . . . . . . . . . . . . . . . . . . . . . . . . . . . . . . . . . . . . . . . . . . . . . . . . . . . . . . . . . . . . . . . . . . . . . . . . . . . . . . . . . . . . . . . . . . . . . . . . . . . . . . . . . . . .    
187	. . . . . C C C . . C C . . . . . . . . . . C C C C C . . . . . . . . . . C C C C . C . . . . . . . . . . . . . . . . . . . . . . . . . . . . . . . . . . . . . . . . . . . . . . . . . . . . . . . . . . . . . . . . . . . . . . . . . . . . . . . . . . . . . . . . . . . . . . . . . . . . . . . . . . . . . . . . . . . . . . . . . . . . . . . . . . . . . . . . . . . . . .    
188	. . . . . C C C . . C C . . . . . . . . . . C C C C C . . . . . . . . . . C C C C . C . . . . . . . . . . . . . . . . . . . . . . . . . . . . . . . . . . . . . . . . . . . . . . . . . . . . . . . . . . . . . . . . . . . . . . . . . . . . . . . . . . . . . . . . . . . . . . . . . . . . . . . . . . . . . . . . . . . . . . . . . . . . . . . . . . . . . . . . . . . . . .    
189	. . . . . A A A . . A A . . . . . . . . . . A A A A A . . . . . . . . . . A A A A . A . . . . . . . . . . . . . . . . . . . . . . . . . . . . . . . . . . . . . . . . . . . . . . . . . . . . . . . . . . . . . . . . . . . . . . . . . . . . . . . . . . . . . . . . . . . . . . . . . . . . . . . . . . . . . . . . . . . . . . . . . . . . . . . . . . . . . . . . . . . . . .    
190	. . . . . T T T . . T T . . . . . . . . . . T T T T T . . . . . . . . . . T T T T . T . . . . . . . . . . . . . . . . . . . . . . . . . . . . . . . . . . . . . . . . . . . . . . . . . . . . . . . . . . . . . . . . . . . . . . . . . . . . . . . . . . . . . . . . . . . . . . . . . . . . . . . . . . . . . . . . . . . . . . . . . . . . . . . . . . . . . . . . . . . . . .    
191	. . . . . T T T . . T T . . . . . . . . . . T T T T T . . . . . . . . . . T T T T . T . . . . . . . . . . . . . . . . . . . . . . . . . . . . . . . . . . . . . . . . . . . . . . . . . . . . . . . . . . . . . . . . . . . . . . . . . . . . . . . . . . . . . . . . . . . . . . . . . . . . . . . . . . . . . . . . . . . . . . . . . . . . . . . . . . . . . . . . . . . . . .    
192	. . . . . T T T . . T T . . . . . . . . . . T T T T T . . . . . . . . . . T T T T . T . . . . . . . . . . . . . . . . . . . . . . . . . . . . . . . . . . . . . . . . . . . . . . . . . . . . . . . . . . . . . . . . . . . . . . . . . . . . . . . . . . . . . . . . . . . . . . . . . . . . . . . . . . . . . . . . . . . . . . . . . . . . . . . . . . . . . . . . . . . . . .    
193	C . . . . C C C . . C C . . . . . C . . . . C C C C C . . . . . . . . . . C C C C . C . . . . . . . . . . . . . . . . . . . . . . . . . . . . . . . . . . . . . . . . . . . . . . . C . . . . . . . . . . . . . . . . . . . . . . . . . . . . . . . . . . . . . . . . . . . . . . . . . . . . . . . . . . . . . . . . . . . . . . . . . . . . . . . . . . . . . . . . . . . . . .    
194	A A A . A A A A A . A A A . . . . A A . . . A A A A A A . A A . . . . . . A A A A A A A A A . . . A A A A A A . A . A . . . A A . A . . A A . . A A A A A A . . . . . . . . . . . . A . . . . . . . . . . . . . . . . . . . . . . . . . . . . . . . . . . . . . . . . . . . . . . . . . . . . . . . . . . . . . . . . . . . . . . . . . . . . . . . . . . . . . . . . . . . . . .    
195	G G G . G G G G G . G G G . . . . G G . . . G G G G G G . G G . . . . . . G G G G G G G G G . G . G G G G G G . G . G . . . G G . G . . G G . . G G G G G G . . . . . . . . . . . . G . . . . . . . . . . . . . . . . . . . . . . . . . . . . . . . . . . . . . . . . . . . . . . . . . . . . . . . . . . . . . . . . . . . . . . . . . . . . . . . . . . . . . . . . . . . . . .    
196	A A A . A A A A A . A A A . . . . A A . . . A A A A A A . A A . . . . . . A A A A A A A A A . A . A A A A A A . A . A . . . A A . A . . A A . . A A A A A A . . . . . . . . . . . . A . . . . . . . . . . . . . . . . . . . . . . . . . . . . . . . . . . . . . . . . . . . . . . . . . . . . . . . . . . . . . . . . . . . . . . . . . . . . . . . . . . . . . . . . . . . . . .    
197	T T T . T T T T T . T T T . . . . T T . . . T T T T T T . T T . . . . . . T T T T T T T T T . T . T T T T T T . T . T . . . T T . T . . T T . . T T T T T T . . . . . . . . . . . . T . . . . . . . . . . . . . . . . . . . . . . . . . . . . . . . . . . . . . . . . . . . . . . . . . . . . . . . . . . . . . . . . . . . . . . . . . . . . . . . . . . . . . . . . . . . . . .    
198	T T T . T T T T T . T T T . . . . T T . . . T T T T T T . T T . . . . . . T T T T T T T T T . T . T T T T T T . T . T . . . T T . T . . T T . . T T T T T T . . . . . . . . . . . . T . . . . . . . . . . . . . . . . . . . . . . . . . . . . . . . . . . . . . . . . . . . . . . . . . . . . . . . . . . . . . . . . . . . . . . . . . . . . . . . . . . . . . . . . . . . . . .    
199	G G G . G G G G G . G G G . . . . G G . . . G G G G G G . G G . . . . . . G G G G G G G G G . G . G G G G G G . G . G . . . G G . G . . G G . . G G G G G G . . . . . . . . . . G . G . . . . . . . . . . . . . . . . . . . . . . . . . . . . . . . . . . . . . . . . . . . . . . . . . . . . . . . . . . . . . . . . . . . . . . . . . . . . . . . . . . . . . . . . . . . . . .    
200	A A A . A A A A A . A A A . . . . A A . . . A A A A A A . A A . . . . . . A A A A A A A A A . A . A A A A A A . A . A . . . A A . A . . A A . . A A A A A A . . . . . . . . . . A . A . . . . . . . . . . . . . . . . . . . . . . . . . . . . . . . . . . . . . . . . . . . . . . . . . . . . . . . . . . . . . . . . . . . . . . . . . . . . . . . . . . . . . . . . . . . . . .    
201	A A A . A A A A A . A A A . . . . A A . . . A A A A A A . A A . . . . . . A A A A A A A A A . A . A A A A A A . A . A . . . A A . A . . A A . . A A A A A A . . . . . . . . . . A . A . . . . . . . . . . . . . . . . . . . . . . . . . . . . . . . . . . . . . . . . . . . . . . . . . . . . . . . . . . . . . . . . . . . . . . . . . . . . . . . . . . . . . . . . . . . . . .    
202	A A A . A A A A A . A A A . . . . A A . . . A A A A A A . A A . . . . . . A A A A A A A A A . A . A A A A A A . A . A . . . A A . A . . A A . . A A A A A A . . . . . . . . . . A . A . . . . . . . . . . . . . . . . . . . . . . . . . . . . . . . . . . . . . . . . . . . . . . . . . . . . . . . . . . . . . . . . . . . . . . . . . . . . . . . . . . . . . . . . . . . . . .    
203	C C C . C C C C C . C C C . . . . C C . . . C C C C C C . C C . . . . . . C C C C C C C C C . C . C C C C C C . C . C . . . C C . C . . C C . . C C C C C C . . . . . . . . . . C . C . . . . . . . . . . . . . . . . . . . . . . . . . . . . . . . . . . . . . . . . . . . . . . . . . . . . . . . . . . . . . . . . . . . . . . . . . . . . . . . . . . . . . . . . . . . . . .    
204	C C C . C C C C C . C C C . . . . C C . . . C C C C C C . C C . . . . . . C C C C C C C C C . C . C C C C C C . C . C . . . C C . C . . C C . . C C C C C C . . . . . . . . . . C . C . . . . . . . . . . . . . . . . . . . . . . . . . . . . . . . . . . . . . . . . . . . . . . . . . . . . . . . . . . . . . . . . . . . . . . . . . . . . . . . . . . . . . . . . . . . . . .    
205	C C C . C C C C C . C C C . . . . C C . . C C C C C C C . C C . . . . . C C C C C C C C C C . C . C C C C C C . C C C . . . C C . C . . C C . . C C C C C C . . . . . . . . . . C . C . . . . . . . . . . . . . . . . . . . . . . . . . . . . . . . . . . . . . . . . . . . . . . . . . . . . . . . . . . . . . . . . . . . . . . . . . . . . . . . . . . . . . . . . . . . . . .    
206	C C C . C C C C C . C C C . . . . C C . . C C C C C C C . C C . . . . C C C C C C C C C C C . C . C C C C C C . C C C . . . C C . C C . C C . . C C C C C C C . . . . . . . . . C . C . . . . . . . . . . . . . . . . . . . . . . . . . . . . . . . . . . . . . . . . . . . . . . . . . . . . . . . . . . . . . . . . . . . . . . . . . . . . . . . . . . . . . . . . . . . . . .    
207	C C C . C C C C C . C C C . . . . C C . . C C C C C C C . C C . . . . C C C C C C C C C C C . C . C C C C C C . C C C . . . C C . C C . C C . . C C C C C C C . . . . . . . . . C . C . . . . . . . . . . . . . . . . . . . . . . . . . . . . . . . . . . . . . . . . . . . . . . . . . . . . . . . . . . . . . . . . . . . . . . . . . . . . . . . . . . . . . . . . . . . . . .    
208	T T T . T T T T T . T T T . . . . T T . . T T T T T T T . T T . . . . T T T T T T T T T T T . T . T T T T T T . T T T . . . T T . T T . T T . . T T T T T T T . . . . . . . . . T . T . . . . . . . . . . . . . . . . . . . . . . . . . . . . . . . . . . . . . . . . . . . . . . . . . . . . . . . . . . . . . . . . . . . . . . . . . . . . . . . . . . . . . . . . . . . . . .    
209	T T T . T T T T T . T T T . . . . T T . . T T T T T T T . T T . . . . T T T T T T T T T T T . T . T T T T T T . T T T . . . T T . T T . T T . . T T T T T T T . . . . . . . . . T . T . . . . . . . . . . . . . . . . . . . . . . . . . . . . . . . . . . . . . . . . . . . . . . . . . . . . . . . . . . . . . . . . . . . . . . . . . . . . . . . . . . . . . . . . . . . . . .    
210	T T T . T T T T T . T T T . . . . T T . . T T T T T T T . T T . . . . T T T T T T T T T T T . T . T T T T T T . T T T . . . T T . T T . T T . . T T T T T T T . . . . . . . . . T . T . . . . . . . . . . . . . . . . . . . . . . . . . . . . . . . . . . . . . . . . . . . . . . . . . . . . . . . . . . . . . . . . . . . . . . . . . . . . . . . . . . . . . . . . . . . . . .    
211	A A A A A A A A A . A A A . . . . A A . . A A A A A A A . A A . . . . A A A A A A A A A A A A A . A A A A A A . A A A . . . A A . A A . A A . . A A A A A A A . . . . . . . . . A . A . . . . . . . . . . . . . . . . . . . . . . . . . . . . . . . . . . . . . . . . . . . . . . . . . . . . . . . . . . . . . . . . . . . . . . . . . . . . . . . . . . . . . . . . . . . . . .    
212	A A A A A A A A A . A A A . A . . A A . . A A A A A A A . A A . . . . A A A A A A A A A A A A A . A A A A A A . A A A . A . A A . A A . A A . . A A A A A A A . . . . . . . . . A . A . . . . . . . . . . . . . . . . . . . . . . . . . . . . . . . . . . . . . . . . . . . . . . . . . . . . . . . . . . . . . . . . . . . . . . . . . . . . . . . . . . . . . . . . . . . . . .    
213	A A A A A A A A A . A A A . A . . A A . . A A A A A A A . A A . . . . A A A A A A A A A A A A A . A A A A A A . A A A . A . A A . A A . A A . . A A A A A A A . . . . . . . . . A . A . . . . . . . . . . . . . . . . . . . . . . . . . . . . . . . . . . . . . . . . . . . . . . . . . . . . . . . . . . . . . . . . . . . . . . . . . . . . . . . . . . . . . . . . . . . . . .    
214	A A A A A A A A A . A A A . A . . A A . . A A A A A A A . A A . . . . A A A A A A A A A A A A A . A A A A A A . A A A . A . A A . A A . A A . . A A A A A A A . . . . . . . . . A . A . . . . . . . . . . . . . . . . . . . . . . . . . . . . . . . . . . . . . . . . . . . . . . . . . . . . . . . . . . . . . . . . . . . . . . . . . . . . . . . . . . . . . . . . . . . . . .    
215	G G G G G G G G G . G G G . G . . G G . . G G G G G G G . G G . . . . G G G G G G G G G G G G G . G G G G G G . G G G . G . G G . G G . G G . . G G G G G G G . . . . . . . . . G . G . . . . . . . . . . . . . . . . . . . . . . . . . . . . . . . . . . . . . . . . . . . . . . . . . . . . . . . . . . . . . . . . . . . . . . . . . . . . . . . . . . . . . . . . . . . . . .    
216	G G G G G G G G G . G G G . G . . G G . . G G G G G G G . G G . . . . G G G G G G G G G G G G G . G G G G G G . G G G . G . G G . G G . G G . . G G G G G G G . . . . . . . . . G . G . . . . . . . . . . . . . . . . . . . . . . . . . . . . . . . . . . . . . . . . . . . . . . . . . . . . . . . . . . . . . . . . . . . . . . . . . . . . . . . . . . . . . . . . . . . . . .    
217	T T T T T T T T T . T T T . T . . T T . . T T T T T T T . T T . . . . T T T T T T T T T T T T T . T T T T T T . T T T T T . T T T T T . T T . . T T T T T T T . . . . . . . . . T T T . . . . . . . . . . . . . . . . . . . . . . . . . . . . . . . . . . . . . . . . . . . . . . . . . . . . . . . . . . . . . . . . . . . . . . . . . . . . . . . . . . . . . . . . . . . . . .    
218	T T T T T T T T T . T T T . T . . T T . . T T T T T T T . T T . . . . T T T T T T T T T T T T T . T T T T T T . T T T T T . T T T T T . T T . . T T T T T T T . . . . . . . . . T T T . . . . . . . . . . . . . . . . . . . . . . . . . . . . . . . . . . . . . . . . . . . . . . . . . . . . . . . . . . . . . . . . . . . . . . . . . . . . . . . . . . . . . . . . . . . . . .    
219	G G G G G G G G G . G G G . G . . G G . . G G G G G G G . G G . . . . G G G G G G G G G G G G G . G G G G G G . G G G G G . G G G G G . G G . . G G G G G G G . . . . . . . . . G G G G . . . . . . . . . . . . . . . . . . . . . . . . . . . . . . . . . . . . . . . . . . . . . . . . . . . . . . . . . . . . . . . . . . . . . . . . . . . . . . . . . . . . . . . . . . . . .    
220	T T T T T T T T T . T T T . T . . T T . . T T T T T T T . T T . . . . T T T T T T T T T T T T T . T T T T T T . T T T T T . T T T T T . T T . . T T T T T T T . . . . . . . . . T T T T . . . . . . . . . . . . . . . . . . . . . . . . . . . . . . . . . . . . . . . . . . . . . . . . . . . . . . . . . . . . . . . . . . . . . . . . . . . . . . . . . . . . . . . . . . . . .    
221	A A A A A A A A A . A A A . A . . A A . . A A A A A A A . A A . . . . A A A A A A A A A A A A A . A A A A A A . A A A A A . A A A A A . A A . . A A A A A A A . . . . . . . . . A A A A . . . . . . . . . . . . . . . . . . . . . . . . . . . . . . . . . . . . . . . . . . . . . . . . . . . . . . . . . . . . . . . . . . . . . . . . . . . . . . . . . . . . . . . . . . . . .    
222	A A A A A A A A A . A A A . A . . A A . . A A A A A A A . A A . . . . A A A A A A A A A A A A A . A A A A A A . A A A A A . A A A A A . A A . . A A A A A A A . . . . . . . . . A A A A . . . . . . . . . . . . . . . . . . . . . . . . . . . . . . . . . . . . . . . . . . . . . . . . . . . . . . . . . . . . . . . . . . . . . . . . . . . . . . . . . . . . . . . . . . . . .    
223	C C C C C C C C C . C C C . C . . C C . . C C C C C C C . C C . . . . C C C C C C C C C C C C C . C C C C C C . C C C C C C C C C C C . C C . . C C C C C C C . . . . . . . . . C C C C . . . . . . . . . . . . . . . . . . . . . . . . . . . . . . . . . . . . . . . . . . . . . . . . . . . . . . . . . . . . . . . . . . . . . . . . . . . . . . . . . . . . . . . . . . . . .    
224	A A A A A A A A A . A A A A A . . A A . . A A A A A A A . A A . . . A A A A A A A A A A A A A A . A A A A A A . A A A A A A A A A A A . A A . . A A A A A A A . . . . . . . . . A A A A . . . . . . . . . . . . . . . . . . . . . . . . . . . . . . . . . . . . . . . . . . . . . . . . . . . . . . . . . . . . . . . . . . . . . . . . . . . . . . . . . . . . . . . . . . . . .    
225	G G G G G G G G G . G G G G G . . G G . . G G G G G G G . G G . . . G G G G G G G G G G G G G G . G G G G G G . G G G G G G G G G G G . G G . . G G G G G G G . . . . . . . . . G G G G . . . . . . . . . . . . . . . . . . . . . . . . . . . . . . . . . . . . . . . . . . . . . . . . . . . . . . . . . . . . . . . . . . . . . . . . . . . . . . . . . . . . . . . . . . . . .    
226	A A A A A A A A A . A A A A A . . A A . . A A A A A A A . A A . . . A A A A A A A A A A A A A A . A A A A A A . A A A A A A A A A A A . A A . . A A A A A A A . . . . . . . . . A A A A . . . . . . . . . . . . . . . . . . . . . . . . . . . . . . . . . . . . . . . . . . . . . . . . . . . . . . . . . . . . . . . . . . . . . . . . . . . . . . . . . . . . . . . . . . . . .    
227	G G G G G G G G G . G G G G G . . G G . . G G G G G G G . G G . . . G G G G G G G G G G G G G G . G G G G G G . G G G G G G G G G G G . G G . . G G G G G G G . . . . . . . . . G G G G . . . . . . . . . . . . . . . . . . . . . . . . . . . . . . . . . . . . . . . . . . . . . . . . . . . . . . . . . . . . . . . . . . . . . . . . . . . . . . . . . . . . . . . . . . . . .    
228	A A A A A A A A A . A A A A A . . A A . A A A A A A A A . A A . . . A A A A A A A A A A A A A A . A A A A A A . A A A A A A A A A A A . A A . . A A A A A A A . . . . . . . . . A A A A . . . . . . . . . . . . . . . . . . . . . . . . . . . . . . . . . . . . . . . . . . . . . . . . . . . . . . . . . . . . . . . . . . . . . . . . . . . . . . . . . . . . . . . . . . . . .    
229	G G G G G G G G G . G G G G G . . G G . G G G G G G G G . G G . . . G G G G G G G G G G G G G G . G G G G G G . G G G G G G G G G G G . G G . . G G G G G G G . . . . . . . . . G G G G . . . . . . . . . . . . . . . . . . . . . . . . . . . . . . . . . . . . . . . . . . . . . . . . . . . . . . . . . . . . . . . . . . . . . . . . . . . . . . . . . . . . . . . . . . . . .    
230	A A A A A A A A A . A A A A A . . A A . A A A A A A A A . A A . . . A A A A A A A A A A A A A A . A A A A A A . A A A A A A A A A A A . A A . . A A A A A A A . A . . . . . . . A A A A . . . . . . . . . . . . . . . . . . . . . . . . . . . . . . . . . . . . . . . . . . . . . . . . . . . . . . . . . . . . . . . . . . . . . . . . . . . . . . . . . . . . . . . . . . . . .    
231	C C C C C C C C C . C C C C C . . C C . C C C C C C C C . C C . . C C C C C C C C C C C C C C C . C C C C C C . C C C C C C C C C C C C C C . . C C C C C C C . C . . . . . . . C C C C . . . . . . . . . . . . . . . . . . . . . . . . . . . . . . . . . . . . . . . . . . . . . . . . . . . . . . . . . . . . . . . . . . . . . . . . . . . . . . . . . . . . . . . . . . . . .    
232	T T T T T T T T T . T T T T T . . T T . T T T T T T T T . T T . . T T T T T T T T T T T T T T T . T T T T T T . T T T T T T T T T T T T T T . . T T T T T T T . T . . . . . . . T T T T . . . . . . . . . . . . . . . . . . . . . . . . . . . . . . . . . . . . . . . . . . . . . . . . . . . . . . . . . . . . . . . . . . . . . . . . . . . . . . . . . . . . . . . . . . . . .    
233	G G G G G G G G G . G G G G G G . G G . G G G G G G G G . G G . . G G G G G G G G G G G G G G G . G G G G G G . G G G G G G G G G G G G G G . . G G G G G G G . G . . . . . . . G G G G . . . . . . . . . . . . . . . . . . . . . . . . . . . . . . . . . . . . . . . . . . . . . . . . . . . . . . . . . . . . . . . . . . . . . . . . . . . . . . . . . . . . . . . . . . . . .    
234	C C C C C C C C C . C C C C C C . C C . C C C C C C C C . C C . . C C C C C C C C C C C C C C C . C C C C C C . C C C C C C C C C C C C C C . . C C C C C C C . C . . . . . . . C C C C . . . . . . . . . . . . . . . . . . . . . . . . . . . . . . . . . . . . . . . . . . . . . . . . . . . . . . . . . . . . . . . . . . . . . . . . . . . . . . . . . . . . . . . . . . . . .    
235	T T T T T T T T T . T T T T T T . T T . T T T T T T T T . T T . . T T T T T T T T T T T T T T T . T T T T T T . T T T T T T T T T T T T T T . . T T T T T T T . T . . . . . . . T T T T . . . . . . . . . . . . . . . . . . . . . . . . . . . . . . . . . . . . . . . . . . . . . . . . . . . . . . . . . . . . . . . . . . . . . . . . . . . . . . . . . . . . . . . . . . . . .    
236	A A A A A A A A A . A A A A A A . A A . A A A A A A A A . A A . . A A A A A A A A A A A A A A A . A A A A A A . A A A A A A A A A A A A A A . . A A A A A A A . A . . . . . . . A A A A . . . . . . . . . . . . . . . . . . . . . . . . . . . . . . . . . . . . . . . . . . . . . . . . . . . . . . . . . . . . . . . . . . . . . . . . . . . . . . . . . . . . . . . . . . . . .    
237	C C C C C C C C C . C C C C C C . C C . C C C C C C C C . C C . . C C C C C C C C C C C C C C C . C C C C C C . C C C C C C C C C C C C C C . . C C C C C C C . C . . . . . . . C C C C . . . . . . . . . . . . . . . . . . . . . . . . . . . . . . . . . . . . . . . . . . . . . . . . . . . . . . . . . . . . . . . . . . . . . . . . . . . . . . . . . . . . . . . . . . . . .    
238	A A A A A A A A A . A A A A A A . A A . A A A A A A A A . A A . . A A A A A A A A A A A A A A A . A A A A A A . A A A A A A A A A A A A A A . A A A A A A A A . A . . . . . . . A A A A . . . . . . . . . . . . . . . . . . . . . . . . . . . . . . . . . . . . . . . . . . . . . . . . . . . . . . . . . . . . . . . . . . . . . . . . . . . . . . . . . . . . . . . . . . . . .    
239	G G G G G G G G G . G G G G G G . G G . G G G G G G G G . G G . . G G G G G G G G G G G G G G G . G G G G G G . G G G G G G G G G G G G G G . G G G G G G G G . G . . . . . . . G G G G . . . . . . . . . . . . . . . . . . . . . . . . . . . . . . . . . . . . . . . . . . . . . . . . . . . . . . . . . . . . . . . . . . . . . . . . . . . . . . . . . . . . . . . . . . . . .    
240	G G G G G G G G G . G G G G G G . G G . G G G G G G G G . G G . . G G G G G G G G G G G G G G G . G G G G G G . G G G G G G G G G G G G G G . G G G G G G G G . G . . . . . . . G G G G . . . . . . . . . . . . . . . . . . . . . . . . . . . . . . . . . . . . . . . . . . . . . . . . . . . . . . . . . . . . . . . . . . . . . . . . . . . . . . . . . . . . . . . . . . . . .    
241	A A A A A A A A A . A A A A A A A A A . A A A A A A A A . A A . . A A A A A A A A A A A A A A A . A A A A A A . A A A A A A A A A A A A A A . A A A A A A A A . A . . . . . . . A A A A . . . . . . . . . . . . . . . . . . . . . . . . . . . . . . . . . . . . . . . . . . . . . . . . . . . . . . . . . . . . . . . . . . . . . . . . . . . . . . . . . . . . . . . . . . . . .    
242	T T T T T T T T T . T T T T T T T T T . T T T T T T T T . T T . . T T T T T T T T T T T T T T T . T T T T T T . T T T T T T T T T T T T T T . T T T T T T T T . T . . . . . . . T T T T . . . . . . . . . . . . . . . . . . . . . . . . . . . . . . . . . . . . . . . . . . . . . . . . . . . . . . . . . . . . . . . . . . . . . . . . . . . . . . . . . . . . . . . . . . . . .    
243	G G G G G G G G G . G G G G G G G G G G G G G G G G G G . G G . . G G G G G G G G G G G G G G G . G G G G G G . G G G G G G G G G G G G G G . G G G G G G G G . G . . . . . . . G G G G . . . . . . . . . . . . . . . . . . . . . . . . . . . . . . . . . . . . . . . . . . . . . . . . . . . . . . . . . . . . . . . . . . . . . . . . . . . . . . . . . . . . . . . . . . . . .    
244	T T T T T T T T T . T T T T T T T T T T T T T T T T T T . T T . . T T T T T T T T T T T T T T T . T T T T T T . T T T T T T T T T T T T T T . T T T T T T T T . T . . . . . . . T T T T . . . . . . . . . . . . . . . . . . . . . . . . . . . . . . . . . . . . . . . . . . . . . . . . . . . . . . . . . . . . . . . . . . . . . . . . . . . . . . . . . . . . . . . . . . . . .    
245	C C C C C C C C C . C C C C C C C C C C C C C C C C C C C C C . . C C C C C C C C C C C C C C C . C C C C C C . C C C C C C C C C C C C C C . C C C C C C C C . C . . . . . . . C C C C . . . . . . . . . . . . . . . . . . . . . . . . . . . . . . . . . . . . . . . . . . . . . . . . . . . . . . . . . . . . . . . . . . . . . . . . . . . . . . . . . . . . . . . . . . . . .    
246	T T T T T T T T T . T T T T T T T T T T T T T T T T T T T T T . . T T T T T T T T T T T T T T T . T T T T T T . T T T T T T T T T T T T T T . T T T T T T T T . T . . . . . . . T T T T . . . . . . . . . . . . . . . . . . . . . . . . . . . . . . . . . . . . . . . . . . . . . . . . . . . . . . . . . . . . . . . . . . . . . . . . . . . . . . . . . . . . . . . . . . . . .    
247	G G G G G G G G G . G G G G G G G G G G G G G G G G G G G G G . . G G G G G G G G G G G G G G G . G G G G G G . G G G G G G G G G G G G G G . G G G G G G G G . G . . . . . . . G G G G . . . . . . . . . G . . . . . . . . . . . . . . . . . . . . . . . . . . . . . . . . . . . . . . . . . . . . . . . . . . . . . . . . . . . . . . . . . . . . . . . . . . . . . . . . . . .    
248	A A A A A A A A A . A A A A A A A A A A A A A A A A A A A A A . . A A A A A A A A A A A A A A A . A A A A A A . A A A A A A A A A A A A A A . A A G A A A A A . A . . . . . . . A A A A . . . . . . . . . A . . . . . . . . . . . . . . . . . . . . . . . . . . . . . . . . . . . . . . . . . . . . . . . . . . . . . . . . . . . . . . . . . . . . . . . . . . . . . . . . . . .    
249	G G G G G G G G G . G G G G G G G G G G G G G G G G G G G G G . . G G G G G G G G G G G G G G G . G G G G A G . G G G G G G G G G G G G G G . G G G G G G G G . G . . . . . . . G G G G . . . . . . . . . G . . . . . . . . . . . . . . . . . . . . . . . . . . . . . . . . . . . . . . . . . . . . . . . . . . . . . . . . . . . . . . . . . . . . . . . . . . . . . . . . . . .    
250	T T T T T T T T T . T T T T T T T T T T T T T T T T T T T T T . . T T T T T T T T T T T T T T T . T T T T T T . T T T T T T T T T T T T T T . T T T T T T T T . T . . . . . . . T T T T . . . . . . . . . T . . . . . . . . . . . . . . . . . . . . . . . . . . . . . . . . . . . . . . . . . . . . . . . . . . . . . . . . . . . . . . . . . . . . . . . . . . . . . . . . . . .    
251	C C C C C C C C C . C C C C C C C C C C C C C C C C C C C C C . . C C C C C C C C C C C C C C C . C C C C C C . C C C C C C C C C C C C C C . C C C C C C C C . C . . . . . . . C C C C . . . . . . . . . C . . . . . . . . . . . . . . . . . . . . . . . . . . . . . . . . . . . . . . . . . . . . . . . . . . . . . . . . . . . . . . . . . . . . . . . . . . . . . . . . . . .    
252	A A A A A A A A A . A A A A A A A A A A A A A A A A A A A A A . . A A A A A A A A A A A A A A A . A A A A A A . A A A A A A A A A A A A A A . A A A A A A A A . A . . . . . . . A A A A . . . . . . . . . A . . . . . . . . . . . . . . . . . . . . . . . . . . . . . . . . . . . . . . . . . . . . . . . . . . . . . . . . . . . . . . . . . . . . . . . . . . . . . . . . . . .    
253	C C C C C C C C C . C C C C C C C C C C C C C C C C C C C C C . . C C C C C C C C C C C C C C C . C C C C C C . C C C C C C C C C C C C C C . C C C C C C C C . C . . . . . . . C C C C . . . . . . . . . C . . . . . . . . . . . . . . . . . . . . . . . . . . . . . . . . . . . . . . . . . . . . . . . . . . . . . . . . . . . . . . . . . . . . . . . . . . . . . . . . . . .    
254	A A A A A A A A A . A A A A A A A A A A A A A A A A A A A A A . . A A A A A A A A A A A A A A A . A A A A A A . A A A A A A A A A A A A A A . A A A A A A A A . A . . . . . . . A A A A . . . . . . . . . A . . . . . . . . . . . . . . . . . . . . . . . . . . . . . . . . . . . . . . . . . . . . . . . . . . . . . . . . . . . . . . . . . . . . . . . . . . . . . . . . . . .    
255	G G G G G G G G G . G G G G G G G G G G G G G G G G G G G G G . . G G G G G G G G G G G G G G G . G G G G G G . G G G G G G G G G G G G G G . G G G G G G G G . G . . . . . . . G G G G . . . . . . . . . G . . . . . . . . . . . . . . . . . . . . . . . . . . . . . . . . . . . . . . . . . . . . . . . . . . . . . . . . . . . . . . . . . . . . . . . . . . . . . . . . . . .    
256	A A A A A A A A A . A A G A A A A A A A A A A A A A A A A A A . . A A A A A A A A A A A A A A A . A A A A A A . A A A A A A A A A A A A A A . A A A A A A A A . A . . . . . . . A A A A . . . . . . . . . A . . . . . . . . . . . . . . . . . . . . . . . . . . . . . . . . . . . . . . . . . . . . . . . . . . . . . . . . . . . . . . . . . . . . . . . . . . . . . . . . . . .    
257	G G G G G G G G G . G G G G G G G G G G G G G G G G G G G G G . . G G G G G G G G G G G G G G G . G G G G G G . G G G G G G G G G G G G G G . G G G G G G G G . G . . . . . . . G G G G . . . . . . . . . G . . . . . . . . . . . . . . . . . . . . . . . . . . . . . . . . . . . . . . . . . . . . . . . . . . . . . . . . . . . . . . . . . . . . . . . . . . . . . . . . . . .    
258	A A A A A A A A A . A A A A A A A A A A A A A A A A A A A A A . . A A A A A A A A A A A A A A A . A A A A A A . A A A A A A A A A A A A A A . A A A A A A A A . A . . . . . . . A A A A . . . . . . . . . A . . . . . . . . . . . . . . . . . . . . . . . . . . . . . . . . . . . . . . . . . . . . . . . . . . . . . . . . . . . . . . . . . . . . . . . . . . . . . . . . . . .    
259	C C C C C C C C C . C C C C C C C C C C C C C C C C C C C C C . . C C C C C C C C C C C C C C C . C C C C C C . C C C C C C C C C C C C C C . C C C C C C C C . C . . . . . . . C C C C . . . . . . . . . C . . . . . . . . . . . . . . . . . . . . . . . . . . . . . . . . . . . . . . . . . . . . . . . . . . . . . . . . . . . . . . . . . . . . . . . . . . . . . . . . . . .    
260	A A A A A A A A A . A A A A A A A A A A A A A A A A A A A A A . . A A A A A A A A A A A A A A A . A A A A A A . A A A A A A A A A A A A A A . A A A A A A A A . A . . . . . . . A A A A . . . . . . . . . A . . . . . . . . . . . . . . . . . . . . . . . . . . . . . . . . . . . . . . . . . . . . . . . . . . . . . . . . . . . . . . . . . . . . . . . . . . . . . . . . . . .    
261	G G G G G G G G G . G G G G G G G G G G G G G G G G G G G G G . . G G G G G G G G G G G G G G G . G G G G G G . G G G G G G G G G G G G G G . G G G G G G G G . G . . . . . . . G G G G . . . . . . . . . G . . . . . . . . . . . . . . . . . . . . . . . . . . . . . . . . . . . . . . . . . . . . . . . . . . . . . . . . . . . . . . . . . . . . . . . . . . . . . . . . . . .    
262	C C C C C C C C C . C C C C C C C C C C C C C C C C C C C C C . . C C C C C C C C C C C C C C C . C C C C C C . C C C C C C C C C C C C C C . C C C C C C C C . C . . . . . . . C C C C . . . . . . . . . C . . . . . . . . . . . . . . . . . . . . . . . . . . . . . . . . . . . . . . . . . . . . . . . . . . . . . . . . . . . . . . . . . . . . . . . . . . . . . . . . . . .    
263	C C C C C C C C C . C C C C C C C C C C C C C C C C C C C C C . . C C C C C C C C C C C C C C C . C C C C C C . C C C C C C C C C C C C C C . C C C C C C C C . C . . . . . . . C C C C . . . . . . . . . C . . . . . . . . . . . . . . . . . . . . . . . . . . . . . . . . . . . . . . . . . . . . . . . . . . . . . . . . . . . . . . . . . . . . . . . . . . . . . . . . . . .    
264	C C C C C C C C C . C C C C C C C C C C C C C C C C C C C C C . . C C C C C C C C C C C C C C C . C C C C C C . C C C C C C C C C C C C C C . C C C C C C C C . C . . . . . . . C C C C . . . . . . . . . C . . . . . . . . . . . . . . . . . . . . . . . . . . . . . . . . . . . . . . . . . . . . . . . . . . . . . . . . . . . . . . . . . . . . . . . . . . . . . . . . . . .    
265	C C C C C C C C C . C C C C C C C C C C C C C C C C C C C C C . . C C C C C C C C C C C C C C C . C C C C C C . C C C C C C C C C C C C C C . C C C C C C C C . C . . . . . . . C C C C . . . . . . . . . C . . . . . . . . . . . . . . . . . . . . . . . . . . . . . . . . . . . . . . . . . . . . . . . . . . . . . . . . . . . . . . . . . . . . . . . . . . . . . . . . . . .    
266	T T T T T T T T T . T T T T T T T T T T T T T T T T T T T T T . . T T T T T T T T T T T T T T T . T T T T T T . T T T T T T T T T T T T T T . T T T T T T T T . T . . . . . . . T T T T . . . . . . . . . T . . . . . . . . . . . . . . . . . . . . . . . . . . . . . . . . . . . . . . . . . . . . . . . . . . . . . . . . . . . . . . . . . . . . . . . . . . . . . . . . . . .    
267	C C C C C C C C C . C C C C C C C C C C C C C C C C C C C C C . . C C C C C C C C C C C C C C C . C C C C C C . C C C C C C C C C C C C C C . C C C C C C C C . C . . . . . . . C C C C . . . . . . . . . C . . . . . . . . . . . . . . . . . . . . . . . . . . . . . . . . . . . . . . . . . . . . . . . . . . . . . . . . . . . . . . . . . . . . . . . . . . . . . . . . . . .    
268	A A A A A A A A A . A A A A A A A A A A A A A A A A A A A A A . . A A A A A A A A A A A A A A A . A A A A A A . A A A A A A A A A A A A A A . A A A A A A A A . A . . . . . . . A A A A . . . . . . . . . A . . . . . . . . . . . . . . . . . . . . . . . . . . . . . . . . . . . . . . . . . . . . . . . . . . . . . . . . . . . . . . . . . . . . . . . . . . . . . . . . . . .    
269	T T T T T T T T T . T T T T T T T T T T T T T T T T T T T T T . . T T T T T T T T T T T T T T T . T T T T T T . T T T T T T T T T T T T T T . T T T T T T T T . T . . . . . . . T T T T . . . . . . . . . T . . . . . . . . . . . . . . . . . . . . . . . . . . . . . . . . . . . . . . . . . . . . . . . . . . . . . . . . . . . . . . . . . . . . . . . . . . . . . . . . . . .    
270	C C C C C C C C C . C C C C C C C C C C C C C C C C C C C C C . . C C C C C C C C C C C C C C C . C C C C C C . C C C C C C C C C C C C C C . C C C C C C C C . C . . . . . . . C C C C . . . . . . . . . C . . . . . . . . . . . . . . . . . . . . . . . . . . . . . . . . . . . . . . . . . . . . . . . . . . . . . . . . . . . . . . . . . . . . . . . . . . . . . . . . . . .    
271	G G G G G G G G G . G G G G G G G G G G G G G G G G G G G G G . . G G G G G G G G G G G G G G G . G G G G G G . G G G G G G G G G G G G G G . G G G G G G G G . G . . . . . . . G G G G . . . . . . . . . G . . . . . . . . . . . . . . . . . . . . . . . . . . . . . . . . . . . . . . . . . . . . . . . . . . . . . . . . . . . . . . . . . . . . . . . . . . . . . . . . . . .    
272	G G G G G G G G G . G G G G G G G G G G G G G G G G G G G G G . . G G G G G G G G G G G G G G G . G G G G G G . G G G G G G G G G G G G G G . G G G G G G G G . G . . . . . . . G G G G . . . . . . . G . G . . . . . . . . . . . . . . . . . . . . . . . . . . . . . . . . . . . . . . . . . . . . . . . . . . . . . . . . . . . . . . . . . . . . . . . . . . . . . . . . . . .    
273	A A A A A A A A A . A A A A A A A A A A A A A A A A A A A A A . . A A A A A A A A A A A A A A A . A A A A A A . A A A A A A A A A A A A A A . A A A A A A A A . A . . . . . . . A A A A . . . . . . . A . A . . . . . . . . . . . . . . . . . . . . . . . . . . . . . . . . . . . . . . . . . . . . . . . . . . . . . . . . . . . . . . . . . . . . . . . . . . . . . . . . . . .    
274	G G G G G G G G G . G G G G G G G G G G G G G G G G G G G G G . . G G G G G G G G G G G G G G G . G G G G G G . G G G G G G G G G G G G G G . G G G G G G G G . G . . . . . . . G G G G . . . . . . . G . G . . . . . . . . . . . . . . . . . . . . . . . . . . . . . . . . . . . . . . . . . . . . . . . . . . . . . . . . . . . . . . . . . . . . . . . . . . . . . . . . . . .    
275	C C C C C C C C C . C C C C C C C C C C C C C C C C C C C C C . . C C C C C C C C C C C C C C C . C C C C C C . C C C C C C C C C C C C C C . C C C C C C C C . C . . . . . . . C C C C . . . . . . . C . C . . . . . . . . . . . . . . . . . . . . . . . . . . . . . . . . . . . . . . . . . . . . . . . . . . . . . . . . . . . . . . . . . . . . . . . . . . . . . . . . . . .    
276	T T T T T T T T T . T T T T T T T T T T T T T T T T T T T T T . . T T T T T T T T T T T T T T T . T T T T T T . T T T T T T T T T T T T T T . T T T T T T T T . T . . . . . . . T T T T . . . . . . . T . T . . . . . . . . . . . . . . . . . . . . . . . . . . . . . . . . . . . . . . . . . . . . . . . . . . . . . . . . . . . . . . . . . . . . . . . . . . . . . . . . . . .    
277	T T T T T T T T T . T T T T T T T T T T T T T T T T T T T T T . . T T T T T T T T T T T T T T T . T T T T T T . T T T T T T T T T T T T T T . T T T T T T T T . T . . . . . . . T T T T . . . . . . . T . T . . . . . . . . . . . . . . . . . . . . . . . . . . . . . . . . . . . . . . . . . . . . . . . . . . . . . . . . . . . . . . . . . . . . . . . . . . . . . . . . . . .    
278	C C C C C C C C C . C C C C C C C C C C C C C C C C C C C C C . . C C C C C C C C C C C C C C C . C C C C C C . C C C C C C C C C C C C C C . C C C C C C C C . C . . . . . . . C C C C . . . . . . . C . C . . . . . . . . . . . . . . . . . . . . . . . . . . . . . . . . . . . . . . . . . . . . . . . . . . . . . . . . . . . . . . . . . . . . . . . . . . . . . . . . . . .    
279	C C C C C C C C C . C C C C C C C C C C C C C C C C C C C C C . . C C C C C C C C C C C C C C C . C C C C C C . C C C C C C C C C C C C C C . C C C C C C C C . C . . . . . . . C C C C . . . . . . . C . C . . . . . . . . . . . . . . . . . . . . . . . . . . . . . . . . . . . . . . . . . . . . . . . . . . . . . . . . . . . . . . . . . . . . . . . . . . . . . . . . . . .    
280	A A A A A A A A A . A A A A A A A A A A A A A A A A A A A A A . . A A A A A A A A A A A A A A A . A A A A A A . A A A A A A A A A A A A A A . A A A A A A A A . A . . . . . . . A A A A . . . . . . . A . A . . . . . . . . . . . . . . . . . . . . . . . . . . . . . . . . . . . . . . . . . . . . . . . . . . . . . . . . . . . . . . . . . . . . . . . . . . . . . . . . . . .    
281	G G G G G G G G G . G G G G G G G G G G G G G G G G G G G G G . . G G G G G G G G G G G G G G G . G G G G G G . G G G G G G G G G G G G G G . G G G G G G G G . G . . . . . . . G G G G . . . . . . . G . G . . . . . . . . . . . . . . . . . . . . . . . . . . . . . . . . . . . . . . . . . . . . . . . . . . . . . . . . . . . . . . . . . . . . . . . . . . . . . . . . . . .    
282	C C C C C C C C C . C C C C C C C C C C C C C C C C C C C C C . . C C C C C C C C C C C C C C C . C C C C C C . C C C C C C C C C C C C C C . C C C C C C C C . C . . . . . . . C C C C . . . . . . . C . C . . . . . . . . . . . . . . . . . . . . . . . . . . . . . . . . . . . . . . . . . . . . . . . . . . . . . . . . . . . . . . . . . . . . . . . . . . . . . . . . . . .    
283	G G G G G G G G G . G G G G G G G G G G G G G G G G G G G G G . . G G G G G G G G G G G G G G G . G G G G G G . G G G G G G G G G G G G G G . G G G G G G G G . G . . . . . . . G G G G . . . . . . . G . G . . . . . . . . . . . . . . . . . . . . . . . . . . . . . . . . . . . . . . . . . . . . . . . . . . . . . . . . . . . . . . . . . . . . . . . . . . . . . . . . . . .    
284	A A A A A A A A A . A A A A A A A A A A A A A A A A A A A A A . . A A A A A A A A A A A A A A A . A A A A A A . A A A A A A A A A A A A A A . A A A A A A A A . A . . . . . . . A A A A . . . . . . . A . A . . . . . . . . . . . . . . . . . . . . . . . . . . . . . . . . . . . . . . . . . . . . . . . . . . . . . . . . . . . . . . . . . . . . . . . . . . . . . . . . . . .    
285	G G G G G G G G G . G G G G G G G G G G G G G G G G G G G G G . . G G G G G G G G G G G G G G G . G G G G G G . G G G G G G G G G G G G G G . G G G G G G G G . G . . . . . . . G G G G . . . . . . . G . G . . . . . . . . . . . . . . . . . . . . . . . . . . . . . . . . . . . . . . . . . . . . . . . . . . . . . . . . . . . . . . . . . . . . . . . . . . . . . . . . . . .    
286	C C C C C C C C C . C C C C C C C C C C C C C C C C C C C C C . . C C C C C C C C C C C C C C C . C C C C C C . C C C C C C C C C C C C C C . C C C C C C C C . C . . . . . . . C C C C . . . . . . . C . C . . . . . . . . . . . . . . . . . . . . . . . . . . . . . . . . . . . . . . . . . . . . . . . . . . . . . . . . . . . . . . . . . . . . . . . . . . . . . . . . . . .    
287	A A A A A A A A A . A A A A A A A A A A A A A A A A A A A A A . . A A A A A A A A A A A A A A A . A A A A A A . A A A A A A A A A A A A A A . A A A A A A A A . A . . . . . . . A A A A . . . . . . . A . A . . . . . . . . . . . . . . . . . . . . . . . . . . . . . . . . . . . . . . . . . . . . . . . . . . . . . . . . . . . . . . . . . . . . . . . . . . . . . . . . . . .    
288	A A A A A A A A A . A A A A A A A A A A A A A A A A A A A A A . . A A A A A A A A A A A A A A A . A A A A A A . A A A A A A A A A A A A A A . A A A A A A A A . A . . . . . . . A A A A . . . . . . . A . A . . . . . . . . . . . . . . . . . . . . . . . . . . . . . . . . . . . . . . . . . . . . . . . . . . . . . . . . . . . . . . . . . . . . . . . . . . . . . . . . . . .    
289	A A A A A A A A A . A A A A A A A A A A A A A A A A A A A A A . . A A A A A A A A A A A A A A A . A A A A A A . A A A A A A A A A A A A A A . A A A A A A A A . A . . . . . . . A A A A . . . . . . . A . A . . . . . . . . . . . . . . . . . . . . . . . . . . . . . . . . . . . . . . . . . . . . . . . . . . . . . . . . . . . . . . . . . . . . . . . . . . . . . . . . . . .    
290	C C C C C C C C C . C C C C C C C C C C C C C C C C C C C C C . . C C C C C C C C C C C C C C C . C C C C C C . C C C C C C C C C C C C C C . C C C C C C C C . C . . . . . . . C C C C . . . . . . . C . C . . . . . . . . . . . . . . . . . . . . . . . . . . . . . . . . . . . . . . . . . . . . . . . . . . . . . . . . . . . . . . . . . . . . . . . . . . . . . . . . . . .    
291	C C C C C C C C C . C C C C C C C C C C C C C C C C C C C C C . . C C C C C C C C C C C C C C C . C C C C C C . C C C C C C C C C C C C C C . C C C C C C C C . C . . . . . . . C C C C . . . . . . . C . C . . . . . . . . . . . . . . . . . . . . . . . . . . . . . . . . . . . . . . . . . . . . . . . . . . . . . . . . . . . . . . . . . . . . . . . . . . . . . . . . . . .    
292	G G G G G G G G G . G G G G G G G G G G G G G G G G G G G G G . . G G G G G G G G G G G G G G G . G G G G G G . G G G G G G G G G G G G G G . G G G G G G G G . G . . . . . . . G G G G . . . . . . . G . G . . . . . . . . . . . . . . . . . . . . . . . . . . . . . . . . . . . . . . . . . . . . . . . . . . . . . . . . . . . . . . . . . . . . . . . . . . . . . . . . . . .    
293	C C C C C C C C C . C C C C C C C C C C C C C C C C C C C C C . . C C C C C C C C C C C C C C C . C C C C C C . C C C C C C C C C C C C C C . C C C C C C C C . C . . . . . . . C C C C . . . . . . . C . C . . . . . . . . . . . . . . . . . . . . . . . . . . . . . . . . . . . . . . . . . . . . . . . . . . . . . . . . . . . . . . . . . . . . . . . . . . . . . . . . . . .    
294	A A A A A A A A A . A A A A A A A A A A A A A A A A A A A A A . . A A A A A A A A A A A A A A A . A A A A A A . A A A A A A A A A A A A A A . A A A A A A A A . A . . . . . . . A A A A . . . . . . . A . A . . . . . . . . . . . . . . . . . . . . . . . . . . . . . . . . . . . . . . . . . . . . . . . . . . . . . . . . . . . . . . . . . . . . . . . . . . . . . . . . . . .    
295	A A A A A A A A A . A A A A A A A A A A A A A A A A A A A A A . . A A A A A A A A A A A A A A A . A A A A A A . A A A A A A A A A A A A A A . A A A A A A A A . A . . . . . . . A A A A . . . . . . . A . A . . . . . . . . . . . . . . . . . . . . . . . . . . . . . . . . . . . . . . . . . . . . . . . . . . . . . . . . . . . . . . . . . . . . . . . . . . . . . . . . . . .    
296	T T T T T T T T T . T T T T T T T T T T T T T T T T T T T T T . . T T T T T T T T T T T T T T T . T T T T T T . T T T T T T T T T T T T T T . T T T T T T T T . T . . . . . . . T T T T . . . . . . . T . T . . . . . . . . . . . . . . . . . . . . . . . . . . . . . . . . . . . . . . . . . . . . . . . . . . . . . . . . . . . . . . . . . . . . . . . . . . . . . . . . . . .    
297	T T T T T T T T T . T T T T T T T T T T T T T T T T T T T T T . . T T T T T T T T T T T T T T T . T T T T T T . T T T T T T T T T T T T T T . T T T T T T T T . T . . . . . . . T T T T . . . . . . . T . T . . . . . . . . . . . . . . . . . . . . . . . . . . . . . . . . . . . . . . . . . . . . . . . . . . . . . . . . . . . . . . . . . . . . . . . . . . . . . . . . . . .    
298	C C C C C C C C C . C C C C C C C C C C C C C C C C C C C C C . . C C C C C C C C C C C C C C C . C C C C C C . C C C C C C C C C C C C C C . C C C C C C C C . C . . . . . . . C C C C . . . . . . . C . C . . . . . . . . . . . . . . . . . . . . . . . . . . . . . . . . . . . . . . . . . . . . . . . . . . . . . . . . . . . . . . . . . . . . . . . . . . . . . . . . . . .    
299	A A A A A A A A A . A A A A A A A A A A A A A A A A A A A A A . . A A A A A A A A A A A A A A A . A A A A A A . A A A A A A A A A A A A A A . A A A A A A A A . A . . . . . . . A A A A . . . . . . . A . A . . . . . . . . . . . . . . . . . . . . . . . . . . . . . . . . . . . . . . . . . . . . . . . . . . . . . . . . . . . . . . . . . . . . . . . . . . . . . . . . . . .    
300	T T T T T T T T T . T T T T T T T T T T T T T T T T T T T T T . . T T T T T T T T T T T T T T T . T T T T T T . T T T T T T T T T T T T T T . T T T T T T T T . T . . . . . . . T T T T . . . . . . . T . T . . . . . . . . . . . . . . . . . . . . . . . . . . . . . . . . . . . . . . . . . . . . . . . . . . . . . . . . . . . . . . . . . . . . . . . . . . . . . . . . . . .    
301	G G G G G G G G G . G G G G G G G G G G G G G G G G G G G G G . . G G G G G G G G G G G G G G G . G G G G G G . G G G G G G G G G G G G G G . G G G G G G G G . G . . . . . . . G G G G . . . . . . . G . G . . . . . . . . . . . . . . . . . . . . . . . . . . . . . . . . . . . . . . . . . . . . . . . . . . . . . . . . . . . . . . . . . . . . . . . . . . . . . . . . . . .    
302	T T T T T T T T T . T T T T T T T T T T T T T T T T T T T T T . . T T T T T T T T T T T T T T T . T T T T T T . T T T T T T T T T T T T T T . T T T T T T T T . T . . . . . . . T T T T . . . . . . . T . T . . . . . . . . . . . . . . . . . . . . . . . . . . . . . . . . . . . . . . . . . . . . . . . . . . . . . . . . . . . . . . . . . . . . . . . . . . . . . . . . . . .    
303	A A A A A A A A A . A A A A A A A A A A A A A A A A A A A A A . . A A A A A A A A A A A A A A A . A A A A A A . A A A A A A A A A A A A A A . A A A A A A A A . A . . . . . . . A A A A . . . . . . . A . A . . . . . . . . . . . . . . . . . . . . . . . . . . . . . . . . . . . . . . . . . . . . . . . . . . . . . . . . . . . . . . . . . . . . . . . . . . . . . . . . . . .    
304	G G G T G G G G G . G G G G G G G G G G G G G G G G G G G G G . . G G G G G G G G G G G G G G G . G G G G G G . G G G G G G G G G G G G G G . G G G G G G G G . G . . . . . . . G G G G . . . . . . . G . G . . . . . . . . . . . . . . . . . . . . . . . . . . . . . . . . . . . . . . . . . . . . . . . . . . . . . . . . . . . . . . . . . . . . . . . . . . . . . . . . . . .    
305	G G G G G G G G G . G G G G G G G G G G G G G G G G G G G G G . . G G G G G G G G G G G G G G G . G G G G G G . G G G G G G G G G G G G G G . G G G G G G G G . G . . . . . . . G G G G . . . . . . . G . G . . . . . . . . . . . . . . . . . . . . . . . . . . . . . . . . . . . . . . . . . . . . . . . . . . . . . . . . . . . . . . . . . . . . . . . . . . . . . . . . . . .    
306	A A A A A A A A A . A A A A A A A A A A A A A A A A A A A A A . . A A A A A A A A A A A A A A A . A A A A A A . A A A A A A A A A A A A A A . A A A A A A A A . A . . . . . . . A A A A . . . . . . . A . A . . . . . . . . . . . . . . . . . . . . . . . . . . . . . . . . . . . . . . . . . . . . . . . . . . . . . . . . . . . . . . . . . . . . . . . . . . . . . . . . . . .    
307	A A A A A A A A A . A A A A A A A A A A A A A A A A A A A A A . . A A A A A A A A A A A A A A A . A A A A A A . A A A A A A A A A A A A A A . A A A A A A A A . A . . . . . . . A A A A . . . . . . . A . A . . . . . . . . . . . . . . . . . . . . . . . . . . . . . . . . . . . . . . . . . . . . . . . . . . . . . . . . . . . . . . . . . . . . . . . . . . . . . . . . . . .    
308	C C C C C C C C C . C C C C C C C C C C C C C C C C C C C C C . . C C C C C C C C C C C C C C C . C C C C C C C C C C C C C C C C C C C C C . C C C C C C C C . C . . . . . . . C C C C . . . . . . . C . C . . . . . . . . . . . . . . . . . . . . . . . . . . . . . . . . . . . . . . . . . . . . . . . . . . . . . . . . . . . . . . . . . . . . . . . . . . . . . . . . . . .    
309	T T T T T T T T T . T T T T T T T T T T T T T T T T T T T T T . . T T T T T T T T T T T T T T T . T T T T T T T T T T T T T T T T T T T T T . T T T T T T T T . T . . . . . . . T T T T . . . . . . . T . T . . . . . . . . . . . . . . . . . . . . . . . . . . . . . . . . . . . . . . . . . . . . . . . . . . . . . . . . . . . . . . . . . . . . . . . . . . . . . . . . . . .    
310	A A A A A A A A A . A A A A A A A A A A A A A A A A A A A A A . . A A A A A A A A A A A A A A A . A A A A A A A A A A A A A A A A A A A A A . A A A A A A A A . A . . . . . . . A A A A . . . . . . . A . A . . . . . . . . . . . . . . . . . . . . . . . . . . . . . . . . . . . . . . . . . . . . . . . . . . . . . . . . . . . . . . . . . . . . . . . . . . . . . . . . . . .    
311	C C C C C C C C C . C C C C C C C C C C C C C C C C C C C C C . . C C C C C C C C C C C C C C C . C C C C C C C C C C C C C C C C C C C C C . C C C C C C C C . C . . . . . . . C C C C . . . . . . . C . C . . . . . . . . . . . . . . . . . . . . . . . . . . . . . . . . . . . . . . . . . . . . . . . . . . . . . . . . . . . . . . . . . . . . . . . . . . . . . . . . . . .    
312	A A A A A A A A A . A A A A A A A A A A A A A A A A A A A A A . . A A A A A A A A A A A A A A A . A A A A A A A A A A A A A A A A A A A A A . A A A A A A A A . A . . . . . . . A A A A . . . . . . A A . A . . . . . . . . . . . . . . . . . . . . . . . . . . . . . . . . . . . . . . . . . . . . . . . . . . . . . . . . . . . . . . . . . . . . . . . . . . . . . . . . . . .    
313	G G G G G G G G G . G G G G G G G G G G G G G G G G G G G G G . . G G G G G G G G G G G G G G G . G G G G G G G G G G G G G G G G G G G G G . G G G G G G G G . G . . . . . . . G G G G . . . . . . G G . G . . . . . . . . . . . . . . . . . . . . . . . . . . . . . . . . . . . . . . . . . . . . . . . . . . . . . . . . . . . . . . . . . . . . . . . . . . . . . . . . . . .    
314	C C C C C C C C C . C C C C C C C C C C C C C C C C C C C C C . . C C C C C C C C C C C C C C C . C C C C C C C C C C C C C C C C C C C C C . C C C C C C C C . C . . . . . . . C C C C . . . . . . C C . C . . . . . . . . . . . . . . . . . . . . . . . . . . . . . . . . . . . . . . . . . . . . . . . . . . . . . . . . . . . . . . . . . . . . . . . . . . . . . . . . . . .    
315	A A A A A A A A A . A A A A A A A A A A A A A A A A A A A A A . . A A A A A A A A A A A A A A A . A A A A A A A A A A A A A A A A A A A A A . A A A A A A A A . A . . . . . . . A A A A . . . . . . A A . A . . . . . . . . . . . . . . . . . . . . . . . . . . . . . . . . . . . . . . . . . . . . . . . . . . . . . . . . . . . . . . . . . . . . . . . . . . . . . . . . . . .    
316	G G G G G G G G G . G G G G G G G G G G G G G G G G G G G G G . . G G G G G G G G G G G G G G G . G G G G G G G G G G G G G G G G G G G G G . G G G G G G G G . G . . . . . . . G G G G . . . . . . G G . G . . . . . . . . . . . . . . . . . . . . . . . . . . . . . . . . . . . . . . . . . . . . . . . . . . . . . . . . . . . . . . . . . . . . . . . . . . . . . . . . . . .    
317	A A A A A A A A A . A A A A A A A A A A A A A A A A A A A A A . . A A A A A A A A A A A A A A A . A A A A A A A A A A A A A A A A A A A A A . A A A A A A A A . A . . . . . . . A A A A . . . . . . A A . A . . . . . . . . . . . . . . . . . . . . . . . . . . . . . . . . . . . . . . . . . . . . . . . . . . . . . . . . . . . . . . . . . . . . . . . . . . . . . . . . . . .    
318	A A A A A A A A A . A T A A A A T A A A T A A A A A A A A A A . . A T A A A A A A A A A A A A A . A A A A A A A A A A A A A A A A A A A A A . A A A A A A A A . A . . . . . . . A A A A . . . . . . A A . A . . . . . . . . . . . . . . . . . . . . . . . . . . . . . . . . . . . . . . . . . . . . . . . . . . . . . . . . . . . . . . . . . . . . . . . . . . . . . . . . . . .    
319	G G G G G G G G G . G G G G G G G G G G G G G G G G G G G G G . . G G G G G G G G G G G G G G G . G G G G G G G G G G G G G G G G G G G G G . G G G G G G G G . G . . . . . . . G G G G . . . . . . G G . G . . . . . . . . . . . . . . . . . . . . . . . . . . . . . . . . . . . . . . . . . . . . . . . . . . . . . . . . . . . . . . . . . . . . . . . . . . . . . . . . . . .    
320	G G G G G G G G G . G G G G G G G G G G G G G G G G G G G G G . . G G G G G G G G G G G G G G G . G G G G G G G G G G G G G G G G G G G G G . G G G G G G G G . G . . . . . . . G G G G . . . . . . G G . G . . . . . . . . . . . . . . . . . . . . . . . . . . . . . . . . . . . . . . . . . . . . . . . . . . . . . . . . . . . . . . . . . . . . . . . . . . . . . . . . . . .    
321	G G G G G G G G G . G G G G G G G G G G G G G G G G G G G G G . . G G G G G G G G G G G G G G G . G G G G G G G G G G G G G G G G G G G G G . G G G G G G G G . G . . . . . . . G G G G . . . . . . G G . G . . . . . . . . . . . . . . . . . . . . . . . . . . . . . . . . . . . . . . . . . . . . . . . . . . . . . . . . . . . . . . . . . . . . . . . . . . . . . . . . . . .    
322	T T T T T T T T T . T T T T T T T T T T T T T T T T T T T T T . . T T T T T T T T T T T T T T T . T T T T T T T T T T T T T T T T T T T T T . T T T T T T T T . T . . . . . . . T T T T . . . . . . T T . T . . . . . . . . . . . . . . . . . . . . . . . . . . . . . . . . . . . . . . . . . . . . . . . . . . . . . . . . . . . . . . . . . . . . . . . . . . . . . . . . . . .    
323	C C C C C C C C C . C C C C C C C C C C C C C C C C C C C C C . . C C C C C C C C C C C C C C C C C C C C C C C C C C C C C C C C C C C C C . C C C C C C C C . C . . . . . . . C C C C . . . . . . C C . C . . . . . . . . . . . . . . . . . . . . . . . . . . . . . . . . . . . . . . . . . . . . . . . . . . . . . . . . . . . . . . . . . . . . . . . . . . . . . . . . . . .    
324	C C C C C C C C C . C C C C C C C C C C C C C C C C C C C C C . . C C C C C C C C C C C C C C C C C C C C C C C C C C C C C C C C C C C C C . C C C C C C C C . C . . . . . . . C C C C . . . . . . C C . C . . . . . . . . . . . . . . . . . . . . . . . . . . . . . . . . . . . . . . . . . . . . . . . . . . . . . . . . . . . . . . . . . . . . . . . . . . . . . . . . . . .    
325	A A A A A A A A A . A A A A A A A A A A A A A A A A A A A A A . . A A A A A A A A A A A A A A A A A A A A A A A A A A A A A A A A A A A A A . A A A A A A A A . A . . . . . . . A A A A . . . . . . A A . A . . . . . . . . . . . . . . . . . . . . . . . . . . . . . . . . . . . . . . . . . . . . . . . . . . . . . . . . . . . . . . . . . . . . . . . . . . . . . . . . . . .    
326	A A A A A A A A A . A A A A A A A A A A A A A A A A A A A A A . . A A A A A A A A A A A A A A A A A A A A A A A A A A A A A A A A A A A A A . A A A A A A A A . A . . . . . . . A A A A . . . . . . A A . A . . . . . . . . . . . . . . . . . . . . . . . . . . . . . . . . . . . . . . . . . . . . . . . . . . . . . . . . . . . . . . . . . . . . . . . . . . . . . . . . . . .    
327	C C C C C C C C C . C C C C C C C C C C C C C C C C C C C C C . . C T C C C C C C C C C C C C C C C C C C C C C C C C C C C C C C C C C C C . C C C C C C C C . C . . . . . . . C C C C . . . . . . C C . C . . . . . . . . . . . . . . . . . . . . . . . . . . . . . . . . . . . . . . . . . . . . . . . . . . . . . . . . . . . . . . . . . . . . . . . . . . . . . . . . . . .    
328	A A A A A A A A A . A A A A A A A A A A A A A A A A A A A A A . . A A A A A A A A A A A A A A A A A A A A A A A A A A A A A A A A A A A A A . A A A A A A A A . A . . . . . . . A A A A . . . . . . A A . A . . . . . . . . . . . . . . . . . . . . . . . . . . . . . . . . . . . . . . . . . . . . . . . . . . . . . . . . . . . . . . . . . . . . . . . . . . . . . . . . . . .    
329	A A A A A A A A A . A A A A A A A A A A A A A A A A A A A A A . . A A A A A A A A A A A A A A A A A A A A A A A A A A A A A A A A A A A A A . A A A A A A A A . A . . . . . . . A A A A . . . . . . A A . A . . . . . . . . . . . . . . . . . . . . . . . . . . . . . . . . . . . . . . . . . . . . . . . . . . . . . . . . . . . . . . . . . . . . . . . . . . . . . . . . . . .    
330	G G G G G G G G G . G G G G G G G G G G G G G G G G G G G G G . . G G G G G G G G G G G G G G G G G G G G G G G G G G G G G G G G G G G G G . G G G G G G G G . G . . . . . . . G G G G . . . . . . G G . G . . . . . . . . . . . . . . . . . . . . . . . . . . . . . . . . . . . . . . . . . . . . . . . . . . . . . . . . . . . . . . . . . . . . . . . . . . . . . . . . . . .    
331	A A A A A A A A A . A A A A A A A A A A A A A A A A A A A A A . . A A A A A A A A A A A A A A A A A A A A A A A A A A A A A A A A A A A A A . A A A A A A A A . A . . . . . . . A A A A . . . . . . A A . A . . . . . . . . . . . . . . . . . . . . . . . . . . . . . . . . . . . . . . . . . . . . . . . . . . . . . . . . . . . . . . . . . . . . . . . . . . . . . . . . . . .    
332	G G G G G G G G G . G G G G G G G G G G G G G G G G G G A G G . . G G G G G G G G G G G G G G G G G G G G G G G G G G G G G G G G G G G G G . G G G G G G G G . G . . . . . . . G G G G . . . . . . G G . G . . . . . . . . . . . . . . . . . . . . . . . . . . . . . . . . . . . . . . . . . . . . . . . . . . . . . . . . . . . . . . . . . . . . . . . . . . . . . . . . . . .    
333	G G G G G G G G G . G G G G G G G G G G G G G G G G G G G G G . . G G G G G G G G G G G G G G G G G G G G G G G G G G G G G G G G G G G G G . G G G G G G G G . G . . . . . . . G G G G . . . . . . G G . G . . . . . . . . . . . . . . . . . . . . . . . . . . . . . . . . . . . . . . . . . . . . . . . . . . . . . . . . . . . . . . . . . . . . . . . . . . . . . . . . . . .    
334	G G G G G G G G G . G G G G G G G G G G G G G G G G G G G G G . . G G G G G G G G G G G G G G G G G G G G G G G G G G G G G G G G G G G G G . G G G G G G G G . G . . . . . . . G G G G . . . . . . G G . G . . . . . . . . . . . . . . . . . . . . . . . . . . . . . . . . . . . . . . . . . . . . . . . . . . . . . . . . . . . . . . . . . . . . . . . . . . . . . . . . . . .    
335	C C C C C C C C C . C C C C C C C C C C C C C C C C C C C C C . . C C C C C C C C C C C C C C C C C C C C C C C C C C C C C C C C C C C C C . C C C C C C C C . C . . . . . . . C C C C . . . . . . C C . C . . . . . . . . . . . . . . . . . . . . . . . . . . . . . . . . . . . . . . . . . . . . . . . . . . . . . . . . . . . . . . . . . . . . . . . . . . . . . . . . . . .    
336	T T T T T T T T T . T T C T T T T T T T T T T T T T T T T T T . . T T T T T T T T T T T T T T T T T T T T T T T T T T T T T T T T T T T T T . T T T T T T T T . T . . . . . . . T T T T . . . . . . T T . T . . . . . . . . . . . . . . . . . . . . . . . . . . . . . . . . . . . . . . . . . . . . . . . . . . . . . . . . . . . . . . . . . . . . . . . . . . . . . . . . . . .    
337	T T T T T T T T T . T T T T T T T T T T T T T T T T T T T T T . . T T T T T T T T T T T T T T T T T T T T T T T T T T T T T T T T T T T T T . T T T T T T T T . T . . . . . . . T T T T . . . . . . T T . T . . . . . . . . . . . . . . . . . . . . . . . . . . . . . . . . . . . . . . . . . . . . . . . . . . . . . . . . . . . . . . . . . . . . . . . . . . . . . . . . . . .    
338	T T T T T T T T T . T T T T T T T T T T T T T T T T T T T T T . . T T T T T T T T T T T T T T T T T T T T T T T T T T T T T T T T T T T T T . T T T T T T T T . T . . . . . . . T T T T . . . . . . T T . T . . . . . . . . . . . . . . . . . . . . . . . . . . . . . . . . . . . . . . . . . . . . . . . . . . . . . . . . . . . . . . . . . . . . . . . . . . . . . . . . . . .    
339	C C C C C C C C C . C C C C C C C C C C C C C C C C C C C C C . . C C C C C C C C C C C C C C C C C C C C C C C C C C C C C C C C C C C C C . C C C C C C C C . C . . . . . . . C C C C . . . . . . C C . C . . . . . . . . . . . . . . . . . . . . . . . . . . . . . . . . . . . . . . . . . . . . . . . . . . . . . . . . . . . . . . . . . . . . . . . . . . . . . . . . . . .    
340	A A A A A A A A A . A A A A A A A A A A A A A A A A A A A A A . . A A A A A A A A A A A A A A A A A A A A A A A A A A A A A A A A A A A A A . A A A A A A A A . A . . . . . . . A A A A . . . . . . A A . A . . . . . . . . . . . . . . . . . . . . . . . . . . . . . . . . . . . . . . . . . . . . . . . . . . . . . . . . . . . . . . . . . . . . . . . . . . . . . . . . . . .    
341	A A A A A A A A A . A A A A A A A A A A A A A A A A A A A A A . . A A A A A A A A A A A A A A A A A A A A A A A A A A A A A A A A A A A A A . A A A A A A A A . A . . . . . . . A A A A . . . . . . A A . A . . . . . . . . . . . . . . . . . . . . . . . . . . . . . . . . . . . . . . . . . . . . . . . . . . . . . . . . . . . . . . . . . . . . . . . . . . . . . . . . . . .    
342	G G G G G G G G G . G G G G G G G G G G G G G G G G G G G G G . . G G G G G G G G G G G G G G G G G G G G G G G G G G G G G G G G G G G G G . G G G G G G G G . G . . . . . . . G G G G . . . . . . G G . G . . . . . . . . . . . . . . . . . . . . . . . . . . . . . . . . . . . . . . . . . . . . . . . . . . . . . . . . . . . . . . . . . . . . . . . . . . . . . . . . . . .    
343	A A A A A A A A A . A A A A A A A A A A A A A A A A A A A A A . . A A A A A A A A A A A A A A A A A A A A A A A A A A A A A A A A A A A A A . A A A A A A A A . A . . . . . . . A A A A . . . . . A A A . A . . . . . . . . . . . . . . . . . . . . . . . . . . . . . . . . . . . . . . . . . . . . . . . . . . . . . . . . . . . . . . . . . . . . . . . . . . . . . . . . . . .    
344	T T T T T T T T T . T T T T T T T T T T T T T T T T T T T T T . . T T T T T T T T T T T T T T T T T T T T T T T T T T T T T T T T T T T T T . T T T T T T T T . T . . . . . . . T T T T . . . . . T T T . T . . . . . . . . . . . . . . . . . . . . . . . . . . . . . . . . . . . . . . . . . . . . . . . . . . . . . . . . . . . . . . . . . . . . . . . . . . . . . . . . . . .    
345	A A A A A A A A A . A A A A A A A A A A A A A A A A A A A A A . . A A A A A A A A A A A A A A A A A A A A A A A A A A A A A A A A A A A A A . A A A A A A A A . A . . . . . . . A A A A . . . . . A A A . A . . . . . . . . . . . . . . . . . . . . . . . . . . . . . . . . . . . . . . . . . . . . . . . . . . . . . . . . . . . . . . . . . . . . . . . . . . . . . . . . . . .    
346	G G G G G G G G G . G G G G G G G G G G G G G G G G G G G G G . . G G G G G G G G G G G G G G G G G G G G G G G G G G G G G G G G G G G G G . G G G G G G G G . G . . . . . . . G G G G . . . . . G G G . G . . . . . . . . . . . . . . . . . . . . . . . . . . . . . . . . . . . . . . . . . . . . . . . . . . . . . . . G . . . . . . . . . . . . . . . . . . . . . . . . . . .    
347	C C C C C C C C C . C C C C C C C C C C C C C C C C C C C C C . . C C C C C C C C C C C C C C C C C C C C C C C C C C C C C C C C C C C C C . C C C C C C C C . C . . . . . . . C C C C . . . . . C C C . C . . . . . . . . . . . . . . . . . . . . . . . . . . . . . . . . . . . . . . . . . . . . . . . . . . . . . . . C . . . . . . . . . . . . . . . . . . . . . . . . . . .    
348	T T T T T T T T T . T T T T T T T T T T T T T T T T T T T T T . . T T T T T T T T T T T T T T T T T T T T T T T T T T T T T T T T T T T T T . T T T T T T T T . T . . . . . . . T T T T . . . . . T T T . T . . . . . . . . . . . . . . . . . . . . . . . . . . . . . . . . . . . . . . . . . . . . . . . . . . . . . . . T . . . . . . . . . . . . . . . . . . . . . . . . . . .    
349	G G G G G G G G G . G G G G G G G G G G G G G G G G G G G G G . . G G G G G G G G G G G G G G G G G G G G G G G G G G G G G G G G G G G G G . G G G G G G G G . G . . . . . . . G G G G . . . . . G G G . G . . . . . . . . . . . . . . . . . . . . . . . . . . . . . . . . . . . . . . . . . . . . . . . . . . . . . . . G . . G . . . . . . . . . . . . . . . . . . . . . . . .    
350	G G G G G G G G G . G G G G G G G G G G G G G G G G G G G G G . . G G G G G G G G G G G G G G G G G G G G G G G G G G G G G G G G G G G G G . G G G G G G G G . G . . . . . . . G G G G . . . . . G G G . G . . . . . . . . . . . . . . . . . . . . . . . . . . . . . . . . . . . . . . . . . . . . . . . . . . . . . . . G . . G . . . . . . . . . . . . . . . . . . . . . . . .    
351	T T T T T T T T T . T T T T T T T T T T T T T T T T T T T T T . . T T T T T T T T T T T T T T T T T T T T T T T T T T T T T T T T T T T T T . T T T T T T T T . T . . . . . . . T T T T . . . . . T T T . T . . . . . . . . . . . . . . . . . . . . . . . . . . . . . . . . . . . . . . . . . . . . . . . . . . . . . . . T . . T . . . . . . . . . . . . . . . . . . . . . . . .    
352	T T T T T T T T T . T T T T T T T T T T T T T T T T T T T T T . . T T T T T T T T T T T T T T T T T T T T T T T T T T T T T T T T T T T T T . T T T T T T T T . T . . . . . . . T T T T . . . . . T T T . T . . . . . . . . . . . . . . . . . . . . . . . . . . . . . . . . . . . . . . . . . . . . . . . . . . . . . . . T . . T . . . . . . . . . . . . . . . . . . . . . . . .    
353	T T T T T T T T T . T T T T T T - T T T T T T T T T T T T T T . . T T T T T T T T T T T T T T T T T T T T T T T T T T T T T T T T T T T T T . T T T T T T T T . T . . . . . . . T T T T . . . . . T T T . T . . . . . . . . . . . . . . . . . . . . . . . . . . . . . . . . . . . . . . . . . . . . . . . . . . . . . . . T . . T . . . . . . . . . . . . . . . . . . . . . . . .    
354	C C C C C C C C C . C C C C C C C C C C C C C C C C C C C C C . . C C C C C C C C C C C C C C C C C C C C C C C C C C C C C C C C C C C C C . C C C C C C C C . C . . . . . . . C C C C . . . . . C C C . C . . . . . . . . . . . . . . . . . . . . . . . . . . . . . . . . . . . . . . . . . . . . . . . . . . . . . . . C . . C . . . . . . . . . . . . . . . . . . . . . . . .    
355	A A A A A A A A A . A A A A A A A A A A A A A A A A A A A A A . . A A A A A A A A A A A A A A A A A A A A A A A A A A A A A A A A A A A A A . A A A A A A A A . A . . . . . . . A A A A . . . . . A A A . A . . . . . . . . . . . . . . . . . . . . . . . . . . . . . . . . . . . . . . . . . . . . . . . . . . . . . . . A . . A . . . . . . . . . . . . A . . . . . . . . . . .    
356	C C C C C C C C C . C C C C C C C C C C C C C C C C C C C C C . . C C C C C C C C C C C C C C C C C C C C C C C C C C C C C C C C C C C C C . C C C C C C C C . C . . . . . . . C C C C . . . . C C C C . C . . . . . . . . . . . . . . . . . . . . . . . . . . . . . . . . . . . . . . . . . . . . . . . . . . . . . . . C . . C . . . . . . . . . . . . C . . . . . . . . . . .    
357	C C C C C C C C C . C C C C C C C C C C C C C C C C C C C C C . . C C C C C C C C C C C C C C C C C C C C C C C C C C C C C C C C C C C C C . C C C C C C C C . C . . . . . . . C C C C . . . . C C C C . C . . . . . . . . . . . . . . . . . . . . . . . . . . . . . . . . . . . . . . . . . . . . . . . . . . . . . . . C . . C . . . . . . . . . . . . C . . . . . . . . . . .    
358	C C C C C C C C C . C C C C C C C C C C C C C C C C C C C C C . . C C C C C C C C C C C C C C C C C C C C C C C C C C C C C C C C C C C C C . C C C C C C C C . C . . . . . . . C C C C . . . . C C C C . C . . . . . . . . . . . . . . . . . . . . . . . . . . . . . . . . . . . . . . . . . . . . . . . . . . . . . . . C . . C . . . . . . . . . . . . C . . . . . . . . . . .    
359	T T T T T T T T T . T T T T T T T T T T T T T T T T T T T T T . . T T T T T T T T T T T T T T T T T T T T T T T T T T T T T T T T T T T T T . T T T T T T T T . T . . . . . . . T T T T . . . . T T T T T T . . . . . . . . . . . . . . . . . . . . . . . . . . . . . . . . . . . . . . . . . . . . . . . . . . . . . . . T . . T . . . . . . . . . . . . T . . . . . . . . . . .    
360	G G G G G G G G G . G G G G G G G G G G G G G G G G G G G G G . . G G G G G G G G G G G G G G G G G G G G G G G G G G G G G G G G G G G G G . G G G G G G G G . G . . . . . . . G G G G . . . . G G G G G G . . . . . . . . . . . . . . . . . . . . . . . . . . . . . . . . . . . . . . . . . . . . . . . . . . . . . . . G . . G . . . . . . . . . . . . G . . . . . . . . . . .    
361	T T T T T T T T T . T T T T T T T T T T T T T T T T T T T T T . . T T T T T T T T T T T T T T T T T T T T T T T T T T T T T T T T T T T T T . T T T T T T T T . T . . . . . . . T T T T . . . . T T T T T T . . . . . . . . . . . . . . . . . . . . . . . . . . . . . . . . . . . . . . . . . . . . . . . . . . . . . . . T . . T . . . . . . . . . . . . T . . . . . . . . . . .    
362	T T T T T T T T T T T T T T T T T T T T T T T T T T T T T T T . . T T T T T T T T T T T T T T T T T T T T T T T T T T T T T T T T T T T T T . T T T T T T T T . T . . . . . . . T T T T . . . . T T T T T T . . . . . . . . . . . . . . . . . . . . . . . . . . . . . . . . . . . . . . . . . . . . . . . . . . . . . . . T . . T . . . . . . . . . . . . T . . . . . . . . . . .    
363	G G G G G G G G G G G G G G G G G G G G G G G G G G G G G G G . . G G G G G G G G G G G G G G G G G G G G G G G G G G G G G G G G G G G G G . G G G G G G G G . G . . . . . . . G G G G . . . . G G G G G G . . . . . . . . . . . . . . . . . . . . . . . . . . . . . . . . . . . . . . . . . . . . . . . . . . . . . . . G . . G . . . . . . . . . . . . G . . . . . . . . . . .    
364	G G G G G G G G G G G G G G G G G G G G G G G G G G G G G G G . . G G G G G G G G G G G G G G G G G G G G G G G G G G G G G G G G G G G G G . G G G G G G G G . G . . . . . . . G G G G . . . . G G G G G G . . . . . . . . . . . . . . . . . . . . . . . . . . . . . . . . . . . . . . . . . . . . . . . . . . . . . . . G . . G . . . . . . . . . . . . G . . . . . . . . . . .    
365	C C C C C C C C C C C C C C C C C C C C C C C C C C C C C C C . . C C C C C C C C C C C C C C C C C C C C C C C C C C C C C C C C C C C C C . C C C C C C C C . C . . . . . . . C C C C . . . . C C C C C C . . . . . . . . . . . . . . . . . . . . . . . . . . . . . . . . . . . . . . . . . . . . . . . . . . . . . . . C . . C . . . . . . . . . . . . C . . . . . . . . . . .    
366	C C C C C C C C C C C C C C C C C C C C C C C C C C C C C C C . . C C C C C C C C C C C C C C C C C C C C C C C C C C C C C C C C C C C C C . C C C C C C C C . C . . . . . . . C C C C . . . . C C C C C C . . . . . . . . . . . . . . . . . . . . . . . . . . . . . . . . . . . . . . . . . . . . . . . . . . . . . . . C . . C . . . . . . . . . . . . C . . . . . . . . . . .    
367	T T T T T T T T T T T T T T T T T T T T T T T T T T T T T T T . . T T T T T T T T T T T T T T T T T T T T T T T T T T T T T T T T T T T T T . T T T T T T T T . T . . . . . . . T T T T . . . . T T T T T T . . . . . . . . . . . . . . . . . . . . . . . . . . . . . . . . . . . . . . . . . . . . . . . . . . . . . . . T . . T . . . . . . . . . . . . T . . . . . . . . . . .    
368	G G G G G G G G G G G G G G G G G G G G G G G G G G G G G G G . . G G G G G G G A G G G G G G G G G G G G G G G G G G G G G G G G G G G G G . G G G G G G G G . G . . . . . . . G G G G . . G . G G G G G G . . . . . . . . . . . . . . . . . . . . . . . . . . . . . . . . . . . . . . . . . . . . . . . . . . . . . . . G . . G . . . . . . . . . . . . G . . . . . . . . . . .    
369	C C C C C C C C C C C T C C C C C C C C C C C C C C C C C C C . . C C C C C C C C C C C C C C C C C C C C C C C C C C C C C C C C C C C C C . C C C C C C C C . C . . . . . . . C C C C . . C . C C C C C C . . . . . . . . . . . . . . . . . . . . . . . . . . . . . . . . . . . . . . . . . . . . . . . . . . . . . . . C . . C . . . . . . . . . . . . C . . . . . . . . . . .    
370	C C C C C C C C C C C C C C C C C C C C C C C C C C C C C C C . . C C C C C C C C C C C C C C C C C C C C C C C C C C C C C C C C C C C C C . C C C C C C C C C C . . . . . . . C C C C . . C . C C C C C C . . . . . . . . . . . . . . . . . . . . . . . . . . . . . . . . . . . . . . . . . . . . . . . . . . . . . . . C . . C . . . . . . . . . . . . C . . . . . . . . . . .    
371	T T T T T T T T T T T T T T T T T T T T T T T T T T T T T T T . . T T T T T T T T T T T T T T T T T T T T T T T T T T T T T T T T T T T T T . T T T T T T T T T T . . . . . . . T T T T . . T . T T T T T T . . . . . . . . . . . . . . . . . . . . . . . . . . . . . . . . . . . . . . . . . . . . . . . . . . . . . . . T . . T . . . . . . . . . . . . T . . . . . . . . . . .    
372	G G G G G G G G G G G G G G G G G G G G G G G G G G G G G G G . . G G G G G G G G G G G G G G G G G G G G G G G G G G G G G G G G G G G G G . G G G G G G G G G G . . . . . . . G G G G . . G . G G G G G G . . . . . . . . . . . . . . . . . . . . . . . . . . . . . . . . . . . . . . . . . . . . . . . . . . . . . . . G . . G . . . . . . . . . . . . G . . . . . . . . . . .    
373	C C C C C C C C C C C T C C C C C C C C C C C C C C C C C C C . . C C C C C C C C C C C C C C C C C C C C C C C C C C C C C C C C C C C C C . C C C C C C C C C C . . . . . . . C C C C . . C . C C C C C C . . . . . . . . . . . . . . . . . . . . . . . . . . . . . . . . . . . . . . . . . . . . . . . . . . . . . . . C . . C . . . . . . . . . . . . C . . . . . . . . . . .    
374	T T T T T T T T T T T T T T T T T T T T T T T T T T T T T T T . . T T T T T T T T T T T T T T T T T T T T T T T T T T T T T T T T T T T T T . T T T T T T T T T T . . . . . . . T T T T . . T . T T T T T T . . . . . . . . . . . . . . . . . . . . . . . . . . . . . . . . . . . . . . . . . . . . . . . . . . . . . . . T . . T . . . . . . . . . . . . T . . . . . . . . . . .    
375	C C C C C C C C C C C C C C C C C C C C C C C C C C C C C C C . . C C C C C C C C C C C C C C C C C C C C C C C C C C C C C C C C C C C C C . C C C C C C C C C C . . . . . . . C C C C . . C . C C C C C C . . . . . . . . . . . . . . . . . . . . . . . . . . . . . . . . . . . . . . . . . . . . . . . . . . . . . . . C . . C . . . . . . . . . . . . C . . . . . . . . . . .    
376	A A A A A A A A A A A A A A A A A A A A A A A A A A A A A A A . . A A A A A A A A A A A A A A A A A A A A A A A A A A A A A A A A A A A A A . A A A A A A A A A A . . . . . . . A A A A . . A . A A A A A A . . . . . . . . . . . . . . . . . . . . . . . . . . . . . . . . . . . . . . . . . . . . . . . . . . . . . . . A . . A . . . . . . . . . . . . A . . . . . . . . . . .    
377	T T T T T T T T T T T T T T T T T T T T T T T T T T T T T T T . . T T T T T T T T T T T T T T T T T T T T T T T T T T T T T T T T T T T T T . T T T T T T T T T T . . . . . . . T T T T . . T . T T T T T T . . . . . . . . . . . . . . . . . . . . . . . . . . . . . . . . . . . . . . . . . . . . . . . . . . . . . . . T . . T . . . . . . . . . . . . T . . . . . . . . . . .    
378	T T T T T T T T T T T T T T T T T T T T T T T T T T T T T T T . . T T T T T T T T T T T T T T T T T T T T T T T T T T T T T T T T T T T T T . T T T T T T T T T T . . . . . . . T T T T . . T . T T T T T T . . . . . . . . . . . . . . . . . . . . . . . . . . . . . . . . . . . . . . . . . . . . . . . . . . . . . . . T . . T . . . . . . . . . . . . T . . . . . . . . . . .    
379	G G G G G G G G G G G G G G G G G G G G G G G G G G G G G G G . . G G G G G G G G G G G G G G G G G G G G G G G G G G G G G G G G G G G G G . G G G G G G G G G G . . . . . . . G G G G . . G . G G G G G G . . . . . . . . . . . . . . . . . . . . . . . . . . . . . . . . . . . . . . . . . . . . . . . . . . . . . . . G . . G . . . . . . . . . . . . G . . . . . . . . . . .    
380	C C C C C C C C C C C C C C C C C C C C C C C C C C C C C C C . . C C C C C C C C C C C C C C C C C C C C C C C C C C C C C C C C C C C C C . C C C C C C C C C C . . . . . . . C C C C . . C . C C C C C C . . . . . . . . . . . . . . . . . . . . . . . . . . . . . . . . . . . . . . . . . . . . . . . . . . . . . . . C . . C . . . . . . . . . . . . C . . . . . . . . . . .    
381	T T T T T T T T T T T T T T T T T T T T T T T T T T T T T T T . . T T T T T T T T T T T T T T T T T T T T T T T T T T T T T T T T T T T T T . T T T T T T T T T T . . . . . . . T T T T . . T . T T T T T T . . . . . . . . . . . . . . . . . . . . . . . . . . . . . . . . . . . . . . . . . . . . . . . . . . . . . . . T . . T . . . . . . . . . . . . T . . . . . . . . . . .    
382	G G G G G G G G G G G G G G G G G G G G G G G G G G G G G G G . . G G G G G G G G G G G G G G G G G G G G G G G G G G G G G G G G G G G G G . G G G G G G G G G G . . . . . . . G G G G . . G . G G G G G G . . . . . . . . . . . . . . . . . . . . . . . . . . . . . . . . . . . . . . . . . . . . . . . . . . . . . . . G . . G . . . . . . . . . . . . G . . . . . . . . . . .    
383	G G G G G G G G G G G G G G G G G G G G G G G G G G G G G G G . . G G G G G G G G G G G G G G G G G G G G G G G G G G G G G G G G G G G G G . G G G G G G G G G G . . . . . . . G G G G . . G . G G G G G G . . . . . . . . . . . . . . . . . . . . . . . . . . . . . . . . . . . . . . . . . . . . . . . . . . . . . . . G . . G . . . . . . . . . . . . G . . . . . . . . . . .    
384	T T T T T T T T T T T T T T T T T T T T T T T T T T T T T T T . . T T T T T T T T T T T T T T T T T T T T T T T T T T T T T T T T T T T T T . T T T T T T T T T T . . . . . . . T T T T . . T . T T T T T T . . . . . . . . . . . . . . . . . . . . . . . . . . . . . . . . . . . . . . . . . . . . . . . . . . . . . . . T . . T . . . . . . . . . . . . T . . . . . . . . . . .    
385	C C C C C C C C C C C C C C C C C C C C C C C C C C C C C C C . . C C C C C C C C C C C C C C C C C C C C C C C C C C C C C C C C C C C C C . C C C C C C C C C C . . . . . . . C C C C . . C . C C C C C C . . . . . . . . . . . . . . . . . . . . . . . . . . . . . . . . . . . . . . . . . . . . . . . . . . . . . . . C . C C . . . . . . . . . . . . C . . . . . . . . . . .    
386	A A A A A A A A A A A A A A A A A A A A A A A A A A A A A A A . . A A A A A A A A A A A A A A A A A A A A A A A A A A A A A A A A A A A A A . A A A A A A A A A A . . . . . . . A A A A . . A . A A A A A A . . . . . . . . . . . . . . . . . . . . . . . . . . . . . . . . . . . . . . . . . . . . . . . . . . . . . . . A . A A . . . . . . . . . . . . A . . . . . . . . . . .    
387	G G G G G G G G G G G G G G G G G G G G G G G G G G G G G G G . . G G G G G G G G G G G G G G G G G G G G G G G G G G G G G G G G G G G G G . G G G G G G G G G G . . . . . . . G G G G . . G . G G G G G G . . . . . . . . . . . . . . . . . . . . . . . . . . . . . . . . . . . . . . . . . . . . . . . . . . . . . . . G . G G . . . . . . . . . . . . G . . . . . . . . . . .    
388	G G G G G G G G G G G G G G G G G G G G G G G G G G G G G G G . . G G G G G G G G G G G G G G G G G G G G G G G G G G G G G G G G G G G G G . G G G G G G G G G G . . . . . . . G G G G . . G . G G G G G G . . . . . . . . . . . . . . . . . . . . . . . . . . . . . . . . . . . . . . . . . . . . . . . . . . . . . . . G . G G . . . . . . . . . . . . G . . . . . . . . . . .    
389	C C C C C C C C C C C C C C C C C C C C C C C C C C C C C C C . . C C C C C C C C C C C C C C C C C C C C C C C C C C C C C C C C C C C C C . C C C C C C C C C C . . . . . . . C C C C . . C . C C C C C C . . . . . . . . . . . . . . . . . . . . . . . . . . . . . . . . . . . . . . . . . . . . . . . . . . . . . . . C . C C . . . . . . . . . . . . C . . . . . . . . . . .    
390	T T T T T T T T T T T T T T T T T T T T T T T T T T T T T T T . . T T T T T T T T T T T T T T T T T T T T T T T T T T T T T T T T T T T T T . T T T T T T T T T T . . . . . . . T T T T . . T . T T T T T T . . . . . . . . . . . . . . . . . . . . . . . . . . . . . . . . . . . . . . . . . . . . . . . . . . . . . . . T . T T . . . . . . T . . . . . T . . . . . . . . . . .    
391	C C C C C C C C C C C C C C C C C C C C C C C C C C C C C C C . . C C C C C C C C C C C C C C C C C C C C C C C C C C C C C C C C C C C C C . C C C C C C C C C C . . . . . . . C C C C . . C . C C C C C C . . . . . . . . . . . . . . . . . . . . . . . . . . . . . . . . . . . . . . . . . . . . . . . . . . . . . . . C . C C . . . . . . C . . . . . C . . . . . . . . . . .    
392	T T T T T T T T T T T T T T T T T T T T T T T T T T T T T T T . . T T T T T T T T T T T T T T T T T T T T T T T T T T T T T T T T T T T T T . T T T T T T T T T T . . . . . . . T T T T . . T . T T T T T T . . . . . . . . . . . . . . . . . . . . . . . . . . . . . . . . . . . . . . . . . . . . . . . . . . . . . . . G . T T . . . . . . T . . . . . T . . . . . . . . . . .    
393	G G G G G G G G G G G G G G G G G G G G G G G G G G G G G G G . . G G G G G G G G G G G G G G G G G G G G G G G G G G G G G G G G G G G G G . G G G G G G G G G G . . . . . . . G G G G . . G . G G G G G G . . . . . . . . . . . . . . . . . . . . . . . . . . . . . . . . . . . . . . . . . . . . . . . . . . . . . . . G . G G . . . . . . G . . . . . G . . . . . . . . . . .    
394	A A A A A A A A A A A A A A A A A A A A A A A A A A A A A A A . . A A A A A A A A A A A A A A A A A A A A A A A A A A A A A A A A A A A A A . A A A A A A A A A A . . . . . . . A A A A . . A . A A A A A A . . . . . . . . . . . . . . . . . . . . . . . . . . . . . . . . . . . . . . . . . . . . . . . . . . . . . . . A . A A . . . . . . A . . . . . A . . . . . . . . . . .    
395	C C C C C C C C C C C C C C C C C C C C C C C C C C C C C C C . . C C C C C C C C C C C C C C C C C C C C C C C C C C C C C C C C C C C C C . C C C C C C C C C C . . . . . . . C C C C . . C . C C C C C C . . . . . . . . . . . . . . . . . . . . . . . . . . . . . . . . . . . . . . . . . . . . . . . . . . . . . . . C . C C . . . . . . C . . . . . C . . . . . . . . . . .    
396	G G G G G G G G G G G G G G G G G G G G G G G G G G G G G G G . . G G G G G G G G G G G G G G G G G G G G G G G G G G G G G G G G G G G G G . G G G G G G G G G G . . . . . . . G G G G . . G . G G G G G G . . . . . . . . . . . . . . . . . . . . . . . . . . . . . . . . . . . . . . . . . . . . . . . . . . . . . . . G . G G . . . . . . G . . . . . G . . . . . . . . . . .    
397	G G G G G G G G G G G G G G G G G G G G G G G G G G G G G G G . . G G G G G G G G G G G G G G G G G G G G G G G G G G G G G G G G G G G G G . G G G G G G G G G G . . . . . . . G G G G . . G . G G G G G G . . . . . . . . . . . . . . . . . . . . . . . . . . . . . . . . . . . . . . . . . . . . . . . . . . . . . . . G . G G . . . . . . G . . . . . G . . . . . . . . . . .    
398	C C C C C C C C C C C C C C C C C C C C C C C C C C C C C C C . . C C C C C C C C C C C C C C C C C C C C C C C C C C C C C C C C C C C C C . C C C C C C C C C C . . . . . . . C C C C . . C . C C C C C C . . . . . . . . . . . . . . . . . . . . . . . . . . . . . . . . . . . . . . . . . . . . . . . . . . . . . . . C . C C . . . . . . C . . . . . C . . . . . . . . . . .    
399	C C C C C C C C C C C C C C C C C C C C C C C C C C C C C C C . . C C C C C C C C C C C C C C C C C C C C C C C C C C C C C C C C C C C C C . C C C C C C C C C C . . . . . . . C C C C . . C . C C C C C C . . . . . . . . . . . . . . . . . . . . . . . . . . . . . . . . . . . . . . . . . . . . . . . . . . . . . . . C . C C . . . . . . C . . . . . C . . . . . . . . . . .    
400	T T T T T T T T T T T T T T T T T T T T T T T T T T T T T T T . . T T T T T T T T T T T T T T T T T T T T T T T T T T T T T T T T T T T T T . T T T T T T T T T T . . . . . . . T T T T . . T . T T T T T T . . . . . . . . . . . . . . . . . . . . . . . . . . . . . . . . . . . . . . . . . . . . . . . . . . . . . . . T . T T . . . . . . T . . . . . T . . . . . . . . . . .    
401	A A A A A A A A A A A A A A A A A A A A A A A A A A A A A A A . . A A A A A A A A A A A A A A A A A A A A A A A A A A A A A A A A A A A A A . A A A A A A A A A A . . . . . . . A A A A . . A . A A A A A A . . . . . . . . . . . . . . . . . . . . . . . . . . . . . . . . . . . . . . . . . . . . . . . . . . . . . . . A . A A . . . . . . A . . . . . A . . . . . . . . . . .    
402	C C C C C C C C C C C C C C C C C C C C C C C C C C C C C C C . . C C C C C C C C C C C C C C C C C C C C C C C C C C C C C C C C C C C C C . C C C C C C C C C C . . . . . . . C C C C . . C . C C C C C C . . . . . . . . . . . . . . . . . . . . . . . . . . . . . . . . . . . . . . . . . . . . . . . . . . . . . . . C . C C . . . . . . C . . . . . C . . . . . . . . . . .    
403	T T T T T T T T T T T T T T T T T T T T T T T T T T T T T T T . . T T T T T T T T T T T T T T T T T T T T T T T T T T T T T T T T T T T T T . T T T T T T T T T T . . . . . . . T T T T . . T . T T T T T T . . . . . . . . . . . . . . . . . . . . . . . . . . . . . . . . . . . . T . . . . . . . . . . . . . . . . . . T . T T . . . . . . T . . . . . T . . . . . . . . . . .    
404	T T T T T T T T T T T T T T T T T T C T T T T T T T T T T T T . . T T T T T T T T T T T T T T T T T T T T T T T T T T T T T T T T T T T T T . T T T T T T T T T T . . . . . . . T T T T . . T . T T T T T T . . . . . . . . . . . . . . . . . . . . . . . . . . . . . . . . . . . . T . . . . . . . . . . . . . . . . . . T . T T . . . . . T T . . . . . T . . . . . . . . . . .    
405	T T T T T T T T T T T T T T T T T T T T T T T T T T T T T T T . . T T T T T T T T T T T T T T T T T T T T T T T T T T T T T T T T T T T T T . T T T T T T T T T T . . . . . . . T T T T . . T . T T T T T T . . . . . . . . . . . . . . . . . . . . . . . . . . . . . . . . . . . . T . . . . . . . . . . . . . . . . . . T . T T . . . . . T T . . . . . T . . . . . . . . . . .    
406	G G G G G G G G G G G G G G G G G G G G G G G G G G G G G G G . . G G G G G G G G G G G G G G G G G G G G G G G G G G G G G G G G G G G G G . G G G G G G G G G G . . . . . . . G G G G . . G . G G G G G G . . . . . . . . . . . . . . . . . . . . . . . . . . . . . . . . . . . . G . . . . . . . . . . . . . . . . . . G . G G . . . . . G G . . . . . G . . . . . . . . . . .    
407	T T T T T T T T T T T T T T T T T T T T T T T T T T T T T T T . . T T T T T T T T T T T T T T T T T T T T T T T T T T T T T T T T T T T T T . T T T T T T T T T T . . . . . . . T T T T . . T . T T T T T T . . . . . . . . . . . . . . . . . . . . . . . . . . . . . . . . . . . . T . . . . . . . . . . . . . . . . . . T . T T . . . . . T T . . . . . T . . . . . . . . . . .    
408	C C C C C C C C C C C C C C C C C C C C C C C C C C C C C C C . . C C C C C C C C C C C C C C C C C C C C C C C C C C C C C C C C C C C C C . C C C C C C C C C C . . . . . . . C C C C . . C . C C C C C C . . . . . . . . . . . . . . . . . . . . . . . . . . . . . . . . . . . . C . . . . . . . . . . . . . . . . . . C . C C . . . . . C C . . . . . C . . . . . . . . . . .    
409	C C C C C C C C C C C C C C C C C C C C C C C C C C C C C C C . . C C C C C C C C C C C C C C C C C C C C C C C C C C C C C C C C C C C C C . C C C C C C C C C C . . . . . . . C C C C . . C . C C C C C C . . . . . . . . . . . . . . . . . . . . . . . . . . . . . . . . . . . . C . . . . . . . . . . . . . . . . . . C . C C . . . . . C C . . . . . C . . . . . . . . . . .    
410	T T T T T T T T T T T T T T T T T T T T T T T T T T T T T T T . . T T T T T T T T T T T T T T T T T T T T T T T T T T T T T T T T T T T T T . T T T T T T T T T T . . . . . . . T T T T . . T . T T T T T T . . . . . . . . . . . . . . . . . . . . . . . . . . . . . . . . . . . . T . . . . . . . . . . . . . . . . . . T . T T . . . . . T T . . . . . T . . . . . . . . . . .    
411	G G G G G G G G G G G G G G G G G G G G G G G G G G G G G G G . . G G G G G G G G G G G G G G G G G G G G G G G G G G G G G G G G G G G G G . G G G G G G G G G G . . . . . . . G G G G . . G . G G G G G G . . . . . . . . . . . . . . . . . . . . . . . . . . . . . . . . . . . . G . . . . . . . . . . . . . . . . . . G . G G . . . . . G G . . . . . G . . . . . . . . . . .    
412	G G G G G G G G G G G G G G G G G G G G G G G G G G G G G G G . . G G G G G G G G G G G G G G G G G G G G G G G G G G G G G G G G G G G G G . G G G G G G G G G G . . . . . . . G G G G . . G . G G G G G G . . . . . . . . . . . . . . . . . . . . . . . . . . . . . . . . . . . . G . . . . . . . . . . . . . . . . . . G . G G . . . . . G G . . . . . G . . . . . . . . . . .    
413	G G G G G G G G G G G G G G G G G G G G G G G G G G G G G G G . . G G G G G G G G G G G G G G G G G G G G G G G G G G G G G G G G G G G G G . G G G G G G G G G G . . . . . . . G G G G . . G G G G G G G G . . . . . . . . . . . . . . . . . . . . . . . . . . . . . . . . . . . . G . . . . . . . . . . . . . . . . . . G . G G . . . . . G G . . . . . G . . . . . . . . . . .    
414	C C C C C C C C C C C C C C C C C C C C C C C C C C C C C C C . . C C C C C C C C C C C C C C C C C C C C C C C C C C C C C C C C C C C C C . C C C C C C C C C C . . . . . . . C C C C . . C C C C C C C C . . . . . . . . . . . . . . . . . . . . . . . . . . . . . . . . . . . . C . . . . . . . . . . . . . . . . . . C . C C . . . . . C C . . . . . C . . . . . . . . . . .    
415	C C C C C C C C C C C C C C C C C C C C C C C C C C C C C C C . . C C C C C C C C C C C C C C C C C C C C C C C C C C C C C C C C C C C C C . C C C C C C C C C C . . . . . . . C C C C . . C C C C C C C C . . . . . . . . . . . . . . . . . . . . . . . . . . . . . . . . . . . . C . . . . . . . . . . . . . . . . . . C . C C . . . . . C C . . . . . C . . . . . . . . . . .    
416	A A A A A A A A A A A A A A A A A A A A A A A A A A A A A A A . . A A A A A A A A A A A A A A A A A A A A A A A A A A A A A A A A A A A A A . A A A A A A A A A A . . . . . . . A A A A . . A A A A A A A A . . . . . . . . . . . . . . . . . . . . . . . . . . . . . . . . . . . A A . . . . . . . . . . . . . . . . . . A . A A . . . . . A A . . . . . A . . . . . . . . . . .    
417	G G G G G G G G G G G G G G G G G G G G G G G G G G G G G G G . . G G G G G G G G G G G G G G G G G G G G G G G G G G G G G G G G G G G G G . G G G G G G G G G G . . . . . . . G G G G G . G G G G G G G G . . . . . . . . . . . . . . . . . . . . . . . . . . . . . . . . . . . G G . . . . . . . . . . . . . . . . . . G . G G . . . . . G G . . . . . G . . . . . . . . . . .    
418	A A A A A A A A A A A A A A A A A A A A A A A A A A A A A A A . . A A A A A A A A A A A A A A A A A A A A A A A A A A A A A A A A A A A A A . A A A A A A A A A A . . . . . . . A A A A A . A A A A A A A A . . . . . . . . . . . . . . . . . . . . . . . . . . . . . . . . . . . A A . . . . . . . . . . . . . . . . . . A . A A . . . . . A A . . . . A A . . . . . . . . . . .    
419	A A A A A A A A A A A A A A A A A A A A A A A A A A A A A A A . . A A A A A A A A A A A A A A A A A A A A A A A A A A A A A A A A A A A A A . A A A A A A A A A A . . . . . . . A A A A A . A A A A A A A A . . . . . . . . . . . . . . . . . . . . . . . . . . . . . . . . . . . A A . . . . . . . . . . . . . . . . . . A . A A . . . . . A A . . . . A A . . . . . . . . . . .    
420	G G G G G G G G G G G G G G G G G G G G G G G G G G G G G G G . . G G G G G G G G G G G G G G G G G G G G G G G G G G G G G G G G G G G G G . G G G G G G G G G G . . . . . . . G G G G G . G G G G G G G G . . . . . . . . . . . . . . . . . . . . . . . . . . . . . . . . . . . G G . . . . . . . . . . . . . . . . . . G . G G . . . . . G G . . . . G G . . . . . . . . . . .    
421	A A A A A A A A A A A A A A A A A A A A A A A A A A A A A A A . . A A A A A A A A A A A A A A A A A A A A A A A A A A A A A A A A A A A A A . A G A A A A A A A A . . . . . . . A A A A A . A A A A A A A A . . . . . . . . . . . . . . . . . . . . . . . . . . . . . . . . . . . A A . . . . . . . . . . . . . . . . . . A . A A . . . . . A A . . . . A A . . . . . . . . . . .    
422	A A A A A A A A A A A A A A A A A A A A A A A A A A A A A A A . . A A A A A A A A A A A A A A A A A A A A A A A A A A A A A A A A A A A A A . A A A A A A A A A A . . . . . . . A A A A A A A A A A A A A A . . . . . . . . . . . . . . . . . . . . . . . . . . . . . . . . . . . A A . . . . . . . . . . . . . . . . . . A . A A . . . . . A A . . . . A A . . . . . . . . . . .    
423	C C C C C C C C C C C C C C C C C C C C C C C C C C C C C C C . . C C C C C C C C C C C C C C C C C C C C C C C C C C C C C C C C C C C C C . C C C C C C C C C C . . . . . . . C C C C C C C C C C C C C C . . . . . . . . . . . . . . . . . . . . . . . . . . . . . . . . . . . C C . . . . . . . . . . . . . . . . . . C . C C . . . . . C C . . . . C C . . . . . . . . . . .    
424	G G G G G G G G G G G G G G G G G G G G G G G G G G G G G G G . . G G G G G G G G G G G G G G G G G G G G G G G G G G G G G G G G G G G G G . G G G G G G G G G G . . . . . . . G G G G G G G G G G G G G G . . . . . . . . . . . . . . . . . . . . . . . . . . . . . . . . . . . G G . . . . . . . . . . . . . . . . . . G . G G . . . . . G G . . . . G G . . . . . . . . . . .    
425	A A A A A A A A A A A A A A A A A A A A A A A A A A A A A A A . . A A A A A A A A A A A A A A A A A A A A A A A A A A A A A A A A A A A A A . A A A A A A A A A A . . . . . . . A A A A A A A A A A A A A A . . . . . . . . . . . . . . . . . . . . . . . . . . . . . . . . . . . A A . . . . . . . . . . . . . . . . . . A . A A . . . . . A A . . . . A A . . . . . . A . . . .    
426	C C C C C C C C C C C C C C C C C C C C C C C C C C C C C C C . . C C C C C C C C C C C C C C C C C C C C C C C C C C C C C C C C C C C C C . C C C C C C C C C C . . . . . . . C C C C C C C C C C C C C C . . . . . . . . . . . . . . . . . . . . . . . . . . . . . . . . . . . C C . . . . . . . . . . . . . . . . . . C . C C . . . . . C C . . . . C C . . . . . . C . . . .    
427	A A A A A A A A A A A A A A A A A A A A A A A A A A A A A A A . . A A A A A A A A A A A A A A A A A A A A A A A A A A A A A A A A A A A A A . A A A A A A A A A A . . . . . . . A A A A A A A A A A A A A A . . . . . . . . . . . . . . . . . . . . . . . . . . . . . . . . . A . A A . . . . . . . . . . . . . . . . . . A . A A . . . . . A A . . . . A A . . . . . . A . . . .    
428	T T T T T T T T T T T T T T T T T T T T T T T T T T T T T T T . . T T T T T T T T T T T T T T T T T T T T T T T T T T T T T T T T T T T T T . T T T T T T T T T T . . . . . . . T T T T T T T T T T T T T T . . . . . T . . . . . . . . . . . . . . T . . . . . . . . . . . . T . T T . . . . . . . . . . . . . . . . . . T . T T . . . . T T T . . . . T T . . . . . T T . . . .    
429	T T T T T T T T T T T T T T T T T T T T T T T T T T T T T T T . . T T T T T T T T T T T T T T T T T T T T T T T T T T T T T T T T T T T T T . T T T T T T T T T T . . . . . . . T T T T T T T T T T T T T T . . . . . T . . . . . . . . . . . . . . A . . . . . . . . . . . . T . T T . . . . . . . . . . . . . . . . . . A . T T . . . . T T T . . . . T T . . . . . A T . . . .    
430	A A A A A A A A A A A A A A A A A A A A A A A A A A A A A A A . . A A A A A A A A A A A A A A A A A A A A A A A A A A A A A A A A A A A A A . A A A A A A A A A A . . . . . . . A A A A A A A A A A A A A A . . . . . A . . . . . . . . . . . . . . A . . . . . . . . . . . A A . A A . . . . . . . . . . . . . . . . . . A . A A . . . . A A A . . . . A A . . . . . A A . . . .    
431	A A A A A A A A A A A A A A A A A A A A A A A A A A A A A A A . . A A A A A A A A A A A A A A A A A A A A A A A A A A A A A A A A A A A A A . A A A A A A A A A A . . . . . . . A A A A A A A A A A A A A A . . . . . A . . . . . . . . . . . . . . A . . . . . . . . . . . A A . A A . . . . . . . . . . . . . . . . . . A . A A . . . . A A A . . . . A A . . . . . A A . . . .    
432	A A A A A A A A A A A A A A A A A A A A A A A A A A A A A A A . . A A A A A A A A A A A A A A A A A A A A A A A A A A A A A A A A A A A A A . A A A A A A A A A A . . . . . . . A A A A A A A A A A A A A A . . . . . A . . . . . . . . . . . . . . A . . . . . . . . . . . A A . A A . . . . . . . . . . . . . . . . . . A . A A . . . . A A A . . . . A A . . . . . A A . . . .    
433	G G G G G G G G G G G G G G G G G G G G G G G G G G G G G G G . . G G G G G G G G G G G G G G G G G G G G G G G G G G G G G G G G G G G G G . G G G G G G G G G G . . . . . . . G G G G G G G G G G G G G G . . . . . G . . . . . . . . . . . . . . G . . . . . . . . . . . G G . G G . . . . . . . . . . . . . . . . . . G . G G . . . . G G G . . . . G G . . . . . G G . . . .    
434	A A A A A A A A A A A A A A A A A A A A A A A A A A A A A A A . . A A A A A A A A A A A A A A A A A A A A A A A A A A A A A A A A A A A A A . A A A A A A A A A A . . . . . . . A A A A A A A A A A A A A A . . . . . A . . . . . . . . . . . . . . A . . . . . . . . . . . A A . A A . . . . . . . . . . . . . . . . . . A . A A . . . . A A A . . . . A A . . . . . A A . . . .    
435	C C C C C C C C C C C C C C C C C C C C C C C C C C C C C C C . . C C C C C C C C C C C C C C C C C C C C C C C C C C C C C C C C C C C C C . C C C C C C C C C C . . . . . . . C C C C C C C C C C C C C C . . . . . C . . . . . . . . . . . . . . C . . . . . . . . . . . C C . C C . . . . . . . . . . . . . . . . . . C . C C . . . . C C C . . . . C C . . . . . C C . . . .    
436	C C C C C C C C C C C C C C C C C C C C C C C C C C C C C C C . . C C C C C C C C C C C C C C C C C C C C C C C C C C C C C C C C C C C C C . C C C C C C C C C C . . . . . . . C C C C C C C C C C C C C C . . . . . C . . . . . . . . . . . . . . C . . . . . . . . . . . C C . C C . . . . . . . . . . . . . . . . . . C . C C . . . . C C C . . . . C C . . . . . C C . . . .    
437	T T T T T T T T T T T T T T T T T T T T T T T T T T T T T T T . . T T T T T T T T T T T T T T T T T T T T T T T T T T T T T T T T T T T T T . T T T T T T T T T T . . . . . . . T T T T T T T T T T T T T T . . . . . T . . . . . . . . . . . . . . T . . . . . . . . . . . T T . T T . . . . . . . . . . . . . . . . . . T . T T . . . . T T T . . . T T T . . . . . T T . . . .    
438	G G G G G G G G G G G G G G G G G G G G G G G G G G G G G G G . . G G G G G G G G G G G G G G G G G G G G G G G G G G G G G G G G G G G G G . G G G G G G G G G G . . . . . . . G G G G G G G G G G G G G G . . . . . G . . . . . . . . . . . . . . G . G . . . . . . . . . G G . G G . . . . . . . . . . . . . . . . . . G . G G . . . . G G G . . . G G G . . . . . G G . . . .    
439	C C C C C C C C C C C C C C C C C C C C C C C C C C C C C C C . . C C C C C C C C C C C C C C C C C C C C C C C C C C C C C C C C C C C C C . C C C C C C C C C C . . . . . . . C C C C C C C C C C C C C C . . . . . C . . . . . . . . . . . . . . C . C . . . . . . . . C C C . C C . . . . . . . . . . . . . . . . . . C . C C . . . . C C C . . . C C C . . . . . C C . . . C    
440	A A A A A A A A A A A A A A A A A A A A A A A A A A A A A A A . . A A A A A A A A A A A A A A A A A A A A A A A A A A A A A A A A A A A A A . A A A A A A A A A A . . . . . . . A A A A A A A A A G A A A A . . . . . A . . . . . . . . . . . . . . A . A . . . . . . . . A A A . A A . . . . . . . . . . . . . . . . . . A . A A . . . . A A A . . . A A A . . . . . A A . . . A    
441	G G G G G G G G G G G G G G G G G G G G G G G G G G G G G G G . . G G G G G G G G G G G G G G G G G G G G G G G G G G G G G G G G G G G G G . G G G G G G G G G G . . . . . . . G G G G G G G G G G G G G G . . . . . G . . . . . . . . . . . . . . G . G . . . . . . . . G G G . G G . . . . . . . . . . . . . . . . . . G . G G . . . . G G G . . . G G G . . . . . G G . . . G    
442	G G G G G G G G G G G G G G G G G G G G G G G G G G G G G G G . G G G G G G G G G G G G G G G G G G G G G G G G G G G G G G G G G G G G G G . G G G G G G G G G G . . . . . . . G G G G G G G G G G G G G G . . . . . G . . . . . . . . . . . . . . G . G . . . . . . . . G G G . G G . . . . . . . . . . . . . . . . . . G . G G . . . . G G G . . . G G G . . . . . G G . . . G    
443	A A A A A A A A A A A A A A A A A A A A A A A A A A A A A A A . A A A A A A A A A A A A A A A A A A A A A A A A A A A A A A A A A A A A A A . A A A A A A A A A A . . . . . . . A A A A A A A A A A A A A A . . . . . A . . . . . . . . . . . . . . A . A . . . . . . . . A A A . A A . . . . . . . . . . . . . . . . . . A . A A . . . . A A A . . . A A A . . . . . A A . . . A    
444	A A A A A A A A A A A A A A A A A A A A A A A A A A A A A A A . A A A A A A A A A A A A A A A A A A A A A A A A A A A A A A A A A A A A A A . A A A A A A A A A A . . . . . . . A A A A A A A A A A A A A A . . . . . A . . . . . . . . . . . . . . A . A . . . . . . . . A A A . A A . . . . . . . . . . . . . . . . . . A . A A . . . . A A A . . . A A A . . . . . A A . . . A    
445	G G G G G G G G G G G G G G G G G G G G G G G G G G G G G G G . G G G G G G G G G G G G G G G G G G G G G G G G G G G G G G G G G G G G G G . G G G G G G G G G G . . . . . . . G G G G G G G G G G G G G G . . . . . G . . . . . . . . . . . . . . G . G . . . . . . . . G G G . G G . . . . . . . . . . . . . . . . . . G . G G . . . . G G G . . G G G G . . . . . G G . . . G    
446	A A A A A A A A A A A A A A A A A A A A A A A A A A A A A A A . A A A A A A A A A A A A A A A A A A A A A A A A A A A A A A A A A A A A A A . A A A A A A A A A A . . . . . . . A A A A A A A A A A A A A A . . . . . A . . . . . . . . . . . . . . A . A . . . . . . . . A A A . A A . . . . . . . . . . . . . . . . . . A . A A . . . . A A A . . A A A A . . . . . A A . . . A    
447	G G G G G G G G G G G G G G G G G G G G G G G G G G G G G G G . G G G G G G G G G G G G G G G G G G G G G G G G G G G G G G G G G G G G G G . G G G G G G G G G G . . . . . . . G G G G G G G G G G G G G G . . . . . G . . G . . . . . . . . . . . G . G . . . . . . . . G G G . G G . . . . . . . . . . . . . . . . . . G . G G . . . . G G G . . G G G G . . . . . G G . . . G    
448	A A A A A A A A A A A A A A A A A A A A A A A A A A A A A A A . A A A A A A A A A A A A A A A A A A A A A A A A A A A A A A A A A A A A A A . A A A A A A A A A A . . . . . . . A A A A A A A A A A A A A A . . . . . A . . A . . . . . . . . . . . A . A . . . . . . . . A A A . A A . . . . . . . . . . . . . . . . . . A . A A . . . . A A A . . A A A A . . . . . A A . . . A    
449	G G G G G G G G G G G G G G G G G G G G G G G G G G G G G G G . G G G G G G G G G G G G G G G G G G G G G G G G G G G G G G G G G G G G G G . G G G G G G G G G G . . . . . . . G G G G G G G G G G G G G G . . . . . G . . G . . . . . . . . . . . G . G . . . . . . . . G G G . G G . . . . . . . . . . . . . . . . . . G . G G . . . . G G G . . G G G G . . . . . G G . . . G    
450	C C C C C C C C C C C C C C C C C C C C C C C C C C C C C C C . C C C C C C C C C C C C C C C C C C C C C C C C C C C C C C C C C C C C C C . C C C C C C C C C C . . . . . . . C C C C C C C C C C C C C C . . . . . C . . C . . . . . . . . . . . C . C . . . . . . . . C C C . C C . . . . . . . . . . . . . . . . . . C . C C . . . . C C C . . C C C C . . . . . C C . . . C    
451	C C C C C C C C C C C C C C C C C C C C C C C C C C C C C C C . C C C C C C C C C C C C C C C C C C C C C C C C C C C C C C C C C C C C C C . C C C C C C C C C C . . . . . . . C C C C C C C C C C C C C C . . . . . C . . C . . . . . . . . . . . C . C . . . . . . . . C C C . C C . . . . . . . . . . . . . . . . . . C . C C . . . . C C C . . C C C C . . . . . C C . . . C    
452	A A A A A A A A A A A A A A A A A A A A A A A A A A A A A A A . A A A A A A A A A A A A A A A A G A A A A A A A A A A A A A A A A A A A A A . A A A A A A A A A A . . . . . . . A A A A A A A A A A A A A A . . . . . A . . A . . . . . . . . . . . A . A . . . . . . . . A A A . A A . . . . . . . . . . . . . . . . . . A . A A . . . . A A A . . A A A A . . . . . A A . . . A    
453	C C C C C C C C C C C C C C C C C C C C C C C C C C C C C C C . C C C C C C C C C C C C C C C C C C C C C C C C C C C C C C C C C C C C C C . C C C C C C C C C C . . . . . . . C C C C C C C C C C C C C C . . . . . C . . C . . . . . . . . . . . C . C . . . . . . . . C C C . C C . . . . . . . . . . . . . . . . . . C . C C . . . . C C C . . C C C C . . . . . C C . . . C    
454	T T T T T T T T T T T T T T T T T T T T T T T T T T T T T T T . T T T T T T T T T T T T T T T T T T T T T T T T T T T T T T T T T T T T T T . T T T T T T T T T T . . . . . . . T T T T T T T T T T T T T T . . . . . T . . T . . . . . . . . . . . T . T . . . . . . . . T T T . T T . . . . . . . . . . . . . . . . . . T . T T . . . . T T T . . T T T T . . . . . T T . . . T    
455	C C C C C C C C C C C C C C C C C C C C C C C C C C C C C C C . C C C C C C C C C C C C C C C C C C C C C C C C C C C C C C C C C C C C C C . C C C C C C C C C C . . . . . . . C C C C C C C C C C C C C C . . . . . C . . C . . . . . . . . . . . C . C . . . . . . . . C C C . C C . . . . . . . . . . . . . . . . . . C . C C . . . . C C C . . C C C C . . . . . C C . . . C    
456	C C C C C C C C C C C C C C C C C C C C C C C C C C C C C C C . C C C C C C C C C C C C C C C C C C C C C C C C C C C C C C C C C C C C C C . C C C C C C C C C C . . . . . . . C C C C C C C C C C C C C C . . . . . C . . C . . . . . . . . . . . C . C . . . . . . . . C C C . C C . . . . . . . . . . . . . . . . . . C . C C . . . . C C C . . C C C C . . . . . C C . . . C    
457	C C C C C C C C C C C C C C C C C C C C C C C C C C C C C C C . C C C C C C C C C C C C C C C C C C C C C C C C C C C C C C C C C C C C C C . C C C C C C C C C C . . . . . . . C C C C C C C C C C C C C C . . . . . C . . C . . . . . . . . . . . C . C . . . . . . . . C C C . C C . . . . . . . . . . . . . . . . . . C . C C . . . . C C C . . C C C C . . . . . C C . . . C    
458	T T T T T T T T T T T T T T T T T T T T T T T T T T T T T T T . T T T T T T T T T T T T T T T T T T T T T T T T T T T T T T T T T T T T T T . T T T T T T T T T T . . . . . . . T T T T T T T T T T T T T T . . . . . T . . T . . . . . . . . . . . T . T . . . . . . . . T T T T T T . . . . . . . . . . . . . . . . . . T . T T . . . . T T T . . T T T T . . . . . T T . . . T    
459	G G G G G G G G G G G G G G G G G G G G G G G G G G G G G G G . G G G G G G G G G G G G G G G G G G G G G G G G G G G G G G G G G G G G G G . G G G G G G G G G G . . . . . . . G G G G G G G G G G G G G G . . . . . G . . G . . . . . . . . . . . G . G . . . . . . . . G G G G G G . . . . . . . . . . . . . . . . . . G . G G . . . . G G G . . G G G G . . . . . G G . . . G    
460	A A A A A A A A A A A A A A A A A A A A A A A A A A A A A A A . A A A A A A A A A A A A A A A A A A A A A A A A C A A A A A A A A A A A A A . A A A A A A A A A A . . . . . . . A A A A A A A A A A A A A A . . . . . A . . A . . . . . . . . . . . A . A . . . . . . . . A A A A A A . . . . . . . . . . . . . . . . . . A . A A . . . . A A A . . A A A A . . . . . A A . . . A    
461	A A A A A A A A A A A A A A A A A A A A A A A A A A A A A A A . A A A A A A A A A A A A A A A A A A A A A A A A A A A A A A A A A A A A A A . A A A A A A A A A A . . . . . . . A A A A A A A A A A A A A A . . . . . A . . A . . . . . . . . . . . A . A . . . . . . . . A A A A A A . . . . . . . . . . . . . . . . . . A . A A . . . . A A A . . A A A A . . . . . A A . . A A    
462	G G G G G G G G G G G G G G G G G G G G G G G G G G G G G G G . G G G G G G G G G G G G G G G G G G G G G G G G G G G G G G G G G G G G G G . G G G G G G G G G G . . . . . . . G G G G G G G G G G G G G G . . . . . G . . G . . . . . . . . . . . G . G . . . . . . . . G G G G G G . . . . . . . . . . . . . . . . . . G . G G . . . . G G G . . G G G G . . . . . G G . . G G    
463	A A A A A A A A A A A A A A A A A A A A A A A A A A A A A A A . A A A A A A A A A A A A A A A A A A A A A A A A A A A A A A A A A A A A A A . A A A A A A A A A A . . . . . . . A A A A A A A A A A A A A A . . . . . A . . A . . . . . . . . . . . A . A . . . . . . . . A A A A A A . . . . . . . . . . . . . . . . . . A . A A . . . . A A A . . A A A A . . . . . A A . . A A    
464	A A A A A A A A A A A A A A A A A A A A A A A A A A A A A A A . A A A A A A A A A A A A A A A A A A A A A A A A A A A A A A A A A A A A A A . A A A A A A A A A A . . . . . . . A A A A A A A A A A A A A A . . . . . A . . A . . . . . . . . . . . A . A . . . . . . . . A A A A A A . . . . . . . . . . . . . . . . . . A . A A . . . . A A A . . A A A A . . . . . A A . . A A    
465	T G G G G G G G G G G G G G G G G G G G G G G G G G G G G G G . G G G G G G G G G G G G G G G G G G G G G G G G G G G G G G G G G G G G G G . G G G G G G G G G G . . . . . . . G G G G G G G G G G G G G G . . . . . G . . G . . . . . . . . . . . G . G . . . . . . . . G G G G G G . . . . . . . . . . . . . . . . . . G . G G . . . . G G G . . G G G G . . . . . G G . . G G    
466	. G G G G G G G G G G G G G G G G G G G G G G G G G G G G G G . G G G G G G G G G G G G G G G G G G G G G G G G G G G G G G G G G G G G G G . G G G G G G G G G G . . . . . . . G G G G G G G G G G G G G G . . . . . G . . G . . . . . . . . . . . G . G . . . . . . . . G G G G G G . . . . . . . . . . . . . . . . . . G . G G . . . . G G G . . G G G G . . . . . G G . . G G    
467	. A A A A A A A A A A A A A A A A A A A A A A A A A A A A A A . A A A A A A A A A A A A A A A A A A A A A A A A A A A A A A A A A A A A A A . A A A A A A A A A A . . . . . . . A A A A A A A A A A A A A A . . . . . A . . A . . . . . . . . . . . A . A . . . . . . . A A A A A A A . . . . . . . . . . . . . . . . . . A . A A . . . . A A A . . A A A A . . . . . A A . . A A    
468	. G G G G G G G G G G G G G G G G G G G G G G G G G G G G G G . G G G G G G G G G G G G G G G G G G G G G G G G G G G G G G G G G G G G G G . G G G G G G G G G G . . . . . . . G G G G G G G G G G G G G G . . . . . G . . G . . . . . . . . . . . G . G . . . . . . . G G G G G G G . . . . . . . . . . . . . . . . . . G . G G . . . . G G G . . G G G G . . . . . G G . . G G    
469	. C C C C C C C C C C C C C C C C C C C C C C C C C C C C C C . C C C C C C C C C C C C C C C C C C C C C C C C C C C C C C C C C C C C C C . C C C C C C C C C C . . . . . . . C C C C C C C C C C C C C C . . . . . C . . C . . . . . . . . . . . C . C . . . . . . . C C C C C C C . . . . . . . . . . . . . . . . . . C . C C . . . . C C C . . C C C C . . . . . C C . . C C    
470	. T T T T T T T T T T T T T T T T T T T T T T T T T T T T T T . T T T T T T T T T T T T T T T T T T T T T T T T T T T T T T T T T T T T T T . T T T T T T T T T T . . . . . . . T T T T T T T T T T T T T T . . . . . T . . T . . . . . . . . . . . T . T . . . . . . . T T T T T T T . . . . . . . . . . . . . . . . . . T . T T . . . . T T T . . T T T T . . . . . T T . . T T    
471	. G G G G G G G G G G G G T G G G G G G G G G G G G G G G G G . G G G G G G G G G G G G G G G G G G G G G G G G G G G G G G G G G G G G G G . G G G G G G G G G G . . . . . . . G G G G G G G G G G G G G G . . . . . G . . G . . . . . . . . . . . G . G . . . . G G . G G G G G G G . . . . . . . . . . . . . . . . . . G . G G . . . . G G G . . G G G G . . . . . G G . . G G    
472	. A A A A A A A A A A A A A A A A A A A A A A A A A A A A A A . A A A A A A A A A A A A A A A A A A A A A A A A A A A A A A A A A A A A A A . A A A A A A A A A A . . . . . . . A A A A A A A A A A A A A A . . . . . A . . A . . . . . . . . . . . A . A . . . . A A . A A A A A A A . . . . . . . . . . . . . . . . . . A . A A . . . . A A A . . A A A A . . . . . A A . . A A    
473	. G G G G G G G G G G G G G G G G G G G G G G G G G G G G G G . G G G G G G G G G G G G G G G G G G G G G G G G G G G G G G G G G G G G G G . G G G G G G G G G G . . . . . . . G G G G G G G G G G G G G G . . . . . G . G G . . . . . . . . . . . G . G . . . . G G . G G G G G G G . . . . . . . . . . . . . . . . . . G . G G . . . . G G G . . G G G G . . . . . G G . . G G    
474	. T T T T T T T T T T T T T T T T T T T T T T T T T T T T T T . T T T T T T T T T T T T T T T T T T T T T T T T T T T T T T T T T T T T T T . T T T T T T T T T T . . . . . . . T T T T T T T T T T T T T T . . . . . T . T T . . . . . . . . . . . T . T . . . . T T . T T T T T T T . . . . . . . . . . . . . . . . . . T . T T . . . . T T T . . T T T T . . . . . T T . . T T    
475	. C C C C C C C C C C C C C C C C C C C C C C C C C C C C C C . C C C C C C C C C C C C C C C C C C C C C C C C C C C C C C C C C C C C C C . C C C C C C C C C C . . . . . . . C C C C C C C C C C C C C C . . . . . C . C C . . . . . . . . . . . C . C . . . . C C . C C C C C C C . . . . . . . . . . . . . . . . . . C . C C . . . . C C C . . C C C C . . . . . C C . . C C    
476	. A A A A A A A A A A A A A A A A A A A A A A A A A A A A A A . A A A A A A A A A A A A A A A A A A A A A A A A A A A A A A A A A A A A A A . A A A A A A A A A A . . . . . . . A A A A A A A A A A A A A A . . . . . A . A A . . . . . . . . . . . A . A . . . . A A . A A A A A A A . . . . . . . . . . . . . . . . . . A . A A . . . . A A A . . A A A A . . . . . A A . . A A    
477	. A A A A A A A A A A A A A A A A A A A A A A A A A A A A A A . A A A A A A A A A A A A A A A A A A A A A A A A A A A A A A A A A A A A A A . A A A A A A A A A A . . . . . . . A A A A A A A A A A A A A A . . . . . A . A A . . . . . . . . . . . A . A . . . . A A . A A A A A A A . . . . . . . . . . . . . . . . . . A . A A . . . . A A A . . A A A A . . . . A A A . . A A    
478	. G G T G G G G G G G G G G G G G G G G G G G G G G G G G G G . G G G G G G G G G G G G G G G G G G G G G G G G G G G G G G G G G G G G G G . G G G G G G G G G G . . . . . . . G G G G G G G G G G G G G G . . . . . G . G G . . . . . . . . . . . G . G . . . . G G . G G G G G G G . . . . . . . . . . . . . . . . . . G . G G . . . . G G G . . G G G G . . . . G G G . . G G    
479	. G G G G G G G G G G G G G G G G G G G G G G G G G G G G G G . G G G G G G G G G G G G G G G G G G G G G G G G G G G G G G G G G G G G G G . G G G G G G G G G G . . . . . . . G G G G G G G G G G G G G G . . . . . G . G G . . . . . . . . . . . G . G . . . . G G . G G G G G G G . . . . . . . . . . . . . . . . . . G . G G . . . . G G G . . G G G G . . . . G G G . . G G    
480	. G G G G G G G G G G G G G G G G G G G G G G G G G G G G G G . G G G G G G G G G G G G G G G G G G G G G G G G G G G G G G G G G G G G G G . G G G G G G G G G G . . . . . . . G G G G G G G G G G G G G G . . . . . G . G G . . . . . . . . . . . G . G . . . . G G . G G G G G G G . . . . . . . . . . . . . . . . . . G . G G . . . . G G G . . G G G G . . . . G G G . . G G    
481	. C C C C C C C C C C C C C C C C C C C C C C C C C C C C C C . C C C C C C C C C C C C C C C C C C C C C C C C C C C C C C C C C C C C C C . C C C C C C C C C C . . . . . . . C C C C C C C C C C C C C C . . . . . C . C C . . . . . . . . . . . C . C . . . . C C . C C C C C C C . . . . . . . . . . . . . . . . . . C . C C . . . . C C C . . C C C C . . . . C C C . . C C    
482	. A A A A A A A A A A A A A A A A A A A A A A A A A A A A A A . A A A A A A A A A A A A A A A A A A A A A A A A A A A A A A A A A A A A A A . A A A A A A A A A A . . . . . . . A A A A A A A A A A A A A A . . . . . A . A A . . . . . . . . . . . A . A . . . . A A . A A A A A A A . . . . . . . . . . . . . . . . . . A . A A . . . . A A A . . A A A A . . . . A A A . . A A    
483	. C C C C C C C C C C C C C C C C C C C C C C C C C C C C C C . C C C C C C C C C C C C C C C C C C C C C C C C C C C C C C C C C C C C C C . C C C C C C C C C C . . . . . . C C C C C C C C C C C C C C C . . . . . C . C C . . . . . . . . . . . C . C . . . . C C . C C C C C C C . . . . . . . . . . . . . . . . . . C . C C . . . . C C C . . C C C C . . . . C C C . . C C    
484	. G G G G G G G G G G G G G G G G G G G G G G G G G G G G G G . G G G G G G G G G G G G G G G G G G G G G G G G G G G G G G G G G G G G G G . G G G G G G G G G G . . . . . . G G G G G G G G G G G G G G G . . . . . G G G G . . . . . . . . . . . G . G . . . . G G . G G G G G G G . . . . . . . . . . . . . . . . . . G . G G . . . . G G G . . G G G G . . . . G G G . . G G    
485	. C C C C C C C C C C C C C C C C C C C C C C C C C C C C C C . C C C C C C C C C C C C C C C C C C C C C C C C C C C C C C C C C C C C C C . C C C C C C C C C C . . . . . . C C C C C C C C C C C C C C C . . . . . C C C C . . . . . . . . . . . C . C . . . . C C . C C C C C C C . . . . . . . . . . . . . . . . . . C . C C . . . . C C C . . C C C C . . . . C C C . . C C    
486	. T T T T T T T T T T T T T T T T T T T T T T T T T T T T T T . T T T T T T T T T T T T T T T T T T T T T T T T T T T T T T T T T T T T T T . T T T T T T T T T T . . . . . . T T T T T T T T T T T T T T T . . . . . T T T T . . . . . . . . . . . T . T . . . . T T . T T T T T T T . . . . . . . . . . . . . . . . . . T . T T . . . . T T T . . T T T T . . . . T T T . . T T    
487	. G G G G G G G G G G G G G G G G G G G G G G G G G G G G G G . G G G G G G G G G G G G G G G G G G G G G G G G G G G G G G G G G G G G G G . G G G G G G G G G G . . . . . . G G G G G G G G G G G G G G G . . . . . G G G G . . . . . . . . . . . G . G . . . . G G . G G G G G G G . . . . . . . . . . . . . . . . . . G . G G . . . . G G G . . G G G G . . . . G G G . . G G    
488	. C C C C C C C C C C C C C C C C C C C C C C C C C C C C C C . C C C C C C C C C C C C C C C C C C C C C C C C C C C C C C C C C C C C C C . C C C C C C C C C C . . . . . . C C C C C C C C C C C C C C C . . . . . C C C C . . . . . . . . . . . C . C . . . . C C . C C C C C C C . . . . . . . . . . . . . . . . . . C . C C . . . . C C C . . C C C C . . . . C C C . . C C    
489	. T T T T T T T T T T T T T T T T T T T T T T T T T T T T T T . T T T T T T T T T T T T T T T T T T T T T T T T T T T T T T T T T T T T T T . T T T T T T T T T T . . . . . . T T T T T T T T T T T T T T T . . T . T T T T T . . . . . . . . . . . T . T . . . . T T . T T T T T T T . . . . . . . . . . . . . . . . . . T . T T . . . . T T T . . T T T T . . . . T T T . . T T    
490	. G G G G G G G G G G G G G G G G G G G G G G G G G G G G G G . G G G G G G G G G G G G G G G G G G G G G G G G G G G G G G G G G G G G G G . G G G G G G G G G G . . . . . . G G G G G G G G G G G G G G G . . G . G G G G G . . . . . . . . . . . G . G . . . . G G . G G G G G G G . . . . . . . . . . . . . . . . . . G . G G . . . . G G G . . G G G G . . . . G G G . . G G    
491	. C C C C C C C C C C C C C C C C C C C C C C C C C C C C C C . C C C C C C C C C C C C C C C C C C C C C C C C C C C C C C C C C C C C C C . C C C C C C C C C C . . . . . . C C C C C C C C C C C C C C C . . C . C C C C C . . . . . . . . . . . C . C . . . . C C . C C C C C C C . . . . . . . . . . . . . . . . . . C . C C . . . . C C C . . C C C C . . . . C C C . . C C    
492	. T T T T T T T T T T T T T T T T T C T T T T T T T T T T T T . T T T T T T T T T T T T T T T T T T T T T T T T T T T T T T T T T T T T T T . T T T T T T T T T T . . . . . . T T T T T T T T T T T T T T T . . T . T T T T T . . . . . . . . . . . T . T . . . . T T . T T T T T T T . . . . . . . . . . . . . . . . . . T . T T . . . . T T T . . T T T T . . . . T T T . . T T    
493	. G G G G G G G G G G G G G G G G G G G G G G G G G G G G G G . G G G G G G G G G G G G G G G G G G G G G G G G G G G G G G G G G G G G G G . G G G G G G G G G G . . . . . . G G G G G G G G G G G G G G G . . G . G G G G G . . . . . . . . . . . G . G . . . . G G G G G G G G G G . . . . . . . . . . . . . . . . . . G . G G . . . . G G G . . G G G G . . . . G G G . . G G    
494	. T T T T T T T T T T T T T T T T T T T T T T T T T T T T T T . T T T T T T T T T T T T T T T T T T T T T T T T T T T T T T T T T T T T T T . T T T T T T T T T T . . . . . . T T T T T T T T T T T T T T T . . T . T T T T T . . . . . . . . . . . T . T . . . . T T T T T T T T T T . . . . . . . . . . . . . . . . . . T . T T . . . . T T T . . T T T T . . . . T T T . . T T    
495	. C C C C C C C C C C C C C C C C C C C C C C C C C C C C C C . C C C C C C C C C C C C C C C C C C C C C C C C C C C C C C C C C C C C C C . C C C C C C C C C C . . . . . . C C C C C C C C C C C C C C C . . C . C C C C C . . . . . . . . . . . C . C . . . . C C C C C C C C C C . . . . . . . . . . . . . . . . . . C . C C . . . . C C C . . C C C C . . . . C C C . . C C    
496	. C C C C C C C C C C C C C C C C C C C C C C C C C C C C C C . C C C C C C C C C C C C C C C C C C C C C C C C C C C C C C C C C C C C C C . C C C C C C C C C C . . . . . . C C C C C C C C C C C C C C C . . C . C C C C C . . . . . . . . . . . C . C . . . . C C C C C C C C C C . . . . . . . . . . . . . . . . . . C . C C . . . . C C C . . C C C C . . . . C C C . . C C    
497	. C C C C C C C C C C C C C C C C C C C C C C C C C C C C C C . C C C C C C C C C C C C C C C C C C C C C C C C C C C C C C C C C C C C C C . C C C C C C C C C C . . . . . . C C C C C C C C C C C C C C C . . C . C C C C C . . . . . . . . . . . C . C . . . . C C C C C C C C C C . . . . . . . . . . . . . . . . . . C . C C . . . . C C C . . C C C C . . . . C C C . . C C    
498	. C C C C C C C C C C C C C C C C C C C C C C C C C C C C C C . C C C C C C C C C C C C C C C C C C C C C C C C C C C C C C C C C C C C C C . C C C C C C C C C C . . . . . . C C C C C C C C C C C C C C C . . C . C C C C C . . . . . . . . . . . C . C . . . . C C C C C C C C C C . . . . . . . . . . . . . . . . . . C . C C . . . . C C C . . C C C C . . . . C C C . . C C    
499	. A A A A A A A A A A A A A A A A A A A A A A A A A A A A A A . A A A A A A A A A A A A A A A A A A A A A A A A A A A A A A A A A A A A A A . A A A A A A A A A A . . . . . . A A A A A A A A A A A A A A A . . A . A A A A A . . . . . . . . . . . A . A . . . . A A A A A A A A A A . . . . . . . . . . . . . . . . . . A . A A . . . . A A A . . A A A A . . . . A A A . . A A    
500	. T T T T T T T T T T T T T T T T T T T T T T T T T T T T T T . T T T T T T T T T T T T T T T T T T T T T T T T T T T T T T T T T T T T T T . T T T T T T T T T T . . . . . . T T T T T T T T T T T T T T T . . T . T T T T T . . . . . . . . . . . T . T . . . . T T T T T T T T T T . . . . . . . . . . . . . . . . . . T . T T . . . . T T T . . T T T T . . . . T T T . . T T    
501	. G G G G G G G G G G G G G G G G G G G G G G G G G G G G G G . G G G G G G G G G G G G G G G G G G G G G G G G G G G G G G G G G G G G G G . G G G G G G G G G G . . . . . . G G G G G G G G G G G G G G G . . G . G G G G G . . . . . . . . . . . G . G . . . . G G G G G G G G G G . . . . . . . . . . . . . . . . . . G . G G . . . . G G G . . G G G G . . . . G G G . . G G    
502	. A A A A A A A A A A A A A A A A A A A A A A A A A A A A A A . A A A A A A A A A A A A A A A A A A A A A A A A A A A A A A A A A A A A A A . A A A A A A A A A A . . . . . . A A A A A A A A A A A A A A A . . A . A A A A A . . . . . . . . . . . A . A . . . . A A A A A A A A A A . . . . . . . . . . . . . . . . . . A . A A . . . . A A A . A A A A A . . . . A A A . . A A    
503	. A A A A A A A A A A A A A A A A A A A A A A A A A A A A A A . A A A A A A A A A A A A A A A A A A A A A A A A A A A A A A A A A A A A A A . A A A A A A A A A A . . . . . . A A A A A A A A A A A A A A A . . A . A A A A A . . . . . . . . . . . A . A . . . . A A A A A A A A A A . . . . . . . . . . . . . . . . . A A . A A . . . . A A A . A A A A A . . . . A A A . . A A    
504	. G G G G G G G G G G G G G G G G G G G G G G G G G G G G G G . G G G G G G G G G G G G G G G G G G G G G G G G G G G G G G G G G G G G G G . G G G G G G G G G G . . . . . . G G G G G G G G G G G G G G G . . G . G G G G G . . . . . . . . . . . G . G . . . . G G G G G G G G G G . . . . . . . . . . . . . . . . . G G . G G . . . . G G G . G G G G G . . . . G G G . . G G    
505	. C C C C C C C C C C C C C C C C C C C C C C C C C C C C C C . C C C C C C C C C C C C C C C C C C C C C C C C C C C C C C C C C C C C C C . C C C C C C C C C C . . . . . . C C C C C C C C C C C C C C C . . C . C C C C C . . . . . . . . . . . C . C . . . . C C C C C C C C C C . . . . . . . . . . . . . . . . . C C . C C . . . . C C C . C C C C C . . . . C C C . . C C    
506	. T T A T T T T T T T T T T T T T T T T T T T T T T T T T T T . T T T T T T T T T T T T T T T T T T T T T T T T T T T T T T T T T T T T T T . T T T T T T T T T T . . . . . . T T T T T T T T T T T T T T T . . T . T T T T T . . . . . . . . . . . T . T . . . . T T T T T T T T T T . . . . . . . . . . . . . . . . . T T . T T . . . . T T T T T T G T T . . . . T T T . . T T    
507	. G G G G G G G G G G G G G G G G G G G G G G G G G G G G G G . G G G G G G G G G G G G G G G G G G G G G G G G G G G G G G G G G G G G G G . G G G G G G G G G G . . . . . . G G G G G G G G G G G G G G G G G G . G G G G G . . . . . . . . . . . G . G . . . . G G G G G G G G G G . . . . . . . . . . . . . . . . . G G . G G . . . . G G G G G G G G G . . . . G G G . . G G    
508	. C C C C C C C C C C C C C C C C C C C C C C C C C C C C C C . C C C C C C C C C C C C C C C C C C C C C C C C C C C C C C C C C C C C C C . C C C C C C C C C C . . . . . . C C C C C C C C C C C C C C C C C C . C C C C C . . . . . . . . . . . C . C . . . . C C C C C C C C C C . . . . . . . . . . . . . . . . . C C . C C . . . . C C C C C C C C C . . . . C C C . . C C    
509	. A A A A A A A A A A A A A A A A A A A A A A A A A A A A A A . A A A A A A A A A A A A A A A A A A A A A A A A A A A A A A A A A A A A A A . A A A A A A A A A A . . . . . . A A A A A A A A A A A A A A A A A A . A A A A A . . . . . . . . . . . A . A . . . A A A A A A A A A A A . . . . . . . . . . . . . . . . . A A . A A . . . . A A A A A A A A A . . . . A A A . . A A    
510	. T T T T T T T T T T T T T T T T T T T T T T T T T T T T T T . T T T T T T T T T T T T T T T T T T T T T T T T T T T T T T T T T T T T T T . T T T T T T T T T T . . . . . . T T T T T T T T T T T T T T T T T T . T T T T T . . . . . . . . . . . T . T . . . T T T T T T T T T T T . . . . . . . . . . . . . . . . . T T . T T . . . . T T T T T T T T T . . . . T T T . . T T    
511	. G G G G G G G G G G G G G G G G G G G G G G G G G G G G G G . G G G G G G G G G G G G G G G G G G G G G G G G G G G G G G G G G G G G G G . G G G G G G G G G G . . . . . . G G G G G G G G G G G G G G G G G G . G G G G G . . . . . . . . . . . G . G . . . G G G G G G G G G G G . . . . . . . . . . . . . . . . . G G . G G . . . . G G G G G G G G G . . . . G G G . . G G    
512	. T T T T T T T T T T T T T T T T T T T T T T T T T T T T T T . T T T T T T T T T T T T T T T T T T T T T T T T T T T T T T T T T T T T T T . T T T T T T T T T T . . . . . . T T T T T T T T T T T T T T T T T T . T T T T T . . . . . . . . . . . T . T . . . T T T T T T T T T T T . . . . . . . . . . . . . . . . . T T . T T . . . . T T T T T T T T T . . . . T T T . . T T    
513	. G G G G G G G G G G G G G G G G G G G G G G G G G G G G G G . G G G G G G G G G G G G G G G G G G G G G G G G G G G G G G G G G G G G G G . G G G G G G G G G G . . . . . . G G G G G G G G G G G G G G G G G G . G G G G G . . . . . . . . . . . G . G . . . G G G G G G G G G G G . . . . . . . . . . . . . . . . . G G . G G . . . . G G G G G G G G G . . . . G G G . . G G    
514	. C C C C C C C C C C C C C C C C C C C C C C C C C C C C C C . C C C C C C C C C C C C C C C C C C C C C C C C C C C C C C C C C C C C C C . C C C C C C C C C C . . . . . . C C C C C C C C C C C C C C C C C C . C C C C C . . . . . . . . . . . C . C . . . C C C C C C C C C C C . . . . . . . . . . . . . . . . . C C . C C . . . . C C C C C C C C C . . . . C C C . . C C    
515	. C C C C C C C C C C C C C C C C C C C C C C C C C C C C C C . C C C C C C C C C C C C C C C C C C C C C C C C C C C C C C C C C C C C C C . C C C C C C C C C C . . . . . . C C C C C C C C C C C C C C C C C C . C C C C C . . . . . . . . . . . C . C . . . C C C C C C C C C C C . . . . . . . . . . . . . . . . . C C . C C . . . . C C C C C C C C C . . . . C C C . . C C    
516	. C C C C C C C C C C C C C C C C C C C C C C C C C C C C C C . C C C C C C C C C C C C C C C C C C C C C C C C C C C C C C C C C C C C C C . C C C C C C C C C C . . . . . . C C C C C C C C C C C C C C C C C C . C C C C C . . . . . . . . . . . C . C . . . C C C C C C C C C C C . . . . . . . . . . . . . . . . . C C . C C . . . . C C C C C C C C C . . . . C C C . . C C    
517	. A A A A A A A A A A A A A A A A A A A A A A A A A A A A A A . A A A A A A A A A A A A A A A A A A A A A A A A A A A A A A A A A A A A A A . A A A A A A A A A A . . . . . . A A A A A A A A A A A A A A A A A A . A A A A A . . . . . . . . . . . A . A . . . A A A A A A A A A A A . . . . . . . . . . . . . . . . . A A . A A . . . . A A A A A A A A A . . . . A A A . . A A    
518	. T T T T T T T T T T T T T T T T T T T T T T T T T T T T T T . T T T T T T T T T T T T T T T T T T T T T T T T T T T T T T T T T T T T T T . T T T T T T T T T T . . . . . . T T T T T T T T T T T T T T T T T T . T T T T T . . . . . . . . . . . T . T . . . T T T T T T T T T T T . . . . . . . . . . . . . . . . . T T . T T . . . . T T T T T T T T T . . . . T T T . . T T    
519	. G G G G G G G G G G G G G G G G G G G G G G G G G G G G G G . G G G G G G G G G G G G G G G G G G G G G G G G G G G G G G G G G G G G G G . G G G G G G G G G G . . . . . . G G G G G G G G G G G G G G G G G G . G G G G G . . . . . . . . . . . G . G . . . G G G G G G G G G G G . . . . . . . . . . . . . . . . . G G . G G . . . . G G G G G G G G G . . . . G G G . . G G    
520	. A A A A A A A A A A A A A A A A A A A A A A A A A A A A A A A A A A A A A A A A A A A A A A A A A A A A A A A A A A A A A A A A A A A A A . A A A A A A A A A A . . . . . . A A A A A A A A A A A A A A A A A A . A A A A A . . . . . . . . . . . A . A . . . A A A A A A A A A A A . . . . . . . . . . . . . . . . . A A . A A . . . . A A A A A A A A A . . . . A A A . . A A    
521	. A A A A A A A A A A A A A A A A A A A A A A A A A A A A A A A A A A A A A A A A A A A A A A A A A A A A A A A A A A A A A A A A A A A A A . A A A A A A A A A A . . . . . . A A A A A A A A A A A A A A A A A A A A A A A A . . . . . . . . . . . A . A . . . A A A A A A A A A A A . . . . . . . . . . . . . . . . . A A . A A . . . . A A A A A A A A A . . . . A A A . . A A    
522	. C C C C C C C C C C C C C C C C C C C C C C C C C C C C C C C C C C C C C C C C C C C C C C C C C C C C C C C C C C C C C C C C C C C C C . C C C C C C C C C C . . . . . . C C C C C C C C C C C C C C C C C C C C C C C C . . . . . . . . . . . C . C . . . C C C C C C C C C C C . . . . . . . . . . . . . . . . . C C . C C . . . . C C C C C C C C C . . . . C C C . . C C    
523	. A A A A A A A A A A A A A A A A A A A A A A A A A A A A A A A A A A A A A A A A A A A A A A A A A A A A A A A A A A A A A A A A A A A A A . A A A A A A A A A A . . . . . . A A A A A A A A A A A A A A A A A A A A A A A A . . . . . . . . . . . A . A . . . A A A A A A A A A A A . . . . . . . . . . . . . . . . . A A . A A . . . . A A A A A A A A A . . . . A A A . . A A    
524	. C C C C C C C C C C C C C C C C C C C C C C C C C C C C C C C C C C C C C C C C C C C C C C C C C C C C C C C C C C C C C C C C C C C C C . C C C C C C C C C C . . . . . . C C C C C C C C C C C C C C C C C C C C C C C C . . . . . . . . . . . C . C . . . C C C C C C C C C C C . . . . . . . . . . . . . . . . . C C . C C . . . . C C C C C C C C C . . . . C C C . . C C    
525	. C C C C C C C C C C C C C C C C C C C C C C C C C C C C C C C C C C C C C C C C C C C C C C C C C C C C C C C C C C C C C C C C C C C C C . C C C C C C C C C C . . . . . . C C C C C C C C C C C C C C C C C C C C C C C C . . . . . . . . . . . C . C . . . C C C C C C C C C C C . . . . . . . . . . . . . . . . . C C C C C . . . . C C C C C C C C C . . . . C C C . . C C    
526	. A A A A A A A A A A A A A A A A A A A A A A A A A A A A A A A A A A A A A A A A A A A A A A A A A A A A A A A A A A A A A A A A A A A A A . A A A A A A A A A A . . . . . . A A A A A A A A A A A A A A A A A A A A A A A A . . . . . . . . . . . A . A . . . A A A A A A A A A A A . . . . . . . . . . . . . . . . . A A A A A . . . . A A A A A A A A A . . . . A A A . . A A    
527	. T T T T T T T T T T T T T T T T T T T T T T T T T T T T T T T T T T T T T T T T T T T T T T T T T T T T T T T T T T T T T T T T T T T T T . T T T T T T T T T T . . . . . . T T T T T T T T T T T T T T T T T T T T T T T T . . . . . . . . . . . T . T . . . T T T T T T T T T T T . . . . . . . . . . . . . . . . . T T T T T . . . . T T T T T T T T T . . . . T T T . . T T    
528	. G G G G G G G G G G G G G G G G G G G G G G G G G G G G G G G G G G G G G G G G G G G G G G G G G G G G G G G G G G G G G G G G G G G G G . G G G G G G G G G G . . . . . . G G G G G G G G G G G G G G G G G G G G G G G G . . . . . . . . . . . G . G . . . G G G G G G G G G G G . . . . . . . . . . . . . . . . . G G G G G . . . . G G G G G G G G G . . . . A G G . . G G    
529	. T C C C C C C C C C C C C C C C C C C C C C C C C C C C C C C C C C C C C C C C C C C C C C C C C C C C C C C C C C C C C C C C C C C C C . C C C C C C C C C C . . . . . . C C C C C C C C C C C C C C C C C C C C C C C C . . . . . . . . . . . C . C . . . C C C C C C C C C C C . . . . . . . . . . . . . . . . . C C C C C . . . . C C C C C C C C C . . . . C C C . . C C    
530	. . C C C C C C C C C C C C C C C C C C C C C C C C C C C C C C C C C C C C C C C C C C C C C C C C C C C C C C C C C C C C C C C C C C C C . C C C C C C C C C C . . . . . . C C C C C C C C C C C C C C C C C C C C C C C C . . . . . . . . . . . C . C . . . C C C C C C C C C C C . . . . . . . . . . . . . . . . . C C C C C . . . . C C C C C C C C C . . . . C C C . . C C    
531	. . A A A A A A A A A A A A A A A A A A A A A A A A A A A A A A A A A A A A A A A A A A A A A A A A A A A A A A A A A A A A A A A A A A A A . A A A A A A A A A A . . . . . . A A A A A A A A A A A A A A A A A A A A A A A A . . . . . . . . . . . A . A . . . A A A A A A A A A A A . . . . . . . . . . . . . . . . . A A A A A . . . . A A A A A A A A A . . . . A A A . . A A    
532	. . T T T T T T T T T T T T T T T T T T T T T T T T T T T T T T T T T T T T T T T T T T T T T T T T T T T T T T T T T T T T T T T T T T T T . T T T T T T T T T T . . . . . . T T T T T T T T T T T T T T T T T T T T T T T T . . . . . . . . . . . T . T . . T T T T T T T T T T T T . . . . . . . . . . . . . . . . . T T T T T . . . . T T T T T T T T T . . . . T T T . . T T    
533	. . T T T T T T T T T T T T T T T T T T T T T T T T T T T T T T T T T T T T T T T T T T T T T T T T T T T T T T T T T T T T T T T T T T T T . T T T T T T T T T T . . . . . . T T T T T T T T T T T T T T T T T T T T T T T T . . . . . . . . . . . T . T . . T T T T T T T T T T T T . . . . . . . . . . . . . . . . . T T T T T . . . . T T T T T T T T T . . . . T T T . . T T    
534	. . G G G G G G G G G G G G G G G G G G G G G G G G G G G G G G G G G G G G G G G G G G G G G G G G G G G G G G G G G G G G G G G G G G G G . G G G G G G G G G G . . . . . . G G G G G G G G G G G G G G G G G G G G G G G G . . . . . . . . . . . G . G . . G G G G G G G G G G G G . . . . . . . . . . . . . . . . . G G G G G . . . . G G G G G G G G G . . . . G G G . . G G    
535	. . C C C C C C C C C C C C C C C C C C C C C C C C C C C C C C C C C C C C C C C C C C C C C C C C C C C C C C C C C C C C C C C C C C C C . C C C C C C C C C C . . . . . . C C C C C C C C C C C C C C C C C C C C C C C C . . . . . . . . . . . C . C . . C C C C C C C C C C C C . . . . . . . . . . . . . . . . . C C C C C . . . . C C C C C C C C C . . . . C C C . . C C    
536	. . T T T T T T T T T T T T T T T T T T T T T T T T T T T T T T T T T T T T T T T T T T T T T T T T T T T T T T T T T T T T T T T T T T T T . T T T T T T T T T T . . . . . . T T T T T T T T T T T T T T T T T T T T T T T T . . . . . . . . . . . T . T . . T T T T T T T T T T T T . . . . . . . . . . . . . . . . . T T T T T . . . . T T T T T T T T T . . . . T T T . . T T    
537	. . G G G G G G G G G G G G G G G G G G G G G G G G G G G G G G G G G G G G G G G G G G G G G G G G G G G G G G G G G G G G G G G G G G G G . G G G G G G G G G G . . . . . . G G G G G G G G G G G G G G G G G G G G G G G G . . . . . . . . . . . G . G . . G G G G G G G G G G G G . . . . . . . . . . . . . . . . . G G G G G . . . . G G G G G G G G G . . G . G G G . . G G    
538	. . A A A A A A A A A A A A A A A A A A A A A A A A A A A A A A A A A A A A A A A A A A A A A A A A A A A A A A A A A A A A A A A A A A A A . A A A A A A A A A A . . . . . . A A A A A A A A A A A A A A A A A A A A A A A A . . . . . . . . . . . A . A . . A A A A A A A A A A A A . . . . . . . . . . . . . . . . . A A A A A . . . . A A A A A A A A A . . A . A A A . . A A    
539	. . T T T T T T T T T T T T T T T T T T T T T T T T T T T T T T T T T T T T T T T T T T T T T T T T T T T T T T T T T T T T T T T T T T T T . T T T T T T T T T T . . . . . . T T T T T T T T T T T T T T T T T T T T T T T T . . . . . . . . . . . T . T . . T T T T T T T T T T T T . . . . . . . . . . . . . . . . . T T T T T . . . . T T T T T T T T T . . T . T T T . . T T    
540	. . C C C C C C C C C C C C C C C C C C C C C C C C C C C C C C C C C C C C C C C C C C C C C C C C C C C C C C C C C C C C C C C C C C C C . C C C C C C C C C C . . . . . . C C C C C C C C C C C C C C C C C C C C C C C C . . . . . . . . . . . C . C . . C C C C C C C C C C C C . . . . . . . . . . . . . . . . . C C C C C . . . . C C C C C C C C C . . C . C C C . . C C    
541	. . G G G G G G G G G G G G G G G G G G G G G G G G G G G G G G G G G G G G G G G G G G G G G G G G G G G G G G G G G G G G G G G G G G G G . G G G G G G G G G G . . . . . . G G G G G G G G G G G G G G G G G G G G G G G G . . . . . . . . . . . G . G . . G G G G G G G G G G G G . . . . . . . . . . . . . . . . . G G G G G . . . . G G G G G G G G G . . G . G G G . . G G    
542	. . A A A A A A A A A A A A A A A A A A A A A A A A A A A A A A A A A A A A A A A A A A A A A A A A A A A A A A A A A A A A A A A A A A A A . A A A A A A A A A A . . . . . . A A A A A A A A A A A A A A A A A A A A A A A A . . . . . . . . . . . A . A . A A A A A A A A A A A A A . . . . . . . . . . . . . . . . . A A A A A . . . . A A A A A A A A A . . A A A A A . . A A    
543	. . T T T T T T T T T T T T T T T T T T T T T T T T T T T T T T T T T T T T T T T T T T T T T T T T T T T T T T T T T T T T T T T T T T T T . T T T T T T T T T T . . . . . . T T T T T T T T T T T T T T T T T T T T T T T T . . . . . . . . . . . T . T . T T T T T T T T T T T T T . . . . . . . . . . . . . . . . . T T T T T . . . . T T T T T T T T T . . T T T T T . . T T    
544	. . G G G G G G G G G G G G G G G G G G G G G G G G G G G G G G G G G G G G G G G G G G G G G G G G G G G G G G G G G G G G G G G G G G G G . G G G G G G G G G G . . . . . . G G G G G G G G G G G G G G G G G G G G G G G G . . . . . . . . . . . G . G . G G G G G G G G G G G G G . . . . . . . . . . . . . . . . . G G G G G . . . . G G G G G G G G G . . G G G G G . . G G    
545	. . A A A A A A A A A A A A A A A A A A A A A A A A A A A A A A A A A A A A A A A A A A A A A A A A A A A A A A A A A A A A A A A A A A A A . A A A A A A A A A A . . . . . . A A A A A A A A A A A A A A A A A A A A A A A A . . . . . . . . . . . A . A . A A A A A A A A A A A A A . . . . . . . . . . . . . . . . . A A A A A . . . . A A A A A A A A A . . A A A A A . . A A    
546	. . G G G G G G G G G G G G G G G G G G G G G G G G G G G G G G G G G G G G G G G G G G G G G G G G G G G G G G G G G G G G G G G G G G G G . G G G G G G G G G G . . . . . . G G G G G G G G G G G G G G G G G G G G G G G G . . . . . . . . . . . G . G . G G G G G G G G G G G G G . . . . . . . . . . . . . . . . . G G G G G . . . . G G G G G G G G G . . G G G G G . . G G    
547	. . T T T T T T T T T T T T T T T T T T T T T T T T T T T T T T T T T T T T T T T T T T T T T T T T T T T T T T T T T T T T T T T T T T T T . T T T T T T T T T T . . . . . . T T T T T T T T T T T T T T T T T T T T T T T T . . . . . . . . . . . T . T . T T T T T T T T T T T T T . . . . . . . . . . . . . . . . . T T T T T . . . . T T T T T T T T T . . T T T T T . . T T    
548	. . C C C C C C C C C C C C C C C C C C C C C C C C C C C C C C C C C C C C C C C C C C C C C C C C C C C C C C C C C C C C C C C C C C C C . C C C C C C C C C C . . . . . . C C C C C C C C C C C C C C C C C C C C C C C C . . . . . . . . . . . C . C . C C C C C C C C C C C C C . . . . . . . . . . . . . . . . . C C C C C . . . . C C C C C C C C C . . C C C C C . . C C    
549	. . T T T T T T T T T T T T T T T T T T T T T T T T T T T T T T T T T T T T T T T T T T T T T T T T T T T T T T T T T T T T T T T T T T T T . T T T T T T T T T T . . . . . . T T T T T T T T T T T T T T T T T T T T T T T T . . . . . . . . . . . T . T . T T T T T T T T T T T T T . . . . . . . . . . . . . . . . . T T T T T . . . . T T T T T T T T T . . T T T T T . . T T    
550	. . G G G G G G G G G G G G G G G G G G G G G G G G G G G G G G G G G G G G G G G G G G G G G G G G G G G G G G G G G G G G G G G G G G G G . G G G G G G G G G G . . . . . . G G G G G G G G G G G G G G G G G G G G G G G G . . . . . . . . . . . G . G . G G G G G G G G G G G G G . . . . . . . . . . . . . . . . . G G G G G . . . . G G G G G G G G G . . G G G G G . . G G    
551	. . C C C C C C C C C C C C C C C C C C C C C C C C C C C C C C C C C C C C C C C C C C C C C C C C C C C C C C C C C C C C C C C C C C C C . C C C C C C C C C C . . . . . . C C C C C C C C C C C C C C C C C C C C C C C C . . . . . . . . . . . C . C . C C C C C C C C C C C C C . . . . . . . . . . . . . . . . . C C C C C . . . . C C C C C C C C C . . C C C C C . . C C    
552	. . T T T T T T T T T T T T T T T T T T T T T T T T T T T T T T T T T T T T T T T T T T T T T T T T T T T T T T T T T T T T T T T T T T T T . T T T T T T T T T T . . . . . . T T T T T T T T T T T T T T T T T T T T T T T T . . . . . . . . . . . T . T . T T T T T T T T T T T T T . . . . . . . . . . . . . . . . . T T T T T . . . . T T T T T T T T T . . T T T T T . . T T    
553	. . G G G G G G G G G G G G G G G G G G G G G G G G G G G G G G G G G G G G G G G G G G G G G G G G G G G G G G G G G G G G G G G G G G G G . G G G G G G G G G G . . . . . . G G G G G G G G G G G G G G G G G G G G G G G G . . . . . . . . . . . G . G . G G G G G G G G G G G G G . . . . . . . . . . . . G . . . . G G G G G . . . . G G G G G G G G G G G G G G G G . . G G    
554	. . A A A A A A A A A A A A A A A A A A A A A A A A A A A A A A A A A A A A A A A A A A A A A A A A A A A A A A A A A A A A A A A A A A A A . A A A A A A A A A A . . . . . . A A A A A A A A A A A A A A A A A A A A A A A A . . . . . . . . . . . A . A . A A A A A A A A A A A A A . . . . . . . . . . . . A . . . . A A A A A . . . . A A A A A A A A A A A A A A A A . . A A    
555	. . T T T T T T T T T T T T T T T T T T T T T T T T T T T T T T T T T T T T T T T T T T T T T T T T T T T T T T T T T T T T T T T T T T T T . T T T T T T T T T T . . . . . . T T T T T T T T T T T T T T T T T T T T T T T T . . . . . . . . . . . T . T . T T T T T T T T T T T T T . . . . . . . . . T . . T . . . . T T T T T . . . . T T T T T T T T T T T T T T T T . . T T    
556	. . G G G G G G G G G G G G G G G G G G G G G G G G G G G G G G G G G G G G G G G G G G G G G G G G G G G G G G G G G G G G G G G G G G G G . G G G G G G G G G G . . . . . . G G G G G G G G G G G G G G G G G G G G G G G G . . . . . . . . . . . G . G . G G G G G G G G G G G G G . . . . . . . . . G . . G . . . . G G G G G . . . . G G G G G G G G G G G G G G G G . . G G    
557	. . A A A A A A A A A A A A A A A A A A A A A A A A A A A A A A A A A A A A A A G A A A A A A A A A A A A A A A A A A A A A A A A A A A A A . A A A A A A A A A A . . . . . . A A A A A A A A A A A A A A A A A A A A A A A A . . . . . . . . . . . A . A . A A A A A A A A A A A A A . . . . . . . . . A . . A . . . . A A A A A . . . . A A A A A A G A A A A A A A A A . . A A    
558	. . G G G G G G G G G G G G G G G G G G G G G G G G G G G G G G G G G G G G G G G G G G G G G G G G G G G G G G G G G G G G G G G G G G G G . G G G G G G G G G G . . . . . . G G G G G G G G G G G G G G G G G G G G G G G G . . . . . . . . . . . G . G . G G G G G G G G G G G G G . . . . . . . . . G . . G . . . . G G G G G . . . . G G G G G G G G G G G G G G G G . . G G    
559	. . G G G G G G G G G G G G G G G G G G G G G G G G G G G G G G G G G G G G G G G G G G G G G G G G G G G G G G G G G G G G G G G G G G G G . G G G G G G G G G G . . . . . . G G G G G G G G G G G G G G G G G G G G G G G G . . . . . . . . . . . G . G . G G G G G G G G G G G G G . . . . . . . . . G . . G . . . . G G G G G . . . . G G G G G G G G G G G G G G G G . . G G    
560	. . G G G G G G G G G G G G G G G G G G G G G G G G G G G G G G G G G G G G G G G G G G G G G G G G G G G G G G G G G G G G G G G G G G G G . G G G G G G G G G G . . . . . . G G G G G G G G G G G G G G G G G G G G G G G G . . . . . . . . . . . G . G . G G G G G G G G G G G G G . . . . . . . . . G . . G . . . . G G G G G . . . . G G G G G G G G G G G G G G G G . . G G    
561	. . G G G G G G G G G G G G G G G G G G G G G G G G G G G G G G G G G G G G G G G G G G G G G G G G G G G G G G G G G G G G G G G G G G G G . G G G G G G G G G G . . . . . . G G G G G G G G G G G G G G G G G G G G G G G G . . . . . . . . . . . G . G . G G G G G G G G G G G G G . . . . . . . . . G . . G . . . . G G G G G . . . . G G G G G G G G G G G G G G G G . . G G    
562	. . A A A A A A A A A A A A A A A A A A A A A A A A A A A A A A A A A A A A A A A A A A A A A A A A A A A A A A A A A A A A A A A A A A A A . A A A A A A A A A A . . . . . . A A A A A A A A A A A A A A A A A A A A A A A A . . . . . . . . . . . A . A . A A A A A A A A A A A A A . . . . . . . . . A A . A . . . . A A A A A . . . . A A A A A A A A A A A A A A A A . . A A    
563	. . C C C C C C C C C C C C C C C C C C C C C C C C C C C C C C C C C C C C C C C C C C C C C C C C C C C C C C C C C C C C C C C C C C C C . C C C C C C C C C C . C . C . C C C C C C C C C C C C C C C C C C C C C C C C C . . . . . . . . . . . C . C . C C C C C C C C C C C C C . . . . . . . . . C C . C . . . . C C C C C . . . . C C C C C C C C C C C C C C C C . . C C    
564	. . A A A A A A A A A A A A A A A A A A A A A A A A A A A A A A A A A A A A A A A A A A A A A A A A A A A A A A A A A A A A A A A A A A A A . A A A A A A A A A A . A . A . A A A A A A A A A A A A A A A A A A A A A A A A A . . . . . . . . . . . A . A . A A A A A A A A A A A A A . . . . . . . . . A A . A . . . . A A A A A . . . . A A A A A A A A A A A A A A A A . . A A    
565	. . T T T T T T T T T T T T T T T T T T T T T T T T T T T T T T T T T T T T T T T T T T T T T T T T T T T T T T T T T T T T T T T T T T T T . T T T T T T T T T T . T . T . T T T T T T T T T T T T T T T T T T T T T T T T T . . . . . . . . . . . T . T . T T T T T T T T T T T T T . . . . . . . . . T T . T . . . . T T T T T . . . . T T T T T T T T T T T T T T T T . . T T    
566	. . C C C C C C C C C C C C C C C C C C C C C C C C C C C C C C C C C C C C C C C C C C C C C C C C C C C C C C C C C C C C C C C C C C C C . C C C C C C C C C C . C . C . C C C C C C C C C C C C C C C C C C C C C C C C C . . . . . . . . . . . C . C . C C C C C C C C C C C C C . . . . . . . . . C C . C . . . . C C C C C . . . . C C C C C C C C C C C C C C C C . . C C    
567	. . T T T T T T T T T T T T T T T T T T T T T T T T T T T T T T T T T T T T T T T T T T T T T T T T T T T T T T T T T T T T T T T T T T T C . T T T T T T T T T T . T . T . T T T T T T T T T T T T T T T T T T T T T T T T T . . . . . . . . . . . T . T . T T T T T T T T T T T T T . . . . . . . . . T T . T . . . . T T T T T . . . . T T T T T C T T T T T T T T T T . . T T    
568	. . A A A A A A A A A A A A A A A A A A A A A A A A A A A A A A A A A A A A A A A A A A A A A A A A A A A A A A A A A A A A A A A A A A A A . A A A A A A A A A A . A . A . A A A A A A A A A A A A A A A A A A A A A A A A A . . . . . . . . . . . A . A . A A A A A A A A A A A A A . . . . . . . . . A A . A . . . . A A A A A . . . . A A A A A A A A A A A A A A A A . . A A    
569	. . C C C C C C C C C C C C C C C C C C C C C C C C C C C C C C C C C C C C C C C C C C C C C C C C C C C C C C C C C C C C C C C C C C C C . C C C C C C C C C C . C . C . C C C C C C C C C C C C C C C C C C C C C C C C C . . . . . . . . . . . C . C . C C C C C C C C C C C C C . . . . . . . . . C C . C . . . . C C C C C . . . . C C C C C C C C C C C C C C C C . . C C    
570	. . C C C C C C C C C C C C C C C C C C C C C C C C C C C C C C C C C C C C C C C C C C C C C C C C C C C C C C C C C C C C C C C C C C C C . C C C C C C C C C C . C . C . C T C C C C C C C C C C C C C C C C C C C C C C C . . . . . . . . . . . C . C . C C C C C C C C C C C C C . . . . . . . . . C C . C . . . . C C C C C . . . . C C C C C C C C C C C C C C C C . . C C    
571	. . C C C C C C C C C C C C C C C C C C C C C C C C C C C C C C C C C C C C C C C C C C C C C C C C C C C C C C C C C C C C C C C C C C C C . C C C C C C C C C C . C . C . C C C C C C C C C C C C C C C C C C C C C C C C C . . . . . . . . . . . C . C . C C C C C C C C C C C C C . . . . . . . . . C C . C . . . . C C C C C . . . . C C C C C C C C C C C C C C C C . . C C    
572	. . A A A A A A A A A A A A A A A A A A A A A A A A A A A A A G A A A A A A A A A A A A A A A A A A A A A A A A A A A A A A A A A A A A A A . A A A A A A A A A A . A . A . A A A A A A A A A A A A A A A A A A A A A A A A A . . . . . . . . . . . A . A . A A A A A A A A A A A A A . . . . . . . . . A A . A . . . . A A A A A . . . . A A A A A A A A A A A A A A A A . . A A    
573	. . A A A A A A A A A A A A A A A A A A A A A A A A A A A A A A A A A A A A A A A A A A A A A A A A A A A A A A A A A A A A A A A A A A A A . A A A A A A A A A A . A . A . A A A A A A A A A A A A A A A A A A A A A A A A A . . . . . . . . . . . A . A . A A A A A A A A A A A A A . . . . . . . . . A A . A . . . . A A A A A . . . . A A A A A A A A A A A A A A A A . . A A    
574	. . G G G G G G G G G G G G G G G G G G G G G G G G G G G G G G G G G G G G G G G G G G G G G G G G G G G G G G G G G G G G G G G G G G G G . G G G G G G G G G G . G . G . G G G G G G G G G G G G G G G G G G G G G G G G G . . . . . . . . . . . G . G . G G G G G G G G G G G G G . . G G . . . . . G G . G . . . . G G G G G . . . . G G G G G G G G G G G G G G G G . . G G    
575	. . G G G G G G G G G G G G G G G G G G G G G G G G G G G G G G G G G G G G G G G G G G G G G G G G G G G G G G G G G G G G G G G G G G G G . G G G G G G G G G G . G . G . G G G G G G G G G G G G G G G G G G G G G G G G G . . . . . . . . . . . G . G . G G G G G G G G G G G G G . . G G . . . . . G G . G . . . . G G G G G . . . . G G G G G G G G G G G G G G G G . . G G    
576	. . G G G G G G G G G G G G G G G G G G G G G G G G G G G G G G G G G G G G G G G G G G G G G G G G G G G G G G G G G G G G G G G G G G G G . G G G G G G G G G G . G . G . G G G G G G G G G G G G G G G G G G G G G G G G G . . . . . . . . . . . G . G . G G G G G G G G G G G G G . . G G . . . . . G G . G . . . . G G G G G . . . . G G G G G G G G G G G G G G G G . . G G    
577	. . G C C C C C C C C C C C C C C C C C C C C C C C C C C C C C C C C C C C C C C C C C C C C C C C C C C C C C C C C C C C C C C C C C C C . C C C C C C C C C C . C . C . C C C C C C C C C C C C C C C C C C C C C C C C C . . . . . . . . . . . C . C . C C C C C C C C C C C C C . . C C . . . . . C C . C . . . . C C C C C . . . . C C C C C C C C C C C C C C C C . . C C    
578	. . C C C C C C C C C C C C C C C C C C C C C C C C C C C C C C C C C C C C C C C C C C C C C C C C C C C C C C C C C C C C C C C C C C C C . C C C C C C C C C C . C . C . C C C C C C C C C C C C C C C C C C C C C C C C C . . . . . . . . . C C C . C . C C C C C C C C C C C C C . . C C . . . . . C C . C . . . . C C C C C . . . . C C C C C C C C C C C C C C C C . . C C    
579	. . C A A A A A A A A A A A A A A A A A A A A A A A A A A A A A A A A A A A A A A A A A A A A A A A A A A A A A A A A A A A A A A A A A A A . A A A A A A A A A A . A . A . A A A A A A A A A A A A A A A A A A A A A A A A A . . . . . . . . . C A A . A . A A A A A A A A A A A A A . . A A . . . . . A A . A . . . . A A A A A . . . . A A A A A A A A A A A A A A A A . . A A    
580	. . A A A A A A A A A A A A A A A A A A A A A A A A A A A A A A A A A A A A A A A A A A A A A A A A A A A A A A A A A A A A A A A A A A A A A A A A A A A A A A A . A . A . A A A A A A A A A A A A A A A A A A A A A A A A A . . . . . . . . . A A A . A . A A A A A A A A A A A A A . . A A . . . . . A A . A . . . . A A A A A . . . . A A A A A A A A A A A A A A A A . . A A    
581	. . A A A A A A A A A A A A A A A A A A A A A A A A A A A A A A A A A A A A A A A A A A A A A A A A A A A A A A A A A A A A A A A A A A A A A A A A A A A A A A A . A . A . A A A A A A A A A A A A A A A A A A A A A A A A A . . . . . . . . . A A A . A . A A A A A A A A A A A A A . . A A . . . . . A A . A . . . . A A A A A . . . . A A A A A A A A A A A A A A A A . . A A    
582	. . G G G G G G G G G G G G G G G G G G G G G G G G G G G G G G G G G G G G G G G G G G G G G G G G G G G G G G G G G G G G G G G G G G G G G G G G G G G G G G G G G . G . G G G G G G G G G G G G G G G G G G G G G G G G G . . . . . . . . G G G G . G . G G G G G G G G G G G G G G G G G . . . . . G G . G . . . . G G G G G . . . . G G G G G G G G G G G G G G G G . . G G    
583	. . G G G G G G G G G G G G G G G G G G G G G G G G G G G G G G G G G G G G G G G G G G G G G G G G G G G G G G G G G G G G G G G G G G G G G G G G G G G G G G G G G . G . G C G G G G G G G G G G G G G G G G G G G G G G G . . . . . . . . G G G G . G . G G G G G G G G G G G G G G G G G . . . . . G G . G . . . . G G G G G . . . . G G G G G G G G G G G G G G G G . . G G    
584	. . A A A A A A A A A A A A A A A A A A A A A A A A A A A A A A A A A A A A A A A A A A A A A A A A A A A A A A A A A A A A A A A A A A A A A A A A A A A A A A A A A . A . A A A A A A A A A A A A A A A A A A A A A A A A A . . . . . . . . A A A A . A . A A A A A A A A A A A A A A A A A . . . . . A A . A . . . . A A A A A . . . . A A A A A A A A A A A A A A A A . . A A    
585	. . A A A A A A A A A A A A A A A A A A A A A A A A A A A A A A A A A A A A A A A A A A A A A A A A A A A A A A A A A A A A A A A A A A A A A A A A A A A A A A A A A . A . A A A A A A A A A A A A A A A A A A A A A A A A A . . . . . . . . A A A A . A . A A A A A A A A A A A A A A A A A . . . . . A A . A . . . . A A A A A . . . . A A A A A A A A A A A A A A A A . . A A    
586	. . G G G G G G G G G G G G G G G G G G G G G G G G G G G G G G G G G G G G G G G G G G G G G G G G G G G G G G G G G G G G G G G G G G G G G G G G G G G G G G G G G . G . G G G G G G G G G G G G G G G G G G G G G G G G G . . . . . . . . G G G G . G . G G G G G G G G G G G G G G G G G . . . . . G G . G . . . . G G G G G . . . . G G G G G G G G G G G G G G G G . . G G    
587	. . G G G G G G G G G G G G G G G G G G G G G G G G G G G G G G G G G G G G G G G G G G G G G G G G G G G G G G G G G G G G G G G G G G G G G G G G G G G G G G G G G . G . G G G G G G G G G G G G G G G G G G G G G G G G G . . . . . . . . G G G G . G . G G G G G G G G G G G G G G G G G . . . . . G G . G . . . . G G G G G . . . . G G G G G G G G G G G G G G G G . . G G    
588	. . T T T T T T T T T T T T T T T T T T T T T T T T T T T T T T T T T T T T T T T T T T T T T T T T T T T T T T T T T T T T T T T T T T T T T T T T T T T T T T T T T . T . T T T T T T T T T T T T T T T T T T T T T T T T T . . . . . . . . T T T T . T . T T T T T T T T T T T T T T T T T . . . . . T T . T . . . . T T T T T . . . . T T T T T T T T T T T T T T T T . . T T    
589	. . T T T T T T T T T T T T T T T T T T T T T T T T T T T T T T T T T T T T T T T T T T T T T T T T T T T T T T T T T T T T T T T T T T T T T T T T T T T T T T T T T . T . T T T T T T T T T T T T T T T T T T T T T T T T T . . . . . . . . T T T T . T . T T T T T T T T T T T T T T T T T . . . . . T T . T . . . . T T T T T . . . . T T T T T T T T T T T T T T T T . . T T    
590	. . C C C C C C C C C C C C C C C C C C C C C C C C C C C C C C C C C C C C C C C C C C C C C C C C C C C C C C C C C C C C C C C C C C C C C C C C C C C C C C C C C . C . C A C C C C C C C C C C C C C C C C C C C C C C C . . . . . . . . C C C C . C . C C C C C C C C C C C C C C C C C . . . . C C C . C . . . . C C C C C . . . . C C C C C C C C C C C C C C C C . . C C    
591	. . T T T T T T T T T T T T T T T T T T T T T T T T T T T T T T T T T T T T T T T T T T T T T T T T T T T T T T T T T T T T T T T T T T T T T T T T T T T T T T T T T . T . T T T T T T T T T T T T T T T T T T T T T T T T T . . . . . . . . T T T T . T . T T T T T T T T T T T T T T T T T . . . . T T T . T . . . . T T T T T . . . . T T T T T T T T T T T T T T T T . . T T    
592	. . G G G G G G G G G G G G G G G G G G G G G G G G G G G G G G G G G G G G G G G G G G G G G G G G G G G G G G G G G G G G G G G G G G G G G G G G G G G G G G G G G . G . G G G G G G G G G G G G G G G G G G G G G G G G G . . . . . . . . G G G G . G . G G G G G G G G G G G G G G G G G . . . . G G G . G . . . . G G G G G . . . . G G G G G G G G G G G G G G G G . . G G    
593	. . C C C C C C C C C C C C C C C C C C C C C C C C C C C C C C C C C C C C C C C C C C C C C C C C C C C C C C C C C C C C C C C C C C C C C C C C C C C C C C C C C . C . C C C C C C C C C C C C C C C C C C C C C C C C C . . . . . . . . C C C C . C . C C C C C C C C C C C C C C C C C . . . . C C C . C . . . . C C C C C . . . . C C C C C C C C C C C C C C C C . . C C    
594	. . C C C C C C C C C C C C C C C C C C C C C C C C C C C C C C C C C C C C C C C C C C C C C C C C C C C C C C C C C C C C C C C C C C C C C C C C C C C C C C C C C . C . C C C C C C C C C C C C C C C C C C C C C C C C C . . . . . . . . C C C C . C . C C C C C C C C C C C C C C C C C . . . . C C C . C . . . . C C C C C . . . . C C C C C C C C C C C C C C C C . . C C    
595	. . A A A A A A A A A A A A A A A A A A A A A A A A A A A A A A A A A A A A A A A A A A A A A A A A A A A A A A A A A A A A A A A A A A A A A A A A A A A A A A A A A . A . A A A A A A A A A A A A A A A A A A A A A A A A A . . . . . . . . A A A A . A . A A A A A A A A A A A A A A A A A . . . . A A A . A . . . . A A A A A . . . . A A A A A A A A A A A A A A A A . . A A    
596	. . C C C C C C C C C C C C C C C C C C C C C C C C C C C C C C C C C C C C C C C C C C C C C C C C C C C C C C C C C C C C C C C C C C C C C C C C C C C C C C C C C . C . C C C C C C C C C C C C C C C C C C C C C C C C C . . . . . . . . C C C C . C . C C C C C C C C C C C C C C C C C . . . . C C C C C . . . . C C C C C . . . . C C C C C C C C C C C C C C C C . . C C    
597	. . C C C C C C C C C C C C C C C C C C C C C C C C C C C C C C C C C C C C C C C C C C C C C C C C C C C C C C C C C C C C C C C C C C C C C C C C C C C C C C C C C . C . C C C C C C C C C C C C C C C C C C C C C C C C C . . . . . . . . C C C C . C . C C C C C C C C C C C C C C C C C . . . . C C C C C . . . . C C C C C . . . . C C C C C C C C C C C C C C C C . . C C    
598	. . C C C C C C C C C C C C C C C C C C C C C C C C C C C C C C C C C C C C C C C C C C C C C C C C C C C C C C C C C C C C C C C C C C C C C C C C C C C C C C C C C . C . C C C C C C C C C C C C C C C C C C C C C C C C C . . . . . . . . C C C C . C . C C C C C C C C C C C C C C C C C . . . . C C C C C . . . . C C C C C . . . . C C C C C C C C C C C C C C C C . . C C    
599	. . A A A A A A A A A A A A A A A A A A A A A A A A A A A A A A A A A A A A A A A A A A A A A A A A A A A A A A A A A A A A A A A A A A A A A A A A A A A A A A A A A . A . A A A A A A A A A A A A A A A A A A A A A A A A A . . . . . . . . A A A A . A . A A A A A A A A A A A A A A A A A . . . . A A A A A . . . . A A A A A . . . . A A A A A A A A A A A A A A A A . . A A    
600	. . G G G G G G G G G G G G G G G G G G G G G G G G G G G G G G G G G G G G G G G G G G G G G G G G G G G G G G G G G G G G G G G G G G G G G G G G G G G G G G G G G . G . G G G G G G G G G G G G G G G G G G G G G G G G G . . . . . . . . G G G G . G . G G G G G G G G G G G G G G G G G . . . . G G G G G . . . . G G G G G . . . . G G G G G G G G G G G G G G G G . . G G    
601	. . T T T T T T T T T T T T T T T T T T T T T T T T T T T T T T T T T T T T T T T T T T T T T T T T T T T T T T T T T T T T T T T T T T T T T T T T T T T T T T T T T . T . T T T T T T T T T T T T T T T T T T T T T T T T T . . . . . . . . T T T T . T . T T T T T T T T T T T T T T T T T . . . . T T T T T . . . . T T T T T . . . . T T T T T T T T T T T T T T T T . . T T    
602	. . G G G G G G G G G G G G G G G G G G G G G G G G G G G G G G G G G G G G G G G G G G G G G G G G G G G G G G G G G G G G G G G G G G G G G G G G G G G G G G G G G . G . G G G G G G G G G G G G G G G G G G G G G G G G G . . . . . . . . G G G G . G . G G G G G G G G G G G G G G G G G . . . . G G G G G . . . . G G G G G . . . G G G G G G G G G G G G G G G G G . . G G    
603	. . T T T T T T T T T T T T T T T T T T T T T T T T T T T T T T T T T T T T T T T T T T T T T T T T T T T T T T T T T T T T T T T T T T T T T T T T T T T T T T T T T . T . T T T T T T T T T T T T T T T T T T T T T T T T T . . . . . . . . T T T T . T . T T T T T T T T T T T T T T T T T . . . . T T T T T . . . . T T T T T . . . T T T T T T T T T T T T T T T T T . . T T    
604	. . C C C C C C C C C C C C C C C C C C C C C C C C C C C C C C C C C C C C C C C C C C C C C C C C C C C C C C C C C C C C C C C C C C C C C C C C C C C C C C C C C . C . C C C C C C C C C C C C C C C C C C C C C C C C C . . . . . . . . C C C C . C . C C C C C C C C C C C C C C C C C . . . . C C C C C . . . . C C C C C . . . C C C C C C C C C C C C C C C C C . . C C    
605	. . A A A A A A A A A A A A A A A A A A A A A A A A A A A A A A A A A A A A A A A A A A A A A A A A A A A A A A A A A A A A A A A A A A A A A A A A A A A A A A A A A . A . A A A A A A A A A A A A A A A A A A A A A A A A A . . . . . . . . A A A A . A . A A A A A A A A A A A A A A A A A . . . . A A A A A . . . . A A A A A . . . A A A A A A A A A A A A A A A A A . . A A    
606	. . G G G G G G G G G G G G G G G G G G G G G G G G G G G G G G G G G G G G G G G G G G G G G G G G G G G G G G G G G G G G G G G G G G G G G G G G G G G G G G G G G . G . G G G G G G G G G G G G G G G G G G G G G G G G G . . . . . . . . G G G G . G . G G G G G G G G G G G G G G G G G . . . . G G G G G . . . . G G G G G . . . G G G G G G G G G G G G G G G G G . . G G    
607	. . C C C C C C C C C C C C C C C C C C C C C C C C C C C C C C C C C C C C C C C C C C C C C C C C C C C C C C C C C C C C C C C C C C C C C C C C C C C C C C C C C . C . C C C C C C C C C C C C C C C C C C C C C C C C C . . . . . . . . C C C C . C . C C C C C C C C C C C C C C C C C . . . . C C C C C . . . . C C C C C . . . C C C C C C C C C C C C C C C C C . . C C    
608	. . T T T T T T T T T T T T T T T T T T T T T T T T T T T T T T T T T T T T T T T T T T T T T T T T T T T T T T T T T T T T T T T T T T T T T T T T T T T T T T T T T . T . T T T T T T T T T T T T T T T T T T T T T T T T T . . . . . . . . T T T T . T . T T T T T T T T T T T T T T T T T . . . . T T T T T . . . . T T T T T . . . T T T T T T T T T T T T T T T T T . . T T    
609	. . G G G G G G G G G G G G G G G G G G G G G G G G G G G G G G G G G G G G G G G G G G G G G G G G G G G G G G G G G G G G G G G G G G G G G G G G G G G G G G G G G . G . G G G G G G G G G G G G G G G G G G G G G G G G G . . . . . . . . G G G G . G . G G G G G G G G G G G G G G G G G . . . . G G G G G . . . . G G G G G . . . G G G G G G G G G G G G G G G G G . . G G    
610	. . G G G G G G G G G G G G G G G G G G G C G G G G G G G G G G G G G G G G G G G G G G G G G G G G G G G G G G G G G G G G G G G G G G G G G G G G G G G G G G G G G . G . G G G G G G G G G G G G G G G G G G G G G G G G G . . . . . . . . G G G G . G . G G G G G G G G G G G G G G G G G . . . . G G G G G . . . . G G G G G . . . G G G G G G G G G G G G G G G G G . . G G    
611	. . A A A A A A A A A A A A A A A A A A A A A A A A A A A A A A A A A A A A A A A A A A A A A A A A A A A A A A A A A A A A A A A A A A A A A A A A A A A A A A A A A . A . A A A A A A A A A A A A A A A A A A A A A A A A A . . . . . . . . A A A A . A . A A A A A A A A A A A A A A A A A . . . . A A A A A . . . . A A A A A . . . A A A A A A A A A A A A A A A A A . . A A    
612	. . G G G G G G G G G G G G G G G G G G G G G G G G G G G G G G G G G G G G G G G G G G G G G G G G G G G G G G G G G G G G G G G G G G G G G G G G G G G G G G G G G . G . G G G G G G G G G G G G G G G G A A G G G G G G G . . . . . . . . G G G G . G . G G G G G G G G G G G G G G G G G . . . . G G G G G . . . . G G G G G . . . G G G G G G G G G G G G G G G G G . . G G    
613	. . T T T T T T T T T T T T T T T T T T T T T T T T T T T T T T T T T T T T T T T T T T T T T T T T T T T T T T T T T T T T T T T T T T T T T T T T T T T T T T T T T . T . T T T T T T T T T T T T T T T T T T T T T T T T T . . . . . . . . T T T T . T . T T T T T T T T T T T T T T T T T . . . . T T T T T . . . . T T T T T . . . T T T T T T T T T T T T T T T T T . . T T    
614	. . C C C C C C C C C C C C C C C C C C C C C C C C C C C C C C C C C C C C C C C C C C C C C C C C C C C C C C C C C C C C C C C C C C C C C C C C C C C C C C C C C . C . C C C C C C C C C C C C C C C C C C C C C C C C C . . . . . . C C C C C C . C . C C C C C C C C C C C C C C C C C . . . . C C C C C . . . . C C C C C . . . C C C C C C C C C C C C C C C C C . . C C    
615	. . A A A A A A A A A A A A A A A A A A A A A A A A A A A A A A A A A A A A A A A A A A A A A A A A A A A A A A A A A A A A A A A A A A A A A A A A A A A A A A A A A . A . A A A A A A A A A A A A A A A A A A A A A A A A A . . . . . A A A A A A A . A . A A A A A A A A A A A A A A A A A . . . . A A A A A . . . . A A A A A . . . A A A A A A A A A A A A A A A A A . . A A    
616	. . A A A A A A A A A A A A A A A A A A A A A A A A A A A A A A A A A A A A A A A A A A A A A A A A A A A A A A A A A A A A A A A A A A A A A A A A A A A A A A A A A . A . A A A A A A A A A A A A A A A A A A A A A A A A A . . . . . A A A A A A A . A . A A A A A A A A A A A A A A A A A . . . . A A A A A . . . . A A A A A . . . A A A A A A A A A A A A A A A A A . . A A    
617	. . C C C C C C C C C C C C C C C C C C C C C C C C C C C C C C C C C C C C C C C C C C C C C C C C C C C C C C C C C C C C C C C C C C C C C C C C C C C C C C C C C . C . C C C C C C C C C C C C C C C C C C C C C C C C C . . . . . C C C C C C C . C . C C C C C C C C C C C C C C C C C . . . . C C C C C . . . . C C C C C . . . C C C C C C C C C C C C C C C C C . . C C    
618	. . A . A A A A A A A A A A A A A A A A A A A A A A A A A A A A A A A A A A A A A A A A A A A A A A A A A A A A A A A A A A A A A A A A A A A A A A A A A A A A A A A . A . A A A A A A A A A A A A A A A A A A A A A A A A A . . . . . A A A A A A A . A . A A A A A A A A A A A A A A A A A . . . . A A A A A . . . . A A A A A . . . A A A A A A A A A A A A A A A A A . . A A    
619	. . G . G G G G G G G G G G G G G G G G G G G G G G G G G G G G G G G G G G G G G G G G G G G G G G G G G G G G G G G G G G G G G G G G G G G G G G G G G G G G G G G . G . G G G G G G G G G G G G G G G G G G G G G G G G G . . . . . G G G G G G G . G . G G G G G G G G G G G G G G G G G . . . . G G G G G . . . . G G G G G . . . G G G G G G G G G G G G G G G G G . . G G    
620	. . G . G G G G G G G G G G G G G G G G G G G G G G G G G G G G G G G G G G G G G G G G G G G G G G G G G G G G G G G G G G G G G G G G G G G G G G G G G G G G G G G . G . G G G G G G G G G G G G G G G G G G G G G G G G G . . . . . G G G G G G G . G . G G G G G G G G G G G G G G G G G . . . . G G G G G . . . . G G G G G . . . G G G G G G G G G G G G G G G G G . . G G    
621	. . G . G G G G G G G G G G G G G G G G G G G G G G G G G G G G G G G G G G G G G G G G G G G G G G G G G G G G G G G G G G G G G G G G G G G G G G G G G G G G G G G . G . G G G G G G G G G G G G G G G G G G G G G G G G G . . . . . G G G G G G G . G . G G G G G G G G G G G G G G G G G . . . . G G G G G . . . . G G G G G . . . G G G G G G G G G G G G G G G G G . . G G    
622	. . T . T T T T T T T T T T T T T T T T T T T T T T T T T T T T T T T T T T T T T T T T T T T T T T T T T T T T T T T T T T T T T T T T T T T T T T T T T T T T T T T . T . T T T T T T T T T T T T T T T T T T T T T T T T T . . . . . T T T T T T T . T . T T T T T T T T T T T T T T T T T . . . . T T T T T . . . . T T T T T . . . T T T T T T T T T T T T T T T T T . . T T    
623	. . T . T T T T T T T T T T T T T T T T T T T T T T T T T T T T T T T T T T T T T T T T T T T T T T T T T T T T T T T T T T T T T T T T T T T T T T T T T T T T T T T . T . T T T T T T T T T T T T T T T T T T T T T T T T T . . . . . T T T T T T T . T . T T T T T T T T T T T T T T T T T . . . . T T T T T . . . . T T T T T . . . T T T T T T T T T T T T T T T T T . . T T    
624	. . G . G G G G G G G G G G G G G G G G G G G G G G G G G G G G G G G G G G G G G G G G G G G G G G G G G G G G G G G G G G G G G G G G G G G G G G G G G G G G G G G . G . G G G G G G G G G G G G G G G G G G G G G G G G G . . . . . G G G G G G G . G . G G G G G G G G G G G G G G G G G . . . . G G G G G . . . . G G G G G . . . G G G G G G G G G G G G G G G G G . . G G    
625	. . A . A A A A A A A A A A A A A A A A A A A A A A A A A A A A A A A A A A A A A A A A A A A A A A A A A A A A A A A A A A A A A A A A A A A A A A A A A A A A A A A . A . A A A A A A A A A A A A A A A A A A A A A A A A A . . . . . A A A A A A A . A . A A A A A A A A A A A A A A A A A . . . . A A A A A . . . . A A A A A . . . A A A A A A A A A A A A A A A A A . . A A    
626	. . . . A A A A A A A A A A A A A A A A A A A A A A A A A A A A A A A A A A A A A A A A A A A A A A A A A A A A A A A A A A A A A A A A A A A A A A A A A A A A A A A . A . A A A A A A A A A A A A A A A A A A A A A A A A A . . . . . A A A A A A A . A . A A A A A A A A A A A A A A A A A . . . . A A A A A . . . . A A A A A . . . A A A A A A A A A A A A A A A A A . . A A    
627	. . . . G G G G G G G G G G G G G G G G G G G G G G G G G G G G G G G G G G G G G G G G G G G G G G G G G G G G G G G G G G G G G G G G G G G G G G G G G G G G G G G . G . G G G G G G G G G G G G G G G G G G G G G G G G G . . . . . G G G G G G G . G . G G G G G G G G G G G G G G G G G . . . . G G G G G . . . . G G G G G . . . G G G G G G G G G G G G G G G G G . . G G    
628	. . . . C C C C C C C C C C C C C C C C C C C C C C C C C C C C C C C C C C C C C C C C C C C C C C C C C C C C C C C C C C C C C C C C C C C C C C C C C C C C C C C . C . C C C C C C C C C C C C C C C C C C C C C C C C C . . . . . C C C C C C C . C . C C C C C C C C C C C C C C C C C . . . . C C C C C . . . . C C C C C . . . C C C C C C C C C C C C C C C C C . . C C    
629	. . . . C C C C C C C C C C C C C C C C C C C C C C C C C C C C C C C C C C C C C C C C C C C C C C C C C C C C C C C C C C C C C C C C C C C C C C C C C C C C C C C . C . C C C C C C C C C C C C C C C C C C C C C C C C C . . . . . C C C C C C C . C . C C C C C C C C C C C C C C C C C . . . . C C C C C . . . . C C C C C . . . C C C C C C C C C C C C C C C C C . . C C    
630	. . . . A A A A A A A A A A A A A A A A A A A A A A A A A A A A A A A A A A A A A A A A A A A A A A A A A A A A A A A A A A A A A A A A A A A A A A A A A A A A A A A . A . A A A A A A A A A A A A A A A A A A A A A A A A A . . . . . A A A A A A A . A . A A A A A A A A A A A A A A A A A . . . . A A A A A . . . . A A A A A . . . A A A A A A A A A A A A A A A A A . . A A    
631	. . . . G G G G G G G G G G G G G G G G G G G G G G G G G G G G G G G G G G G G G G G G G G G G G G G G G G G G G G G G G G G G G G G G G G G G G G G G G G G G G G G . G . G G G G G G G G G G G G G G G G G G G G G G G G G . . . . . G G G G G G G . G . G G G G G G G G G G G G G G G G G . . . . G G G G G . . . . G G G G G . . . G G G G G G G G G G G G G G G G G . . G G    
632	. . . . C C C C C C C C C C C C C C C C C C C C C C C C C C C C C C C C C C C C T C C C C C C C C C C C C C C C C C C C C C C C C C C C C C C C C C C C C C C C C C C . C . C C C C C C C C C C C C C C C C C C C C C C C C C . . . . . C C C C C C C . C . C C C C C C C C C C C C C C C C C . . . . C C C C C . . . . C C C C C . . . C C C C C C C T C C C C C C C C C . . C C    
633	. . . . G G G G G G G G G G G G G G G G G G G G G G G G G G G G G G G G G G G G G G G G G G G G G G G G G G G G G G G G G G G G G G G G G G G G G G G G G G G G G G G . G . G G G G G G G G G G G G G G G G G G G G G G G G G . . . . . G G G G G G G . G . G G G G G G G G G G G G G G G G G . . . . G G G G G . . . . G G G G G . . . G G G G G G G G G G G G G G G G G . . G G    
634	. . . . A A A A A A A A A A A A A A A A A A A A A A A A A A A A A A A A A A A A A A A A A A A A A A A A A A A A A A A A A A A A A A A A A A A A A A A A A A A A A A A . A . A A A A A A A A A A A A A A A A A A A A A A A A A . . . . . A A A A A A A . A . A A A A A A A A A A A A A A A A A . . . . A A A A A . . . . A A A A A . . . A A A A A A A A A A A A A A A A A . . A A    
635	. . . . G G G G G G G G G G G G G G G G G G G G G G G G G G G G G G G G G G G G G G G G G G G G G G G G G G G G G G G G G G G G G G G G G G G G G G G G G G G G G G G . G . G G G G G G G G G G G G G G G G G G G G G G G G G . . . . . G G G G G G G . G . G G G G G G G G G G G G G G G G G . . . . G G G G G . . . . G G G G G . . . G G G G G G G G G G G G G G G G G . . G G    
636	. . . . T T T T T T T T T T T T T T T T T T T T T T T T T T T T T T T T T T T T T T T T T T T T T T T T T T T T T T T T T T T T T T T T T T T T T T T T T T T T T T T . T . T T T T T T T T T T T T T T T T T T T T T T T T T . . . . . T T T T T T T . T T T T T T T T T T T T T T T T T T T . . . . T T T T T . . . . T T T T T . . . T T T T T T T T T T T T T T T T T . . T T    
637	. . . . A A A A A A A A A A A A A A A A A A A A A A A A A A A A A A A A A A A A A A A A A A A A A A A A A A A A A A A A A A A A A A A A A A A A A A A A A A A A A A A . A . A A A A A A A A A A A A A A A A A A A A A A A A A . . . . . A A A A A A A . A A A A A A A A A A A A A A A A A A A . . . . A A A A A . . . . A A A A A . . . A A A A A A A A A A A A A A A A A . . A A    
638	. . . . T T T T T T T T T T T T T T T T T T T T T T T T T T T T T T T T T T T T T T T T T T T T T T T T T T T T T T T T T T T T T T T T T T T T T T T T T T T T T T T . T . T T T T T T T T T T T T T T T T T T T T T T T T T . . . . . T T T T T T T . T T T T T T T T T T T T T T T T T T T . . . . T T T T T . . . . T T T T T . . . T T T T T T T T T T T T T T T T T . . T T    
639	. . . . T T T T T T T T T T T T T T T T T T T T T T T T T T T - T T T T T T T T T T T T T T T T T T T T T T T T T T T T T T T T T T T T T T T T T T T T T T T T T T T . T . T T T T T T T T T T T T T T T T T T T T T T T T T . . . T . T T T T T T T . T T T T T T T T T T T T T T T T T T T . . . . T T T T T . . . . T T T T T . . . T T T T T T T T T T T T T T T T T . . T T    
640	. . . . C C C C C C C C C C C C C C C C C C C C C C C C C C C C C C C C C C C C C C C C C C C C C C C C C C C C C C C C C C C C C C C C C C C C C C C C C C C C C C C . C . C C C C C C C C C C C C C C C C C C C C C C C C C . . . C . C C C C C C C . C C C C C C C C C C C C C C C C C C C . . . . C C C C C . . . . C C C C C . . . C C C C C C C C C C C C C C C C C . . C C    
641	. . . . C C C C C C C C C C C C C C C C C C C C C C C C C C C C C C C C C C C C C C C C C C C C C C C C C C C C C C C C C C C C C C C C C C C C C C C C C C C C C C C . C . C C C C C C C C C C C C C C C C C C C C C C C C C . . . C . C C C C C C C . C C C C C C C C C C C C C C C C C C C . . . . C C C C C . . . . C C C C C . . . C C C C C C C C C C C C C C C C C . . C C    
642	. . . . G A A A A A A A A A A A A A A A A A A A A A A A A A A A A A A A A A A A A A A A A A A A A A A A A A A A A A A A A A A A A A A A A A A A A A A A A A A A A A A . A . A A A A A A A A A A A A A A A A A A A A A A A A A . . . A A A A A A A A A . A A A A A A A A A A A A A A A A A A A . . . . A A A A A . . . . A A A A A . . . A A A A A A A A A A A A A A A A A . . A A    
643	. . . . . T T T T T T T T T T T T T T T T T T T T T T T T T T T T T T T T T T T T T T T T T T T C T T T T T T T T T T T T T T T T T T T T T T T T T T T T T T T T T T . T . T T T T T T T T T T T T T T T T T T T T T T T T T . . . T T T T T T T T T . T T T T T T T T T T T T T T T T T T T . . . . T T T T T . . . . T T T T T . . . T T T T T T T T T T T T T T T T T . . T T    
644	. . . . . C C C C C C C C C C C C C C C C C C C C C C C C C C C C C C C C C C C C C C C C C C C C C C C C C C C C C C C C C C C C C C C C C C C C C C C C C C C C C C . C . C C C C C C C C C C C C C C C C C C C C C C C C C . . . C C C C C C C C C . C C C C C C C C C C C C C C C C C C C . . . . C C C C C . . . . C C C C C . . . C C C C C C C C C C C C C C C C C . . C C    
645	. . . . . C C C C C C C C C C C C C C C C C C C C C C C C C C C C C C C C C C C C C C C C C C C C C C C C C C C C C C C C C C C C C C C C C C C C C C C C C C C C C C . C . C C C C C C C C C C C C C C C C C C C C C C C C C . . . C C C C C C C C C . C C C C C C C C C C C C C C C C C C C . . . . C C C C C . . . . C C C C C . . . C C C C C C C C C C C C C C C C C . . C C    
646	. . . . . T T T T T T T T T T T T T T T T T T T T T T T T T T T T T T T T T T T T T T T T T T T T T T T T T T T T T T T T T T T T T T T T T T T T T T T T T T T T T T . T . T T T T T T T T T T T T T T T T T T T T T T T T T . . . T T T T T T T T T . T T T T T T T T T T T T T T T T T T T . . . . T T T T T . . . . T T T T T . . . T T T T T T T T T T T T T T T T T . . T T    
647	. . . . . T T T T T T T T T T T T T T T T T T T T T T T T T T T T T T T T T T T T T T T T T T T T T T T T T T T T T T T T T T T T T T T T T T T T T T T T T T T T T T . T . T T T T T T T T T T T T T T T T T T T T T T T T T . . . T T T T T T T T T . T T T T T T T T T T T T T T T T T T T . . . . T T T T T . . . . T T T T T . . . T T T T T T T T T T T T T T T T T . . T T    
648	. . . . . C C C C C C C C C C C C C C C C C C C C C C C C C C C C C C C C C C C C C C C C C C C C C C C C C C C C C C C C C C C C C C C C C C C C C C C C C C C C C C . C . C C C C C C C C C C C C C C C C C C C C C C C C C . . . C C C C C C C C C . C C C C C C C C C C C C C C C C C C C . . . C C C C C C . . . C C C C C C . . . C C C C C C C C C C C C C C C C C . . C C    
649	. . . . . C C C C C C C C C C C C C C C C C C C C C C C C C C C C C C C C C C C C C C C C C C C C C C C C C C C C C C C C C C C C C C C C C C C C C C C C C C C C C C . C . C C C C C C C C C C C C C C C C C C C C C C C C C . . . C C C C C C C C C . C C C C C C C C C C C C C C C C C C C . . . C C C C C C . . . C C C C C C . . . C C C C C C C C C C C C C C C C C . . C C    
650	. . . . . G G G G G G G G G G G G G G G G G G G G G G G G G G G G G G G G G G G G G G G G G G G G G G G G G G G G G G G G G G G G G G G G G G G G G G G G G G G G G G . G . G G G G G G G G G G G G G G G G G G G G G G G G G . . . G G G G G G G G G . G G G G G G G G G G G G G G G G G G G . . . G G G G G G . . . G G G G G G . . . G G G G G G G G G G G G G G G G G . . G G    
651	. . . . . A A A A A A A A A A A A A A A A A A A A A A A A A A A A A A A A A A A A A A A A A A A A A A A A A A A A A A A A A A A A A A A A A A A A G A A A A A A A A A . A . A A A A A A A A A A A A A A A A A A A A A A A A A . . . A A A A A A A A A . A A A A A A A A A A A A A A A A A A A . . . A A A A A A . . . A A A A A A . . . A G A A A A A A A A A A A A A A A . . A A    
652	. . . . . C C C C C C C C C C C C C C C C C C C C C C C C C C C C C C C C C C C C C C C C C C C C C C C C C C C C C C C C C C C C C C C C C C C C C C C C C C C C C C . C . C C C C C C C C C C C C C C C C C C C C C C C C C . . . C C C C C C C C C . C C C C C C C C C C C C C C C C C C C . . . C C C C C C . . . C C C C C C . . . C C C C C C C C C C C C C C C C C . . C C    
653	. . . . . C C C C C C C C C C C C C C C C C C C C C C C C C C C C C C C C C C C C C C C C C C C C C C C C C C C C C C C C C C C C C C C C C C C C C C C C C C C C C C . C . C C C C C C C C C C C C C C C C C C C C C C C C C . . . C C C C C C C C C . C C C C C C C C C C C C C C C C C C C . . . C C C C C C . . . C C C C C C . . . C C C C C C C C C C C C C C C C C . . C C    
654	. . . . . C C C C C C C C C C C C C C C C C C C C C C C C C C C C C C C C C C C C C C C C C C C C C C C C C C C C C C C C C C C C C C C C C C C C C C C C C C C C C C . C . C C C C C C C C C C C C C C C C C C C C C C C C C . . . C C C C C C C C C . C C C C C C C C C C C C C C C C C C C . . . C C C C C C . . . C C C C C C . . . C C C C C C C C C C C C C C C C C . . C C    
655	. . . . . C C C C C C C C C C C C C C C C C C C C C C C C C C C C C C C C C C C C C C C C C C C C C C C C C C C C C C C C C C C C C C C C C C C C C C C C C C C C C C . C . C C C C C C C C C C C C C C C C C C C C C C C C C . . . C C C C C C C C C . C C C C C C C C C C C C C C C C C C C . . . C C C C C C . . . C C C C C C . . . C C C C C C C C C C C C C C C C C . . C C    
656	. . . . . A A A A A A A A A A A A A A A A A A A A A A A A A A A A A A A A A A A A A A A A A A A A A A A A A A A A A A A A A A A A A A A A A A A A A A A A A A A A A A . A . A A A A A A A A A A A A A A A A A A A A A A A A A . . . A A A A A A A A A . A A A A A A A A A A A A A A A A A A A . . . A A A A A A . . . A A A A A A . . . A A A A A A A A A A A A A A A A A . . A A    
657	. . . . . G G G G G G G G G G G G G G G G G G G G G G G G G G G G G G G G G G G G G G G G G G G G G G G G G G G G G G G G G G G G G G G G G G G G G G G G G G G G G G . G . G G G G G G G G G G G G G G G G G G G G G G G G G . . . G G G G G G G G G . G G G G G G G G G G G G G G G G G G G . . . G G G G G G . . . G G G G G G . . . G G G G G G G G G G G G G G G G G . . G G    
658	. . . . . T T T T T T T T T T T T T T T T T T T T T T T T T T T T T T T T T T T T T T T T T T T T T T T T T T T T T T T T T T T T T T T T T T T T T T T T T T T T T T . T . T T T T T T T T T T T T T T T T T T T T T T T T T . . . T T T T T T T T T . T T T T T T T T T T T T T T T T T T T . . . T T T T T T . . . T T T T T T . . . T T T T T T T T T T T T T T T T T . . T T    
659	. . . . . G G G G G G G G G G G G G G G G G G G G G G G G G G G G G G G G G G G G G G G G G G G G G G G G G G G G G G G G G G G G G G G G G G G G G G G G G G G G G G . G . G G G G G G G G G G G G G G G G G G G G G G G G G . . . G G G G G G G G G . G G G G G G G G G G G G G G G G G G G . . . G G G G G G . . . G G G G G G . . . G G G G G G G G G G G G G G G G G . . G G    
660	. . . . . T T T T T T T T T T T T T T T T T T T T T T T T T T T T T T T T T T T T T T T T T T T T T T T T T T T T T T T T T T T T T T T T T T T T T T T T T T T T T T . T . T T T T T T T T T T T T T T T T T T T T T T T T T . . . T T T T T T T T T . T T T T T T T T T T T T T T T T T T T . . . T T T T T T . . . T T T T T T . . . T T T T T T T T T T T T T T T T T . . T T    
661	. . . . . G G G G G G G G G G G G G G G G G G G G G G G G G G G G G G G G G G G G G G G G G G G G G G G G G G G G G G G G G G G G G G G G G G G G G G G G G G G G G G . G . G G G G G G G G G G G G G G G G G G G G G G G G G . . . G G G G G G G G G . G G G G G G G G G G G G G G G G G G G . . . G G G G G G . . . G G G G G G . . . G G G G G G G G G G G G G G G G G . . G G    
662	. . . . . A A A A A A A A A A A A A A A A A A A A A A A A A A A A A A A A A A A A A A A A A A A A A A A A A A A A A A A A A A A A A A A A A A A A A A A A A A A A A A . A . A A A A A A A A A A A A A A A A A A A A A A A A A . . . A A A A A A A A A . A A A A A A A A A A A A A A A A A A A . . . A A A A A A . . . A A A A A A . . . A A A A A A A A A A A A A A A A A . . A A    
663	. . . . . T T T T T T T T T T T T T T T T T T T T T T T T T T T T T T T T T T T T T T T T T T T T T T T T T T T T T T T T T T T T T T T T T T T T T T T T T T T T T T . T . T T T T T T T T T T T T T T T T T T T T T T T T T . . . T T T T T T T T T . T T T T T T T T T T T T T T T T T T T . . . T T T T T T . . . T T T T T T . . . T T T T T T T T T T T T T T T T T . . T T    
664	. . . . . G G G G G G G G G G G G G G G G G G G G G G G G G G G G G G G G G G G G G G G G G G G G G G G G G G G G G G G G G G G G G G G G G G G G G G G G G G G G G G . G . G G G G G G G G G G G G G G G G G G G G G G G G G . . . G G G G G G G G G . G G G G G G G G G G G G G G G G G G G . . . G G G G G G . . . G G G G G G . . . G G G G G G G G G G G G G G G G G . . G G    
665	. . . . . A A A A A A A A A A A A A A A A A A A A A A A A A A A A A A A A A A A A A A A A A A A A A A A A A A A A A A A A A A A A A A A A A A A A A A A A A A A A A A . A . A A A A A A A A A A A A A A A A A A A A A A A A A . . . A A A A A A A A A . A A A A A A A A A A A A A A A A A A A . . . A A A A A A . . . A A A A A A . . . A A A A A A A A A A A A A A A A A . . A A    
666	. . . . . G G G G G G G G G G G G G G G G G G G G G G G G G G G G G G G G G G G A G G G G G G G G G G G G G G G G G G G G G G G G G G G G G G G G G G G G G G G G G G . G . G G G G G G G G G G G G G G G G G G G G G G G G G . . . G G G G G G G G G . G G G G G G G G G G G G G G G G G G G . . . G G G G G G . . . G G G G G G . . . G G G G G G G A G G G G G G G G G . . G G    
667	. . . . . C C C C C C C C C C C C C C C C C C C C C C C C C C C C C C C C C C C C C C C C C C C C C C C C C C C C C C C C C C C C C C C C C C C C C C C C C C C C C C . C C C C C C C C C C C C C C C C C C C C C C C C C C C . . . C C C C C C C C C . C C C C C C C C C C C C C C C C C C C . . . C C C C C C . . . C C C C C C . . . C C C C C C C C C C C C C C C C C . . C C    
668	. . . . . A A A A A A A A A A A A A A A A A A A A A A A A A A A A A A A A A A A A A A A A A A A A A A A A A A A A A A A A A A A A A A A A A A A A A A A A A A A A A A . A A A A A A A A A A A A A A A A A A A A A A A A A A A . . . A A A A A A A A A . A A A A A A A A A A A A A A A A A A A . . . A A A A A A . . . A A A A A A . . . A A A A A A A A A A A A A A A A A . . A A    
669	. . . . . G G G G G G G G G G G G G G G G G G G G G G G G G G G G G G G G G G G G G G G G G G G G G G G G G G G G G G G G G G G G G G G G G G G G G G G G G G G G G G . G G G G G G G G G G G G G G G G G G G G G G G G G G G . . . G G G G G G G G G G G G G G G G G G G G G G G G G G G G G . . . G G G G G G . . . G G G G G G . . . G G G G G G G G G G G G G G G G G . . G G    
670	. . . . . G G G G G G G G G G G G G G G G G G G G G G G G G G G G G G G G G G G G G G G G G G G G G G G G G G G G G G G G G G G G G G G G G G G G G G G G G G G G G G . G G G G G G G G G G G G G G G G G G G G G G G G G G G . . G G G G G G G G G G G G G G G G G G G G G G G G G G G G G G . . . G G G G G G . . . G G G G G G . . . G G G G G G G G G G G G G G G G G . . G G    
671	. . . . . G G G G G G G G G G G G G G G G G G G G G G G G G G G G G G G G G G G G G G G G G G G G G G G G G G G G G G G G G G G G G G G G G G G G G G G G G G G G G G . G G G G G G G G G G G G G G G G G G G G G G G G G G G . . G G G G G G G G G G G G G G G G G G G G G G G G G G G G G G . . . G G G G G G . . . G G G G G G . . . G G G G G G G G G G G G G G G G G . . G G    
672	. . . . . C C C C C C C C C C C C C C C C C C C C C C C C C C C C C C C C C C C C C C C C C C C C C C C C C C C C C C C C C C C C C C C C C C C C C C C C C C C C C C . C C C C C C C C C C C C C C C C C C C C C C C C C C C . . C C C C C C C C C C C C C C C C C C C C C C C C C C C C C C . . . C C C C C C . . . C C C C C C . . . C C C C C C C C C C C C C C C C C . . C C    
673	. . . . . A A A A A A A A A A A A A A A A A A A A A A A A A A A A A A A A A A A A A A A A A A A A A A A A A A A A A A A A A A A A A A A A A A A A A A A A A A A A A A . A A A A A A A A A A A A A A A A A A A A A A A A A A A . . A A A A A A A A A A A A A A A A A A A A A A A A A A A A A A . . . A A A A A A . . . A A A A A A . . . A A A A A A A A A A A A A A A A A . . A A    
674	. . . . . A A A A A A A A A A A A A A A A A A A A A A A A A A A A A A A A A A A A A A A A A A A A A A A A A A A A A A A A A A A A A A A A A A A A A A A A A A A A A A . A A A A A A A A A A A A A A A A A A A A A A A A A A A . . A A A A A A A A A A A A A A A A A A A A A A A A A A A A A A . . . A A A A A A . . . A A A A A A . . . A A A A A A A A A A A A A A A A A . . A A    
675	. . . . . C C C C C C C C C C T C C C C C C C C C C C C C C C C C C C C C C C C C C C C C C C C C C C C C C C C C C C C C C C C C C C C C C C C C C C C C C C C C C C . C C C C C C C C C C C C C C C C C C C C C C C C C C C . . C C C C C C C C C C C C C C C C C C C C C C C C C C C C C C . . . C C C C C C . . . C C C C C C . . . C C C C C C C C C C C C C C C C C . . C C    
676	. . . . . T T T T T T T T T T T T T T T T T T T T T T T T T T T T T T T T T T T T T T T T T T T T T T T T T T T T T T T T T T T T T T T T T T T T T T T T T T T T T T . T T T T T T T T T T T T T T T T T T T T T T T T T T T . . T T T T T T T T T T T T T T T T T T T T T T T T T T T T T T . . . T T T T T T . . . T T T T T T . . . T T T T T T T T T T T T T T T T T . . T T    
677	. . . . . A A A A A A A A A A A A A A A A A A A A A A A A A A A A A A A A A A A A A A A A A A A A A A A A A A A A A A A A A A A A A A A A A A A A A A A A A A A A A A . A A A A A A A A A A A A A A A A A A A A A A A A A A A . . A A A A A A A A A A A A A A A A A A A A A A A A A A A A A A . . . A A A A A A . . . A A A A A A . . . A A A A A A A A A A A A A A A A A . . A A    
678	. . . . . A C C C C C C C C C C C C C C C C C C C C C C C C C C C C C C C C C C C C C C C C C C C C C C C C C C C C C C C C C C C C C C C C C C C C C C C C C C C C C . C C C C C C C C C C C C C C C C C C C C C C C C C C C . . C C C C C C C C C C C C C C C C C C C C C C C C C C C C C C . . . C C C C C C . . . C C C C C C . . . C C C C C C C C C C C C C C C C C . . C C    
679	. . . . . . C C C C C C C C C C C C C C C C C C C C C C C C C C C C C C C C C C C C C C C C C C C C C C C C C C C C C C C C C C C C C C C C C C C C C C C C C C C C C . C C C C C C C C C C C C C C C C C C C C C C C C C C C . . C C C C C C C C C C C C C C C C C C C C C C C C C C C C C C . . . C C C C C C . . . C C C C C C . . . C C C C C C C C C C C C C C C C C . . C C    
680	. . . . . . T T T T T T T T T T T T T T T T T T T T T T T T T T T T T T T T T T T T T T T T T T T T T T T T T T T T T T T T T T T T T T T T T T T T T T T T T T T T T . T T T T T T T T T T T T T T T T T T T T T T T T T T T . . T T T T T T T T T T T T T T T T T T T T T T T T T T T T T T . . . T T T T T T . . . T T T T T T . . . T T T T T T T T T T T T T T T T T . . T T    
681	. . . . . . G G G G G G G G G G G G G G G G G G G G G G G G G G G G G G G G G G G G G G G G G G G G G G G G G G G G G G G G G G G G G G G G G G G G G G G G G G G G G . G G G G G G G G G G G G G G G G G G G G G G G G G G G . . G G G G G G G G G G G G G G G G G G G G G G G G G G G G G G . . . G G G G G G . . . G G G G G G . . . G G G G G G G G G G G G G G G G G . . G G    
682	. . . . . . C C C C C C C C C C C C C C C C C C C C C C C C C C C C C C C C C C C C C C C C C C C C C C C C C C C C C C C C C C C C C C C C C C C C C C C C C C C C C . C C C C C C C C C C C C C C C C C C C C C C C C C C C . . C C C C C C C C C C C C C C C C C C C C C C C C C C C C C C . . . C C C C C C . . . C C C C C C . . . C C C C C C C C C C C C C C C C C . . C C    
683	. . . . . . T T T T T T T T T T T T T T T T T T T T T T T T T T T T T T T T T T T T T T T T T T T T T T T T T T T T T T T T T T T T T T T T T T T T T T T T T T T T T . T T T T T T T T T T T T T T T T T T T T T T T T T T T . . T T T T T T T T T T T T T T T T T T T T T T T T T T T T T T . . . T T T T T T . . . T T T T T T . . . T T T T T T T T T T T T T T T T T . . T T    
684	. . . . . . C C C C C C C C C C C C C C C C C C C C C C C C C C C C C C C C C C C C C C C C C C C C C C C C C C C C C C C C C C C C C C C C C C C C C C C C C C C C C . C C C C C C C C C C C C C C C C C C C C C C C C C C C . . C C C C C C C C C C C C C C C C C C C C C C C C C C C C C C . . . C C C C C C . . . C C C C C C . . . C C C C C C C C C C C C C C C C C . . C C    
685	. . . . . . C C C C C C C C C C C C C C C C C C C C C C C C C C C C C C C C C C C C C C C C C C C C C C C C C C C C C C C C C C C C C C C C C C C C C C C C C C C C C . C C C C C C C C C C C C C C C C C C C C C C C C C C C . . C C C C C C C C C C C C C C C C C C C C C C C C C C C C C C . . . C C C C C C . . . C C C C C C . . . C C C C C C C C C C C C C C C C C . . C C    
686	. . . . . . A A A A A A A A A A A A A A A A A A A A A A A A A A A A A A A A A A A A A A A A A A A A A A A A A A A A A A A A A A A A A A A A A A A A A A A A A A A A A . A A A A A A A A A A A A A A A A A A A A A A A A A A A . . A A A A A A A A A A A A A A A A A A A A A A A A A A A A A A . . . A A A A A A . . . A A A A A A . . . A A A A A A A A A A A A A A A A A . . A A    
687	. . . . . . G G G G G G G G G G G G G G G G G G G G G G G G G G G G G G G G G G G G G G G G G G G G G G G G G G G G G G G G G G G G G G G G G G G G G G G G G G G G G . G G G G G G G G G G G G G G G G G G G G G G G G G G G . . G G G G G G G G G G G G G G G G G G G G G G G G G G G G G G . . . G G G G G G . . . G G G G G G . . . G G G G G G G G G G G G G G G G G . . G G    
688	. . . . . . C C C C C C C C C C C C C C C C C C C C C C C C C C C C C C C C C C C C C C C C C C C C C C C C C C C C C C C C C C C C C C C C C C C C C C C C C C C C C . C C C C C C C C C C C C C C C C C C C C C C C C C C C . . C C C C C C C C C C C C C C C C C C C C C C C C C C C C C C . . . C C C C C C . . . C C C C C C . . . C C C C C C C C C C C C C C C C C . . C C    
689	. . . . . . A A A A A A A A A A A A A A A A A A A A A A A A A A A A A A A A A A A A A A A A A A A A A A A A A A A A A A A A A A A A A A A A A A A A A A A A A A A A A . A A A A A A A A A A A A A A A A A A A A A A A A A A A . . A A A A A A A A A A A A A A A A A A A A A A A A A A A A A A . . . A A A A A A . . . A A A A A A . . . A A A A A A A A A A A A A A A A A . . A A    
690	. . . . . . G G G G G G G G G G G G G G G G G G G G G G G G G G G G G G G G G G G G G G G G G G G G G G G G G G G G G G G G G G G G G G G G G G G G G G G G G G G G N . G G G G G G G G G G G G G G G G G G G G G G G G G G G . . G G G G G G G G G G G G G G G G G G G G G G G G G G G G G G . . . G G G G G G . . . G G G G G G . . . G G G G G G G G G G G G G G G G G . . G G    
691	. . . . . . T T T T T T T T T T T T T T T T T T T T T T T T T T T T T T T T T T T T T T T T T T T T T T T T T T T T T T T T T T T T T T T T T T T T T T T T T T T T T . T T T T T T T T T T T T T T T T T T T T T T T T T T T . . T T T T T T T T T T T T T T T T T T T T T T T T T T T T T T . . . T T T T T T . . . T T T T T T . . . T T T T T T T T T T T T T T T T T . . T T    
692	. . . . . . G G G G G G G G G G G G G G G G G G G G G G G G G G G G G G G G G G G G G G G G G G G G G G G G G G G G G G G G G G G G G G G G G G G G G G G G G G G G G . G G G G G G G G G G G G G G G G G G G G G G G G G G G . . G G G G G G G G G G G G G G G G G G G G G G G G G G G G G G . . . G G G G G G . . . G G G G G G . . G G G G G G G G G G G G G G G G G G . . G G    
693	. . . . . . C C C C C C C C C C C C C C C C C C C C C C C C C C C C C C C C C C C C C C C C C C C C C C C C C C C C C C C C C C C C C C C C C C C C C C C C C C C C C . C C C C C C C C C C C C C C C C C C C C C C C C C C C . . C C C C C C C C C C C C C C C C C C C C C C C C C C C C C C . . . C C C C C C . . . C C C C C C . . C C C C C C C C C C C C C C C C C C . . C C    
694	. . . . . . T T T T T T T T T T T T T T T T T T T T T T T T T T T T T T T T T T T T T T T T T T T T T T T T T T T T T T T T T T T T T T T T T T T T T T T T T T T T T . T T T T T T T T T T T T T T T T T T T T T T T T T T T . . T T T T T T T T T T T T T T T T T T T T T T T T T T T T T T . . . T T T T T T . . . T T T T T T . . T T T T T T T T T T T T T T T T T T . . T T    
695	. . . . . . T T T T T T T T T T T T T T T T T T T T T T T T T T T T T T T T T T T T T T T T T T T T T T T T T T T T T T T T T T T T T T T T T T T T T T T T T T T T T . T T T T T T T T T T T T T T T T T T T T T T T T T T T . . T T T T T T T T T T T T T T T T T T T T T T T T T T T T T T . . . T T T T T T . . . T T T T T T . . T T T T T T T T T T T T T T T T T T . . T T    
696	. . . . . . G G G G G G G G G G G G G G G G G G G G G G G G G G G G G G G G G G G G G G G G G G G G G G G G G G G G G G G G G G G G G G G G G G G G G G G G G G G G G . G G G G G G G G G G G G G G G G G G G G G G G G G G G . . G G G G G G G G G G G G G G G G G G G G G G G G G G G G G G . . . G G G G G G . . . G G G G G G . . G G G G G G G G G G G G G G G G G G . . G G    
697	. . . . . . G G G G G G G G G G G G G G G G G G G G G G G G G G G G G G G G G G G G G G G G G G G G G G G G G G G G G G G G G G G G G G G G G G G G G G G G G G G G G . G G G G G G G G G G G G G G G G G G G G G G G G G G G . . G G G G G G G G G G G G G G G G G G G G G G G G G G G G G G . . . G G G G G G . . . G G G G G G . . G G G G G G G G G G G G G G G G G G . . G G    
698	. . . . . . G A A A A A A A A A A A A A A A A A A A A A A A A A A A A A A A A A A A A A A A A A A A A A A A A A A A A A A A A A A A A A A A A A A A A A A A A A A A A . A A A A A A A A A A A A A A A A A A A A A A A A A A A . . A A A A A A A A A A A A A A A A A A A A A A A A A A A A A A . . . A A A A A A . . . A A A A A A . . A A A A A A A A A A A A A A A A A A . . A A    
699	. . . . . . . C C C C C C C C C C C C C C C C C C C C C C C C C C C C C C C C C C C C C C C C C C C C C C C C C C C C C C C C C C C C C C C C C C C C C C C C C C C C . C C C C C C C C C C C C C C C C C C C C C C C C C C C . . C C C C C C C C C C C C C C C C C C C C C C C C C C C C C C . . . C C C C C C . . . C C C C C C . . C C C C C C C C C C C C C C C C C C . . C C    
700	. . . . . . . A A A A A A A A A A A A A A A A A A A A A A A A A A A A A A A A A A A A A A A A A A A A A A A A A A A A A A A A A A A A A A A A A A A A A A A A A A A A . A A A A A A A A A A A A A A A A A A A A A A A A A A A . . A A A A A A A A A A A A A A A A A A A A A A A A A A A A A A . . . A A A A A A . . . A A A A A A . . A A A A A A A A A A A A A A A A A A . . A A    
701	. . . . . . . C C C C C C C C C C C C C C C C C C C C C C C C C C C C C C C C C C C C C C C C C C C C C C C C C C C C C C C C C C C C C C C C C C C C C C C C C C C C . C C C C C C C C C C C C C C C C C C C C C C C C C C C . . C C C C C C C C C C C C C C C C C C C C C C C C C C C C C C . . . C C C C C C . . C C C C C C C . . C C C C C C C C C C C C C C C C C C . . C C    
702	. . . . . . . C C C C C C C C C C C C C C C C C C C C C C C C C C C C C C C C C C C C C C C C C C C C C C C C C C C C C C C C C C C C C C C C C C C C C C C C C C C C . C C C C C C C C C C C C C C C C C C C C C C C C C C C . . C C C C C C C C C C C C C C C C C C C C C C C C C C C C C C . . . C C C C C C . . C C C C C C C . . C C C C C C C C C C C C C C C C C C . . C C    
703	. . . . . . . A A A A A A A A A A A A A A A A A A A A A A A A A A A A A A A A A A A A A A A A A A A A A A A A A A A A A A A A A A A A A A A A A A A A A A A A A A A A . A A A A A A A A A A A A A A A A A A A A A A A A A A A . . A A A A A A A A A A A A A A A A A A A A A A A A A A A A A A . . . A A A A A A . . A A A A A A A . . A A A A A A A A A A A A A A A A A A . . A A    
704	. . . . . . . C C C C C C C C C C C C C C C C C C C C C C C C C C C C C C C C C C C C C C C C C C C C C C C C C C C C C C C C C C C C C C C C C C C C C C C C C C C C . C C C C C C C C C C C C C C C C C C C C C C C C C C C . . C C C C C C C C C C C C C C C C C C C C C C C C C C C C C C . . . C C C C C C . . C C C C C C C . . C C C C C C C C C C C C C C C C C C . . C C    
705	. . . . . . . A A A A A A A A A A A A A A A A A A A A A A A A A A A A A A A A A A A A A A A A A A A A A A A A A A A A A A A A A A A A A A A A A A A A A A A A A A A A . A A A A A A A A A A A A A A A A A A A A A A A A A A A . . A A A A A A A A A A A A A A A A A A A A A A A A A A A A A A . . . A A A A A A . . A A A A A A A . . A A A A A A A A A A A A A A A A A A . . A A    
706	. . . . . . . C C C C C C C C C C C C C C C C C C C C C C C C C C C C C C C C C C C C C C C C C C C C C C C C C C C C C C C C C C C C C C C C C C C C C C C C C C C C . C C C C C C C C C C C C C C C C C C C C C C C C C C C . . C C C C C C C C C C C C C C C C C C C C C C C C C C C C C C . . . C C C C C C . . C C C C C C C . . C C C C C C C C C C C C C C C C C C . . C C    
707	. . . . . . . - - - - - - - - - - - - N - - - - - - - - - - - - - - - - - - - - - - - - - - - - - - - - - - - - - - - - - - - - - - - - - - - - - - - - - - - - - - - . - - - - - - - - - - - - - - - - - - - - - - - - - - - . . - - - - - - - - - - - - - - - - - - - - - - - - - - - - - - . . . - - - - - - . . - - - - - - - . . - - - - - - - - - - - - - - - - - - . . - -    
708	. . . . . . . C C C C C C C C C C C C C C C C C C C C C C C C C C C C C C C C C C C C C C C C C C C C C C C C C C C C C C C C C C C C C C C C C C C C C C C C C C C C . C C C C C C C C C C C C C C C C C C C C C C C C C C C . . C C C C C C C C C C C C C C C C C C C C C C C C C C C C C C . . . C C C C C C . C C C C C C C C . . C C C C C C C C C C C C C C C C C C . . C C    
709	. . . . . . . C C C C C C C C C C C C C C C C C C C C C C C C C C C C C C C C C C C C C C C C C C C C C C C C C C C C C C C C C C C C C C C C C C C C C C C C C C C C . C C C C C C C C C C C C C C C C C C C C C C C C C C C . . C C C C C C C C C C C C C C C C C C C C C C C C C C C C C C . . . C C C C C C . C C C C C C C C . . C C C C C C C C C C C C C C C C C C . . C C    
710	. . . . . . . C C . C C C C C C C C C C C C C C C C C C C C C C C C C C C C C C C C C C C C C C C C C C C C C C C C C C C C C C C C C C C C C C C C C C C C C C C C C . C C C C C C C C C C C C C C C C C C C C C C C C C C C . . C C C C C C C C C C C C C C C C C C C C C C C C C C C C C C . . . C C C C C C . C C C C C C C C . . C C C C C C C C C C C C C C C C C C . . C C    
711	. . . . . . . T T . T T T T T T T T T T T T T T T T T T T T T T T T T T T T T T T T T T T T T T T T T T T T T T T T T T T T T T T T T T T T T T T T T T T T T T T T T . T T T T T T T T T T T T T T T T T T T T T T T T T T T . . T T T T T T T T T T T T T T T T T T T T T T T T T T T T T T . . . T T T T T T . T T T T T T T T . . T T T T T T T T T T T T T T T T T T . . T T    
712	. . . . . . . T T . T T T T T T T T T T T T T T T T T T T T T T T T T T T T T T T T T T T T T T T T T T T T T T T T T T T T T T T T T T T T T T T T T T T T T T T T T . T T T T T T T T T T T T T T T T T T T T T T T T T T T . . T T T T T T T T T T T T T T T T T T T T T T T T T T T T T T . . . T T T T T T . T T T T T T T T . . T T T T T T T T T T T T T T T T T T . . T T    
713	. . . . . . . T T . T T T T T T T T T T T T T T T T T T T T T T T T T T T T T T T T T T T T T T T T T T T T T T T T T T T T T T T T T T T T T T T T T T T T T T T T T . T T T T T T T T T T T T T T T T T T T T T T T T T T T . . T T T T T T T T T T T T T T T T T T T T T T T T T T T T T T . . . T T T T T T . T T T T T T T T . . T T T T T T T T T T T T T T T T T T . . T T    
714	. . . . . . . G G . G G G G G G G G G G G G G G G G G G G G G G G G G G G G G G G G G G G G G G G G G G G G G G G G G G G G G G G G G G G G G G G G G G G G G G G G G . G G G G G G G G G G G G G G G G G G G G G G G G G G G . . G G G G G G G G G G G G G G G G G G G G G G G G G G G G G G . . . G G G G G G . G G G G G G G G . . G G G G G G G G G G G G G G G G G G . . G G    
715	. . . . . . . C C . C C C C C C C C C C C C C C C C C C C C C C C C C C C C C C C C C C C C C C C C C C C C C C C C C C C C C C C C C C C C C C C C C C C C C C C C C . C C C C C C C C C C C C C C C C C C C C C C C C C C C . . C C C C C C C C C C C C C C C C C C C C C C C C C C C C C C . . . C C C C C C . C C C C C C C C . . C C C C C C C C C C C C C C C C C C . . C C    
716	. . . . . . . T T . T T T T T T T T T T T T T T T T T T T T T T T T T T T T T T T T T T T T T T T T T T T T T T T T T T T T T T T T T T T T T T T T T T T T T T T T T . T T T T T T T T T T T T T T T T T T T T T T T T T T T . . T T T T T T T T T T T T T T T T T T T T T T T T T T T T T T . . . T T T T T T . T T T T T T T T . . T T T T T T T T T T T T T T T T T T . . T T    
717	. . . . . . . G G . G G G G G G G G G G G G G G G G G G G G G G G G G G G G G G G G G G G G G G G G G G G G G G G G G G G G G G G G G G G G G G G G G G G G G G G G G . G G G G G G G G G G G G G G G G G G G G G G G G G G G . . G G G G G G G G G G G G G G G G G G G G G G G G G G G G G G . . . G G G G G G . G G G G G G G G . . G G G G G G G G G G G G G G G G G G . . G G    
718	. . . . . . . . . . G G G G G G G G G G G G G G G G G G G G G G G G G G G G G G G G G G G G G G G G G G G G G G G G G G G G G G G G G G G G G G G G G G G G G G G G G . G G G G G G G G G G G G G G G G G G G G G G G G G G G . . G G G G G G G G G G G G G G G G G G G G G G G G G G G G G G . . . G G G G G G . G G G G G G G G . . G G G G G G G G G G G G G G G G G G . . G G    
719	. . . . . . . . . . T T T T T T T T T T T T T T T T T T T T T T T T T T T T T T T T T T T T T T T T T T T T T T T T T T T T T T T T T T T T T T T T T T T T T T T T T . T T T T T T T T T T T T T T T T T T T T T T T T T T T . . T T T T T T T T T T T T T T T T T T T T T T T T T T T T T T . . . T T T T T T . T T T T T T T T . . T T T T T T T T T T T T T T T T T T . . T T    
720	. . . . . . . . . . G G G G G G G G G G G G G G G G G G G G G G G G G G G G G G G G G G G G G G G G G G G G G G G G G G G G G G G G G G G G G G G G G G G G G G G G G . G G G G G G G G G G G G G G G G G G G G G G G G G G G . . G G G G G G G G G G G G G G G G G G G G G G G G G G G G G G . . . G G G G G G . G G G G G G G G . . G G G G G G G G G G G G G G G G G G . . G G    
721	. . . . . . . . . . T T T T T T T T T T T T T T T T T T T T T T T T T T T T T T T T T T T T T T T T T T T T T T T T T T T T T T T T T T T T T T T T T T T T T T T T T . T T T T T T T T T T T T T T T T T T T T T T T T T T T . . T T T T T T T T T T T T T T T T T T T T T T T T T T T T T T . . . T T T T T T . T T T T T T T T . . T T T T T T T T T T T T T T T T T T . . T T    
722	. . . . . . . . . . G G G G G G G G G G G G G G G G G G G G G G G G G G G G G G G G G G G G G G G G G G G G G G G G G G G G G G G G G G G G G G G G G G G G G G G G G . G G G G G G G G G G G G G G G G G G G G G G G G G G G . . G G G G G G G G G G G G G G G G G G G G G G G G G G G G G G . . . G G G G G G . G G G G G G G G . . G G G G G G G G G G G G G G G G G G . . G G    
723	. . . . . . . . . . T T T T T T T T T T T T T T T T T T T T T T T T T T T T T T T T T T T T T T T T T T T T T T T T T T T T T T T T T T T T T T T T T T T T T T T T T . T T T T T T T T T T T T T T T T T T T T T T T T T T T . . T T T T T T T T T T T T T T T T T T T T T T T T T T T T T T . . . T T T T T T . T T T T T T T T . . T T T T T T T T T T T T T T T T T T . . T T    
724	. . . . . . . . . . G G G G G G G G G G G G G G G G G G G G G G G G G G G G G G G G G G G G G G G G G G G G G G G G G G G G G G G G G G G G G G G G G G G G G G G G G . G G G G G G G G G G G G G G G G G G G G G G G G G G G . . G G G G G G G G G G G G G G G G G G G G G G G G G G G G G G . . . G G G G G G . G G G G G G G G . . G G G G G G G G G G G G G G G G G G . . G G    
725	. . . . . . . . . . G G G G G G G G G G G G G G G G G G G G G G G G G G G G G G G G G G G G G G G G G G G G G G G G G G G G G G G G G G G G G G G G G G G G G G G G G . G G G G G G G G G G G G G G G G G G G G G G G G G G G . . G G G G G G G G G G G G G G G G G G G G G G G G G G G G G G . . . G G G G G G . G G G G G G G G . . G G G G G G G G G G G G G G G G G G . . G G    
726	. . . . . . . . . . A A A A A A A A A A A A A A A A A A A A A A A A A A A A A A A A A A A A A A A A A A A A A A A A A A A A A A A A A A A A A A A A A A A A A A A A A . A A A A A A A A A A A A A A A A A A A A A A A A A A A . . A A A A A A A A A A A A A A A A A A A A A A A A A A A A A A . . . A A A A A A . A A A A A A A A . . A A A A A A A A A A A A A A A A A A . . A A    
727	. . . . . . . . . . T T T T T T T T T T T T T T T T T T T T T T T T T T T T T T T T T T T T T T T T T T T T T T T T T T T T T T T T T T T T T T T T T T T T T T T T T . T T T T T T T T T T T T T T T T T T T T T T T T T T T . . T T T T T T T T T T T T T T T T T T T T T T T T T T T T T T . . . T T T T T T . T T T T T T T T . . T T T T T T T T T T T T T T T T T T . . T T    
728	. . . . . . . . . . G G G G G G T G G G G G G G G G G G G G G G G G G G G G G G G G G G G G G G G G G G G G G G G G G G G G G G G G G G G G G G G G G G G G G G G G G . G G G G G G G G G G G G G G G G G G G G G G G G G G G . . G G G A G G G G G G G G G G G G G G G G G G G G G G G G G G . . . G G G G G G . G G G G G G G G . . G G G G G G G G G G G G G G G G G G . . G G    
729	. . . . . . . . . . C C C C C C . C C C C C C C C C C C C C C C C C C C C C C C C C C C C C C C C C C C C C C C C C C C C C C C C C C C C C C C C C C C C C C C C C C . C C C C C C C C C C C C C C C C C C C C C C C C C C C . . C C C C C C C C C C C C C C C C C C C C C C C C C C C C C C . . . C C C C C C . C C C C C C C C . . C C C C C C C C C C C C C C C C C C . . C C    
730	. . . . . . . . . . C C C C C C . C C C C C C C C C C C C C C C C C C C C C C C C C C C C C C C C C C C C C C C C C C C C C C C C C C C C C C C C C C C C C C C C C C . C C C C C C C C C C C C C C C C C C C C C C C C C C C . . C C C C C C C C C C C C C C C C C C C C C C C C C C C C C C . . . C C C C C C . C C C C C C C C . . C C C C C C C C C C C C C C C C C C . . C C    
731	. . . . . . . . . . A A A A A A . A A A A A A A A A A A A A A A A A A A A A A A A A A A A A A A A A A A A A A A A A A A A A A A A A A A A A A A A A A A A A A A A A A . A A A A A A A A A A A A A A A A A A A A A A A A A A A . . A A A A A A A A A A A A A A A A A A A A A A A A A A A A A A . . . A A A A A A . A A A A A A A A . . A A A A A A A A A A A A A A A A A A . . A A    
732	. . . . . . . . . . G G G G G G . G G G G G G G G G G G G G G G G G G G G G G G G G G G G G G G G G G G G G G G G G G G G G G G G G G G G G G G G G G G G G G G G G G . G G G G G G G G G G G G G G G G G G G G G G G G G G G . . G G G G G G G G G G G G G G G G G G G G G G G G G G G G G G . . . G G G G G G . G G G G G G G G . . G G G G G G G G G G G G G G G G G G . . G G    
733	. . . . . . . . . . T T T T . T . T T T T T T T T T T T T T T T T T T T T T T T T T T T T T T T T T T T T T T T T T T T T T T T T T T T T T T T T T T T T T T T T T T . T T T T T T T T T T T T T T T T T T T T T T T T T T T . . T T T T T T T T T T T T T T T T T T T T T T T T T T T T T T . . . T T T T T T . T T T T T T T T . . T T T T T T T T T T T T T T T T T T . . T T    
734	. . . . . . . . . . G G G . . G . G G G G G G G G G G G G G G G G G G G G G G G G G G G G G G G G G G G G G G G G G G G G G G G G G G G G G G G G G G G G G G G G G G . G G G G G G G G G G G G G G G G G G G G G G G G G G G . . G G G G G G G G G G G G G G G G G G G G G G G G G G G G G G . . . G G G G G G . G G G G G G G G . . G G G G G G G G G G G G G G G G G G . . G G    
735	. . . . . . . . . . G G G . . G . G G G G G G G G G G G G G G G G N G G G G G G G G G G G G G G G G G G G G G G G G G G G G G G G G G G G G G G G G G G G G G G G G G . G G G G G G G G G G G G G G G G G G G G G G G G G G G . . G G G G G G G G G G G G G G G G G G G G G G G G G G G G G G . . . G G G G G G . G G G G G G G G . . G G G G G G G G G G G G G G G G G G . . G G    
736	. . . . . . . . . . G G G . . G . G G G G G G G G G G G G G G G G G G G G G G G G G G G G G G G G G G G G G G G G G G G G G G G G G G G G G G G G G G G G G G G G G G . G G G A G G G G G G G G G G G G G G G G G G G G G G G . . G G G G G G G G G G G G G G G G G G G G G G G G G G G G G G . . . G G G G G G . G G G G G G G G . . G G G G G G G G G G G G G G G G G G . . G G    
737	. . . . . . . . . . A A A . . A . A A A A A A A A A A A A A A A A A A A A A A A A A A A A A A A A A A A A A A A A A A A A A A A A A A A A A A A A A A A A A A A A A A . A A A A A A A A A A A A A A A A A A A A A A A A A A A . . A A A A A A A A A A A A A A A A A A A A A A A A A A A A A A . . . A A A A A A . A A A A A A A A . . A A A A A A A A A A A A A A A A A A . . A A    
738	. . . . . . . . . . A A A . . A . A A A A A A A A A A A A A A A A A A A A A A A A A A A A A A A A A A A A A A A A A A A A A A A A A A A A A A A A A A A A A A A A A A . A A A A A A A A A A A A A A A A A A A A A A A A A A A . . A A A A A A A A A A A A A A A A A A A A A A A A A A A A A A . . . A A A A A A . A A A A A A A A . . A A A A A A A A A A A A A A A A A A . . A A    
739	. . . . . . . . . . G G G . . G . G G G G G G G G G G G G G G G G G G G G G G G G G G G G G G G G G G G G G G G G G G G G G G G G G G G G G G G G G G G G G G G G G G . G G G G G G G G G G G G G G G G G G G G G G G G G G G . . G G G G G G G G G G G G G G G G G G G G G G G G G G G G G G . . G G G G G G G . G G G G G G G G . . G G G G G G G G G G G G G G G G G G . . G G    
740	. . . . . . . . . . C C C . . C . C C C C C C C C C C C C C C C C C C C C C C C C C C C C C C C C C C C C C C C C C C C C C C C C C C C C C C C C C C C C C C C C C C . C C C C C C C C C C C C C C C C C C C C C C C C C C C . . C C C C C C C C C C C C C C C C C C C C C C C C C C C C C C . . C C C C C C C . C C C C C C C C . . C C C C C C C C C C C C C C C C C C C C C C    
741	. . . . . . . . . . A A A . . A . A A A A A A A A A A A A A A A A A A A A A A A A A A A A A A A A A A A A A A A A A A A A A A A A A A A A A A A A A A A A A A A A A A . A A A A A A A A A A A A A A A A A A A A A A A A A A A . . A A A A A A A A A A A A A A A A A A A A A A A A A A A A A A . . A A A A A A A . A A A A A A A A . . A A A A A A A A A A A A A A A A A A A A A A    
742	. . . . . . . . . . G G G . . G . G G G G G G G G G G G G G G G G G G G G G G G G G G G G G G G G G G G G G G G G G G G G G G G G G G G G G G G G G G G G G G G G G G . G G G G G G G G G G G G G G G G G G G G G G G G G G G . . G G G G G G G G G G G G G G G G G G G G G G G G G G G G G G . . G G G G G G G . G G G G G G G G . . G G G G G G G G G G G G G G G G G G G G G G    
743	. . . . . . . . . . C C C . . C . C C C C C C C C C C C C C C C C C C C C C C C C C C C C C C C C C C C C C C C C C C C C C C C C C C C C C C C C C C C C C C C C C C . C C C C C C C C C C C C C C C C C C C C C C C C C C C . . C C C C C C C C C C C C C C C C C C C C C C C C C C C C C C . . C C C C C C C . C C C C C C C C . . C C C C C C C C C C C C C C C C C C C C C C    
744	. . . . . . . . . . T T T . . T . T T T T T T T T T T T T T T T T T T T T T T T T T T T T T T T T T T T T T T T T T T T T T T T T T T T T T T T T T T T T T T T T T T . T T T T T T T T T T T T T T T T T T T T T T T T T T T . . T T T T T T T T T T T T T T T T T T T T T T T T T T T T T T . . T T T T T T T . T T T T T T T T . . T T T T T T T T T T T T T T T T T T T T T T    
745	. . . . . . . . . . C C C . . C . C C C C C C C C C C C C C C C C C C C C C C C C C C C C C C C C C C C C C C C C C C C C C C C C C C C C C C C C C C C C C C C C C C . C C C C C C C C C C C C C C C C C C C C C C C C C C C . . C T C C C C C C C C C C C C C C T C C C C C C C C C C C C C . . C C C C C C C . C C C T C C C C . . C C C C C C C C C C C C C C C C C C T C C C    
746	. . . . . . . . . . T T T . . T . T T T T T T T T T T T T T T T T T T T T T T T T T T T T T T T T T T T T T T T T T T T T T T T T T T T T T T T T T T T T T T T T T T . T T T T T T T T T T T T T T T T T T T T T T T T T T T . . T T T T T T T T T T T T T T T T T T T T T T T T T T T T T T . . T T T T T T T . T T T T T T T T . . T T T T T T T T T T T T T T T T T T T T T T    
747	. . . . . . . . . . C C C . . C . C C C C C C C C C C C C C C C C C C C C C C C C C C C C C C C C C C C C C C C C C C C C C C C C C C C C C C C C C C C C C C C C C C . C C C C C C C C C C C C C C C C C C C C C C C C C C C . . C T C C C C C C C C C C C C C C C C C C C C C C C C C C C C . . C C C C C C C . C C C C C C C C . . C C C C C C C C C C C C C C C C C C C C C C    
748	. . . . . . . . . . T T T . . T . T T T T T T T T T T T T T T T T T T T T T T T T T T T T T T T T T T T T T T T T T T T T T T T T T T T T C T T T T T T T T T T T T T . T T T T T T T T T T T T T T T T T T T T T T T T T T T . . T T T T T T T T T T T T T T T T T T T T T T T T T T T T T T . . T T T T T T T . T T T T T T T T . . T T T T T T T C T T T T T T T T T T T T T T    
749	. . . . . . . . . . G G G . . G . G G G G G G G G G G G G G G G G G G G G G G G G G G G G G G G G G G G G G G G G G G G G G G G G G G G G G G G G G G G G G G G G G G . G G G G G G G G G G G G G G G G G G G G G G G G G G G . . G G G G G G G G G G G G G G G G G G G G G G G G G G G G G G . . G G G G G G G . G G G G G G G G . . G G G G G G G G G G G G G G G G G G G G G G    
750	. . . . . . . . . . G G G . . G . G G G G G G G G G G G G G G G G G G G G G G G G G G G G G G G G G G G G G G G G G G G G G G G G G G G G G G G G G G G G G G G G G G . G G G G G G G G G G G G G G G G G G G G G G G G G G G . . G G G G G G G G G G G G G G G G G G G G G G G G G G G G G G . . G G G G G G G . G G G G G G G G . . G G G G G G G G G G G G G G G G G G G G G G    
751	. . . . . . . . . . C C C . . C . C C C C C C C C C C C C C C C C C C C C C T C C C C C C C C C C C C C C C C C C C C C C C C C C C C C C C C C C C C C C C C C C C C . C C C C C C C C C C C C C C C C C C C C C C C C C C C . . C C C C C C C C C C C C C C C C C C C C C C C T C C C C C C . . C C C C C C C . C C C C C C C C . . C C C C C C C C C C C C C C C C C C C C C C    
752	. . . . . . . . . . A A A . . A . A A A A A A A A A A A A A A A A A A A A A A A A A A A A A A A A A A A A A A A A A A A A A A A A A A A A A A A A A A A A A A A A A A . A A A A A A A A A A A A A A A A A A A A A A A A A A A . . A A A A A A A A A A A A A A A A A A A A A A A A A A A A A A . . A A A A A A A . A A A A A A A A . . A A A A A A A A A A A A A A A A A A A A A A    
753	. . . . . . . . . . C C C . . C . C C C C C C C C C C C C C C C C C C C C C C C C C C C C C C C C C C C C C C C C C C C C C C C C C C C C C C C C C C C C C C C C C C . C C C C C C C C C C C C C C C C C C C C C C C C C C C . . C C C C C C C C C C C C C C C C C C C C C C C C C C C C C C . . C C C C C C C . C C C C C C C C . . C C C C C C C C C C C C C C C C C C C C C C    
754	. . . . . . . . . . C C C . . C . C C C C C C C C C C C C C C C C C C C C C C C C C C C C T C C C C C C C C C C C C C C C C C C C C C C C C C C C C C C C C C C C C C . C C C C C C C C C C C C C C C C C C C C C C C C C C C . . C C C C C C C C C C C C C C C C C C C C C C C C C C C C C C . . C C C C C C C . C C C C C C C C . . C C C C C C C C C C C C C C C C C C C C C C    
755	. . . . . . . . . . G G G . . G . G G G G G G G G G G G G G G G G G G G G G G G G G G G G G G G G G G G G G G G G G G G G G G G G G G G G G G G G G G G G G G G G G G . G G G G G G G G G G G G G G G G G G G G G G G G G G G . . G G G G G G G G G G G G G G G G G G G G G G G G G G G G G G . . G G G G G G G . G G G G G G G G . . G G G G G G G G G G G G G G G G G G G G G G    
756	. . . . . . . . . . T T T . . T . T T T T T T T T T T T T T T T T T T T T T T T T T T T T T T T T T T T T T T T T T T T T T T T T T T T T T T T T T T T T T T T T T T . T T T T T T T T T T T T T T T T T T T T T T T T T T T . . T T T T T T T T T T T T T T T T T T T T T T T T T T T T T T . . T T T T T T T . T T T T T T T T . . T T T T T T T T T T T T T T T T T T T T T T    
757	. . . . . . . . . . . A A . . A . A A A A A A A A A A A A A A A A A A A A A A A A A A A A A A A A A A A A A A A A A A A A A A A A A A A A A A A A A A A A A A A A A A . A A A A A A A A A A A A A A A A A A A A A A A A A A A . . A A A A A A A A A A A A A A A A A A A A A A A A A A A A A A . . A A A A A A A . A A A A A A A A . . A A A A A A A A A A A A A A A A A A A A A A    
758	. . . . . . . . . . . A A . . A . A A A A A A A A A A A A A A A A A A A A A A A A A A A A A A A A A A A A A A A A A A A A A A A A A A A A A A A A A A A A A A A A A A . A A A A A A A A A A A A A A A A A A A A A A A A A A A . . A A A A A A A A A A A A A A A A A A A A A A A A A A A A A A . . A A A A A A A . A A A A A A A A . . A A A A A A A A A A A A A A A A A A A A A A    
759	. . . . . . . . . . . C C . . C . C C C C C C C C C C C C C C C C C C C C C C C C C C C C C C C C C C C C C C C C C C C C C C C C C C C C C C C C C C C C C C C C C C . C C C C C C C C C C C C C C C C C C C C C C C C C C C . . C C C C C C C C C C C C C C C C C C C C C C C C C C C C C C . . C C C C C C C . C C C C C C C C . . C C C C C C C C C C C C C C C C C C C C C C    
760	. . . . . . . . . . . C C . . C . C C C C C C C C C C C C C C C C C C C C C C C C C C C C C C C C C C C C C C C C C C C C C C C C C C C C C C C C C C C C C C C C C C . C C C C C C C C C C C C C C C C C C C C C C C C C C C . . C C C C C C C C C C C C C C C C C C C C C C C C C C C C C C . . C C C C C C C . C C C C C C C C . . C C C C C C C C C C C C C C C C C C C C C C    
761	. . . . . . . . . . . C A . . A . A A A A A A A A A A A A A A A A A A A A A A A A A A A A A A A A A A A A A A A A A A A A A A A A A A A A A A A A A A A A A A A A A A . A A A A A A A A A A A A A A A A A A A A A A A A A A A . . A A A A A A A A A A A A A A A A A A A A A A A A A A A A A A . . A A A A A A A . A A A A A A A A . . A A A A A A A A A A A A A A A A A A A A A A    
762	. . . . . . . . . . . . G . . G . G G G G G G G G G G G G G G G G G G G G G G G G G G G G G G G G G G G G G G G G G G G G G G G G G G G G G G G G G G G G G G G G G G . G G G G G G G G G G G G G G G G G G G G G G G G G G G . . G G G G G G G G G G G G G G G G G G G G G G G G G G G G G G . . G G G G G G G . G G G G G G G G . . G G G G G G G G G G G G G G G G G G G G G G    
763	. . . . . . . . . . . . T . . T . T T T T T T T T T T T T T T T T T T T T T T T T T T T T T T T T T T T T T T T T T T T T T T T T T T T T T T T T T T T T T T T T T T . T T T T T T T T T T T T T T T T T T T T T T T T T T T . . T T T T T T T T T T T T T T T T T T T T T T T T T T T T T T . . T T T T T T T . T T T T T T T T . . T T T T T T T T T T T T T T T T T T T T T T    
764	. . . . . . . . . . . . G . . G . G G G G G G G G G G G G G G G G G G G G G G G G G G G G G G G G G G G G G G G G G G G G G G G G G G G G G G G G G G G G G G G G G G . G G G G G G G G G G G G G G G G G G G G G G G G G G G . . G G G G G G G G G G G G G G G G G G G G G G G G G G G G G G . . G G G G G G G . G G G G G G G G . . G G G G G G G G G G G G G G G G G G G G G G    
765	. . . . . . . . . . . . G . . G . G G G G G G G G G G G G G G G G G G G G G G G G G G G G G G G G G G G G G G G G G G G G G G G G G G G G G G G G G G G G G G G G G G . G G G G G G G G G G G G G G G G G G G G G G G G G G G . . G G G G G G G G G G G G G G G G G G G G G G G G G G G G G G . . G G G G G G G . G G G G G G G G . . G G G G G G G G G G G G G G G G G G G G G G    
766	. . . . . . . . . . . . G . . G . G G G G G G G G G G G G G G G G G G G G G G G G G G G G G G G G G G G G G G G G G G G G G G G G G G G G G G G G G G G G G G G G G G . G G G G G G G G G G G G G G G G G G G G G G G G G G G . . G G G G G G G G G G G G G G G G G G G G G G G G G G G G G G . . G G G G G G G . G G G G G G G G . . G G G G G G G G G G G G G G G G G G G G G G    
767	. . . . . . . . . . . . C . . C . C C C C C C C C C C C C C C C C C C C C C C C C C C C C C C C C C C C C C C C C C C C C C C C C C C C C C C C C C C C C C C C C C C . C C C C C C C C C C C C C C C C C C C C C C C C C C C . . C C C C C C C C C C C C C C C C C C C C C C C C C C C C C C . . C C C C C C C . C C C C C C C C . . C C C C C C C C C C C C C C C C C C C C C C    
768	. . . . . . . . . . . . C . . C . C C C C C C C C C C C C C C C C C C C C C C C C C C C C C C C C C C C C C C C C C C C C C C C C C C C C C C C C C C C C C C C C C C . C C C C C C C C C C C C C C C C C C C C C C C C C C C . . C C C C C C C C C C C C C C C C C C C C C C C C C C C C C C . . C C C C C C C . C C C C C C C C . . C C C C C C C C C C C C C C C C C C C C C C    
769	. . . . . . . . . . . . T . . T . T T T T T T T T T T T T T T T T T T T T T T T T T T T T T T T T T T T T T T T T T T T T T T T T T T T T T T T T T T T T T T T T T T . T T T T T T T T T T T T T T T T T T T T T T T T T T T . . T T T T T T T T T T T T T T T T T T T T T T T T T T T T T T . . T T T T T T T . T T T T T T T T . . T T T T T T T T T T T T T T T T T T T T T T    
770	. . . . . . . . . . . . . . . G . G G G G G G G G G G G G G G G G G G G G G G G G G G G G G G G G G G G G G G G G G G G G G G G G G G G G G G G G G G G G G G G G G G . G G G G G G G G G G G G G G G G G G G G G G G G G G G . . G G G G G G G G G G G G G G G G G G G G G G G G G G G G G G . . G G G G G G G . G G G G G G G G . . G G G G G G G G G G G G G G G G G G G G G G    
771	. . . . . . . . . . . . . . . C . C C C C C C C C C C C C C C C C C C C C C C C C C C C C C C C C C C C C C C C C C C C C C C C C C C C C C C C C C C C C C C C C C C . C C C C C C C C C C C C C C C C C C C C C C C C C C C . . C C C C C C C C C C C C C C C C C C C C C C C C C C C C C C . . C C C C C C C . C C C C C C C C . . C C C C C C C C C C C C C C C C C C C C C C    
772	. . . . . . . . . . . . . . . T . T T T T T T T T T T T T T T T T T T T T T T T T T T T T T T T T T T T T T T T T T T T T T T T T T T T T T T T T T T T T T T T T T T . T T T T T T T T T T T T T T T T T T T T T T T T T T T . . T T T T T T T T T T T T T T T T T T T T T T T T T T T T T T . . T T T T T T T . T T T T T T T T . . T T T T T T T T T T T T T T T T T T T T T T    
773	. . . . . . . . . . . . . . . A . A A A A A A A A A A A A A A A A A A A A A A A A A A A A A A A A A A A A A A A A A A A A A A A A A A A A A A A A A A A A A A A A A A . A A A A A A A A A A A A A A A A A A A A A A A A A A A . . A A A A A A A A A A A A A A A A A A A A A A A A A A A A A A . . A A A A A A A . A A A A A A A A . . A A A A A A A A A A A A A A A A A A A A A A    
774	. . . . . . . . . . . . . . . G . G G G G G G G G G G G G G G G G G G G G G G G G G G G G G G G G G G G G G G G G G G G G G G G G G G G G G G G G G G G G G G G G G G . G G G G G G G G G G G G G G G G G G G G G G G G G G G . . G G G G G G G G G G G G G G G G G G G G G G G G G G G G G G . . G G G G G G G . G G G G G G G G . . G G G G G G G G G G G G G G G G G G G G G G    
775	. . . . . . . . . . . . . . . G . G G G G G G G G G G G G G G G G G G G G G G G G G G G G G G G G G G G G G G G G G G G G G G G G G G G G G G G G G G G G G G G G G G . G G G G G G G G G G G G G G G G G G G G G G G G G G G . . G G G G G G G G G G G G G G G G G G G G G G G G G G G G G G . . G G G A G A G . G G G G G G G G . . G G G G G G G G G G G G G G G G G G G G G G    
776	. . . . . . . . . . . . . . . T . T T T T T T T T T T T T T T T T T T T T T T T T T T T T T T T T T T T T T T T T T T T T T T T T T T T T T T T T T T T T T T T T T T . T T T T T T T T T T T T T T T T T T T T T T T T T T T . . T T T T T T T T T T T T T T T T T T T T T T T T T T T T T T . . T T T T T T T . T T T T T T T T . . T T T T T T T T T T T T T T T T T T T T T T    
777	. . . . . . . . . . . . . . . G . G G G G G G G G G G G G G G G G G G G G G G G G G G G G G G G G G G G G G G G G G G G G G G G G G G G G G G G G G G G G G G G G G G . G G G G G G G G G G G G G G G G G G G G G G G G G G G . . G G G G G G G G G G G G G G G G G G G G G G G G G G G G G G . . G G G G G G G . G G G G G G G G . . G G G G G G G G G G G G G G G G G G G G G G    
778	. . . . . . . . . . . . . . . . . C C C C C C C C C C C C C C C C C C C C C C C C C C C C C C C C C C C C C C C C C C C C C C C C C C C C C C C C C C C C C C C C C C . C C C C C C C C C C C C C C C C C C C C C C C C C C C . . C C C C C C C C C C C C C C C C C C C C C C C C C C C C C C . . C C C C C C C . C C C C C C C C . . C C C C C C C C C C C C C C C C C C C C C C    
779	. . . . . . . . . . . . . . . . . G G G G G G G G G G G G G G G G G G G G G G G G G G G G G G G G G G G G G G G G G G G G G G G G G G G G G G G G G G G G G G G G G G . G G G G G G G G G G G G G G G G G G G G G G G G G G G . . G G G G G G G G G G G G G G G G G G G G G G G G G G G G G G . . G G G G G G G . G G G G G G G G . . G G G G G G G G G G G G G G G G G G G G G G    
780	. . . . . . . . . . . . . . . . . G G G G G G G G G G G G G G G G G G G G G G G G G G G G G G G G G G G G G G G G G G G G G G G G G G G G G G G G G G G G G G G G G G . G G G G G G G G G G G G G G G G G G G G G G G G G G G . . G G G G G G G G G G G G G G G G G G G G G G G G G G G G G G . . G G G G G G G . G G G G G G G G . . G G G G G G G G G G G G G G G G G G G G G G    
781	. . . . . . . . . . . . . . . . . C C C C C C C C C C C C C C C C C C C C C C C C C C C C C C C C C C C C C C C C C C C C C C C C C C C C C C C C C C C C C C C C C C . C C C C C C C C C C C C C C C C C C C C C C C C C C C . . C C C C C C C C C C C C C C C C C C C C C C C C C C C C C C . . C C C C C C C . C C C C C C C C . . C C C C C C C C C C C C C C C C C C C C C C    
782	. . . . . . . . . . . . . . . . . A A A A A A A A A A A A A A A A A A A A A A A A A A A A A A A A A A A A A A A A A A A A A A A A A A A A A A A A A A A A A A A A A A . A A A A A A A A A A A A A A A A A A A A A A A A A A A . . A C A A A A A A A A A A A A A A A A A A A A A A A A A A A A . . A A A A A A A . A A A A A A A A . . A A A A A A A A A A A A A A A A A A A A A A    
783	. . . . . . . . . . . . . . . . . C C C C C C C C C C C C C C C C C C C C C C C C C C C C C C C C C C C C C C C C C C C C C C C C C C C C C C C C C C C C C C C C C C . C C C C C C C C C C C C C C C C C C C C C C C C C C C . . C C C C C C C C C C C C C C C C C C C C C C C C C C C C C C . . C C C C C C C . C C C C C C C C . . C C C C C C C C C C C C C C C C C C C C C C    
784	. . . . . . . . . . . . . . . . . C C C C C C C C C C C C C C C C C C C C C C C C C C C C C C C C C C C C C C C C C C C C C C C C C C C C C C C C C C C C C C C C C C . C C C C C C C C C C C C C C C C C C C C C C C C C C C . . C C C C C C C C C C C C C C C C C C C C C C C C C C C C C C . . C C C C C C C . C C C C C C C C . . C C C C C C C C C C C C C C C C C C C C C C    
785	. . . . . . . . . . . . . . . . . A A A A A A A A A A A A A A A A A A A A A A A A A A A A A A A A A A A A A A A A A A A A A A A A A A A A A A A A A A A A A A A A A A . A A A A A A A A A A A A A A A A A A A A A A A A A A A . . A A A A A A A A A A A A A A A A A A A A A A A A A A A A A A . . A A A A A A A . A A A A A A A A . . A A A A A A A A A A A A A A A A A A A A A A    
786	. . . . . . . . . . . . . . . . . C C C C C C C C C C C C C C C C C C C C C C C C C C C C C C C C C C C C C C C C C C C C C C C C C C C C C C C C C C C C C C C C C C . C C C C C C C C C C C C C C C C C C C C C C C C C C C . C C C C C C C C C C C C C C C C C C C C C C C C C C C C C C C . . C C C C C C C . C C C C C C C C . . C C C C C C C C C C C C C C C C C C C C C C    
787	. . . . . . . . . . . . . . . . . A A A A A A A A A A A A A A A A A A A A A A A A A A A A A A A A A A A A A A A A A A A A A A A A A A A A A A A A A A A A A A A A A A . A A A A A A A A A A A A A A A A A A A A A A A A A A A . A A A A A A A A A A A A A A A A A A A A A A A A A A A A A A A . . A A A A A A A . A A A A A A A A . . A A A A A A A A A A A A A A A A A A A A A A    
788	. . . . . . . . . . . . . . . . . T T T T T T T T T T T T T T T T T T T T T T T T T T T T T T T T T T T T T T T T T T T T T T T T T T T T T T T T T T T T T T T T T T . T T T T T T T T T T T T T T T T T T T T T T T T T T T . T T T T T T T T T T T T T T T T T T T T T T T T T T T T T T T . . T T T T T T T . T T T T T T T T . . T T T T T T T T T T T T T T T T T T T T T T    
789	. . . . . . . . . . . . . . . . . C C C C C C C C C C C C C C C C C C C C C C C C C C C C C C C C C C C C C C C C C C C C C C C C C C C C C C C C C C C C C C C C C C . C C C C C C C C C C C C C C C C C C C C C C C C C C C . C C C C C C C C C C C C C C C C C C C C C C C C C C C C C C C . . C C C C C C C . C C C C C C C C . . C C C C C C C C C C C C C C C C C C C C C C    
790	. . . . . . . . . . . . . . . . . C C C C C C C C C C C C C C C C C C C C C C C C C C C C C C C C C C C C C C C C C C C C C C C C C C C C C C C C C C C C C C C C C C . C C C C C C C C C C C C C C C C C C C C C C C C C C C . C C C C C C C C C C C C C C C C C C C C C C C C C C C C C C C . . C C C C C C C . C C C C C C C C . . C C C C C C C C C C C C C C C C C C C C C C    
791	. . . . . . . . . . . . . . . . . G G G G G G G G G G G G G G G G G G G G G G G G G G G G G G G G G G G G G G G G G G G G G G G G G G G G G G G G C G G G G G G G G G . G G G G G G G G G G G G G G G G G G G G G G G G G G G . G G G G G G G G G G G G G G G G G G G G G G G G G G G G G G G . . G G G G G G G . G G G G G G G G . . G G C G G G G G G G G G G G G G G G G G G G    
792	. . . . . . . . . . . . . . . . . C C C C C C C C C C C C C C C C C C C C C C C C C C C C C C C C C C C C C C C C C C C C C C C C C C C C C C C C C C C C C C C C C C . C C C C C C C C C C C C C C C C C C C C C C C C C C C . C C C C C C C C C C C C C C C C C C C C C C C C C C C C C C C . . C C C C C C C . C C C C C C C C . . C C C C C C C C C C C C C C C C C C C C C C    
793	. . . . . . . . . . . . . . . . . A A A A A A A A A A A A A C A A A A A A A A A A A A A A A A A A A A A A A A A A A A A A A A A A A A A A A A A A A A A A A A A A A A . A A A A A A A A A A A A A A A A A A A A A A A A A A A . A A A A A A A A A A A A A A A A A A A A A A A A A A A A A A A . . A A A A A A A . A A A A A A A A . . A A A A A A A A A A A A A A A A A A A A A A    
794	. . . . . . . . . . . . . . . . . C C C C C C C C C C C C C C C C C C C C C C C C C C C C C C C C C C C C C C C C C C C C C C C C C C C C C C C C C C C C C C C C C C . C C C C C C C C C C C C C C C C C C C C C C C C C C C . C C C C C C C C C C C C C C C C C C C C C C C C C C C C C C C . . C C C C C C C . C C C C C C C C . . C C C C C C C C C C C C C C C C C C C C C C    
795	. . . . . . . . . . . . . . . . . T T T T T T T T T T T T T T T T T T T T T T T T T T T T T T T T T T T T T T T T T T T T T T T T T T T T T T T T T T T T T T T T T T . T T T T T T T T T T T T T T T T T T T T T T T T T T T . T T T T T T T T T T T T T T T T T T T T T T T T T T T T T T T . . T T T T T T T . T T T T T T T T . . T T T T T T T T T T T T T T T T T T T T T T    
796	. . . . . . . . . . . . . . . . . C C C C C C C C C C C C C C C C C C C C C C C C C C C C C C C C C C C C C C C C C C C C C C C C C C C C C C C C C C C C C C C C C C . C C C C C C C C C C C C C C C C C C C C C C C C C C C . C C C C C C C C C C C C C C C C C C C C C C C C C C C C C C C . . C C C C C C C . C C C C C C C C . . C C C C C C C C C C C C C C C C C C C C C C    
797	. . . . . . . . . . . . . . . . . A A A A A A A A A A A A A A A A A A A A A A A A A A A A A A A A A A A A A A A A A A A A A A A A A A A A A A A A A A A A A A A A A A . A A A A A A A A A A A A A A A A A A A A A A A A A A A . A A A A A A A A A A A A A A A A A A A A A A A A A A A A A A A . . A A A A A A A . A A A A A A A A . . A A A A A A A A A A A A A A A A A A A A A A    
798	. . . . . . . . . . . . . . . . . A A A A A A A A A A A A A A A A A A A A A A A A A A A A A A A A A A A A A A A A A A A A A A A A A A A A A A A A A A A A A A A A A A . A A A A A A A A A A A A A A A A A A A A A A A A A A A . A A A A A A A A A A A A A A A A A A A A A A A A A A A A A A A . . A A A A A A A . A A A A A A A A . . A A A A A A A A A A A A A A A A A A A A A A    
799	. . . . . . . . . . . . . . . . . T T T T T T T T T T T T T T T T T T T T T T T T T T T T T T T T T T T T T T T T T T T T T T T T T T T T T T T T T T T T T T T T T T . T T T T T T T T T T T T T T T T T T T T T T T T T T T . T T T T T T T T T T T T T T T T T T T T T T T T T T T T T T T . . T T T T T T T . T T T T T T T T . . T T T T T T T T T T T T T T T T T T T T T T    
800	. . . . . . . . . . . . . . . . . C C C C C C C C C C C C C C C C C C C C C C C C C C C C C C C C C C C C C C C C C C C C C C C C C C C C C C C C C C C C C C C C C C . C C C C C C C C C C C C C C C C C C C C C C C C C C C . C C C C C C C C C C C C C C C C C C C C C C C C C C C C C C C . . C C C C C C C . C C C C C C C C . . C C C C C C C C C C C C C C C C C C C C C C    
801	. . . . . . . . . . . . . . . . . A A A A A A A A A A A A A A A A A A A A A A A A A A A A A A A A A A A A A A A A A A A A A A A A A A A A A A A A A A A A A A A A A A . A A A A A A A A A A A A A A A A A A A A A A A A A A A . A A A A A A A A A A A A A A A A A A A A A A A A A A A A A A A . . A A A A A A A . A A A A A A A A . . A A A A A A A A A A A A A A A A A A A A A A    
802	. . . . . . . . . . . . . . . . . T T T T T T T T T T T T T T T T T T T T T T T T T T T T T T T T T T T T T T T T T T T T T T T T T T T T T T T T T T T T T T T T T T . T T T T T T T T T T T T T T T T T T T T T T T T T T T . T T T T T T T T T T T T T T T T T T T T T T T T T T T T T T T . . T T T T T T T . T T T T T T T T . . T T T T T T T T T T T T T T T T T T T T T T    
803	. . . . . . . . . . . . . . . . . G G G G G G G G G G G G G G G G G G G G G G G G G G G G G G G G G G G G G G G G G G G G G G G G G G G G G G G G G G G G G G G G G G . G G G G G G G G G G G G G G G G G G G G G G G G G G G . G G G G G G G G G G G G G G G G G G G G G G G G G G G G G G G . . G G G G G G G . G G G G G G G G . . G G G G G G G G G G G G G G G G G G G G G G    
804	. . . . . . . . . . . . . . . . . T T T T T T T T T T T T T T T T T T T T T T T T T T T T T T T T T T T T T T T T T T T T T T T T T T T T T T T T T T T T T T T T T T . T T T T T T T T T T T T T T T T T T T T T T T T T T T . T T T T T T T T T T T T T T T T T T T T T T T T T T T T T T T . . T T T T T T T . T T T T T T T T . . T T T T T T T T T T T T T T T T T T T T T T    
805	. . . . . . . . . . . . . . . . . G G G G G G G G G G G G G G G G G G G G G G G G G G G G G G G G G G G G G G G G G G G G G G G G G G G G G G G G G G G G G G G G G G . G G G G G G G G G G G G G G G G G G G G G G G G G G G . G G G G G G G G G G G G G G G G G G G G G G G G G G G G G G G . . G G G G G G G . G G G G G G G G . . G G G G G G G G G G G G G G G G G G G G G G    
806	. . . . . . . . . . . . . . . . . A A A A A A A A A A A A A A A A A A A A A A A A A A A A A A A A A A A A A A A A A A A A A A A A A A A A A A A A A A A A A A A A A A . A A A A A A A A A A A A A A A A A A A A A A A A A A A . A A A A A A A A A A A A A A A A A A A A A A A A A A A A A A A . . A A A A A A A . A A A A A A A A . . A A A A A A A A A A A A A A A A A A A A A A    
807	. . . . . . . . . . . . . . . . . T T T T T T T T T T T T T T T T T T T T T T T T T T T T T T T T T T T T T T T T T T T T T T T T T T T T T T T T T T T T T T T T T T . T T T T T T T T T T T T T T T T T T T T T T T T T T T . T T T T T T T T T T T T T T T T T T T T T T T T T T T T T T T . . T T T T T T T . T T T T T T T T . . T T T T T T T T T T T T T T T T T T T T T T    
808	. . . . . . . . . . . . . . . . . G G G G G G G G G G G G G G G G G G G G G G G G G G G G G G G G G G G G G G G G G G G G G G G G G G G G G G G G G G G G G G G G G G . G G G G G G G G G G G G G G G G G G G G G G G G G G G . G G G G G G G G G G G G G G G G G G G G G G G G G G G G G G G . . G G G G G G G . G G G G G G G G . . G G G G G G G G G G G G G G G G G G G G G G    
809	. . . . . . . . . . . . . . . . . G G G G G G G G G G G G G G G G G G G G G G G G G G G G G G G G G G G G G G G G G G G G G G G G G G G G G G G G G G G G G G G G G G . G G G G G G G G G G G G G G G G G G G G G G G G G G G . G G G G G G G G G G G G G G G G G G G G G G G G G G G G G G G . . G G G G G G G . G G G G G G G G . . G G G G G G G G G G G G G G G G G G G G G G    
810	. . . . . . . . . . . . . . . . . C C C C C C C C C C C C C C C C C C C C C C C C C C C C C C C C C C C C C C C C C C C C C C C C C C C C C C C C C C C C C C C C C C . C C C C C C C C C C C C C C C C C C C C C C C C C C C . C C C C C C C C C C C C C C C C C C C C C C C C C C C C C C C . . C C C C C C C . C C C C C C C C . . C C C C C C C C C C C C C C C C C C C C C C    
811	. . . . . . . . . . . . . . . . . C C C C C C C C C C C C C C C C C C C C C C C C C C C C C C C C C C C C C C C C C C C C C C C C C C C C C C C C C C C C C C C C C C . C C C C C C C C C C C C C C C C C C C C C C C C C C C . C C C C C C C C C C C C C C C C C C C C C C C C C C C C C C C . . C C C C C C C . C C C C C C C C . . C C C C C C C C C C C C C C C C C C C C C C    
812	. . . . . . . . . . . . . . . . . A A A A A A A A A A A A A A A A A A A A A A A A A A A A A A A A A A A A A A A A A A A A A A A A A A A A A A A A A A A A A A A A A A . A A A A A A A A A A A A A A A A A A A A A A A A A A A . A A A A A A A A A A A A A A A A A A A A A A A A A A A A A A A . . A A A A A A A . A A A A A A A A . . A A A A A A A A A A A A A A A A A A A A A A    
813	. . . . . . . . . . . . . . . . . T T T T T T T T T T T T T T T T T T T T T T T T T T T T T T T T T T T T T T T T T T T T T T T T T T T T T T T T T T T T T T T T T T . T T T T T T T T T T T T T T T T T T T T T T T T T T T . T T T T T T T T T T T T T T T T T T T T T T T T T T T T T T T . . T T T T T T T . T T T T T T T T . . T T T T T T T T T T T T T T T T T T T T T T    
814	. . . . . . . . . . . . . . . . . A A A A A A A A A A A A A A A A A A A A A A A A A A A A A A A A A A A A A A A A A G A A A A A A A A A A A A A A A A A A A A A A A A . A A A A A A A A A A A A A A A A A A A A A A A A A A G . A A A A A A A A A A A A A A A A A A A A A A A A A A A A A A A . . A A A A A A A . A A A A A A A A . . A A A A A A A A A A A A A A A A A A C A A A    
815	. . . . . . . . . . . . . . . . . C C C C C C C C C C C C C C C C C C C C C C C C C C C C C C C C C C C C C C C C C C C C C C C C C C C C C C C C C C C C C C C C C C . C C C C C C C C C C C C C C C C C C C C C C C C C C C . C C C C C C C C C C C C C C C C C C C C C C C C C C C C C C C . . C C C C C C C . C C C C C C C C . . C C C C C C C C C C C C C C C C C C C C C C    
816	. . . . . . . . . . . . . . . . . C C C C C C C C C C C C C C C C C C C C C C C C C C C C C C C C C C C C C C C C C C C C C C C C C C C C C C C C C C C C C C C C C C . C C C C C C C C C C C C C C C C C C C C C C C C C C C . C C C C C C C C C C C C C C C C C C C C C C C C C C C C C C C . . C C C C C C C . C C C C C C C C . . C C C C C C C C C C C C C C C C C C C C C C    
817	. . . . . . . . . . . . . . . . . T T T T T T T T T T T T T T T T T T T T T T T T T T T T T T T T T T T T T T T T T T T T T T T T T T T T T T T T T T T T T T T T T T . T T T T T T T T T T T T T T T T T T T T T T T T T T T . T T T T T T T T T T T T T T T T T T T T T T T T T T T T T T T . . T T T T T T T . T T T T T T T T . . T T T T T T T T T T T T T T T T T T T T T T    
818	. . . . . . . . . . . . . . . . . G G G G G G G G G G G G G G G G G G G G G G G G G G G G G G G G G G G G G G G G G G G G G G G G G G G G G G G G G G G G G G G G G G . G G G G G G G G G G G G G G G G G G G G G G G G G G G . G G G G G G G G G G G G G G G G G G G G G G G G G G G G G G G . . G G G G G G G . G G G G G G G G . . G G G G G G G G G G G G G G G G G G G G G G    
819	. . . . . . . . . . . . . . . . . A A A A A A A A A A A A A A A A A A A A A A A A A A A A A A A A A A A A A A A A A A A A A A A A A A A A A A A A A A A A A A A A A A . A A A A A A A A A A A A A A A A A A A A A A A A A A A . A A A A A A A A A A A A A A A A A A A A A A A A A A A A A A A . . A A A A A A A . A A A A A A A A . . A A A A A A A A A A A A A A A A A A A A A A    
820	. . . . . . . . . . . . . . . . . T T T T T T T T T T T T T T T T T T T T T T T T T T T T T T T T T T T T T T T T T T T T T T T T T T T T T T T T T T T T T T T T T T . T T T T T T T T T T T T T T T T T T T T T T T T T T T . T T T T T T T T T T T T T T T T T T T T T T T T T T T T T T T . . T T T T T T T . T T T T T T T T . . T T T T T T T T T T T T T T T T T T T T T T    
821	. . . . . . . . . . . . . . . . . G G G G G G G G G G G G G G G G G G G G G G G G G G G G G G G G G G G G G G G G G G G G G G G G G G G G G G G G G G G G G G G G G G . G G G G G G G G G G G G G G G G G G G G G G G G G G G . G G G G G G G G G G G G G G G G G G G G G G G G G G G G G G G G . G G G G G G G . G G G G G G G G . . G G G G G G G G G G G G G G G G G G G G G G    
822	. . . . . . . . . . . . . . . . . T T T T T T T T T T T T T T T T T T T T T T T T T T T T T T T T T T T T T T T T T T T T T T T T T T T T T T T T T T T T T T T T T T . T T T T T T T T T T T T T T T T T T T T T T T T T T T . T T T T T T T T T T T T T T T T T T T T T T T T T T T T T T T T . T T T T T T T . T T T T T T T T . . T T T T T T T T T T T T T T T T T T T T T T    
823	. . . . . . . . . . . . . . . . . G G G G G G G G G G G G G G G G G G G G G G G G G G G G G G G G G G G G G G G G G G G G G G G G G G G G G G G G G G G G G G G G G G . G G G G G G G G G G G G G G G G G G G G G G G G G G G . G G G G G G G G G G G G G G G G G G G G G G G G G G G G G G G G . G G G G G G G . G G G G G G G G . . G G G G G G G G G G G G G G G G G G G G G G    
824	. . . . . . . . . . . . . . . . . A A A A A A A A A A A A A A A A A A A A A A A A A A A A A A A A A A A A A A A A A A A A A A A A A A A A A A A A A A A A A A A A A A . A A A A A A A A A A A A A A A A A A A A A A A A A A A . A A A A A A A A A A A A A A A A A A A A A A A A A A A A A A A A . A A A A A A A . A A A A A A A A . . A A A A A A A A A A A A A A A A A A A A A A    
825	. . . . . . . . . . . . . . . . . T T T . T T T T T T T T T T T T T T T T T T T T T T T T T T T T T T T T T T T T T T T T T T T T T T T T T T T T T T T T T T T T T T . T T T T T T T T T T T T T T T T T T T T T T T T T T T . T T T T T T T T T T T T T T T T T T T T T T T T T T T T T T T T . T T T T T T T . T T T T T T T T . . T T T T T T T T T T T T T T T T T T T T T T    
826	. . . . . . . . . . . . . . . . . G G G . G G G G G G G G A G G G G G G G G G G G G G G G G G G G G G G G G G G G G G G G G G G G G G G G G G G G G G G G G G G G G G . G G G G G G G G G G G G G G G G G G G G G G G G G G G . G G G G G G G G G G G G G G G G G G G G G G G G G G G G G G G G . G G G G A G G . G G G G G G G G . . G G G G G G G G G G G G G G G G G G G G G G    
827	. . . . . . . . . . . . . . . . . C C C . C C C C C C C C C C C C C C C C C C C C C C C C C C C C C C C C C C C C C C C C C C C C C C C C C C C C C C C C C C C C C C . C C C C C C C C C C C C C C C C C C C C C C C C C C C . C C C C C C C C C C C C C C C C C C C C C C C C C C C C C C C C . C C C C C C C . C C C C C C C C . . C C C C C C C C C C C C C C C C C C C C C C    
828	. . . . . . . . . . . . . . . . . T T T . . T T T T T T T T T T T T T T T T T T T T T T T T T T T T T T T T T T T T T T T T T T T T T T T T T T T T T T T T T T T T T . T T T T T T T T T T T T T T T T T T T T T T T T T T T . T T T T T T T T T T T T T T T T T T T T T T T T T T T T T T T T T T T T T T T T . T T T T T T T T . . T T T T T T T T T T T T T T T T T T T T T T    
829	. . . . . . . . . . . . . . . . . G G G . . G G G G G G G G G G G G G G G G G G G G G G G G G G G G G G G G G G G G G G G G G G G G G G G G G G G G G G G G G G G G G . G G G G G G G G G G G G G G G G G G G G G G G G G G G . G G G G G G G G G G G G G G G G G G G G G G G G G G G G G G G G G G G G G G G G . G G G G G G G G . . G G G G G G G G G G G G G G G G G G G G G G    
830	. . . . . . . . . . . . . . . . . A A . . . A A A A A A A A A A A A A A A A A A A A A A A A A A A A A A A A A A A A A A A A A A A A A A A A A A A A A A A A A A A A A . A A A A A A A A A A A A A A A A A A A A A A A A A A A . A A A A A A A A A A A A A A A A A A A A A A A A A A A A A A A A A A A A A A A A . A A A A A A A A . . A A A A A A A A A A A A A A A A A A A A A A    
831	. . . . . . . . . . . . . . . . . G G . . . G G G G G G G G G G G G G G G G G G G G G G G G G G G G G G G G G G G G G G G G G G G G G G G G G G G G G G G G G G G N G . G G G G G G G G G G G G G G G G G G G G G G G G G G G . G G G G G G G G G G G G G G G G G G G G G G G G G G G G G G G G G G G G G G G G . G G G G G G G G . . G G G G G G G G G G G G G G G G G G G G G G    
832	. . . . . . . . . . . . . . . . . T . . . . T T T T T T T T T T T T T T T T T T T T T T T T T T T T T T T T T T T T T T T T T T T T T T T T T T T T T T T T T T T T T . T T T T T T T T T T T T T T T T T T T T T T T T T T T . T T T T T T T T T T T T T T T T T T T T T T T T T T T T T T T T T T T T T T T T . T T T T T T T T . . T T T T T T T T T T T T T T T T T T T T T T    
833	. . . . . . . . . . . . . . . . . G . . . . G G G G G G G G G G G G G G G G G G G G G G G G G G G G G G G G G G G G G G G G G G G G G G G G G G G G G G G G G G G G G . G G G G G G G G G G G G G G G G G G G G G G G G G G G . G G G G G G G G G G G G G G G G G G G G G G G G G G G G G G G G G G G G G G G G . G G G G G G G G . . G G G G G G G G G G G G G G G G G G G G G G    
834	. . . . . . . . . . . . . . . . . A . . . . A A A A A A A A A A A A A A A A A A A A A A A A A A A A A A A A A A A A A A A A A A A A A A A A A A A A A A A A A A A A A . A A A A A A A A A A A A A A A A A A A A A A A A A A A . A A A A A A A A A A A A A A A A A A A A A A A A A A A A A A A A A A A A A A A A . A A A A A A A A . . A A A A A A A A A A A A A A A A A A A A A A    
835	. . . . . . . . . . . . . . . . . T . . . . T T T T T T T T T T T T T T T T T T T T T T T T T T T T T T T T T T T T T T T T T T T T T T T T T T T T T T T T T T T T T . T T T T T T T T T T T T T T T T T T T T T T T T T T T . T T T T T T T T T T T T T T T T T T T T T T T T T T T T T T T T T T T T T T T T . T T T T T T T T . . T T T T T T T T T T T T T T T T T T T T T T    
836	. . . . . . . . . . . . . . . . . G . . . . G G G G G G G G G G G G G G G G G G G G G G G G G G G G G G G G G G G G G G G G G G G G G G G G G G G G G G G G G G G G G . G G G G G G G G G G G G G G G G G G G G G G G G G G G . G G G G G G G G G G G G G G G G G G G G G G G G G G G G G G G G G G G G G G G G . G G G G G G G G . . G G G G G G G G G G G G G G G G G G G G G G    
837	. . . . . . . . . . . . . . . . . A . . . . A A A A A A A A A A A A A A A A A A A A A A A A A A A A A A A A A A A A A A A A A A A A A A A A A A A A A A A A A A A A A . A A A A A A A A A A A A A A A A A A A A A A A A A A A . A A A A A A A A A A A A A A A A A A A A A A A A A A A A A A A A A A A A A A A A . A A A A A A A A . . A A A A A A A A A A A A A A A A A A A A A A    
838	. . . . . . . . . . . . . . . . . . . . . . G G G G G G G G G G G G G G G G G G G G G G G G G G G G G G G G G G G G G G G G G G G G G G G G G G G G G G G G G G G G G . G G G G G G G G G G G G G G G G G G G G G G G G G G G . G G G G G G G G G G G G G G G G G G G G G G G G G G G G G G G G G G G G G G G G . G G G G G G G G . . G G G G G G G G G G G G G G G G G G G G G G    
839	. . . . . . . . . . . . . . . . . . . . . . T T T T T T T T T T T T T T T T T T T T T T T T T T T T T T T T T T T T T T T T T T T T T T T T T T T T T T T T T T T T T . T T T T T T T T T T T T T T T T T T T T T T T T T T T . T T T T T T T T T T T T T T T T T T T T T T T T T T T T T T T T T T T T T T T T . T T T T T T T T . . T T T T T T T T T T T T T T T T T T T T T T    
840	. . . . . . . . . . . . . . . . . . . . . . A A A A A A A A A A A A A A A A A A A A A A A A A A A A A A A A A A A A A A A A A A A A A A A A A A A A A A A A A A A A A . A T A A A A A A A A A A A A A A A A A A A A A A A A A . A A A A A A A A A A A A A A A A A A A A A A A A A A A A A A A A A A A A A A A A . A A A A A A A A . . A A A A A A A A A A A A A A A A A A A A A A    
841	. . . . . . . . . . . . . . . . . . . . . . A A A A A A A A A A A A A A A A A A A A A A A A A A A A A A A A A A A A A A A A A A A A A A A A A A A A A A A A A A A A A . A A A A A A A A A A A A A A A A A A A A A A A A A A A . A A A A A A A A A A A A A A A A A A A A A A A A A A A A A A A A A A A A A A A A . A A A A A A A A . . A A A A A A A A A A A A A A A A A A A A A A    
842	. . . . . . . . . . . . . . . . . . . . . . A A A A A A A A A A A A A A A A A A A A A A A A A A A A A A A A A A A A A A A A A A A A A A A A A A A A A A A A A A A A A . A A A A A A A A A A A A A A A A A A A A A A A A A A A . A A A A A A A A A A A A A A A A A A A A A A A A A A A A A A A A A A A A A A A A . A A A A A A A A . . A A A A A A A A A A A A A A A A A A A A A A    
843	. . . . . . . . . . . . . . . . . . . . . . A A A A A A A A A A A A A A A A A A A A A A A A A A A A A A A A A A A A A A A A A A A A A A A A A A A A A A A A A A A A A . A A A A A A A A A A A A A A A A A A A A A A A A A A A . A A A A A A A A A A A A A A A A A A A A A A A A A A A A A A A A A A A A A A A A . A A A A A A A A . . A A A A A A A A A A A A A A A A A A A A A A    
844	. . . . . . . . . . . . . . . . . . . . . . A A A A A A A A A A A A A A A A A A A A A A A A A A A A A A A A A A A A A A A A A A A A A A A A A A A A A A A A A A A A A . A A A A A A A A A A A A A A A A A A A A A A A A A A A . A A A A A A A A A A A A A A A A A A A A A A A A A A A A A A A A A A A A A A A A . A A A A A A A A . . A A A A A A A A A A A A A A A A A A A A A A    
845	. . . . . . . . . . . . . . . . . . . . . . C C C C C C C C C C C C C C C C C C C C C C C C C C C C C C C C C C C C C C C C C C C C C C C C C C C C C C C C C C C C C . C C C C C C C C C C C C C C C C C C C C C C C C C C C . C C C C C C C C C C C C C C C C C C C C C C C C C C C C C C C C C C C C C C C C . C C C C C C C C . . C C C C C C C C C C C C C C C C C C C C C C    
846	. . . . . . . . . . . . . . . . . . . . . . C C C C C C C C C C C C C C C C C C C C C C C C C C C C C C C C C C C C C C C C C C C C C C C C C C C C C C C C C C C C C . C C C C C C C C C C C C C C C C C C C C C C C C C C C . C C C C C C C C C C C C C C C C C C C C C C C C C C C C C C C C C C C C C C C C . C C C C C C C C . . C C C C C C C C C C C C C C C C C C C C C C    
847	. . . . . . . . . . . . . . . . . . . . . . C C C C C C C C C C C C C C C C C C C C C C C C C C C C C C C C C C C C C C C C C C C C C C C C C C C C C C C C C C C C C . C C C C C C C C C C C C C C C C C C C C C C C C C C C . C C C C C C C C C C C C C C C C C C C C C C C C C C C C C C C C C C C C C C C C . C C C C C C C C . . C C C C C C C C C C C C C C C C C C C C C C    
848	. . . . . . . . . . . . . . . . . . . . . . G G G G G G G G G G G G G G G G G G G G G G G G G G G G G G G G G G G G G G G G G G G G G G G G G G G G G G G G G G G G N . G G G G G G G G G G G G G G G G G G G G G G G G G G G . G G G G G G G G G G G G G G G G G G G G G G G G G G G G G G G G G G G G G G G G . G G G G G G G G . . G G G G G G G G G G G G G G G G G G G G G G    
849	. . . . . . . . . . . . . . . . . . . . . . T T T T T T T T T T T T T T T T T T T T T T T T T T T T T T T T T T T T T T T T T T T T T T T T T T T T T T T T T T T T T . T T T T T T T T T T T T T T T T T T T T T T T T T T T . T T T T T T T T T T T T T T T T T T T T T T T T T T T T T T T T T T T T T T T T . T T T T T T T T . . T T T T T T T T T T T T T T T T T T T T T T    
850	. . . . . . . . . . . . . . . . . . . . . . - - - - - - - - - - - - - - - - - - - - - - - C - - - - - - - - - - - - - - - - - - - - - - - - - - - - - - - - - - - - - . - - - - - - - - - - - - - - - - - - - - - - - - - - - . - - - - - - - - - - - - - - - - - - - - - - - - - - - - - - - - - - - - - - - - . - - - - - - - - . . - - - - - - - - - - - - - - - - - - - - - -    
851	. . . . . . . . . . . . . . . . . . . . . . G G G G G G G G G G G G G G G G G G G G G G G G G G G G G G G G G G G G G G G G G G G G G G G G G G G G G G G G G G G G G . G G G G G G G G G G G G G G G G G G G G G G G G G G G . G G G G G G G G G G G G G G G G G G G G G G G G G G G G G G G G G G G G G G G G . G G G G G G G G . . G G G G G G G G G G G G G G G G G G G G G G    
852	. . . . . . . . . . . . . . . . . . . . . . A A A A A A A A A A A A A A A A A A A A A A A A A A A A A A A A A A A A A A A A A A A A A A A A A A A A A A A A A A A A A . A A A A A A A A A A A A A A A A A A A A A A A A A A A . A A A A A A A A A A A A A A A A A A A A A A A A A A A A A A A A A A A A A A A A . A A A A A A A A . . A A A A A A G A A A A A A A A A A A A A A A    
853	. . . . . . . . . . . . . . . . . . . . . . G G G G G G G G G G G G G G G G G G G G G G G G G G G G G G G G G G G G G G G G G G G G G G G G G G G G G G G G G G G G G . G G G G G G G G G G G G G G G G G G G G G G G G G G G . G G G G G G G G G G G G G G G G G G G G G G G G G G G G G G G G G G G G G G G G . G G G G G G G G . . G G G G G G G G G G G G G G G G G G G G G G    
854	. . . . . . . . . . . . . . . . . . . . . . C C C C C C C C . C C C C C C C C C C C C C C C C C C C C C C C C C C C C C C C C C C C C C C C C C C C C C C C C C C C C . C C C C C C C C C C C C C C C C C C C C C C C C C C C . C C C C C C C C C C C C C C C C C C C C C C C C C C C C C C C C C C C C C C C C . C C C C C C C C . . C C C C C C C C C C C C C C C C C C C C C C    
855	. . . . . . . . . . . . . . . . . . . . . . T T T T T T T T . T T T T T T T T T T T T T T T T T T T T T T T T T T T T T T T T T T T T T T T T T T T T T T T T T T T T . T T T T T T T T T T T T T T T T T T T T T T T T T T T . T T T T T T T T T T T T T T T T T T T T T T T T T T T T T T T T T T T T T T T T . T T T T T T T T . . T T T T T T T T T T T T T T T T T T T T T T    
856	. . . . . . . . . . . . . . . . . . . . . . G G G G G G G G . G G . G G G G G G G G G G G G G G G G G G G G G G G G G G G G G G G G G G G G G G G G G G G G G G G G G . G G G G G G G G G G G G G G G G G G G G G G G G G G G . G G G G G G G G G G G G G G G G G G G G G G G G G G G G G G G G G G G G G G G G . G G G G G G G G . . G G G G G G G G G G G G G G G G G G G G G G    
857	. . . . . . . . . . . . . . . . . . . . . . T T T T T T T T . T T . T T T T T T T T T T T T T T T T T T T T T T T T T T T T T T T T T T T T T T T T T T T T T T T T T . T T T T T T T T T T T T T T T T T T T T T T T T T T T . T T T T T T T T T T T T T T T T T T T T T T T T T T T T T T T T T T T T T T T T . T T T T T T T T . . T T T T T T T T T T T T T T T T T T T T T T    
858	. . . . . . . . . . . . . . . . . . . . . . G G G G G G G G . G G . G G G G G G G G G G G G G G G G G G G G G G G G G G G G G G G G G G G G G G G G G G G G G G G G G . G G G G G G G G G G G G G G G G G G G G G G G G G G G . G G G G G G G G G G G G G G G G G G G G G G G G G G G G G G G G G G G G G G G G . G G G G G G G G . . G G G G G G G G G G G G G G G G G G G G G G    
859	. . . . . . . . . . . . . . . . . . . . . . A A A A A A A A . A A . A A A A A A A A A A A A A A A A A A A A A A A A A A A A A A A A A A A A A A A A A A A A A A A A A . A A A A A A A A A A A A A A A A A A A A A A A A A A A . A A A A A A A A A A A A A A A A A A A A A A A A A A A A A A A A A A A A A A A A . A A A A A A A A . . A A A A A A A A A A A A A A A A A A A A A A    
860	. . . . . . . . . . . . . . . . . . . . . . G G G G G G G T . G G . G G G G G G G G G G G G G G G G G G G G G G G G G G G G G G G G G G G G G G G G G G G G G G G G G . G G G G G G G G G G G G G G G G G G G G G G G G G G G . G G G G G G G G G G G G G G G G G G G G G G G G G G G G G G G G G G G G G G G G . G G G G G G G G . G G G G G G G G G G G G G G G G G G G G G G G    
861	. . . . . . . . . . . . . . . . . . . . . . G G G G G G G . . G G . G G G G G G G G G G G G G G G G G G G G G G G G G G G G G G G G G G G G G G G G G G G G G G G G G . G G G G G G G G G G G G G G G G G G G G G G G G G G G . G G G G G G G G G G G G G G G G G G G G G G G G G G G G G G G G G G G G G G G G . G G G G G G G G G G G G G G G G G G G G G G G G G G G G G G G G    
862	. . . . . . . . . . . . . . . . . . . . . . A A A A A A A . . A A . A A A A A A A A A A A A A A A A A A A A A A A A A A A A A A A A A A A A A A A A A A A A A A A A A . A A A A A A A A A A A A A A A A A A A A A A A A A A A . A A A A A A A A A A A A A A A A A A A A A A A A A A A A A A A A A A A A A A A A . A A A A A A A A A A A A A A A A A A A A A A A A A A A A A A A A    
863	. . . . . . . . . . . . . . . . . . . . . . C C C C C C C . . C C . A C C C C C C C C C C C C C C C C C C C C C C C C C C C C C C C C C C C C C C C C C C C C C C C C . C C C C C C C C C C C C C C C C C C C C C C C C C C C . C C C C C C C C C C C C C C C C C C C C C C C C C C C C C C C C C C C C C C C C . C C C C C C C C C C C C C C C C C C C C C C C C C C C C C C C C    
864	. . . . . . . . . . . . . . . . . . . . . . C C C C C C C . . C C . . C C C C C C C C C C C C C C C C C C C C C C C C C C C C C C C C C C C C C C C C C C C C C C C C . C C C C C C C C C C C C C C C C C C C C C C C C C C C . C C C C C C C C C C C C C C C C C C C C C C C C C C C C C C C C C C C C C C C C . C C C C C C C C C C C C C C C C C C C C C C C C C C C C C C C C    
865	. . . . . . . . . . . . . . . . . . . . . . C A A A A A A . . A A . . A A A A A A A A A A A A A A A A A A A A A A A A A A A A A A A A A A A A A A A A A A A A A A A A . A A A A A A A A A A A A A A A A A A A A A A A A A A A . A A A A A A A A A A A A A A A A A A A A A A A A A A A A A A A A A A A A A A A A . A A A A A A A A A A A A A A A A A A A A A A A A A A A A A A A A    
866	. . . . . . . . . . . . . . . . . . . . . . . G G G G G G . . G G . . G G G G G G G G G G G G G G G G G G G G G G G G G G G G G G G G G G G G G G G G G G G G G G G G . G G G G G G G G G G G G G G G G G G G G G G G G G G G . G G G G G G G G G G G G G G G G G G G G G G G G G G G G G G G G G G G G G G G G . G G G G G G G G G G G G G G G G G G G G G G G G G G G G G G G G    
867	. . . . . . . . . . . . . . . . . . . . . . . T T T T T T . . T T . . T T T T T T T T T T T T T T T T T T T T T T T T T T T T T T T T T T T T T T T T T T T T T T T T . T T T T T T T T T T T T T T T T T T T T T T T T T T T . T T T T T T T T T T T T T T T T T T T T T T T T T T T T T T T T T T T T T T T T . T T T T T T T T T T T T T T T T T T T T T T T T T T T T T T T T    
868	. . . . . . . . . . . . . . . . . . . . . . . A A A A A A . . A A . . A A A A A A A A A A A A A A A A A A A A A A A A A A A A A A A A A A A A A A A A A A A A A A A A . A A A A A A A A A A A A A A A A A A A A A A A A A A A . A A A A A A A A A A A A A A A A A A A A A A A A A A A A A A A A A A A A A A A A . A A A A A A A A A A A A A A A A A A A A A A A A A A A A A A A A    
869	. . . . . . . . . . . . . . . . . . . . . . . . A A A A A . . A A . . A A A A A A A A A A A A A A A A A A A A A A A A A A A A A A A A A A A A A A A A A A A A A A A A . A A A C A A A A A A A A A A A A A A A A A A A A A A A . A A A A A A A A A A A A A A A A A A A A A A A A A A A A A A A A A A A A A A A A . A A A A A A A A A A A A A A A A A A A A A A A A A A A A A A A A    
870	. . . . . . . . . . . . . . . . . . . . . . . . A A A A A . . A A . . A A A A A A A A A A A A A A A A A A A A A A A A A A A A A A A A A A A A A A A A A A A A A A A A . A A A A A A A A A A A A A A A A A A A A A A A A A A A . A A A A A A A A A A A A A A A A A A A A A A A A A A A A A A A A A A A A A A A A . A A A A A A A A A A A A A A A A A A A A A A A A A A A A A A A A    
871	. . . . . . . . . . . . . . . . . . . . . . . . A A A A A . . A A . . A A A A A A A A A A A A A A A A A A A A A A A A A A A A A A A A A A A A A A A A A A A A A A A A . A A A A A A A A A A A A A A A A A A A A A A A A A A A . A A A A A A A A A A A A A A A A A A A A A A A A A A A A A A A A A A A A A A A A . A A A A A A A A A A A A A A A A A A A A A A A A A A A A A A A A    
872	. . . . . . . . . . . . . . . . . . . . . . . . T C C T T . . C C . . C C C C C C C T T T T T T T C C C C C C C C C T C C C T T C C C C C C C T T T C C C C C T C C C . C C C C C C T C C C T C C C C T C C T T T T T T T T T . C C C C C C C C C C C C C C C C C C C C C C C C C C C T T C C T T T T T T T T T . C C C C C T T C C C T T T T C C C C C C C C C T T C C C C C T C  49	 transition  
873	. . . . . . . . . . . . . . . . . . . . . . . . T T T T T . . T T . . T T T T T T T T T T T T T T T T T T T T T T T T T T T T T T T T T T T T T T T T T T T T T T T T . T T T T T T T T T T T T T T T T T T T T T T T T T T T . T T T T T T T T T T T T T T T T T T T T T T T T T T T T T T T T T T T T T T T T . T T T T T T T T T T T T T T T T T T T T T T T T T T T T T T T T    
874	. . . . . . . . . . . . . . . . . . . . . . . . G G G G G . . G G . . G G G G G G G G G G G G G G G G G G G G G G G G G G G G G G G G G G G G G G G G G G G G G G G G . G G G G G G G G G G G G G G G G G G G G G G G G G G G . G G G G G G G G G G G G G G G G G G G G G G G G G G G G G G G G G G G G G G G G . G G G G G G G G G G G G G G G G G G G G G G G G G G G G G G G G    
875	. . . . . . . . . . . . . . . . . . . . . . . . T T T T T . . T T . . T T T T T T T T T T T T T T T T T T T T T T T T T T T T T T T T T T T T T T T T T T T T T T T T . T T T T T T T T T T T T T T T T T T T T T T T T T T T . T T T T T T T T T T T T T T T T T T T T T T T T T T T T T T T T T T T T T T T T . T T T T T T T T T T T T T T T T T T T T T T T T T T T T T T T T    
876	. . . . . . . . . . . . . . . . . . . . . . . . C C C C C . . C C . . C C C C C C C C C C C C C C C C C C C C C C C C C C C C C C C C C C C C C C C C C C C C C C C C . C C C C C C C C C C C C C C C C C C C C C C C C C C C . C C C C C C C C C C C C C C C C C C C C C C C C C C C C C C C C C C C C C C C C . C C C C C C C C C C C C C C C C C C C C C C C C C C C C C C C C    
877	. . . . . . . . . . . . . . . . . . . . . . . . C C C C C . . C C . . C C C C C C C C C C C C C C C C C C C C C C C C C C C C C C C C C C C C C C C C C C C C C C C C . C C C C C C C C C C C C C C C C C C C C C C C C C C C . C C C C C C C C C C C C C C C C C C C C C C C C C C C C C C C C C C C C C C C C . C C C C C C C C C C C C C C C C C C C C C C C C C C C C C C C C    
878	. . . . . . . . . . . . . . . . . . . . . . . . C C C C C . . C C . . C C C C C C C C C C C C C C C C C C C C C C C C C C C C C C C C C C C C C C C C C C C C C C C C . C C C C C C C C C C C C C C C C C C C C C C C C C C C . C C C C C C C C C C C C C C C C C C C C C C C C C C C C C C C C C C C C C C C C . C C C C C C C C C C C C C C C C C C C C C C C C C C C C C C C C    
879	. . . . . . . . . . . . . . . . . . . . . . . . A A A A A . . . A . . C A A A A A A A A A A A A A A A A A A A A A A A A A A A A A A A A A A A A A A A A A A A A A A A . A A A A A A A A A A A A A A A A A A A A A A A A A A A . A A A A A A A A A A A A A A A A A A A A A A A A A A A A A A A A C A A A A A A A . A A A A A A A A A A A A A A A A A A A A A A A A A A A A A A A A    
880	. . . . . . . . . . . . . . . . . . . . . . . . C C C C C . . . C . . . C C C C C C C C C C C C C C C C C C C C C C C C C C C C C C C C C C C C C C C C C C C C C C C . C C C C C C C C C C C C C C C C C C C C C C C C C C C . C C C C C C C C C C C C C C C C C C C C C C C C C C C C C C C C C C C C C C C C . C C C C C C C C C C C C C C C C C C C C C C C C C C C C C C C C    
881	. . . . . . . . . . . . . . . . . . . . . . . . T T T T T . . . T . . . T T T T T T T T T T T T T T T T T T T T T T T T T T T T T T T T T T T T T T T T T T T T T T T . T T T T T T T T T T T T T T T T T T T T T T T T T T T . T T T T T T T T T T T T T T T T T T T T T T T T T T T T T T T T T T T T T T T T . T T T T T T T T T T T T T T T T T T T T T T T T T T T T T T T T    
882	. . . . . . . . . . . . . . . . . . . . . . . . A A A A A . . . A . . . A A A A A A A A A A A A A A A A A A A A A A A A A A A A A A A A G A A A A A A A A A A A A A A . A A A A A A A A A A A A A A A A A A A A A A A A A A A . A A A A A A A A A A G A A A A A A A A A A A A A A A A A A A A A A A A A A A A A . A A A A A A A A A A A A A A A A A A A A A A A A A A A A A A A A    
883	. . . . . . . . . . . . . . . . . . . . . . . . G G G G G . . . G . . . G G G G G G G G G G G G G G G G G G G G G G G G G G G G G G G G G G G G G G G G G G G G G G G . G G G G G G G G G G G G G G G G G G G G G G G G G G G . G G G G G G G G G G G G G G G G G G G G G G G G G G G G G G G G G G G G G G G G . G G G G G G G G G G G G G G G G G G G G G G G G G G G G G G G G    
884	. . . . . . . . . . . . . . . . . . . . . . . . A A A A A . . . A . . . A A A A A A A A A A A A A A A A A A A A A A A A A A A A A A A A A A A A A A A A A A A A A A A . A A A A A A A A A A A A A A A A A A A A A A A A A A A . A A A A A A A A A A A A A A A A A A A A A A A A A A A A A A A A A A A A A A A A . A A A A A A A A A A A A A A A A A A A A A A A A A A A A A A A A    
885	. . . . . . . . . . . . . . . . . . . . . . . . C C C C C . . . C . . . C C C C C C C C C C C C C C C C C C C C C C C C C C C C C C C C C C C C C C C C C C C C C C C . C C C C C C C C C C C C C C C C C C C C C C C C C C C . C C C C C C C C C C C C C C C C C C C C C C C C C C C C C C C C C C C C C C C C . C C C C C C C C C C C C C C C C C C C C C C C C C C C C C C C C    
886	. . . . . . . . . . . . . . . . . . . . . . . . A A A A A . . . A . . . A A A A A A A A A A A A A A A A A A A A A A A A A A A A A A A A A A A A A A A A A A A A A A A . A A A A A A A A A A A A A A A A A A A A A A A A A A A . A A A A A A A A A A A A A A A A A A A A A A A A A A A A A A A A A A A A A A A A . A A A A A A A A A A A A A A A A A A A A A A A A A A A A A A A A    
887	. . . . . . . . . . . . . . . . . . . . . . . . T T T T T . . . T . . . T T T T T T T T T T T T T T T T T T T T T T T T T T T T T T T T T T T T T T T T T T T T T T T . T T T T T T T T T T T T T T T T T T T T T T T T T T T . T T T T T T T T T T T T T T T T T T T T T T T T T T T T T T T T T T T T T T T T . T T T T T T T T T T T T T T T T T T T T T T T T T T T T T T T T    
888	. . . . . . . . . . . . . . . . . . . . . . . . C C C C C . . . C . . . C C C C C C C C C C C C C C C C C C C C C C C C C C C C C C C C C C C C C C C C C C C C C C C . C C C C C C C C C C C C C C C C C C C C C C C C C C C . C C C C C C C C C C C C C C C C C C C C C C C C C C C C C C C C C C C C C C C C . C C C C C C C C C C C C C C C C C C C C C C C C C C C C C C C C    
889	. . . . . . . . . . . . . . . . . . . . . . . . A A A A A . . . A . . . A A A A A A A A A A A A A A A A A A A A A A A A A A A A A A A A A A A A A A A A A A A A A A A . A A A A A A A A A A A A A A A A A A A A A A A A A A A . A A A A A A A A A A A A A A A A A A A A A A A A A A A A A A A A A A A A A A A A . A A A A A A A A A A A A A A A A A A A A A A A A A A A A A A A A    
890	. . . . . . . . . . . . . . . . . . . . . . . . T T T T T . . . T . . . T T T T T T T T T T T T T T T T T T T T T T T T T T T T T T T T T T T T T T T T T T T T T T T . T T T T T T T T T T T T T T T T T T T T T T T T T T T . T T T T T T T T T T T T T T T T T T T T T T T T T T T T T T T T T T T T T T T T . T T T T T T T T T T T T T T T T T T T T T T T T T T T T T T T T    
891	. . . . . . . . . . . . . . . . . . . . . . . . A A A A A . . . A . . . A A A A A A A A A A A A A A A A A A A A A A A A A A A A A A A A A A A A A A A A A A A A A A A . A A A A A A A A A A A A A A A A A A A A A A A A A A A . A A A A A A A A A A A A A A A A A A A A A A A A A A A A A A A A A A A A A A A A . A A A A A A A A A A A A A A A A A A A A A A A A A A A A A A A A    
892	. . . . . . . . . . . . . . . . . . . . . . . . T T T T T . . . T . . . T T T T T T T T T T T T T T T T T T T T T T T T T T T T T T T T T T T T T T T T T T T T T T T . T T T T T T T T T T T T T T T T T T T T T T T T T T T . T T T T T T T T T T T T T T T T T T T T T T T T T T T T T T T T T T T T T T T T . T T T T T T T T T T T T T T T T T T T T T T T T T T T T T T T T    
893	. . . . . . . . . . . . . . . . . . . . . . . . C C C C C . . . C . . . C C C C C C C C C C C C C C C C C C C C C C C C C C C C C C C C C C C C C C C C C C C C C C C . C C C C C C C C C C C C C C C C C C C C C C C C C C C . C C T C C C C C C C C C C C C C C C C C C C C C C C C C C C C C C C C C C C C C . C C C C C C C C C C C C C C C C C C C C C C C C C C C C C C C C    
894	. . . . . . . . . . . . . . . . . . . . . . . . T T T T T . . . T . . . T T T T T T T T T T T T T T T T T T T T T T T T T T T T T T T T T T T T T T T T T T T T T T T . T T T T T T T T T T T T T T T T T T T T T T T T T T T . T T T T T T T T T T T T T T T T T T T T T T T T T T T T T T T T T T T T T T T T . T T T T T T T T T T T T T T T T T T T T T T T T T T T T T T T T    
895	. . . . . . . . . . . . . . . . . . . . . . . . T A A A A . . . A . . . A A A A A A A A A A A A A A A A A A A A A A A A A A A A A A A A A A A A A A A A A A A A A A A . A A A A A A A A A A A A A A A A A A A A A A A A A A A . A A A A A A A A A A A A A A A A A A A A A A A A A A A A A A A A A A A A A A A A . A A A A A A A A A A A A A A A A A A A A A A A A A A A A A A A A    
896	. . . . . . . . . . . . . . . . . . . . . . . . . . . C C . . . C . . . C C C C C C C C C C C C C C C C C C C C C C C C C C C C C C C C C C C C C C C C C C C C C C C . C C C C C C C C C C C C C C C C C C C C C C C C C C C . C C C C C C C C C C C C C C C C C C C C C C C C C C C C C C C C C C C C C C C C . C C C C C C C C C C C C C C C C C C C C C C C C C C C C C C C C    
897	. . . . . . . . . . . . . . . . . . . . . . . . . . . A . . . . A . . . A A A A A A A A A A A A A A A A A A A A A A A A A A A A A A A A A A A A A A A A A A A A A A A . N A A A A A A A A A A A A A A A A A A A A A A A A A A . A A A A A A A A A A A A A A A A A A A A A A A A A A A A A A A A A A A A A A A A . A A A A A A A A A A A A A A A A A A A A A A A A A A A A A A A A    
898	. . . . . . . . . . . . . . . . . . . . . . . . . . . . . . . . . . . . T T T T T T T T T T T T T T T T T T T T T T T T T T T T T T T T T T T T T T T T T T T T T T T . T T T T T T T T T T T T T T T T T T T T T T T T T T T . T T T T T T T T T T T T T T T T T T T T T T T T T T T T T T T T T T T T T T T T . T T T T T T T T T T T T T T T T T T T T T T T T T T T T T T T T    
899	. . . . . . . . . . . . . . . . . . . . . . . . . . . . . . . . . . . . T T T T T T T T T T T T T T T T T T T T T T T T T T T T T T T T T T T T T T T T T T T T T T T . T T T T T T T T T T T T T T T T T T T T T T T T T T T . T T T T T T T T T T T T T T T T T T T T T T T T T T T T T T T T T T T T T T T T . T T T T T T T T T T T T T T T T T T T T T T T T T T T T T T T T    
900	. . . . . . . . . . . . . . . . . . . . . . . . . . . . . . . . . . . . T T T T T T T T T T T T T T T T T T T T T T T T T T T T T T T T T T T T T T T T T T T T T T T . T T T T T T T T T T T T T T T T T T T T T T T T T T T . T T T T T T T T T T T T T T T T T T T T T T T T T T T T T T T T T T T T T T T T . T T T T T T T T T T T T T T T T T T T T T T T T T T T T T T T T    
901	. . . . . . . . . . . . . . . . . . . . . . . . . . . . . . . . . . . . T A A A A A A A A A A A A A A A A A A A A A A A A A A A A A A A A A A A A A A A A A A A A A A . A A A A A A A A A A A A A A A A A A A A A A A A A A A . A A A A A A A A A A A A A A A A A A A A A A A A A A A A A A A A A A A A A A A A . A A A A A A A A A A A A A A A A A A A A A A A A A A A A A A A A    
902	. . . . . . . . . . . . . . . . . . . . . . . . . . . . . . . . . . . . . A A A A A A A A A A A A A A A A A A A A A A A A A A A A A A A A A A A A A A A A A A A A A A . A A A A A A A A A A A A A A A A A A A A A A A A A A A . A A A A A A A A A A A A A A A A A A A A A A A A A A A A A A A A A A A A A A A A . A A A A A A A A A A A A A A A A A A A A A A A A A A A A A A A A    
903	. . . . . . . . . . . . . . . . . . . . . . . . . . . . . . . . . . . . . G G G G G G G G G G G G G G G G G G G G G G G G G G G G G G G G G G G G G G G G G G G G G G . G G G G G G G G G G G G G G G G G G G G G G G G G G G . G G G G G G G G G G G G G G G G G G G G G G G G G G G G G G G G G G G G G G G G . G G G G G G G G G G G G G G G G G G G G G G G G G G G G G G G G    
904	. . . . . . . . . . . . . . . . . . . . . . . . . . . . . . . . . . . . . C C C C C C C C C C C C C C C C C C C C C C C C C C C C C C C C C C C C C C C C C C C C C C . C C C C C C C C C C C C C C C C C C C C C C C C C C C . C C C C C C C C C C C C C C C C C C C C C C C C C C C C C C C C C C C C C C C C . C C C C C C C C C C C C C C C C C C C C C C C C C C C C C C C C    
905	. . . . . . . . . . . . . . . . . . . . . . . . . . . . . . . . . . . . . A A A A A A G . A A A A A A A A A A A A A A A A A A A A A A A A A A A A A A A A A A A A A A . A A A A A A A A A A A A A A A A A A A A A A A A A A A . A A A A A A A A A A A A A A A A A A A A A A A A A A A A A A A A A A A A A A A A . A A A A A A A A A A A A A G A A A A A A A A A A A A A A A A A A    
906	. . . . . . . . . . . . . . . . . . . . . . . . . . . . . . . . . . . . . C C C C C C C . C C C C . C C C C C C C C C C C C C C C C C C C C C C C C C C C C C C C C C . C C C C C C C C C C C C C C C C C C C C C C C C C C C . C C C C C C C C C C C C C C C C C C C C C C C C C C C C C C C C C C C C C C C C . C C C C C C C C C C C C C C C C C C C C C C C C C C C C C C C C    
907	. . . . . . . . . . . . . . . . . . . . . . . . . . . . . . . . . . . . . T T T T T T T . T T T T . T T T T T T T T T T T T T T T T T T T T T T T T T T T T T T T T T . T T T T T T T T T T T T T T T T T T T T T T T T T T T . T T T T T T T T T T T T T T T T T T T T T T T T T T T T T T T T T T T T T T T T . T T T T T T T T T T T T T T T T T T T T T T T T T T T T T T T T    
908	. . . . . . . . . . . . . . . . . . . . . . . . . . . . . . . . . . . . . T T T T T T T . . T T T . T T T T T T T T T T T T T T T T T T T T T T T T T T T T T T T T T . T T T T T T T T T T T T T T T T T T T T T T T T T T T . T T T T T T T T T T T T T T T T T T T T T T T T T T T T T T T T T T T T T T T T . T T T T T T T T T T T T T T T T T T T T T T T T T T T T T T T T    
909	. . . . . . . . . . . . . . . . . . . . . . . . . . . . . . . . . . . . . C C C C C C C . . C C C . C C C C C C C T C C C C C C C T C C C C C C C C C C C C C C C C C . C C C C C C C C C C C C C C C C C C C C C C C C C C C . C C C C C C C C C C C C C C C C C C C C C C C C C C C C C C C C C C C C C C C C . C C C C C C C C C C C C C C C C C C C C C C C C C C C C C C C C    
910	. . . . . . . . . . . . . . . . . . . . . . . . . . . . . . . . . . . . . C C C C C C C . . C C C . C C C C C C C C C C C C C C C C C C C C C C C C C C C C C C C C C . C C C C C C C C C C C C C C C C C C C C C C C C C C C . C C C C C C C C C C C C C C C C C C C C C C C C C C C C C C C C C C C C C C C C . C C C C C C C C C C C C C C C C C C C C C C C C C C C C C C C C    
911	. . . . . . . . . . . . . . . . . . . . . . . . . . . . . . . . . . . . . T T T T T T T . . T T T . T T T T T T T . T T T T T T T T T T T T T T T T T T T T T T T T T . T T T T T T T T T T T T T T T T T T T T T T T T T T T . T T T T T T T T T T T T T T T T T T T T T T T T T T T T T T T T T T T T T T T T . T T T T T T T T T T T T T T T T T T T T T T T T T T T T T T T T    
912	. . . . . . . . . . . . . . . . . . . . . . . . . . . . . . . . . . . . . T T T T T T T . . T T T . T T T T T T T . T T T T T T T T T T T T T T T T T T T T T T T T T . T T T T T T T T T T T T T T T T T T T T T T T T T T T . T T T T T T T T T T T T T T T T T T T T T T T T T T T T T T T T T T T T T T T T . T T T T T T T T T T T T T T T T T T T T T T T T T T T T T T T T    
913	. . . . . . . . . . . . . . . . . . . . . . . . . . . . . . . . . . . . . T T T T T T T . . T T T . T T T T T T T . T T T T T T T T T T T T T T T T T T T T T T T T T . T T T T T T T T T T T T T T T T T T T T T T T T T T T . T T T T T T T T T T T T T T T T T T T T T T T T T T T T T T T T T T T T T T T T . T T T T T T T T T T T T T T T T T T T T T T T T T T T T T T T T    
914	. . . . . . . . . . . . . . . . . . . . . . . . . . . . . . . . . . . . . C C C . . C C . . C C C . . C C T C C C . C C C C C C C C C C C C C C C C C C C C C C C C C . C C C C C C C C C C C C C C C C C C C C C C C C C C C . C C C C C C C C C C C C C C C C C C C C C C C C C C C C C C C C C C C C C C C C . C C C C C C C C C C C C C C C C C C C C C C C C C C C C C C C C    
915	. . . . . . . . . . . . . . . . . . . . . . . . . . . . . . . . . . . . . A A A . . A A . . A A A . . . A . A A G . A A A A A A A A A A A A A A A A A A A A A A A A A . A A A A A A A A A A A A A A A A A A A A A A A A A A A . A A A A A A A A A A A A A A A A A A G A A A A A A A A A A A A A A A A A A A A A . A A A A A A A A A A A A A A A A A A A A A A A A A A A A A A A A    
916	. . . . . . . . . . . . . . . . . . . . . . . . . . . . . . . . . . . . . G G G . . A G . . G G G . . . G . G G G . G G G G G G G G G G G G G G G G G G G G G G G G G . G G G G G G G G G G G G G G G G G G G G G G G G G G G . G G G G G G G G G G G G G G G G G G G G G G G G G G G G G G G G G G G G G G G G . G G G G G G G G G G G G G G G G G G G G G G G G G G G G G G G G    
917	. . . . . . . . . . . . . . . . . . . . . . . . . . . . . . . . . . . . . A A A . . . A . . A A A . . . A . A A A . A A A A A A A A A A A A A A A A A A A A A A A A A . A A A A A A A A A A A A A A A A A A A A A A A A A A A . A A A A A A A A A A A A A A A A A A A A A A A A A A A A A A A A A A A A A A A A . A A A A A A A A A A A A A A A A A A A A A A A A A A A A A A A A    
918	. . . . . . . . . . . . . . . . . . . . . . . . . . . . . . . . . . . . . A A A . . . A . . A A A . . . A . A A A . A A A A A A A A A A A A A A A A A A A A A A A A A . A A A A A A A A A A A A A A A A A A A A A A A A A A A . A A A A A A A A A A A A A A A A A A A A A A A A A A A A A A A A A A A A A A A A . A A A A A A A A A A A A A A A A A A A A A A A A A A A A A A A A    
919	. . . . . . . . . . . . . . . . . . . . . . . . . . . . . . . . . . . . . T T T . . . T . . T T T . . . T . T T T . T T T T T T T T T T T T T T T T T T T T T T T T T . T T T T T T T T T T T T T T T T T T T T T T T T T T T . T T T T T T T T T T T T T T T T T T T T T T T T T T T T T T T T T T T T T T T T . T T T T T T T T T T T T T T T T T T T T T T T T T T T T T T T T    
920	. . . . . . . . . . . . . . . . . . . . . . . . . . . . . . . . . . . . . G G G . . . G . . G G G . . . . . G G G . G G G G G G G G G G G G G G G G G G G G G G G G G . G G G G G G G G G G G G G G G G G G G G G G G G G G G . G G G G G G G G G G G G G G G G G G G G G G G G G G G G G G G G G G G G G G G G . G G G G G G G G G G G G G G G G G G G G G G G G G G G G G G G G    
921	. . . . . . . . . . . . . . . . . . . . . . . . . . . . . . . . . . . . . C C C . . . C . . C C C . . . . . C C C . C C C C C C C C C C C C T C C C C C C C C C C C C . C C C C C C C C C C C C C C C C C C C C C C C C C C C . C C C C C C C C C C C C C C C C C C C C C C C C C C C C C C C C C C C C C C C C . C C C C C C C C C C C C C C C C C C C C C C C C C C C C C C C C    
922	. . . . . . . . . . . . . . . . . . . . . . . . . . . . . . . . . . . . . C C C . . . C . . C C C . . . . . C C C . C C C C C C C C C C C C C C C C C C C C C C C C C . C C C C C C C C C C C C C C C C C C C C C C C C C C C . C C C C C C C C C C C C C C C C C C C C C C C C C C C C C C C C C C C C C C C C . C C C C C C C C C C C C C C C C C C C C C C C C C C C C C C C C    
923	. . . . . . . . . . . . . . . . . . . . . . . . . . . . . . . . . . . . . C C C . . . C . . C C C . . . . . C C C . C C C C C C C C C C C C C C C C C C C C C C C C C . C C C C C C C C C C C C C C C C C C C C C C C C C C C . C C C C C C C C C C C C C C C C C C C C C C C C C C C C C C C C C C C C C C C C . C C C C C C C C C C C C C C C C C C C C C C C C C C C C C C C C    
924	. . . . . . . . . . . . . . . . . . . . . . . . . . . . . . . . . . . . . T T T . . . T . . T T T . . . . . T T T . T T T T T T T T T T T T T T T T T T T T T T T T T . T T T T T T T T T T T T T T T T T T T T T T T T T T T . T T T T T T T T T T T T T T T T T T T T T T T T T T T T T T T T T T T T T T T T . T T T T T T T T T T T T T T T T T T T T T T T T T T T T T T T T    
925	. . . . . . . . . . . . . . . . . . . . . . . . . . . . . . . . . . . . . G G G . . . G . . G G G . . . . . G G G . G G G G G G G G G G G G G G G G G G G G G G G G G . G G G G G G G G G G G G G G G G G G G G G G G G G G G . G G G G G G G G G G G G G G G G G G G G G G G G G G G G G G G G G G G G G G G G . G G G G G G G G G G G G G G G G G G G G G G G G G G G G G G G G    
926	. . . . . . . . . . . . . . . . . . . . . . . . . . . . . . . . . . . . . A A A . . . A . . A A A . . . . . A A A . A A A A A A A A A A A A A A A A A A A A A A A A A . A A A A A A A A A A A A A A A A A A A A A A A A A A A . A A A A A A A A A A A A A A A A A A A A A A A A A A A A A A A A A A A A A A A A . A A A A A A A A A A A A A A A A A A A A A A A A A A A A A A A A    
927	. . . . . . . . . . . . . . . . . . . . . . . . . . . . . . . . . . . . . T T T . . . T . . T T T . . . . . T T T . T T T T T T T T T T T T T T T T T T T T T T T T T . T T T T T T T T T T T T T T T T T T T T T T T T T T T . T T T T T T T T T T T T T T T T T T T T T T T T T T T T T T T T T T T T T T T T . T T T T T T T T T T T T T T T T T T T T T T T T T T T T T T T T    
928	. . . . . . . . . . . . . . . . . . . . . . . . . . . . . . . . . . . . . T T T . . . T . . T T T . . . . . T T T . T T T T T T T T T T T T T T T T T T T T T T T T T . T T T T T T T T T T T T T T T T T T T T T T T T T T T . T T T T T T T T T T T T T T T T T T T T T T T T T T T T T T T T T T T T T T T T . T T T T T T T T T T T T T T T T T T T T T T T T T T T T T T T T    
929	. . . . . . . . . . . . . . . . . . . . . . . . . . . . . . . . . . . . . C C C . . . C . . C C C . . . . . C C C . C C C C C C C C C C C C C C C C C C C C C C C C C . C C C C C C C C C C C C C C C C C C C C C C C C C C C . C C C C C C C C C C C C C C C C C C C C C C C C C C C C C C C C C C C C C C C C . C C C C C C C C C C C C C C C C C C C C C C C C C C C C C C C C    
930	. . . . . . . . . . . . . . . . . . . . . . . . . . . . . . . . . . . . . T T T . . . T . . T T . . . . . . T . T . T T T T T T T T T T T T T T T T T T T T T T T T T . T T T T T T T T T T T T T T T T T T T T T T T T T T T . T T T T T T T T T T T T T T T T T T T T T T T T T T T T T T T T T T T T T T T T . T T T T T T T T T T T T T T T T T T T T T T T T T T T T T T T T    
931	. . . . . . . . . . . . . . . . . . . . . . . . . . . . . . . . . . . . . G G G . . . G . . G G . . . . . . G . G . G G G G G G G G G G G G G G G G G G G G G G G G G . G G G G G G G G G G G G G G G G G G G G G G G G G G G . G G G G G G G G G G G G G G G G G G G G G G G G G G G G G G G G G G G G G G G G . G G G G G G G G G G G G G G G G G G G G G G G G G G G G G G G G    
932	. . . . . . . . . . . . . . . . . . . . . . . . . . . . . . . . . . . . . T T T . . . T . . T T . . . . . . T . T . T T T T T T T T T T T T T T T T T T T T T T T T T . T T T T T T T T T T T T T T T T T T T T T T T T T T T . T T T T T T T T T T T T T T T T T T T T T T T T T T T T T T T T T T T T T T T T . T T T T T T T T T T T T T T T T T T T T T T T T T T T T T T T T    
933	. . . . . . . . . . . . . . . . . . . . . . . . . . . . . . . . . . . . . G G G . . . G . . G G . . . . . . G . G . G G G G G G G G G G G G G G G G G G G G G G G G G . G G G G G G G G G G G G G G G G G G G G G G G G G G G . G G G G G G G G G G G G G G G G G G G G G G G G G G G G G G G G G G G G G G G G . G G G G G G G G G G G G G G G G G G G G G G G G G G G G G G G G    
934	. . . . . . . . . . . . . . . . . . . . . . . . . . . . . . . . . . . . . G G G . . . G . . G G . . . . . . G . G . G G G G G G G G G G G G G G G G G G G G G G G G G . G G G G G G G G G G G G G G G G G G G G G G G G G G G . G G G G G G G G G G G G G G G G G G G G G G G G G G G G G G G G G G G G G G G G . G G G G G G G G G G G G G G G G G G G G G G G G G G G G G G G G    
935	. . . . . . . . . . . . . . . . . . . . . . . . . . . . . . . . . . . . . A A A . . . A . . A A . . . . . . A . A . A A A A A A A A A A A A A A A A A A A A A A A A A . A A A A A A A A A A A A A A A A A A A A A A A A A A A . A A A A A A A A A A A A A A A A A A A A A A A A A A A A A A A A A A A A A A A A . A A A A A A A A A A A A A A A A A A A A A A A A A A A A A A A A    
936	. . . . . . . . . . . . . . . . . . . . . . . . . . . . . . . . . . . . . C C C . . . C . . C C . . . . . . C . C . C C C C C C C C C C C C C C C C C C C C C C C C C C C C C C C C C C C C C C C C C C C C C C C C C C C C C . C C C C C C C C C C C C C C C C C C C C C C C C C C C C C C C C C C C C C C C C . C C C C C C C C C C C C C C C C C C C C C C C C C C C C C C C C    
937	. . . . . . . . . . . . . . . . . . . . . . . . . . . . . . . . . . . . . A A A . . . A . . A A . . . . . . A . A . A A A A A A A A A A A A A A A A A A A A A A A A A A A A A A A A A A A A A A A A A A A A A A A A A A A A A . A A A A A A A A A A A A A A A A A A A A A A A A A A A A A A A A A A A A A A A A . A A A A A A A A A A A A A A A A A A A A A A A A A A A A A A A A    
938	. . . . . . . . . . . . . . . . . . . . . . . . . . . . . . . . . . . . . T T T . . . T . . T T . . . . . . T . T . T T T T T T T T T T T T T T T T T T T T T T T T T T T T T T T T T T T T T T T T T T T T T T T T T T T T T . T T T T T T T T T T T T T T T T T T T T T T T T T T T T T T T T T T T T T T T T . T T T T T T T T T T T T T T T T T T T T T T T T T T T T T T T T    
939	. . . . . . . . . . . . . . . . . . . . . . . . . . . . . . . . . . . . . T T T . . . T . . T T . . . . . . T . T . T T T T T T T T T T T T T T T T T T T T T T T T T T T T T T T T T T T T T T T T T T T T T T T T T T T T T . T T T T T T T T T T T T T T T T T T T T T T T T T T T T T T T T T T T T T T T T . T T T T T T T T T T T T T T T T T T T T T T T T T T T T T T T T    
940	. . . . . . . . . . . . . . . . . . . . . . . . . . . . . . . . . . . . . T C C . . . C . . C C . . . . . . C . C . C C C C C C C C C C C C C C C C C C C C C C C C C C C C C C C C C C C C C C C C C C C C C C C C C C C C C . C C T C C C C C C C C C C C C C C T C C C C C C C C C C C C C C C C C C C C C C . C C C C C C C C C C C C C C C C C C C C C C C C C C C C C C C C    
941	. . . . . . . . . . . . . . . . . . . . . . . . . . . . . . . . . . . . . C T T . . . T . . T T . . . . . . T . T . T T T T T T T T T T T T T T T T T T T T T T T T T T T T T T T T T T T T T T T T T T T T T T T T T T T T T . T T T T T T T T T T T T T T T T T T T T T T T T T T T T T T T T T T T T T T T T . T T T T T T T T T T T T T T T T T T T T T T T T T T T T T T T T    
942	. . . . . . . . . . . . . . . . . . . . . . . . . . . . . . . . . . . . . . . A . . . A . . A . . . . . . . A . A . A A A A A A A A A A A A A A A A A A A A A A A A A A A A A A A A A A A A A A A A A A A A A A A A A A A A A . A A A A A A A A A A A A A A A A A A A A A A A A A A A A A A A A A A A A A A A A . A A A A A A A A A A A A A A A A A A A A A A A A A A A A A A A A    
943	. . . . . . . . . . . . . . . . . . . . . . . . . . . . . . . . . . . . . . . G . . . G . . G . . . . . . . G . G . G G G G G G G G G G G G G G G G G G G G G G G G G G G G G G G G G G G G G G G G G G G G G G G G G G G G G . G G G G G G G G G G G G G G G G G G G G G G G G G G G G G G G G G G G G G G G G . G G G G G G G G G G G G G G G G G G G G G G G G G G G G G G G G    
944	. . . . . . . . . . . . . . . . . . . . . . . . . . . . . . . . . . . . . . . A . . . A . . A . . . . . . . A . A . A A A A A A A A A A A A A A A A A A A A A A A A A A A A A A A A A A A A A A A A A A A A A A A A A A A A A . A A A A A A A A A A A A A A A A A A A A A A A A A A A A A A A A A A A A A A A A . A A A A A A A A A A A A A A A A A A A A A A A A A A A A A A A A    
945	. . . . . . . . . . . . . . . . . . . . . . . . . . . . . . . . . . . . . . . T . . . T . . T . . . . . . . T . T . T T T T T T T T T T T T T T T T T T T T T T T T T T T T T T T T T T T T T T T T T T T T T T T T T T T T T . T T T T T T T T T T T T T T T T T T T T T T T T T T T T T T T T T T T T T T T T . T T T T T T T T T T T T T T T T T T T T T T T T T T T T T T T T    
946	. . . . . . . . . . . . . . . . . . . . . . . . . . . . . . . . . . . . . . . A . . . T . . . . . . . . . . A . A . A A A A A A A A A A A A A A A A A A A A A A A A A A A A A A A A A A A A A A A A A A A A A A A A A A A A A . A A A A A A A A A A A A A A A A A A A A A A A A A A A A A A A A A A A A A A A A . A A A A A A A A A A A A A A A A A A A A A A A A A A A A A A A A    
947	. . . . . . . . . . . . . . . . . . . . . . . . . . . . . . . . . . . . . . . A . . . . . . . . . . . . . . A . A . A A A A A A A A A A A A A A A A A A A A A A A A A A A A A A A A A A A A A A A A A A A A A A A A A A A A A . A A A A A A A A A A A A A A A A A A A A A A A A A A A A A A A A A A A A A A A A . A A A A A A A A A A A A A A A A A A A A A A A A A A A A A A A A    
948	. . . . . . . . . . . . . . . . . . . . . . . . . . . . . . . . . . . . . . . T . . . . . . . . . . . . . . T . T . T T T T T T T T T T T T T T T T T T T T T T T T T T T T T T T T T T T T T T T T T T T T T T T T T T T T T . T T T T T T T T T T T T T T T T T T T T T T T T T T T T T T T T T T T T T T T T . T T T T T T T T T T T T T T T T T T T T T T T T T T T T T T T T    
949	. . . . . . . . . . . . . . . . . . . . . . . . . . . . . . . . . . . . . . . C . . . . . . . . . . . . . . C . C . C C C C C C C C C C C C C C C C C C C C C C C C C C C C C C C C C C C C C C C C C C C C C C C C C C C C C . C C C C C C C C C C C C C C C C C C C C C C C C C C C C C C C C C C C C C C C C . C C C C C C C C C C C C C C C C C C C C C C C C C C C C C C C C    
950	. . . . . . . . . . . . . . . . . . . . . . . . . . . . . . . . . . . . . . . C . . . . . . . . . . . . . . C . C . C C C C C C C C C C C C C C C C C C C C C C C C C C C C C C C C C C C C C C C C C C C C C C C C C C C C C . C C C C C C C C C C C C C C C C C C C C C C C C C C C C C C C C C C C C C C C C . C C C C C C C C C C C C C C C C C C C C C C C C C C C C C C C C    
951	. . . . . . . . . . . . . . . . . . . . . . . . . . . . . . . . . . . . . . . A . . . . . . . . . . . . . . . . A . A A A A A A A A A A A A A A A A A A A A A A A A A A A A A A A A A A A A A A A A A A A A A A A A A A A A A . A A A A A A A A A A A A A A A A A A A A A A A A A A A A A A A A A A A A A A A A . A A A A A A A A A A A A A A A A A A A A A A A A A A A A A A A A    
952	. . . . . . . . . . . . . . . . . . . . . . . . . . . . . . . . . . . . . . . G . . . . . . . . . . . . . . . . G . G G G G G G G G G G G G G G G G G G G G G G G G G G G G G G G G G G G G G G G G G G G G G G G G G G G G G . G G G G G G G G G G G G G G G G G G G G G G G G G G G G G G G G G G G G G G G G . G G G G G G G G G G G G G G G G G G G G G G G G G G G G G G G G    
953	. . . . . . . . . . . . . . . . . . . . . . . . . . . . . . . . . . . . . . . C . . . . . . . . . . . . . . . . C . C C C C C C C C C C C C C C C C C C C C C C C C C C C C C C C C C C C C C C C C C C C C C C C C C C C C C . C C C C C C C C C C C C C C C C C C C C C C C C C C C C C C C C C C C C C C C C . C C C C C C C C C C C C C C C C C C C C C C C C C C C C C C C C    
954	. . . . . . . . . . . . . . . . . . . . . . . . . . . . . . . . . . . . . . . A . . . . . . . . . . . . . . . . A . A A A A A A A A A A A A A A A A A A A A A A A A A A A A A A A A A A A A A A A A A A A A A A A A A A A A A . A A A A A A A A A A A A A A A A A A A A A A A A A A A A A A A A A A A A A A A A . A A A A A A A A A A A A A A A A A A A A A A A A A A A A A A A A    
955	. . . . . . . . . . . . . . . . . . . . . . . . . . . . . . . . . . . . . . . C . . . . . . . . . . . . . . . . C . C C C C C C C C C C C C C C C C C C C C C C C C C C C C C C C C C C C C C C C C C C C C C C C C C C C C C . C C C C C C C C C C C C C C C C C C C C C C C C C C C C C C C C C C C C C C C C . C C C C C C C C C C C C C C C C C C C C C C C C C C C C C C C C    
956	. . . . . . . . . . . . . . . . . . . . . . . . . . . . . . . . . . . . . . . G . . . . . . . . . . . . . . . . G . G G G G G G G G G G G G G G G G G G G G G G G G G G G G G G G G G G G G G G G G G G G G G G G G G G G G G . G G G G G G G G G G G G G G G G G G G G G G G G G G G G G G G G G G G G G G G G . G G G G G G G G G G G G G G G G G G G G G G G G G G G G G G G G    
957	. . . . . . . . . . . . . . . . . . . . . . . . . . . . . . . . . . . . . . . T . . . . . . . . . . . . . . . . T . T T T T T T T T T T T T T T T T T T T T T T T T T T T T T T T T T T T T T T T T T T T T T T T T T T T T T . T T T T T T T T T T T T T T T T T T T T T T T T T T T T T T T T T T T T T T T T . T T T T T T T T T T T T T T T T T T T T T T T T T T T T T T T T    
958	. . . . . . . . . . . . . . . . . . . . . . . . . . . . . . . . . . . . . . . G . . . . . . . . . . . . . . . . . . G G G G G G G G G G G G G G G G G G G G G G G G G G G G G G G G G G G G G G G G G G G G G G G G G G G G G . G G G G G G G G G G G G G G G G G G G G G G G G G G G G G G G G G G G G G G G G . G G G G G G G G G G G G G G G G G G G G G G G G G G G G G G G G    
959	. . . . . . . . . . . . . . . . . . . . . . . . . . . . . . . . . . . . . . . . . . . . . . . . . . . . . . . . . . G G G G G G G G G G G G G G G G G G G G G G G G G G G G G G G G G G G G G G G G G G G G G G G G G G G G G . G G G G G G G G G G G G G G G G G G G G G G G G G G G G G G G G G G G G G G G G . G G G G G G G G G G G G G G G G G G G G G G G G G G G G G G G G    
960	. . . . . . . . . . . . . . . . . . . . . . . . . . . . . . . . . . . . . . . . . . . . . . . . . . . . . . . . . . A A A A A A A A A A A A A A A A A A A A A A A A A A A A A A A A A A A A A A A A A A A A A A A A A A A A A . A A A A A A A A A A A A A A A A A A A A A A A A A A A A A A A A A A A A A A A A . A A A A A A A A A A A A A A A A A A A A A A A A A A A A A A A A    
961	. . . . . . . . . . . . . . . . . . . . . . . . . . . . . . . . . . . . . . . . . . . . . . . . . . . . . . . . . . T T T T T T T T T T T T T T T T T T T T T T T T T T T T T T T T T T T T T T T T T T T T T T T T T T T T T . T T T T T T T T T T T T T T T T T T T T T T T T T T T T T T T T T T T T T T T T . T T T T T T T T T T T T T T T T T T T T T T T T T T T T T T T T    
962	. . . . . . . . . . . . . . . . . . . . . . . . . . . . . . . . . . . . . . . . . . . . . . . . . . . . . . . . . . T T T T T T . T T T T T T T T T T T T T T T T T T T T T T T T T T T T T T T T T T T T T T T T T T T T T T . T T T T T T T T T T T T T T T T T T T T T T T T T T T T T T T T T T T T T T T T . T T T T T T T T T T T T T T T T T T T T T T T T T T T T T T T T    
963	. . . . . . . . . . . . . . . . . . . . . . . . . . . . . . . . . . . . . . . . . . . . . . . . . . . . . . . . . . T T T T T T . T T T T T T T T T T T T T T T T T T T T T T T T T T T T T T T T T T T T T T T T T T T T T T . T T T T T T T T T T T T T T T T T T T T T T T T T T T T T T T T T T T T T T T T . T T T T T T T T T T T T T T T T T T T T T T T T T T T T T T T T    
964	. . . . . . . . . . . . . . . . . . . . . . . . . . . . . . . . . . . . . . . . . . . . . . . . . . . . . . . . . . G G G G G G . G G G G G G G G G G G G G G G G G G G G G G G G G G G G G G G G G G G G G G G G G G G G G G . G G G G G G G G G G G G G G G G G G G G G G G G G G G G G G G G G G G G G G G G . G G G G G G G G G G G G G G G G G G G G G G G G G G G G G G G G    
965	. . . . . . . . . . . . . . . . . . . . . . . . . . . . . . . . . . . . . . . . . . . . . . . . . . . . . . . . . . G G G G G G . G G G G G G G G G G G G G G G G G G G G G G G G G G G G G G G G G G G G G G G G G G G G G G . G G G G G G G G G G G G G G G G G G G G G G G G G G G G G G G G G G G G G G G G . G G G G G G G G G G G G G G G G G G G G G G G G G G G G G G G G    
966	. . . . . . . . . . . . . . . . . . . . . . . . . . . . . . . . . . . . . . . . . . . . . . . . . . . . . . . . . . A A A A A G . A A A A A A A A A A A A A A A A A A A A A A A A A A A A A A A A A A A A A A A A A A A A A A . A A A A A A A A A A A A A A A A A A A A A A A A A A A A A A A A A A A A A A A A . A G A A A A A A A A A A A A A A A A A A A A A A A A A A A A A A    
967	. . . . . . . . . . . . . . . . . . . . . . . . . . . . . . . . . . . . . . . . . . . . . . . . . . . . . . . . . . A A A A A A . A A A A A A A A A A A A A A A A A A A A A A A A A A A A A A A A A A A A A A A A A A A A A A . A A A A A A A A A A A A A A A A A A A A A A A A A A A A A A A A A A A A A A A A . A A A A A A A A A A A A A A A A A A A A A A A A A A A A A A A A    
968	. . . . . . . . . . . . . . . . . . . . . . . . . . . . . . . . . . . . . . . . . . . . . . . . . . . . . . . . . . A A A A A A . A A A A A A A A A A A A A A A A A A A A A A A A A A A A A A A A A A A A A A A A A A A A A A . A A A A A A A A A A A A A A A A A A A A A A A A A A A A A A A A A A A A A A A A . A A A A A A A A A A A A A A A A A A A A A A A A A A A A A A A A    
969	. . . . . . . . . . . . . . . . . . . . . . . . . . . . . . . . . . . . . . . . . . . . . . . . . . . . . . . . . . C C C C C C . C C C C C C C C C C C C C C C C C C C C C C C C C C C C C C C C C C C C C C C C C C C C C C . C C C C C C C C C C C C C C C C C C C C C C C C C C C C C C C C C C C C C C C C . C C C C C C C C C C C C C C C C C C C C C C C C C C C C C C C C    
970	. . . . . . . . . . . . . . . . . . . . . . . . . . . . . . . . . . . . . . . . . . . . . . . . . . . . . . . . . . T T T T T T . . T T T T T T T T T T T T T T T T T T T T T T T T T T T T T T T T T T T T T T T T T T T T T . T T T T T T T T T T T T T T T T T T T T T T T T T T T T T T T T T T T T T T T T . T T T T T T T T T T T T T T T T T T T T T T T T T T T T T T T T    
971	. . . . . . . . . . . . . . . . . . . . . . . . . . . . . . . . . . . . . . . . . . . . . . . . . . . . . . . . . . A A A A A A . . A A A A A A A A A A A A A A A A A A A A A A A A A A A A A A A A A A A A A A A A A A A A A . A A A A A A A A A A A A A A A A A A A A A A A A A A A A A A A A A A A A A A A A . A A A A A A A A A A A A A A A A A A A A A A A A A A A A A A A A    
972	. . . . . . . . . . . . . . . . . . . . . . . . . . . . . . . . . . . . . . . . . . . . . . . . . . . . . . . . . . A A A A A A . . A A A A A A A A A A A A A A A A A A A A A A A A A A A A A A A A A A A A A A A A A A A A A . A A A A A A A A A A A A A A A A A A A A A A A A A A A A A A A A A A A A A A A A . A A A A A A A A A A A A A A A A A A A A A A A A A A A A A A A A    
973	. . . . . . . . . . . . . . . . . . . . . . . . . . . . . . . . . . . . . . . . . . . . . . . . . . . . . . . . . . C C C C C C . . C C C C C C C C C C C C C C C C C C C C C C C C C C C C C C C C C C C C C C C C C C C C C . C C C C C C C C C C C C C C C C C C C C C C C C C C C C C C C C C C C C C C C C . C C C C C C C C C C C C C C C C C C C C C C C C C C C C C C C C    
974	. . . . . . . . . . . . . . . . . . . . . . . . . . . . . . . . . . . . . . . . . . . . . . . . . . . . . . . . . . A A A A A A . . A A A A A A A A A A A A A A A A A A A A A A A A A A A A A A A A A A A A A A A A A A A A A . A A A A A A A A A A A A A A A A A A A A A A A A A A A A A A A A A A A A A A A A . A A A A A A A A A A A A A A A A A A A A A A A A A A A A A A A A    
975	. . . . . . . . . . . . . . . . . . . . . . . . . . . . . . . . . . . . . . . . . . . . . . . . . . . . . . . . . . A A A A A A . . A A A A A A A A A A A A A A A A A A A A A A A A A A A A A A A A A A A A A A A A A A A A A . A A A A A A A A A A A A A A A A A A A A A A A A A A A A A A A A A A A A A A A A . A A A A A A A A A A A A A A A A A A A A A A A A A A A A A A A A    
976	. . . . . . . . . . . . . . . . . . . . . . . . . . . . . . . . . . . . . . . . . . . . . . . . . . . . . . . . . . G G G G G G . . G G G G G G G G G G G G G G G G G G G G G G G G G G G G G G G G G G G G G G G G G G G G G . G G G G G G G G G G G G G G G G G G G G G G G G G G G G G G G G G G G G G G G G . G G G G G G G G G G G G G G G G G G G G G G G G G G G G G G G G    
977	. . . . . . . . . . . . . . . . . . . . . . . . . . . . . . . . . . . . . . . . . . . . . . . . . . . . . . . . . . T T T T . T . . T T T T T T T T T T T T T T T T T T T T T T T T T T T T T T T T T T T T T T T T T T T T T . T T T T T T T T T T T T T T T T T T T T T T T T T T T T T T T T T T T T T T T T . T T T T T T T T T T T T T T T T T T T T T T T T T T T T T T T T    
978	. . . . . . . . . . . . . . . . . . . . . . . . . . . . . . . . . . . . . . . . . . . . . . . . . . . . . . . . . . T T T T . T . . T T T T T T T T T T T T T T T T T T T T T T T T T T T T T T T T T T T T T T T T T T T T T . T T T T T T T T T T T T T T T T T T T T T T T T T T T T T T T T T T T T T T T T . T T T T T T T T T T T T T T T T T T T T T T T T T T T T T T T T    
979	. . . . . . . . . . . . . . . . . . . . . . . . . . . . . . . . . . . . . . . . . . . . . . . . . . . . . . . . . . . C C C . C . . C C C C C C C C C C C C C C C C C C C C C C C C C C C C C C C C C C C C C C C C C C C C C . C C C C C C C C C C C C C C C C C T C C C C C C C C C C C C C C C C C C C C C C . C C C C C C C C C C C C C C C C C C C C C C C C C C C C C C C C    
980	. . . . . . . . . . . . . . . . . . . . . . . . . . . . . . . . . . . . . . . . . . . . . . . . . . . . . . . . . . . T T T . T . . T T T T T T T T T T T T T T T T T T T T T T T T T T T T T T T T T T T T T T T T T T T T T . T T T T T T T T T T T T T T T T T T T T T T T T T T T T T T T T T T T T T T T T . T T T T T T T T T T T T T T T T T T T T T T T T T T T T T T T T    
981	. . . . . . . . . . . . . . . . . . . . . . . . . . . . . . . . . . . . . . . . . . . . . . . . . . . . . . . . . . . C C C . C . . C C C C C C C C C C C C C C C C C C C C C C C C C C C C C C C C C C C C C C C C C C C C C . C C C C C C C C C C C C C C C C C C C C C C C C C C C C C C C C C C C C C C C C . C C C C C C C C C C C C C C C C C C C C C C C C C C C C C C C C    
982	. . . . . . . . . . . . . . . . . . . . . . . . . . . . . . . . . . . . . . . . . . . . . . . . . . . . . . . . . . . T T T . T . . T T T T T T T T T T T T T T T T T T T T T T T T T T T T T T T T T T T T T T T T T T T T T . T T T T T T T T T T T T T T T T T T T T T T T T T T T T T T T T T T T T T T T T . T T T T T T T T T T T T T T T T T T T T T T T T T T T T T T T T    
983	. . . . . . . . . . . . . . . . . . . . . . . . . . . . . . . . . . . . . . . . . . . . . . . . . . . . . . . . . . . T T T . T . . T - - - - T T T - - - - - T - - T T T T T T - T T - T T T T - - - T T - T T T T T T T T T . - - - - - - - - - - - - - - - - - - - - - - - - - - - T T T T T T T T T T T T T . T T T T - T T - - - T T T T - - - - - - - T T T T - T T T T T T  56	 indel  
984	. . . . . . . . . . . . . . . . . . . . . . . . . . . . . . . . . . . . . . . . . . . . . . . . . . . . . . . . . . . T T T . T . . G - - - - T T T - - - - - T - - T T T T T T - T T - T T T T - - - T T - T T T T T T T T T . - - - - - - - - - - - - - - - - - - - - - - - - - - - T T T T T T T T T T T T T . T T T T - T T - - - T T T T - - - - - - - T T T T - T T T T T T  56	 indel  
985	. . . . . . . . . . . . . . . . . . . . . . . . . . . . . . . . . . . . . . . . . . . . . . . . . . . . . . . . . . . T T T . T . . . T T T T T T T T T T T T T T T T T T T T T T T T T T T T T T T T T T T T T T T T T T T T . T T T T T T T T T T T T T T T T T T T T T T T T T T T T T T T T T T T T T T T T . T T T T T T T T T T T T T T T T T T T T T T T T T T T T T T T T    
986	. . . . . . . . . . . . . . . . . . . . . . . . . . . . . . . . . . . . . . . . . . . . . . . . . . . . . . . . . . . G G G . G . . . G G G G G G G G G G G G G G G G G G G G G G G G G G G G G G G G G G G G G G G G G G G G . G G G G G G G G G G G G G G G G G G G G G G G G G G G G G G G G G G G G G G G G . G G G G G G G G G G G G G G G G G G G G G G G G G G G G G G G G    
987	. . . . . . . . . . . . . . . . . . . . . . . . . . . . . . . . . . . . . . . . . . . . . . . . . . . . . . . . . . . T T T . T . . . T T T T T T T T T T T T T T T T T T T T T T T T T T T T T T T T T T T T T T T T T T T T . T T T T T T T T T T T T T T T T T T T T T T T T T T T T T T T T T T T T T T T T . T T T T T T T T T T T T T T T T T T T T T T T T T T T T T T T T    
988	. . . . . . . . . . . . . . . . . . . . . . . . . . . . . . . . . . . . . . . . . . . . . . . . . . . . . . . . . . . T T T . T . . . T T T T T T T T T T T T T T T T T T T T T T T T T T T T T T T T T T T T T T T T T T T T . T T T T T T T T T T T T T T T T T T T T T T T T T T T T T T T T T T T T T T T T . T T T T T T T T T T T T T T T T T T T T T T T T T T T T T T T T    
989	. . . . . . . . . . . . . . . . . . . . . . . . . . . . . . . . . . . . . . . . . . . . . . . . . . . . . . . . . . . G G G . G . . . G G G G G G G G G G G G G G G G G G G G G G G G G G G G G G G G G G G G G G G G G G G G . G G G G G G G G G G G G G G G G G G G G G G G G G G G G G G G G G G G G G G G G . G G G G G G G G G G G G G G G G G G G G G G G G G G G G G G G G    
990	. . . . . . . . . . . . . . . . . . . . . . . . . . . . . . . . . . . . . . . . . . . . . . . . . . . . . . . . . . . T T . . T . . . T T T T T T T T T T T T T T T T T T T T T T T T T T T T T T T T T T T T T T T T T T T T . T T T T T T T T T T T T T T T T T T T T T T T T T T T T T T T T T T T T T T T T . T T T T T T T T T T T T T T T T T T T T T T T T T T T T T T T T    
991	. . . . . . . . . . . . . . . . . . . . . . . . . . . . . . . . . . . . . . . . . . . . . . . . . . . . . . . . . . . A A . . A . . . A A A A A A A A A A A A A A A A A A A A A A A A A A A A A A A A A A A A A A A A A A A A . A A A A A A A A A A A A A A A A A A A A A A A A A A A A A A A A A A A A A A A A A A A A A A A A A A A A A A A A A A A A A A A A A A A A A A A A A    
992	. . . . . . . . . . . . . . . . . . . . . . . . . . . . . . . . . . . . . . . . . . . . . . . . . . . . . . . . . . . T T . . T . . . T T T T T T T T T T T T T T T T T T T T T T T T T T T T T T T T T T T T T T T T T T T T . T T T T T T T T T T T T T T T T T T T T T T T T T T T T T T T T T T T T T T T T T T T T T T T T T T T T T T T T T T T T T T T T T T T T T T T T T    
993	. . . . . . . . . . . . . . . . . . . . . . . . . . . . . . . . . . . . . . . . . . . . . . . . . . . . . . . . . . . T T . . T . . . T T T T T T T T T T T T T T T T T T T T T T T T T T T T T T T T T T T T T T T T T T T T . T T T T T T T T T T T T T T T T T T T T T T T T T T T T T T T T T T T T T T T T T T T T T T T T T T T T T T T T T T T T T T T T T T T T T T T T T    
994	. . . . . . . . . . . . . . . . . . . . . . . . . . . . . . . . . . . . . . . . . . . . . . . . . . . . . . . . . . . T T . . T . . . T . T T T T T T T T T T T T T T T T T T T T T T T T T T T T T T T T T T T T T T T T T T . T T T T T T T T T T T T T T T T T T T T T T T T T T T T T T T T T T T T T T T T T T T T T T T T T T T T T T T T T T T T T T T T T T T T T T T T T    
995	. . . . . . . . . . . . . . . . . . . . . . . . . . . . . . . . . . . . . . . . . . . . . . . . . . . . . . . . . . . T T . . T . . . T . T C T T T T T T T T T T T T T T T T T T T T T T T T T T T T T T T T T T T T T T T T . T T T T T T T T T T T T T T T T T T T T T T T T T T T T T T T T T T T T T T T T T T T T T T T T T T T T T T T T T T T T T T T T T T T T T T T T T    
996	. . . . . . . . . . . . . . . . . . . . . . . . . . . . . . . . . . . . . . . . . . . . . . . . . . . . . . . . . . . C C . . C . . . C . C . C C C C C C C C C C C C C C C C C C C C C C C C C C C C C C C C C C C C C C C C . C C C C C C C C C C C C C C C C C T C C C C C C C C C C C C C C C C C C C C C C C C C C C C C C C C C C C C C C C C C C C C C C C C C C C C C C C    
997	. . . . . . . . . . . . . . . . . . . . . . . . . . . . . . . . . . . . . . . . . . . . . . . . . . . . . . . . . . . T T . . T . . . T . T . T T T T T T T T T T T T T T T T T T T T T T T T T T T T T T T T T T T T T T T T . T T T T T T T T T T T T T T T T T T T T T T T T T T T T T T T T T T T T T T T T T T T T T T T T T T T T T T T T T T T T T T T T T T T T T T T T T    
998	. . . . . . . . . . . . . . . . . . . . . . . . . . . . . . . . . . . . . . . . . . . . . . . . . . . . . . . . . . . T T . . . . . . T . T . T T T T T T T T T T T T T T T T T T T T T T T T T T T T T T T T T T T T T T T T . T T T T T T T T T T T T T T T T T T T T T T T T T T T T T T T T T T T T T T T T T T T T T T T T T T T T T T T T T T T T T T T T T T T T T T T T T    
999	. . . . . . . . . . . . . . . . . . . . . . . . . . . . . . . . . . . . . . . . . . . . . . . . . . . . . . . . . . . T T . . . . . . T . T . T T T T T T T T T T T T T T T T T T T T T T T T T T T T T T T T T T T T T T T T . T T T T T T T T T T T T T T T T T T T T T T T T T T T T T T T T T T T T T T T T T T T T T T T T T T T T T T T T T T T T T T T T T T T T T T T T T    
1000	. . . . . . . . . . . . . . . . . . . . . . . . . . . . . . . . . . . . . . . . . . . . . . . . . . . . . . . . . . . A A . . . . . . A . A . A A A A A A A A A A A A A A A A A A A A A A A A A A A A A A A A A A A A A A A A . A A A A A A A A A A A A A A A A A A A A A A A A A A A A A A A A A A A A A A A A A A A A A A A A A A A A A A A A A A A A A A A A A A A A A A A A A    
1001	. . . . . . . . . . . . . . . . . . . . . . . . . . . . . . . . . . . . . . . . . . . . . . . . . . . . . . . . . . . T T . . . . . . T . T . T T T T T T T T T T T T T T T T T T T T T T T T T T T T T T T T T T T T T T T T . T T T T T T T T T T T T T T T T T T T T T T T T T T T T T T T T T T T T T T T T T T T T T T T T T T T T T T T T T T T T T T T T T T T T T T T T T    
1002	. . . . . . . . . . . . . . . . . . . . . . . . . . . . . . . . . . . . . . . . . . . . . . . . . . . . . . . . . . . G . . . . . . . G . G . G G G G G G G G G G G G G G G G G G G G G G G G G G G G G G G G G G G G G G G G . G G G G G G G G G G G G G G G G G G G G G G G G G G G G G G G G G G G G G G G G G G G G G G G G G G G G G G G G G G G G G G G G G G G G G G G G G    
1003	. . . . . . . . . . . . . . . . . . . . . . . . . . . . . . . . . . . . . . . . . . . . . . . . . . . . . . . . . . . G . . . . . . . G . G . G G G G G G G G G G G G G G G G G G G G G G G G G G G G G G G G G G G G G G G G . G G G G G G G G G G G G G G G G G G G G G G G G G G G G G G G G G G G G G G G G G G G G G G G G G G G G G G G G G G G G G G G G G G G G G G G G G    
1004	. . . . . . . . . . . . . . . . . . . . . . . . . . . . . . . . . . . . . . . . . . . . . . . . . . . . . . . . . . . G . . . . . . . G . G . G G G G G G G G G G G G G G G G G G G G G G G G G G G G G G G G G G G G G G G G . G G G G G G G G G G G G G G G G G G G G G G G G G G G G G G G G G G G G G G G G G G G G G G G G G G G G G G G G G G G G G G G G G G G G G G G G G    
1005	. . . . . . . . . . . . . . . . . . . . . . . . . . . . . . . . . . . . . . . . . . . . . . . . . . . . . . . . . . . - . . . . . . . - . - . - - - - - - - - - - - - - - - - - - - - - - - - - - - - - - - - - - - - - - - - . - - - - - - - - - - - - - - - - - - - - - - - - - - - - - - - - - - - - - - - - - - G - - - - - - - - - - - - - - - - - - - - - - - - - - - - - -    
1006	. . . . . . . . . . . . . . . . . . . . . . . . . . . . . . . . . . . . . . . . . . . . . . . . . . . . . . . . . . . T . . . . . . . T . T . T T T T T T T T T T T T T T T T T T T T T T T T T T T T T T T T T T T T T T T T . T T T T T T T T T T T T T T T T T T T T T T T T T T T T T T T T T T T T T T T T T T T T T T T T T T T T T T T T T T T T T T T T T T T T T T T T T    
1007	. . . . . . . . . . . . . . . . . . . . . . . . . . . . . . . . . . . . . . . . . . . . . . . . . . . . . . . . . . . C . . . . . . . C . C . C C C C C C C C C C C C C C C C C C C C C C C C C C C C C C C C C C C C C C C C . C C C C C C C C C C C C C C C C C C C C C C C C C C C C C C C C C C C C C C C C C C C C C C C C C C C C C C C C C C C C C C C C C C C C C C C C C    
1008	. . . . . . . . . . . . . . . . . . . . . . . . . . . . . . . . . . . . . . . . . . . . . . . . . . . . . . . . . . . T . . . . . . . T . . . T T T T T T T T T T T T T T T T T T T T T T T T T T T T T T T T T T T T T T T T . T T T T T T T T T T T T T T T T T T T T T T T T T T T T T T T T T T T T T T T T T T T T T T T T T T T T T T T T T T T T T T T T T T T T T T T T T    
1009	. . . . . . . . . . . . . . . . . . . . . . . . . . . . . . . . . . . . . . . . . . . . . . . . . . . . . . . . . . . A . . . . . . . A . . . A A A A A A A A A A A A A A A A A A A A A A A A A A A A A A A A A A A A A A A A . A A A A A A A A A A A A A A A A A A A A A A A A A A A A A A A A A A A A A A A A A A A A A A A A A A A A A A A A A A A A A A A A A A A A A A A A A    
1010	. . . . . . . . . . . . . . . . . . . . . . . . . . . . . . . . . . . . . . . . . . . . . . . . . . . . . . . . . . . T . . . . . . . T . . . T T T T T T T T T T T T T T T T T T T T T T T T T T T T T T T T T T T T T T T T . T T T T T T T T T T T T T T T T T T T T T T T T T T T T T T T T T T T T T T T T T T T T T T T T T T T T T T T T T T T T T T T T T T T T T T T T T    
1011	. . . . . . . . . . . . . . . . . . . . . . . . . . . . . . . . . . . . . . . . . . . . . . . . . . . . . . . . . . . T . . . . . . . T . . . T T T T T T T T T T T T T T T T T T T T T T T T T T T T T T T T T T T T T T T T . T T T T T T T T T T T T T T T T T T T T T T T T T T T T T T T T T T T T T T T T T T T T T T T T T T T T T T T T T T T T T T T T T T T T T T T T T    
1012	. . . . . . . . . . . . . . . . . . . . . . . . . . . . . . . . . . . . . . . . . . . . . . . . . . . . . . . . . . . T . . . . . . . T . . . T T T T T T T T T T T T T T T T T T T T T T T T T T T T T T T T T T T T T T T T . T T T T T T T T T T T T T T T T T T T T T T T T T T T T T T T T T T T T T T T T T T T T T T T T T T T T T T T T T T T T T T T T T T T T T T T T T    
1013	. . . . . . . . . . . . . . . . . . . . . . . . . . . . . . . . . . . . . . . . . . . . . . . . . . . . . . . . . . . A . . . . . . . A . . . A A A A A A A A A A A A A A A A A A A A A A A A A A A A A A A A A A A A A A A A . A A A A A A A A A A A A A A A A A A A A A A A A A A A A A A A A A A A A A A A A A A A A A A A A A A A A A A A A A A A A A A A A A A A A A A A A A    
1014	. . . . . . . . . . . . . . . . . . . . . . . . . . . . . . . . . . . . . . . . . . . . . . . . . . . . . . . . . . . A . . . . . . . A . . . A A A A A A A A A A A A A A A A A A A A A A A A A A A A A A A A A A A A A A A A . A A A A A A A A A A A A A A A A A A A A A A A A A A A A A A A A A A A A A A A A A A A A A A A A A A A A A A A A A A A A A A A A A A A A A A A A A    
1015	. . . . . . . . . . . . . . . . . . . . . . . . . . . . . . . . . . . . . . . . . . . . . . . . . . . . . . . . . . . . . . . . . . . . . . . T T T T T T T T T T T T T T T T T T T T T T T T T T T T T T T T T T T T T T T T . T T T T T T T T T T T T T T T T T T T T T T T T T T T T T T T T T T T T T T T T T T T T T T T T T T T T T T T T T T T T T T T T T T T T T T T T T    
1016	. . . . . . . . . . . . . . . . . . . . . . . . . . . . . . . . . . . . . . . . . . . . . . . . . . . . . . . . . . . . . . . . . . . . . . . T T T T T T T T T T T T T T T T T T - T T T T T T T T T T T T T T T T T T T T T . T T T T T T T T T T T T T T T T T T T T T T T T T T T T T T T T T T T T T T T T T T T T T T T T T T T T T T T T T T T T T T T T T T T T T T T T T    
1017	. . . . . . . . . . . . . . . . . . . . . . . . . . . . . . . . . . . . . . . . . . . . . . . . . . . . . . . . . . . . . . . . . . . . . . . A A A A A A A A A A A A A A A A A A A A A A A A A A A A A A A A A A A A A A A A . A A A A A A A A A A A A A A A A A A A A A A A A A A A A A A A A A A A A A A A A A A A A A A A A A A A A A A A A A A A A A A A A A A A A A A A A A    
1018	. . . . . . . . . . . . . . . . . . . . . . . . . . . . . . . . . . . . . . . . . . . . . . . . . . . . . . . . . . . . . . . . . . . . . . . A A A A A A A A A A A A A A A A A A A A A A A A A A A A A A A A A A A A A A A A . A A A A A A A A A A A A A A A A A A A A A A A A A A A A A A A A A A A A A A A A A A A A A A A A A A A A A A A A A A A A A A A A A A A A A A A A A    
1019	. . . . . . . . . . . . . . . . . . . . . . . . . . . . . . . . . . . . . . . . . . . . . . . . . . . . . . . . . . . . . . . . . . . . . . . T T T T T T T T T T T T T T T T T T T T T T T T T T T T T T T T T T T T T T T T . T T T T T T T T T T T T T T T T T T T T T T T T T T T T T T T T T T T T T T T T T T T T T T T T T T T T T T T T T T T T T T T T T T T T T T T T T    
1020	. . . . . . . . . . . . . . . . . . . . . . . . . . . . . . . . . . . . . . . . . . . . . . . . . . . . . . . . . . . . . . . . . . . . . . . C C C C C C C C C C C C C C C C C C C C C C C C C C C C C C C C C C C C C C C C . C C T C C C C C C C C C C C C C C T C C C C C C C C C C C C C C C C C C C C C C C C C C C C C C C C C C C C C C C C C C C C C C C C C C C C C C C    
1021	. . . . . . . . . . . . . . . . . . . . . . . . . . . . . . . . . . . . . . . . . . . . . . . . . . . . . . . . . . . . . . . . . . . . . . . T T T T T T T T T T T T T T T T T T T T T T T T T T T T T T T T T T T T T T T T . T T T T T T T T T T T T T T T T T T T T T T T T T T T T T T T T T T T T T T T T T T T T T T T T T T T T T T T T T T T T T T T T T T T T T T T T T    
1022	. . . . . . . . . . . . . . . . . . . . . . . . . . . . . . . . . . . . . . . . . . . . . . . . . . . . . . . . . . . . . . . . . . . . . . . A A A A A A A A A A A A A A A A A A A A A A A A A A A A A A A A A A A A A A A A . A A A A A A A A A A A A A A A A A A A A A A A A A A A A A A A A A A A A A A A A A A A A A A A A A A A A A A A A A A A A A A A A A A A A A A A A A    
1023	. . . . . . . . . . . . . . . . . . . . . . . . . . . . . . . . . . . . . . . . . . . . . . . . . . . . . . . . . . . . . . . . . . . . . . . G G G G G G G G G G G G G G G G G G G G G G G G G G G G G G G G G G G G G G G G . G G G G G G G G G G G G G G G G G G G G G G G G G G G G G G G G G G G G G G G G G G G G G G G G G G G G G G G G G G G G G G G G G G G G G G G G G    
1024	. . . . . . . . . . . . . . . . . . . . . . . . . . . . . . . . . . . . . . . . . . . . . . . . . . . . . . . . . . . . . . . . . . . . . . . G G G G . G G G G G G G G G G G G G G G G G G G G G G G G G G G G G G G G G G G . G G G G G G G G G G G G G G G G G G G G G G G G G G G G G G G G G G G G G G G G G G G G G G G G G G G G G G G G G G G G G G G G G G G G G G G G G    
1025	. . . . . . . . . . . . . . . . . . . . . . . . . . . . . . . . . . . . . . . . . . . . . . . . . . . . . . . . . . . . . . . . . . . . . . . A A A A . A A A A A A A A A A A A A A A A A A A A A A A A A A A A A A A A A A A . A A A A A A A A A A A A A A A A A A A A A A A A A A A A A A A A A A A A A A A A A A A A A A A A A A A A A A A A A A A A A A A A A A A A A A A A A    
1026	. . . . . . . . . . . . . . . . . . . . . . . . . . . . . . . . . . . . . . . . . . . . . . . . . . . . . . . . . . . . . . . . . . . . . . . T T T T . A T T T T T T T T T T T T T T T T T T T T T T T T T T T T T T T T T T . T T T T T T T T T T T T T T T T T T T T T T T T T T T T T T T T T T T T T T T T T T T T T T T T T T T T T T T T T T T T T T T T T T T T T T T T T    
1027	. . . . . . . . . . . . . . . . . . . . . . . . . . . . . . . . . . . . . . . . . . . . . . . . . . . . . . . . . . . . . . . . . . . . . . . T T T T . . T T T T T T T T T T T T T T T T T T T T T T T T T T T T T T T T T T . T T T T T T T T T T T T T T T T T T T T T T T T T T T T T T T T T T T T T T T T T T T T T T T T T T T T T T T T T T T T T T T T T T T T T T T T T    
1028	. . . . . . . . . . . . . . . . . . . . . . . . . . . . . . . . . . . . . . . . . . . . . . . . . . . . . . . . . . . . . . . . . . . . . . . T T T T . . T T T T T T T T T T T T T T T T T T T T T T T T T T T T T T T T T T . T T T T T T T T T T T T T T T T T T T T T T T T T T T T T T T T T T T T T T T T T T T T T T T T T T T T T T T T T T T T T T T T T T T T T T T T T    
1029	. . . . . . . . . . . . . . . . . . . . . . . . . . . . . . . . . . . . . . . . . . . . . . . . . . . . . . . . . . . . . . . . . . . . . . . T T T T . . T T T T T T T T T T T T T T T T T T T T T T T T T T T T T T T T T T . T T T T T T T T T T T T T T T T T T T T T T T T T T T T T T T T T T T T T T T T T T T T T T T T T T T T T T T T T T T T T T T T T T T T T T T T T    
1030	. . . . . . . . . . . . . . . . . . . . . . . . . . . . . . . . . . . . . . . . . . . . . . . . . . . . . . . . . . . . . . . . . . . . . . . G G G G . . G G G G G G G G G G G G G G G G G G G G G G G G G G G G G G G G G G . G G G G G G G G G G G G G G G G G G G G G G G G G G G G G G G G G G G G G G G G G G G G G G G G G G G G G G G G G G G G G G G G G G G G G G G G G    
1031	. . . . . . . . . . . . . . . . . . . . . . . . . . . . . . . . . . . . . . . . . . . . . . . . . . . . . . . . . . . . . . . . . . . . . . . A . A A . . A A A A A A A A A A A A A A A A A A A A A A A A A A A A A A A A A A . A A A A A A A A A A A A A A A A A A A A A A A A A A A A A A A A A A A A A A A A A A A A A A A A A A A A A A A A A A A A A A A A A A A A A A A A A    
1032	. . . . . . . . . . . . . . . . . . . . . . . . . . . . . . . . . . . . . . . . . . . . . . . . . . . . . . . . . . . . . . . . . . . . . . . T . T T . . T T T T T T T T T T T T T T T T T T T T T T T T T T T T T T T T T T . T T T T T T T T T T T T T T T T T T T T T T T T T T T T T T T T T T T T T T T T T T T T T T T T T T T T T T T T T T T T T T T T T T T T T T T T T    
1033	. . . . . . . . . . . . . . . . . . . . . . . . . . . . . . . . . . . . . . . . . . . . . . . . . . . . . . . . . . . . . . . . . . . . . . . A . A A . . A A G A A A A A A A A A A A A A A A A A A A A A A A A A A A A A A A . A A A A A A A A A A A A A A A A A A A A A A A A A A A A A A A A A A A A A A A A A A A A A A A A A A A A A A A A A A A A A A A A A A A A A A A A A    
1034	. . . . . . . . . . . . . . . . . . . . . . . . . . . . . . . . . . . . . . . . . . . . . . . . . . . . . . . . . . . . . . . . . . . . . . . T . T T . . T T . T T T T T T T T T T T T T T T T T T T T T T T T T T T T T T T . T T T T T T T T T T T T T T T T T T T T T T T T T T T T T T T T T T T T T T T T T T T T T T T T T T T T T T T T T T T T T T T T T T T T T T T T T    
1035	. . . . . . . . . . . . . . . . . . . . . . . . . . . . . . . . . . . . . . . . . . . . . . . . . . . . . . . . . . . . . . . . . . . . . . . A . A A . . A A . A A A A A A A A A A A A A A A A A A A A A A A A A A A A A A A . A A A A A A A A A A A A A A A A A A A A A A A A A A A A A A A A A A A A A A A A A A A A A G A A A A A A A A A A A A A A A A A A A A A A A A A A A    
1036	. . . . . . . . . . . . . . . . . . . . . . . . . . . . . . . . . . . . . . . . . . . . . . . . . . . . . . . . . . . . . . . . . . . . . . . A . A A . . A A . A A A A A A A A A A A A A A A A A A A A A A A A A A A A A A A . A A A A A A A A A A A A A A A A A A A A A A A A A A A A A A A A A A A A A A A A A A A A A A A A A A A A A A A A A A A A A A A A A A A A A A A A A    
1037	. . . . . . . . . . . . . . . . . . . . . . . . . . . . . . . . . . . . . . . . . . . . . . . . . . . . . . . . . . . . . . . . . . . . . . . T . T T . . T T . T T T T T T T T T T T T T T T T T T T T T T T T T T T T T T T . T T T T T T T T T T T T T T T T T T T T T T T T T T T T T T T T T T T T T T T T T T T T T T T T T T T T T T T T T T T T T T T T T T T T T T T T T    
1038	. . . . . . . . . . . . . . . . . . . . . . . . . . . . . . . . . . . . . . . . . . . . . . . . . . . . . . . . . . . . . . . . . . . . . . . A . A A . . A A . A A A A A A A A A A A A A A A A A A A A A A A A A A A A A A A . A A A A A A A A A A A A A A A A A A A A A A A A A A A A A A A A A A A A A A A A A A A A A A A A A A A A A A A A A A A A A A A A A A A A A A A A A    
1039	. . . . . . . . . . . . . . . . . . . . . . . . . . . . . . . . . . . . . . . . . . . . . . . . . . . . . . . . . . . . . . . . . . . . . . . A . A G . . A A . A A A A A A A A A A A A A A A A A A A A A A A A A A A A A A A . A A A A A A A A A A A A A A A A A A A A A A A A A A A A A A A A A A A A A A A A A A A A A A A A A A A A A A A A A A A A A A A A A A A A A A A A A    
1040	. . . . . . . . . . . . . . . . . . . . . . . . . . . . . . . . . . . . . . . . . . . . . . . . . . . . . . . . . . . . . . . . . . . . . . . G . G . . . G G . G G G G T G G G G G G G G G G G G G G G G G G G G G G G G G G . G G G G G G G G G G G G G G G G G G G G G G G G G G G G G G G G G G G G G G G G G G G G G G G G G G G G G G G G G G G G G G G G G G G G G G G G G    
1041	. . . . . . . . . . . . . . . . . . . . . . . . . . . . . . . . . . . . . . . . . . . . . . . . . . . . . . . . . . . . . . . . . . . . . . . G . G . . . G G . G G G G . G G G G G G G G G G G G G G G G G G G G G G G G G G . G G G G G G G G G G G G G G G G G G G G G G G G G G G G G G G G G G G G G G G G G G G G G G G G G G G G G G G G G G G G G G G G G G G G G G G G G    
1042	. . . . . . . . . . . . . . . . . . . . . . . . . . . . . . . . . . . . . . . . . . . . . . . . . . . . . . . . . . . . . . . . . . . . . . . C . C . . . C C . C C C C . C C C C C C C C C C C C C C C C C C C C C C C C C C . C C C C C C C C C C C C C C C C C C C C C C C C C C C C C C C C C C C C C C C C C C C C C C C C C C C C C C C C C C C C C C C C C C C C C C C C C    
1043	. . . . . . . . . . . . . . . . . . . . . . . . . . . . . . . . . . . . . . . . . . . . . . . . . . . . . . . . . . . . . . . . . . . . . . . C . C . . . C C . C C C C . C C C C C C C C C C C C C C C C C C C C C C C C C C . C C C C C C C C C C C C C C C C C C C C C C C C C C C C C C C C C C C C C C C C C C C C C C C C C C C C C C C C C C C C C C C C C C C C C C C C C    
1044	. . . . . . . . . . . . . . . . . . . . . . . . . . . . . . . . . . . . . . . . . . . . . . . . . . . . . . . . . . . . . . . . . . . . . . . A . A . . . A A . A A A A . A A A A A A A A A A A A A A A A A A A A A A A A A A . A A A A A A A A A A A A A A A A A A A A A A A A A A A A A A A A A A A A A A A A A A A A A A A A A A A A A A A A A A A A A A A A A A A A A A A A A    
1045	. . . . . . . . . . . . . . . . . . . . . . . . . . . . . . . . . . . . . . . . . . . . . . . . . . . . . . . . . . . . . . . . . . . . . . . G . G . . . G G . G G G G . G G G G G G G G G G G G G G G G G G G G G G G G G G . G G G G G G G G G G G G G G G G G G G G G G G G G G G G G G G G G G G G G G G G G G G G G G G G G G G G G G G G G G G G G G G G G G G G G G G G G    
1046	. . . . . . . . . . . . . . . . . . . . . . . . . . . . . . . . . . . . . . . . . . . . . . . . . . . . . . . . . . . . . . . . . . . . . . . G . G . . . G G . G G G G . G G G G G G G G G G G G G G G G G G G G G G G G G G . G G G G G G G G G G G G G G G G G G G G G G G G G G G G G G G G G G G G G G G G G G G G G G G G G G G G G G G G G G G G G G G G G G G G G G G G G    
1047	. . . . . . . . . . . . . . . . . . . . . . . . . . . . . . . . . . . . . . . . . . . . . . . . . . . . . . . . . . . . . . . . . . . . . . . T . T . . . G G . G G T T . T T T G T T G T T T T G G G T T G T T T T T T T T T . G G G G G G G G G G G G G G G G G G G G G G G G G G G T T T T T T T T T T T T T T T T T T G T T G G G T T T T G G G G G G G T T T T G T T T T T T  49	 transversion  
1048	. . . . . . . . . . . . . . . . . . . . . . . . . . . . . . . . . . . . . . . . . . . . . . . . . . . . . . . . . . . . . . . . . . . . . . . G . G . . . G G . G G G G . G G G G G G G G G G G G G G G G G G G G G G G G G G . G G G G G G G G G G G G G G G G G G G G G G G G G G G G G G G G G G G G G G G G G G G G G G G G G G G G G G G G G G G G G G G G G G G G G G G G G    
1049	. . . . . . . . . . . . . . . . . . . . . . . . . . . . . . . . . . . . . . . . . . . . . . . . . . . . . . . . . . . . . . . . . . . . . . . T . T . . . T T . T T T T . T T T T T T T T T T T T T T T T T T T T T T T T T T . T T T T T T T T T T T T T T T T T T T T T T T T T T T T T T T T T T T T T T T T T T T T T T T T T T T T T T T T T T T T T T T T T T T T T T T T T    
1050	. . . . . . . . . . . . . . . . . . . . . . . . . . . . . . . . . . . . . . . . . . . . . . . . . . . . . . . . . . . . . . . . . . . . . . . A . A . . . A A . A A A A . A A A A A A A A A A A A A A A A A A A A A A A A A A . A A A A A A A A A A A A A A A A A A A A A A A A A A A A A A A A A A A A A A A A A A A A A A A A A A A A A A A A A A A A A A A A A A A A A A A A A    
1051	. . . . . . . . . . . . . . . . . . . . . . . . . . . . . . . . . . . . . . . . . . . . . . . . . . . . . . . . . . . . . . . . . . . . . . . C . C . . . C C . C C C C . C C C C C C C C C C C C C C C C C C C C C T C C C C . C C C C C C C C C C C C C C C C C C C C C C C C C C C C C C C C C C C C C C C C C C C C C C C C C C C C C C C C C C C C C C C C C C C C C C C C C    
1052	. . . . . . . . . . . . . . . . . . . . . . . . . . . . . . . . . . . . . . . . . . . . . . . . . . . . . . . . . . . . . . . . . . . . . . . T . T . . . T T . T C C T . T C T T T T T T T T T T T T T T T T T T T T T T T T . T T T T T T T T T T T T T T T T T T T T T T T T T T T T T T T T T T T T T T T T T T T T T T T T T T T T T T T T T T T T T T T T T T T T T T T T T    
1053	. . . . . . . . . . . . . . . . . . . . . . . . . . . . . . . . . . . . . . . . . . . . . . . . . . . . . . . . . . . . . . . . . . . . . . . A . A . . . A A . A . . A . A . A A A A A A A A A A A A A A A A A A A A A A A A . A A A A A A A A A A A A A A A A A A A A A A A A A A A A A A A A A A A A A A A A A A A A A A A A A A A A A A A A A A A A A A A A A A A A A A A A A    
1054	. . . . . . . . . . . . . . . . . . . . . . . . . . . . . . . . . . . . . . . . . . . . . . . . . . . . . . . . . . . . . . . . . . . . . . . A . A . . . A A . A . . A . A . A A A A A A A A A A A A A A A A A A A A A A A A . A A A A A A A A A A A A A A A A A A A A A A A A A A A A A A A A A A A A A A A A A A A A A A A A A A A A A A A A A A A A A A A A A A A A A A A A A    
1055	. . . . . . . . . . . . . . . . . . . . . . . . . . . . . . . . . . . . . . . . . . . . . . . . . . . . . . . . . . . . . . . . . . . . . . . C . C . . . C C . C . . C . C . C C C C C C C C C C C C C C C C C C C C C C C C . C C C C C C C C C C C C C C C C C C C C C C C C C C C C C C C C C C C C C C C C C C C C C C C C C C C C C C C C C C C C C C C C C C C C C C C C C    
1056	. . . . . . . . . . . . . . . . . . . . . . . . . . . . . . . . . . . . . . . . . . . . . . . . . . . . . . . . . . . . . . . . . . . . . . . T . T . . . T T . T . . T . T . T T T T T T T T T T T T T T T T T T T T T T T T . T T T T T T T T T T T T T T T T T T T T T T T T T T T T T T T T T T T T T T T T T T T T T T T T T T T T T T T T T T T T T T T T T T T T T T T T T    
1057	. . . . . . . . . . . . . . . . . . . . . . . . . . . . . . . . . . . . . . . . . . . . . . . . . . . . . . . . . . . . . . . . . . . . . . . G . G . . . G G . G . . G . G . G G G G G G G G G G G G G G G G G G G G G G G G . G G G G G G G G G G G G G G G G G G G G G G G G G G G G G G G G G G G G G G G G G G G G G G G G G G G G G G G G G G G G G G G G G G G G G G G G G    
1058	. . . . . . . . . . . . . . . . . . . . . . . . . . . . . . . . . . . . . . . . . . . . . . . . . . . . . . . . . . . . . . . . . . . . . . . C . C . . . C C . C . . C . C . C C C C C C C C C C C C C C C C C C C C C C C C . C C C C C C C C C C C C C C C C C C C C C C C C C C C C C C C C C C C C C C C C C C C C C C C C C C C C C C C C C C C C C C C C C C C C C C C C C    
1059	. . . . . . . . . . . . . . . . . . . . . . . . . . . . . . . . . . . . . . . . . . . . . . . . . . . . . . . . . . . . . . . . . . . . . . . T . T . . . T T . T . . T . T . T T T T T T T T T T T T T T T T T T T T T T T T . T T T T T T T T T T T T T T T T T T T T T T T T T T T T T T T T T T T T T T T T T T T T T T T T T T T T T T T T T T T T T T T T T T T T T T T T T    
1060	. . . . . . . . . . . . . . . . . . . . . . . . . . . . . . . . . . . . . . . . . . . . . . . . . . . . . . . . . . . . . . . . . . . . . . . G . G . . . G G . G . . G . G . G G G G G G G G G G G G G G G G G G G G G G G G . G G G G G G G G G G G G G G G G G G A G G G G G G G G G G G G G G G G G G G G G G G G G G G G G G G G G G G G G G G G G G G G G G G G G G G G G G    
1061	. . . . . . . . . . . . . . . . . . . . . . . . . . . . . . . . . . . . . . . . . . . . . . . . . . . . . . . . . . . . . . . . . . . . . . . C . C . . . C C . C . . C . C . C C C C C C C C C C C C C C C C C C C C C C C C . C C C C C C C C C C C C C C C C C C C C C C C C C C C C C C C C C C C C C C C C C C C C C C C C C C C C C C C C C C C C C C C C C C C C C C C C C    
1062	. . . . . . . . . . . . . . . . . . . . . . . . . . . . . . . . . . . . . . . . . . . . . . . . . . . . . . . . . . . . . . . . . . . . . . . T . T . . . T T . T . . T . T . T T T T T T T T T T T T T T T T T T T T T T T T . T T T T T T T T T T T T T T T T T T T T T T T T T T T T T T T T T T T T T T T T T T T T T T T T T T T T T T T T T T T T T T T T T T T T T T T T T    
1063	. . . . . . . . . . . . . . . . . . . . . . . . . . . . . . . . . . . . . . . . . . . . . . . . . . . . . . . . . . . . . . . . . . . . . . . C . C . . . C C . C . . C . C . C C C C C C C C C C C C C C C C C C C C C C C C . C C C C C C C C C C C C C C C C C C C C C C C C C C C C C C C C C C C C C C C C C C C C C C C C C C C C C C C C C C C C C C C C C C C C C C C C C    
1064	. . . . . . . . . . . . . . . . . . . . . . . . . . . . . . . . . . . . . . . . . . . . . . . . . . . . . . . . . . . . . . . . . . . . . . . A . . . . . A A . A . . A . A . A A A A A A A A A A A A A A A A A A A A A A A A . A A A A A A A A A A A A A A A A A A A A A A A A A A A A A A A A A A A A A A A A A A A A A A A A A A A A A A A A A A A A A A A A A A A A A A A A A    
1065	. . . . . . . . . . . . . . . . . . . . . . . . . . . . . . . . . . . . . . . . . . . . . . . . . . . . . . . . . . . . . . . . . . . . . . . C . . . . . C C . C . . C . C . C C C C C C C C C C C C C C C C C C C C C C C C C C C C C C C C C C C C C C C C C C C C C C C C C C C C C C C C C C C C C C C C C C C C C C C C C C C C C C C C C C C C C C C C C C C C C C C C C C    
1066	. . . . . . . . . . . . . . . . . . . . . . . . . . . . . . . . . . . . . . . . . . . . . . . . . . . . . . . . . . . . . . . . . . . . . . . T . . . . . T T . T . . T . T . T T T T T T T T T T T T T T T T T T T T T T T T T T T T T T T T T T T T T T T T T T T T T T T T T T T T T T T T T T T T T T T T T T T T T T T T T T T T T T T T T T T T T T T T T T T T T T T T T T    
1067	. . . . . . . . . . . . . . . . . . . . . . . . . . . . . . . . . . . . . . . . . . . . . . . . . . . . . . . . . . . . . . . . . . . . . . . G . . . . . G G . G . . G . G . G G G G G G G G G G G G G G G G G G G G G G G G G G G G G G G G G G G G G G G G G G G G G G G G G G G G G G G G G G G G G G G G G G G G G G G G G G G G G G G G G G G G G G G G G G G G G G G G G G    
1068	. . . . . . . . . . . . . . . . . . . . . . . . . . . . . . . . . . . . . . . . . . . . . . . . . . . . . . . . . . . . . . . . . . . . . . . C . . . . . C C . C . . C . C . C C C C C C C C C C C C C C C C C C C C C C C C C C C C C C C C C C C C C C C C C C C C C C C C C C C C C C C C C C C C C C C C C C C C C C C C C C C C C C C C C C C C C C C C C C C C C C C C C C    
1069	. . . . . . . . . . . . . . . . . . . . . . . . . . . . . . . . . . . . . . . . . . . . . . . . . . . . . . . . . . . . . . . . . . . . . . . . . . . . . . T . T . . T . T . T T T T T T T T T T T T T T T T T T T T T T T T T T T T T T T T T T T T T T T T T T T T T T T T T T T T T T T T T T T T T T T T T T T T T T T T T T T T T T T T T T T T T T T T T T T T T T T T T T    
1070	. . . . . . . . . . . . . . . . . . . . . . . . . . . . . . . . . . . . . . . . . . . . . . . . . . . . . . . . . . . . . . . . . . . . . . . . . . . . . . A . A . . A . A . A A A A A A A A A A A A A A A A A A A A A A A A A A A A A A A A A A A A A A A A A A A A A A A A A A A A A A A A A A A A A A A A A A A A A A A A A A A A A A A A A A A A A A A A A A A A A A A A A A    
1071	. . . . . . . . . . . . . . . . . . . . . . . . . . . . . . . . . . . . . . . . . . . . . . . . . . . . . . . . . . . . . . . . . . . . . . . . . . . . . . A . A . . A . A . A A A A A A A A A A A A A A A A A A A A A A A A A A A A A A A A A A A A A A A A A A A A A A A A A A A A A A A A A A A A A A A A A A A A A A A A A A A A A A A A A A A A A A A A A A A A A A A A A A    
1072	. . . . . . . . . . . . . . . . . . . . . . . . . . . . . . . . . . . . . . . . . . . . . . . . . . . . . . . . . . . . . . . . . . . . . . . . . . . . . . C . C . . C . C . C C C C C C C C C C C C C C C C C C C C C C C C C C C C C C C C C C C C C C C C C C C C C C C C C C C C C C C C C C C C C C C C C C C C C C C C C C C C C C C C C C C C C C C C C C C C C C C C C C    
1073	. . . . . . . . . . . . . . . . . . . . . . . . . . . . . . . . . . . . . . . . . . . . . . . . . . . . . . . . . . . . . . . . . . . . . . . . . . . . . . C . C . . C . C . C C C C C C C C C C C C C C C C C C C C C C C C C C C C C C C C C C C C C C C C C C C C C C C C C C C C C C C C C C C C C C C C C C C C C C C C C C C C C C C C C C C C C C C C C C C C C C C C C C    
1074	. . . . . . . . . . . . . . . . . . . . . . . . . . . . . . . . . . . . . . . . . . . . . . . . . . . . . . . . . . . . . . . . . . . . . . . . . . . . . . A . A . . A . A . A A A A A A A A A A A A A A A A A A A A A A A A A A A A A A A A A A A A A A A A A A A A A A A A A A A A A A A A A A A A A A A A A A A A A A A A A A A A A A A A A A A A A A A A A A A A A A A A A A    
1075	. . . . . . . . . . . . . . . . . . . . . . . . . . . . . . . . . . . . . . . . . . . . . . . . . . . . . . . . . . . . . . . . . . . . . . . . . . . . . . T . T . . T . T . T T T T T T T T T T T T T T T T T T T T T T T T T T T T T T T T T T T T T T T T T T T T T T T T T T T T T T T T T T T T T T T T T T T T T T T T T T T T T T T T T T T T T T T T T T T T T T T T T T    
1076	. . . . . . . . . . . . . . . . . . . . . . . . . . . . . . . . . . . . . . . . . . . . . . . . . . . . . . . . . . . . . . . . . . . . . . . . . . . . . . . . A . . T . A . A A A A A A A A A A A A A A A A A A A A A A A A A A A A A A A A A A A A A A A A A A A A A A A A A A A A A A A A A A A A A A A A A A A A A A A A A A A A A A A A A A A A A A A A A A A A A A A A A A    
1077	. . . . . . . . . . . . . . . . . . . . . . . . . . . . . . . . . . . . . . . . . . . . . . . . . . . . . . . . . . . . . . . . . . . . . . . . . . . . . . . . A . . . . A . C A A A A A A A A A A A A A A A A A A A A A A A A A A A A A A A A A A A A A A A A A A A A A A A A A A A A A A A A A A A A A A A A A A A A A A A A A A A A A A A A A A A A A A A A A A A A A A A A A    
1078	. . . . . . . . . . . . . . . . . . . . . . . . . . . . . . . . . . . . . . . . . . . . . . . . . . . . . . . . . . . . . . . . . . . . . . . . . . . . . . . . A . . . . A . . A A A A A A A A A A A A A A A A A A A A A A A A A A A A A A A A A A A A A A A A A A A A A A A A A A A A A A A A A A A A A A A A A A A A A A A A A A A A A A A A A A A A A A A A A A A A A A A A A    
1079	. . . . . . . . . . . . . . . . . . . . . . . . . . . . . . . . . . . . . . . . . . . . . . . . . . . . . . . . . . . . . . . . . . . . . . . . . . . . . . . . A . . . . C . . A A A A A A A A A A A A A A A A A A A A A A A A A A A A A A A A A A A A A A A A A A A A A A A A A A A A A A A A A A A A A A A A A A A A A A A A A A A A A A A A A A A A A A A A A A A A A A A A A    
1080	. . . . . . . . . . . . . . . . . . . . . . . . . . . . . . . . . . . . . . . . . . . . . . . . . . . . . . . . . . . . . . . . . . . . . . . . . . . . . . . . . . . . . . . . C C C C C C C C C C C C C C C C C C C C C C C C C C C C C C C C C C C C C C C C C C C C C C C C C C C C C C C C C C C C C C C C C C C C C C C C C C C C C C C C C C C C C C C C C C C C C C C C C    
1081	. . . . . . . . . . . . . . . . . . . . . . . . . . . . . . . . . . . . . . . . . . . . . . . . . . . . . . . . . . . . . . . . . . . . . . . . . . . . . . . . . . . . . . . . A A A A A A A A A A A A A A A A A A A A A A A A A A A A A A A A A A A A A A A A A A A A A A A A A A A A A A A A A A A A A A A A A A A A A A A A A A A A A A A A A A A A A A A A A A A A A A A A A    
1082	. . . . . . . . . . . . . . . . . . . . . . . . . . . . . . . . . . . . . . . . . . . . . . . . . . . . . . . . . . . . . . . . . . . . . . . . . . . . . . . . . . . . . . . . G G G G G G G G G G G G G G G G G G G G G G G G G G G G G G G G G G G G G G G G G G G G G G G G G G G G G G G G G G G G G G G G G G G G G G G G G G G G G G G G G G G G G G G G G G G G G G G G G    
1083	. . . . . . . . . . . . . . . . . . . . . . . . . . . . . . . . . . . . . . . . . . . . . . . . . . . . . . . . . . . . . . . . . . . . . . . . . . . . . . . . . . . . . . . . A A A A A A A A A A A A A A A A A A A A A A A A A A A A A A A A A A A A A A A A A A A A A A A A A A A A A A A A A A A A A A A A A A A A A A A A A A A A A A A A A A A A A A A A A A A A A A A A A    
1084	. . . . . . . . . . . . . . . . . . . . . . . . . . . . . . . . . . . . . . . . . . . . . . . . . . . . . . . . . . . . . . . . . . . . . . . . . . . . . . . . . . . . . . . . T T T T T T T T T T T T T T T T T T T T T T T T T T T T T T T T T T T T T T T T T T T T T T T T T T T T T T T T T T T T T T T T T T T T T T T T T T T T T T T T T T T T T T T T T T T T T T T T T    
1085	. . . . . . . . . . . . . . . . . . . . . . . . . . . . . . . . . . . . . . . . . . . . . . . . . . . . . . . . . . . . . . . . . . . . . . . . . . . . . . . . . . . . . . . . T T . T T T T T T T T T T T T T T T T T T T T T T T T T T T T T T T T T T T T T T T T T T T T T T T T T T T T T T T T T T T T T T T T T T T T T T T T T T T T T T T T T T T T T T T T T T T T T T    
1086	. . . . . . . . . . . . . . . . . . . . . . . . . . . . . . . . . . . . . . . . . . . . . . . . . . . . . . . . . . . . . . . . . . . . . . . . . . . . . . . . . . . . . . . . A A . A A A A A A A A A A A A A A A A A A A A A A A A A A A A A A A A A A A A A A A A A A A A A A A A A A A A A A A A A A A A A A A A A A A A A A A A A A A A A A A A A A A A A A A A A A A A A A    
1087	. . . . . . . . . . . . . . . . . . . . . . . . . . . . . . . . . . . . . . . . . . . . . . . . . . . . . . . . . . . . . . . . . . . . . . . . . . . . . . . . . . . . . . . . A G . G G G G G G G G G G G G G G G G G G G G G G G G G G G G G G G G G G G G G G G G G G G G G G G G G G G G G G G G G G G G G G G G G G G G G G G G G G G G G G G G G G G G G G G G G G G G G G    
1088	. . . . . . . . . . . . . . . . . . . . . . . . . . . . . . . . . . . . . . . . . . . . . . . . . . . . . . . . . . . . . . . . . . . . . . . . . . . . . . . . . . . . . . . . . . . G G G G G G G G G G G G G G G G G G G G G G G G G G G G G G G G G G G G G G G G G G G G G G G G G G G G G G G G G G G G G G G G G G G G G G G G G G G G G G G G G G G G G G G G G G G G G G    
1089	. . . . . . . . . . . . . . . . . . . . . . . . . . . . . . . . . . . . . . . . . . . . . . . . . . . . . . . . . . . . . . . . . . . . . . . . . . . . . . . . . . . . . . . . . . . C C C C C C C C C C C C C C C C C C C C C C C C C C C C C C C C C C C C C C C C C C C C C C C C C C C C C C C C C C C C C C C C C C C C C C C C C C C C C C C C C C C C C C C C C C C C C C    
1090	. . . . . . . . . . . . . . . . . . . . . . . . . . . . . . . . . . . . . . . . . . . . . . . . . . . . . . . . . . . . . . . . . . . . . . . . . . . . . . . . . . . . . . . . . . . C C C C C C C C C C C C C C C C C C C C C C C C C C C C C C C C C C C C C C C C C C C C C C C C C C C C C C C C C C C C C C C A C C C C C C C C C C C C C C C C C C C C C C C C C C C C C C    
1091	. . . . . . . . . . . . . . . . . . . . . . . . . . . . . . . . . . . . . . . . . . . . . . . . . . . . . . . . . . . . . . . . . . . . . . . . . . . . . . . . . . . . . . . . . . . A A A A A A A A A A A A A A A A A A A A A A A A A A A A A A A A A A A A A A A A A A A A A A A A A A A A A A A A A A A A A A A . A A A A A A A A A A A A A A A A A A A A A A A A A A A A A A    
1092	. . . . . . . . . . . . . . . . . . . . . . . . . . . . . . . . . . . . . . . . . . . . . . . . . . . . . . . . . . . . . . . . . . . . . . . . . . . . . . . . . . . . . . . . . . . A A A A A A A A A A A A A A A A A A A A A A A A A A A A A A A A A A A A A A A A A A A A A A A A A A A A A A A A A A A A A A A . A A A A A A A A A A A A A A A A A A A A A A A A A A A A A A    
1093	. . . . . . . . . . . . . . . . . . . . . . . . . . . . . . . . . . . . . . . . . . . . . . . . . . . . . . . . . . . . . . . . . . . . . . . . . . . . . . . . . . . . . . . . . . . C C C C C C C C C C C C C C C C C C C C C C C C C C C C C C C C C C C C C C C C C C C C C C C C C C C C C C C C C C C C C C C . C C C C C C C C C C C C C C C C C C C C C C C C C C C C C C    
1094	. . . . . . . . . . . . . . . . . . . . . . . . . . . . . . . . . . . . . . . . . . . . . . . . . . . . . . . . . . . . . . . . . . . . . . . . . . . . . . . . . . . . . . . . . . . C C C C C C C C C C C C C C C C C C C C C C C C C C C C C C C C C C C C C C C C C C C C C C C C C C C C C C C C C C C C C C C . C C C C C C C C C C C C C C C C C C C C C C C C C C C C C C    
1095	. . . . . . . . . . . . . . . . . . . . . . . . . . . . . . . . . . . . . . . . . . . . . . . . . . . . . . . . . . . . . . . . . . . . . . . . . . . . . . . . . . . . . . . . . . . A A A A A A A A A A A A A A A A A A A A A A A A A A A A A A A A A A A A A A A A A A A A A A A A A A A A A A A A A A A A A A A . A A A A A A A A A A A A A A A A A A A A A A A A A A A A A A    
1096	. . . . . . . . . . . . . . . . . . . . . . . . . . . . . . . . . . . . . . . . . . . . . . . . . . . . . . . . . . . . . . . . . . . . . . . . . . . . . . . . . . . . . . . . . . . A A A A A A A A A A A A A A A A A A A A A A A A A A A A A A A A A A A A A A A A A A A A A A A A A A A A A A A A A A A A A A A . A A A A A A A A A A A A A A A A A A A A A A A A A A A A A A    
1097	. . . . . . . . . . . . . . . . . . . . . . . . . . . . . . . . . . . . . . . . . . . . . . . . . . . . . . . . . . . . . . . . . . . . . . . . . . . . . . . . . . . . . . . . . . . G G G G G G G G G G G G G G G G G G G G G G G G G G G G G G G G G G G G G G G G G G G G G G G G G G G G G G G G G G G G G G G . G G G G G G G G G G G G G G G G G G G G G G G G G G G G G G    
1098	. . . . . . . . . . . . . . . . . . . . . . . . . . . . . . . . . . . . . . . . . . . . . . . . . . . . . . . . . . . . . . . . . . . . . . . . . . . . . . . . . . . . . . . . . . . G G G G G G G G G G G G G G G G G G G G G G G G G G G G G G G G G G G G G G G G G G G G G G G G G G G G G G G G G G G G G G G . G G G G G G G G G G G G G G G G G G G G G G G G G G G G G G    
1099	. . . . . . . . . . . . . . . . . . . . . . . . . . . . . . . . . . . . . . . . . . . . . . . . . . . . . . . . . . . . . . . . . . . . . . . . . . . . . . . . . . . . . . . . . . . T T T T T T T T T T T T T T T T T T T T T T T T T T T T T T T T T T T T T T T T T T T T T T T T T T T T T T T T T T T T T T T . T T T T T T T T T T T T T T T T T T T T T T T T T T T T T T    
1100	. . . . . . . . . . . . . . . . . . . . . . . . . . . . . . . . . . . . . . . . . . . . . . . . . . . . . . . . . . . . . . . . . . . . . . . . . . . . . . . . . . . . . . . . . . . G G G G G G G G G G G G G G G G G G G G G G G G G G G G G G G G G G G G G G G G G G G G G G G G G G G G G G G G G G G G G G G . G G G G G G G G G G G G G G G G G G G G G G G G G G G G G G    
1101	. . . . . . . . . . . . . . . . . . . . . . . . . . . . . . . . . . . . . . . . . . . . . . . . . . . . . . . . . . . . . . . . . . . . . . . . . . . . . . . . . . . . . . . . . . . T T T T T T T T T T T T T T T T T T T T T T T T T T T T T T T T T T T T T T T T T T T T T T T T T T T T T T T T T T T T T T T . T T T T T T T T T T T T T T T T T T T T T T T T T T T T T T    
1102	. . . . . . . . . . . . . . . . . . . . . . . . . . . . . . . . . . . . . . . . . . . . . . . . . . . . . . . . . . . . . . . . . . . . . . . . . . . . . . . . . . . . . . . . . . . A A A A A A A A A A A A A A A A A A A A A A A A A A A A A A A A A A A A A A A A A A A A A A A A A A A A A A A A A A A A A A A . A A A A A A A A A A A A A A A A A A A A A A A A A A A A A A    
1103	. . . . . . . . . . . . . . . . . . . . . . . . . . . . . . . . . . . . . . . . . . . . . . . . . . . . . . . . . . . . . . . . . . . . . . . . . . . . . . . . . . . . . . . . . . . A A A A A A A A A A A A A A A A A A A A A A A A A A A A A A A A A A A A A A A A A A A A A A A A A A A A A A A A A A A A A A A . A A A A A A A A A A A A A A A A A A A A A A A A A A A A A A    
1104	. . . . . . . . . . . . . . . . . . . . . . . . . . . . . . . . . . . . . . . . . . . . . . . . . . . . . . . . . . . . . . . . . . . . . . . . . . . . . . . . . . . . . . . . . . . C C C C C C C C C C C C C C C C C C C C C C C C C C C C C C C C C C C C C C C C C C C C C T C C C C C C C C C C C C C C C C C . C C C C C C C C C C C C C C C C C C C C C C C C C C C C C C    
1105	. . . . . . . . . . . . . . . . . . . . . . . . . . . . . . . . . . . . . . . . . . . . . . . . . . . . . . . . . . . . . . . . . . . . . . . . . . . . . . . . . . . . . . . . . . . T T T T T T T T T T T T T T T T T T T T T T T T T T T T T T T T T T T T T T T T T T T T T . T T T T T T T T T T T T T T T T T . T T T T T T T T T T T T T T T T T T T T T T T T T T T T T T    
1106	. . . . . . . . . . . . . . . . . . . . . . . . . . . . . . . . . . . . . . . . . . . . . . . . . . . . . . . . . . . . . . . . . . . . . . . . . . . . . . . . . . . . . . . . . . . G G G G G G G G G G G G G G G G G G G G G G G G G G G G G G G G G G G G G G G G G G G G G . G G G G G G G G G G G G G G G G G . G G G G G G G G G G G G G G G G G G G G G G G G G G G G G G    
1107	. . . . . . . . . . . . . . . . . . . . . . . . . . . . . . . . . . . . . . . . . . . . . . . . . . . . . . . . . . . . . . . . . . . . . . . . . . . . . . . . . . . . . . . . . . . T T T T T T T T T T T T T T T T T T T T T T T T T T T T T T T T T T T T T T T T T T T T T . T T T T T T T T T T T T T T T T T . T T T T T T T T T T T T T T T T T T T T T T T T T T T T T T    
1108	. . . . . . . . . . . . . . . . . . . . . . . . . . . . . . . . . . . . . . . . . . . . . . . . . . . . . . . . . . . . . . . . . . . . . . . . . . . . . . . . . . . . . . . . . . . C C C C C C C C C C C C C C C C C C C C C C C C C C C C C C C C C C C C C C C C C C C C C . C C C C C C C C C C C C C C C C C . C C C C C C C C C C C C C C C C C C C C C C C C C C C C C C    
1109	. . . . . . . . . . . . . . . . . . . . . . . . . . . . . . . . . . . . . . . . . . . . . . . . . . . . . . . . . . . . . . . . . . . . . . . . . . . . . . . . . . . . . . . . . . . A A A A A A A A A A A A A A A A A A A A A A A A A A A A A A A A A A A A A A A A A A A A A . A A A A A A A A A A A A A A A A A . A A A A A A A A A A A A A A A A A A A A A A A A A A A A A A    
1110	. . . . . . . . . . . . . . . . . . . . . . . . . . . . . . . . . . . . . . . . . . . . . . . . . . . . . . . . . . . . . . . . . . . . . . . . . . . . . . . . . . . . . . . . . . . T T T T T T T . T T T T T T T T T T T T T T T T T T T T T T T T T T T T T T T T T T T T T . T T T T T T T T T T T T T T T T T . T T T T T T T T T T T T T T T T T T T T T T T T T T T T T T    
1111	. . . . . . . . . . . . . . . . . . . . . . . . . . . . . . . . . . . . . . . . . . . . . . . . . . . . . . . . . . . . . . . . . . . . . . . . . . . . . . . . . . . . . . . . . . . T T T T T T T . T T T T T T T T T T T T T T T T T T T T T T T T T T T T T T T T T T T T T . T T T T T T T T T T T T T T T T T . T T T T T T T T T T T T T T T T T T T T T T T T T T T T T T    
1112	. . . . . . . . . . . . . . . . . . . . . . . . . . . . . . . . . . . . . . . . . . . . . . . . . . . . . . . . . . . . . . . . . . . . . . . . . . . . . . . . . . . . . . . . . . . C C C C C C C . C T C C C C C C C C C C C C C T C C C C C C C C C C C C C C C C C C C C C . C C C C C C C C C C C C C C C C C . C C C C C C C C C C C C C C C C C C C C C C C C C C C C C C    
1113	. . . . . . . . . . . . . . . . . . . . . . . . . . . . . . . . . . . . . . . . . . . . . . . . . . . . . . . . . . . . . . . . . . . . . . . . . . . . . . . . . . . . . . . . . . . T T T T T T T . T . T T T T T T T T T T T C T . T T T T T T T T T T T T T T . T T T T T T . T T T T T T T T T T T T T T T T T . T T T T T T T T T T T T T T T T T T T T T T T T T T T T T T    
1114	. . . . . . . . . . . . . . . . . . . . . . . . . . . . . . . . . . . . . . . . . . . . . . . . . . . . . . . . . . . . . . . . . . . . . . . . . . . . . . . . . . . . . . . . . . . A A A A A A A . A . A A A A A A A A A A A . A . A A A A A A A A A A A A A A . A A A A A A . A A A A A A A A A A A T A A A A A . A A A A A A A A A A A A A A A A A A A A A A A A A A A A A A    
1115	. . . . . . . . . . . . . . . . . . . . . . . . . . . . . . . . . . . . . . . . . . . . . . . . . . . . . . . . . . . . . . . . . . . . . . . . . . . . . . . . . . . . . . . . . . . G G G G G G G . G . G G G G G G G G G G G . G . G G G G G G G G G G G G G G . G G G G G G . G G G G G G G . G G G . G G G . G . G G G G G G G G G G G G G G G G G G G G G G G G G G G G G G    
1116	. . . . . . . . . . . . . . . . . . . . . . . . . . . . . . . . . . . . . . . . . . . . . . . . . . . . . . . . . . . . . . . . . . . . . . . . . . . . . . . . . . . . . . . . . . . C C C C C C C . C . C C C C C C C C C C C . C . C C C C C C C C C C C C C C . C C C C C C . C C C C C C C . C C C . C C C . C . C C C C C C C C C C C C C C C C C C C C C C C C C C C C C C    
1117	. . . . . . . . . . . . . . . . . . . . . . . . . . . . . . . . . . . . . . . . . . . . . . . . . . . . . . . . . . . . . . . . . . . . . . . . . . . . . . . . . . . . . . . . . . . T T T T T T T . T . T T T T T T T T T T T . T . T T T T T T T T T T T T T T . T T T T T T . T T T T T T T . T T T . T T T . T . T T T T T T T T T T T T T T T T T T T T T T T T T T T T T T    
1118	. . . . . . . . . . . . . . . . . . . . . . . . . . . . . . . . . . . . . . . . . . . . . . . . . . . . . . . . . . . . . . . . . . . . . . . . . . . . . . . . . . . . . . . . . . . A A A A A A A . A . A A A A A A A A A A A . A . A A A A A A A A A A A A A A . A A A A A A . A A A A A A A . A A A . A A A . A . A A A A A A A A A A A A A A A A A A A A A A A A A A A A A A    
1119	. . . . . . . . . . . . . . . . . . . . . . . . . . . . . . . . . . . . . . . . . . . . . . . . . . . . . . . . . . . . . . . . . . . . . . . . . . . . . . . . . . . . . . . . . . . A A A A A A A . A . A A A A A A A A A A A . A . A A A A A A A A A A A A A A . A A A A A A . A A A A A A A . A A A . A A A . A . A A A A A A A A A A A A A A A A A A A A A A A A A A A A A A    
1120	. . . . . . . . . . . . . . . . . . . . . . . . . . . . . . . . . . . . . . . . . . . . . . . . . . . . . . . . . . . . . . . . . . . . . . . . . . . . . . . . . . . . . . . . . . . T T T T T T T . T . T T T T T T T T T T T . T . T T T T T T T T T T T T T T . T T T T T T . T T T T T T T . T T T . T T T . T . T T T T T T T T T T T T T T T T T T T T T T T T T T T T T T    
1121	. . . . . . . . . . . . . . . . . . . . . . . . . . . . . . . . . . . . . . . . . . . . . . . . . . . . . . . . . . . . . . . . . . . . . . . . . . . . . . . . . . . . . . . . . . . A A A A A A A . A . A A A A A A A A A A A . A . A A A A A A A A A A A A A A . A A A A A A . A A A A A A A . A A A . A A A . A . A A A A A A A A A A A A A A A A A A A A A A A A A A A A A A    
1122	. . . . . . . . . . . . . . . . . . . . . . . . . . . . . . . . . . . . . . . . . . . . . . . . . . . . . . . . . . . . . . . . . . . . . . . . . . . . . . . . . . . . . . . . . . . A A A A A A A . A . A A A A A A A A A A A . A . A A A A A A A A A A A A A A . A A A A A A . A A A A A A A . A A A . A A A . A . A A A A A A A A A A A A A A A A A A A A A A A A A A A A A A    
1123	. . . . . . . . . . . . . . . . . . . . . . . . . . . . . . . . . . . . . . . . . . . . . . . . . . . . . . . . . . . . . . . . . . . . . . . . . . . . . . . . . . . . . . . . . . . A A A A A A A . A . A A A A A A A A A A A . A . A A A A A A A A A A A A A A . A A A A A A . A A A A A A A . A A A . A A A . A . A A A A A A A A A A A A A A A A A A A A A A A A A A A A A A    
1124	. . . . . . . . . . . . . . . . . . . . . . . . . . . . . . . . . . . . . . . . . . . . . . . . . . . . . . . . . . . . . . . . . . . . . . . . . . . . . . . . . . . . . . . . . . . A A A A A A A . A . A A A A A A A A A A A . A . A A A A A A A A A A A A A A . A A A A A A . A A A A A A A . A A A . A A A . A . A A A A A A A A A A A A A A A A A A A A A A A A A A A A A A    
1125	. . . . . . . . . . . . . . . . . . . . . . . . . . . . . . . . . . . . . . . . . . . . . . . . . . . . . . . . . . . . . . . . . . . . . . . . . . . . . . . . . . . . . . . . . . . T T T T T T T . T . T T T T T T T T T T T . T . T T T T T T T T T T T T T T . T T T T T T . T T T T T T T . T T T . T T T . T . T T T T T T T T T T T T T T T T T T T T T T T T T T T T T T    
1126	. . . . . . . . . . . . . . . . . . . . . . . . . . . . . . . . . . . . . . . . . . . . . . . . . . . . . . . . . . . . . . . . . . . . . . . . . . . . . . . . . . . . . . . . . . . A A A A A A A . A . A A A A A A A A A A A . A . A A A A A A A A A A A A A A . A A A A A A . A A A A A A A . A A A . A A A . A . A A A A A A A A A A A A A A A A A A A A A A A A A A A A A A    
1127	. . . . . . . . . . . . . . . . . . . . . . . . . . . . . . . . . . . . . . . . . . . . . . . . . . . . . . . . . . . . . . . . . . . . . . . . . . . . . . . . . . . . . . . . . . . A A A A A . A . A . A A A A A A A A A A A . A . A A A A A A A A A A A A A A . A A A A A A . A A A A A A A . A A A . A A A . A . A A A A A A A A A A A A A A A A A A A A A A A A A A A A A A    
1128	. . . . . . . . . . . . . . . . . . . . . . . . . . . . . . . . . . . . . . . . . . . . . . . . . . . . . . . . . . . . . . . . . . . . . . . . . . . . . . . . . . . . . . . . . . . A A A A A . A . A . A A A A A A A A A A A . A . A A A A A A A A A A A A A A . A A A A A A . A A A A A A A . A A A . A A A . A . A A A A A A A A A A A A A A A A A A A A A A A A A A A A A A    
1129	. . . . . . . . . . . . . . . . . . . . . . . . . . . . . . . . . . . . . . . . . . . . . . . . . . . . . . . . . . . . . . . . . . . . . . . . . . . . . . . . . . . . . . . . . . . G G G G G . G . G . G G G G G G G G G G G . G . G G G G G G G G G G G G G G . G G G G G G . G G G G G G G . G G G . G G G . G . G G G G G G G G G G G G G G G G G G G G G G G G G G G G G G    
1130	. . . . . . . . . . . . . . . . . . . . . . . . . . . . . . . . . . . . . . . . . . . . . . . . . . . . . . . . . . . . . . . . . . . . . . . . . . . . . . . . . . . . . . . . . . . T T T T T . T . T . T T T T T T T T T T T . T . T T T T T T T T T T T T T T . T T T T T T . T T T T T T T . T T T . T T T . T . T T T T T T T T T T T T T T T T T T T T T T T T T T T T T T    
1131	. . . . . . . . . . . . . . . . . . . . . . . . . . . . . . . . . . . . . . . . . . . . . . . . . . . . . . . . . . . . . . . . . . . . . . . . . . . . . . . . . . . . . . . . . . . C C C C C . C . C . C C C C C C C C C C C . C . C C C C C C C C C C C C C C . C C C C C C . C C C C C C C . C . C . C C C . C . C C C C C C C C C C C C C C C C C C C C C C C C C C C C C C    
1132	. . . . . . . . . . . . . . . . . . . . . . . . . . . . . . . . . . . . . . . . . . . . . . . . . . . . . . . . . . . . . . . . . . . . . . . . . . . . . . . . . . . . . . . . . . . . . . . . . . . . . . . . . . . . . . . . . . . . . . . . . . . A . . . . . . . . . . . . . . . . . . . . . . . . . . . . . . . . . A A A A A A A A A A A A A A A A A A A A A A A A A A A A    
1133	. . . . . . . . . . . . . . . . . . . . . . . . . . . . . . . . . . . . . . . . . . . . . . . . . . . . . . . . . . . . . . . . . . . . . . . . . . . . . . . . . . . . . . . . . . . . . . . . . . . . . . . . . . . . . . . . . . . . . . . . . . . A . . . . . . . . . . . . . . . . . . . . . . . . . . . . . . . . . A A A A A A A A A A A A A A A A A A A A A A A A A A A A    
1134	. . . . . . . . . . . . . . . . . . . . . . . . . . . . . . . . . . . . . . . . . . . . . . . . . . . . . . . . . . . . . . . . . . . . . . . . . . . . . . . . . . . . . . . . . . . . . . . . . . . . . . . . . . . . . . . . . . . . . . . . . . . A . . . . . . . . . . . . . . . . . . . . . . . . . . . . . . . . . A A A A A A A A A A A A A A A A A A A A A A A A A A A A    
1135	. . . . . . . . . . . . . . . . . . . . . . . . . . . . . . . . . . . . . . . . . . . . . . . . . . . . . . . . . . . . . . . . . . . . . . . . . . . . . . . . . . . . . . . . . . . . . . . . . . . . . . . . . . . . . . . . . . . . . . . . . . . A . . . . . . . . . . . . . . . . . . . . . . . . . . . . . . . . . A A A A A A A A A A A A A A A A A A A A A A A A A A A A    
1136	. . . . . . . . . . . . . . . . . . . . . . . . . . . . . . . . . . . . . . . . . . . . . . . . . . . . . . . . . . . . . . . . . . . . . . . . . . . . . . . . . . . . . . . . . . . . . . . . . . . . . . . . . . . . . . . . . . . . . . . . . . . T . . . . . . . . . . . . . . . . . . . . . . . . . . . . . . . . . T T T T T T T T T T T T T T T T T T T T T T T T T T T T    
1137	. . . . . . . . . . . . . . . . . . . . . . . . . . . . . . . . . . . . . . . . . . . . . . . . . . . . . . . . . . . . . . . . . . . . . . . . . . . . . . . . . . . . . . . . . . . . . . . . . . . . . . . . . . . . . . . . . . . . . . . . . . . A . . . . . . . . . . . . . . . . . . . . . . . . . . . . . . . . . A A A A A A A A A A A A A A A A A A A A A A A A A A A A    
1138	. . . . . . . . . . . . . . . . . . . . . . . . . . . . . . . . . . . . . . . . . . . . . . . . . . . . . . . . . . . . . . . . . . . . . . . . . . . . . . . . . . . . . . . . . . . . . . . . . . . . . . . . . . . . . . . . . . . . . . . . . . . . . . . . . . . . . . . . . . . . . . . . . . . . . . . . . . . . . A A A A A A A A A A A A A A A A A A A A A A A A A A A A    
1139	. . . . . . . . . . . . . . . . . . . . . . . . . . . . . . . . . . . . . . . . . . . . . . . . . . . . . . . . . . . . . . . . . . . . . . . . . . . . . . . . . . . . . . . . . . . . . . . . . . . . . . . . . . . . . . . . . . . . . . . . . . . . . . . . . . . . . . . . . . . . . . . . . . . . . . . . . . . . . A A A A A A A A A A A A A A A A A A A A A A A A A A A A    
1140	. . . . . . . . . . . . . . . . . . . . . . . . . . . . . . . . . . . . . . . . . . . . . . . . . . . . . . . . . . . . . . . . . . . . . . . . . . . . . . . . . . . . . . . . . . . . . . . . . . . . . . . . . . . . . . . . . . . . . . . . . . . . . . . . . . . . . . . . . . . . . . . . . . . . . . . . . . . . . T T T T T T T T T T T T T T T T T T T T T T T T T T T T    
1141	. . . . . . . . . . . . . . . . . . . . . . . . . . . . . . . . . . . . . . . . . . . . . . . . . . . . . . . . . . . . . . . . . . . . . . . . . . . . . . . . . . . . . . . . . . . . . . . . . . . . . . . . . . . . . . . . . . . . . . . . . . . . . . . . . . . . . . . . . . . . . . . . . . . . . . . . . . . . . G G G G G G G G G G G G G G G G G G G G G G G G G G G G    
1142	. . . . . . . . . . . . . . . . . . . . . . . . . . . . . . . . . . . . . . . . . . . . . . . . . . . . . . . . . . . . . . . . . . . . . . . . . . . . . . . . . . . . . . . . . . . . . . . . . . . . . . . . . . . . . . . . . . . . . . . . . . . . . . . . . . . . . . . . . . . . . . . . . . . . . . . . . . . . . C C C C C C C C C C C C C C C C C C C C C C C C C C C C    
1143	. . . . . . . . . . . . . . . . . . . . . . . . . . . . . . . . . . . . . . . . . . . . . . . . . . . . . . . . . . . . . . . . . . . . . . . . . . . . . . . . . . . . . . . . . . . . . . . . . . . . . . . . . . . . . . . . . . . . . . . . . . . . . . . . . . . . . . . . . . . . . . . . . . . . . . . . . . . . . C C C C C C C C C C C C C C C C C C C C C C C C C C C C    
1144	. . . . . . . . . . . . . . . . . . . . . . . . . . . . . . . . . . . . . . . . . . . . . . . . . . . . . . . . . . . . . . . . . . . . . . . . . . . . . . . . . . . . . . . . . . . . . . . . . . . . . . . . . . . . . . . . . . . . . . . . . . . . . . . . . . . . . . . . . . . . . . . . . . . . . . . . . . . . . . . . G . . G G . . . . . . . . A G G A A A A A . . A .    
1145	. . . . . . . . . . . . . . . . . . . . . . . . . . . . . . . . . . . . . . . . . . . . . . . . . . . . . . . . . . . . . . . . . . . . . . . . . . . . . . . . . . . . . . . . . . . . . . . . . . . . . . . . . . . . . . . . . . . . . . . . . . . . . . . . . . . . . . . . . . . . . . . . . . . . . . . . . . . . . . . . . . . . . . . . . . . . . C . . C C C C C . . C .    
1146	. . . . . . . . . . . . . . . . . . . . . . . . . . . . . . . . . . . . . . . . . . . . . . . . . . . . . . . . . . . . . . . . . . . . . . . . . . . . . . . . . . . . . . . . . . . . . . . . . . . . . . . . . . . . . . . . . . . . . . . . . . . . . . . . . . . . . . . . . . . . . . . . . . . . . . . . . . . . . . . . . . . . . . . . . . . . . T . . T T T T T . . T .    
1147	. . . . . . . . . . . . . . . . . . . . . . . . . . . . . . . . . . . . . . . . . . . . . . . . . . . . . . . . . . . . . . . . . . . . . . . . . . . . . . . . . . . . . . . . . . . . . . . . . . . . . . . . . . . . . . . . . . . . . . . . . . . . . . . . . . . . . . . . . . . . . . . . . . . . . . . . . . . . . . . . . . . . . . . . . . . . . G . . G G G G G . . G .    
1148	. . . . . . . . . . . . . . . . . . . . . . . . . . . . . . . . . . . . . . . . . . . . . . . . . . . . . . . . . . . . . . . . . . . . . . . . . . . . . . . . . . . . . . . . . . . . . . . . . . . . . . . . . . . . . . . . . . . . . . . . . . . . . . . . . . . . . . . . . . . . . . . . . . . . . . . . . . . . . . . . . . . . . . . . . . . . . A . . . . . A A . . . .    
1149	. . . . . . . . . . . . . . . . . . . . . . . . . . . . . . . . . . . . . . . . . . . . . . . . . . . . . . . . . . . . . . . . . . . . . . . . . . . . . . . . . . . . . . . . . . . . . . . . . . . . . . . . . . . . . . . . . . . . . . . . . . . . . . . . . . . . . . . . . . . . . . . . . . . . . . . . . . . . . . . . . . . . . . . . . . . . . A . . . . . A A . . . .    
1150	. . . . . . . . . . . . . . . . . . . . . . . . . . . . . . . . . . . . . . . . . . . . . . . . . . . . . . . . . . . . . . . . . . . . . . . . . . . . . . . . . . . . . . . . . . . . . . . . . . . . . . . . . . . . . . . . . . . . . . . . . . . . . . . . . . . . . . . . . . . . . . . . . . . . . . . . . . . . . . . . . . . . . . . . . . . . . A . . . . . A A . . . .    
1151	. . . . . . . . . . . . . . . . . . . . . . . . . . . . . . . . . . . . . . . . . . . . . . . . . . . . . . . . . . . . . . . . . . . . . . . . . . . . . . . . . . . . . . . . . . . . . . . . . . . . . . . . . . . . . . . . . . . . . . . . . . . . . . . . . . . . . . . . . . . . . . . . . . . . . . . . . . . . . . . . . . . . . . . . . . . . . T . . . . . T T . . . .    
1152	. . . . . . . . . . . . . . . . . . . . . . . . . . . . . . . . . . . . . . . . . . . . . . . . . . . . . . . . . . . . . . . . . . . . . . . . . . . . . . . . . . . . . . . . . . . . . . . . . . . . . . . . . . . . . . . . . . . . . . . . . . . . . . . . . . . . . . . . . . . . . . . . . . . . . . . . . . . . . . . . . . . . . . . . . . . . . G . . . . . G G . . . .    
1153	. . . . . . . . . . . . . . . . . . . . . . . . . . . . . . . . . . . . . . . . . . . . . . . . . . . . . . . . . . . . . . . . . . . . . . . . . . . . . . . . . . . . . . . . . . . . . . . . . . . . . . . . . . . . . . . . . . . . . . . . . . . . . . . . . . . . . . . . . . . . . . . . . . . . . . . . . . . . . . . . . . . . . . . . . . . . . A . . . . . A . . . . .    
1154	. . . . . . . . . . . . . . . . . . . . . . . . . . . . . . . . . . . . . . . . . . . . . . . . . . . . . . . . . . . . . . . . . . . . . . . . . . . . . . . . . . . . . . . . . . . . . . . . . . . . . . . . . . . . . . . . . . . . . . . . . . . . . . . . . . . . . . . . . . . . . . . . . . . . . . . . . . . . . . . . . . . . . . . . . . . . . A . . . . . A . . . . .    
1155	. . . . . . . . . . . . . . . . . . . . . . . . . . . . . . . . . . . . . . . . . . . . . . . . . . . . . . . . . . . . . . . . . . . . . . . . . . . . . . . . . . . . . . . . . . . . . . . . . . . . . . . . . . . . . . . . . . . . . . . . . . . . . . . . . . . . . . . . . . . . . . . . . . . . . . . . . . . . . . . . . . . . . . . . . . . . . A . . . . . A . . . . .    
1156	. . . . . . . . . . . . . . . . . . . . . . . . . . . . . . . . . . . . . . . . . . . . . . . . . . . . . . . . . . . . . . . . . . . . . . . . . . . . . . . . . . . . . . . . . . . . . . . . . . . . . . . . . . . . . . . . . . . . . . . . . . . . . . . . . . . . . . . . . . . . . . . . . . . . . . . . . . . . . . . . . . . . . . . . . . . . . C . . . . . C . . . . .    
1157	. . . . . . . . . . . . . . . . . . . . . . . . . . . . . . . . . . . . . . . . . . . . . . . . . . . . . . . . . . . . . . . . . . . . . . . . . . . . . . . . . . . . . . . . . . . . . . . . . . . . . . . . . . . . . . . . . . . . . . . . . . . . . . . . . . . . . . . . . . . . . . . . . . . . . . . . . . . . . . . . . . . . . . . . . . . . . A . . . . . A . . . . .    
1158	. . . . . . . . . . . . . . . . . . . . . . . . . . . . . . . . . . . . . . . . . . . . . . . . . . . . . . . . . . . . . . . . . . . . . . . . . . . . . . . . . . . . . . . . . . . . . . . . . . . . . . . . . . . . . . . . . . . . . . . . . . . . . . . . . . . . . . . . . . . . . . . . . . . . . . . . . . . . . . . . . . . . . . . . . . . . . C . . . . . C . . . . .    
1159	. . . . . . . . . . . . . . . . . . . . . . . . . . . . . . . . . . . . . . . . . . . . . . . . . . . . . . . . . . . . . . . . . . . . . . . . . . . . . . . . . . . . . . . . . . . . . . . . . . . . . . . . . . . . . . . . . . . . . . . . . . . . . . . . . . . . . . . . . . . . . . . . . . . . . . . . . . . . . . . . . . . . . . . . . . . . . T . . . . . T . . . . .    
1160	. . . . . . . . . . . . . . . . . . . . . . . . . . . . . . . . . . . . . . . . . . . . . . . . . . . . . . . . . . . . . . . . . . . . . . . . . . . . . . . . . . . . . . . . . . . . . . . . . . . . . . . . . . . . . . . . . . . . . . . . . . . . . . . . . . . . . . . . . . . . . . . . . . . . . . . . . . . . . . . . . . . . . . . . . . . . . T . . . . . T . . . . .    
1161	. . . . . . . . . . . . . . . . . . . . . . . . . . . . . . . . . . . . . . . . . . . . . . . . . . . . . . . . . . . . . . . . . . . . . . . . . . . . . . . . . . . . . . . . . . . . . . . . . . . . . . . . . . . . . . . . . . . . . . . . . . . . . . . . . . . . . . . . . . . . . . . . . . . . . . . . . . . . . . . . . . . . . . . . . . . . . T . . . . . T . . . . .
```
